# The origin of the ligand-controlled regioselectivity in Rh-catalyzed [(2 + 2) + 2] carbocyclizations: steric *vs.* stereoelectronic effects†
†Electronic supplementary information (ESI) available: Computational details, Cartesian coordinates and vibrational frequencies of all optimized structures. See DOI: 10.1039/c5sc02307f



**DOI:** 10.1039/c5sc02307f

**Published:** 2015-08-25

**Authors:** Douglas W. Crandell, Shivnath Mazumder, P. Andrew Evans, Mu-Hyun Baik

**Affiliations:** a Department of Chemistry , Indiana University , 800 E. Kirkwood Ave. , Bloomington , IN 47405 , USA . Email: mbaik@indiana.edu; b Department of Chemistry , Queen's University , 90 Bader Lane , Kingston , ON K7L 3N6 , Canada . Email: andrew.evans@chem.queensu.ca; c Department of Chemistry , Korea Advanced Institute of Science & Technology (KAIST) , Daejeon , 305-701 , South Korea; d Center for Catalytic Hydrocarbon Functionalizations , Institute for Basic Science (IBS) , Daejeon , 305-701 , South Korea

## Abstract

Density functional theory calculations demonstrate that the reversal of regiochemical outcome of the addition for substituted methyl propiolates in the rhodium-catalyzed [(2 + 2) + 2] carbocyclization with PPh_3_ and (*S*)-xyl-binap as ligands is both electronically and sterically controlled. For example, the ester functionality polarizes the alkyne π* orbital to favor overlap of the methyl-substituted terminus of the alkyne with the p_π_-orbital of the alkenyl fragment of the rhodacycle during alkyne insertion with PPh_3_ as the ligand. In contrast, the sterically demanding xyl-binap ligand cannot accommodate the analogous alkyne orientation, thereby forcing insertion to occur at the sterically preferred ester terminus, overriding the electronically preferred orientation for alkyne insertion.

## Abstract 2

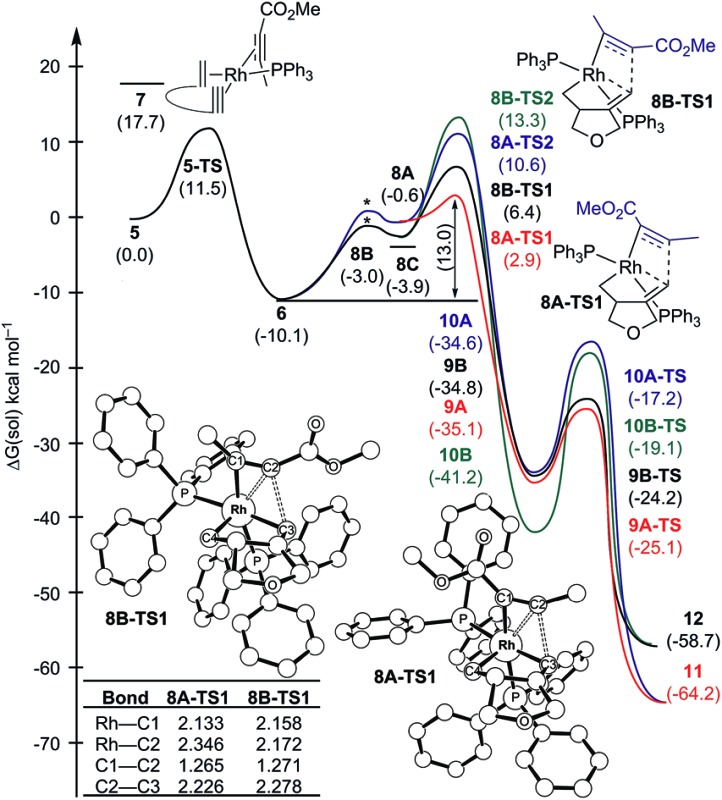
Density functional theory calculations demonstrate that the reversal of regiochemical outcome of the addition for substituted methyl propiolates in the rhodium-catalyzed [(2 + 2) + 2] carbocyclization with PPh_3_ and (*S*)-xyl-binap as ligands is both electronically and sterically controlled.

## 

Transition metal-catalyzed carbocyclization reactions are valuable transformations that permit the construction of complex polycyclic systems in an atom-economical manner.^1^ In this context, the venerable metal-catalyzed [2 + 2 + 2] reaction is particularly interesting, due to its propensity to assemble multiple π-components in a chemo-, regio-, and stereoselective fashion.^2–5^ Previously, we described the first regiodivergent intermolecular rhodium-catalyzed [(2 + 2) + 2] cycloaddition of 1,6-enynes with unsymmetrical alkynes to obtain bicyclohexa-1,3-dienes.^4,5^ A key and striking feature of that study was the ability to reverse the regioselectivity as a function of the ancillary ligand. Although the experimental findings were clear and decisive (≥19 : 1 selectivity in each case), the origin of this regiocontrol was rather speculative. A tentative proposal was that the ancillary ligand may impact the migratory insertion of an alkyl *vs.* alkenyl to an electronically biased methyl propiolate, which would result in different metallacycles that would ultimately furnish the constitutional isomers **3** and **4**, respectively. Although this explanation is entirely plausible, it does not provide a rationale as to why the nature of the ancillary ligand may direct the migratory insertion in this manner. Clearly, a more precise understanding of this insertion step is desirable for developing a concept that may allow for more rationally planning chemo-, regio-, and stereoselective carbocyclization reactions.

Herein, we present a computational study^6^ to elucidate the role of the ancillary phosphine ligands on the outcome of the rhodium-catalyzed [(2 + 2) + 2] carbocyclization of the oxygen-tethered 1,6-enyne **1** with the substituted methyl propiolate **2** (Scheme 1).

**Scheme 1:**
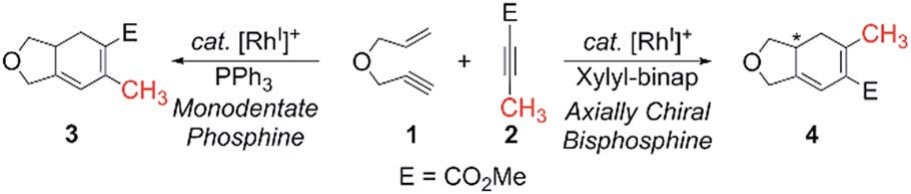


Scheme 2 and Fig. 1 summarize the proposed catalytic cycle for the process with a monodentate ligand, namely triphenylphosphine, calculated using 6-31G**/B3LYP-D3 density functional theory (see the ESI† for full computational details). Following the binding of the 1,6-enyne **1** to afford intermediate **5**, oxidative addition traversing the transition state **5-TS** at 11.5 kcal mol^–1^ generates the rhodacyclopentene **6**, which is 10.1 kcal mol^–1^ lower in solution phase Gibbs free energy than the reactant complex **5**. A second pathway was also considered, where prior to oxidative addition, one of the phosphine ligands dissociates from **5** and is replaced by an alkyne molecule to afford an alternative reactant complex **7**. However, this substitution process is unfavorable by 17.7 kcal mol^–1^ (Fig. 1), however, and renders intermediate **7** higher in energy than the transition state **5-TS**, disqualifying any reaction pathway that may be considered from **7**. For example, the oxidative coupling of the tethered ene–yne moiety to form a rhodacycle that is equivalent to **6** from **7** requires an additional 18.3 kcal mol^–1^ in activation energy.

**Scheme 2:**
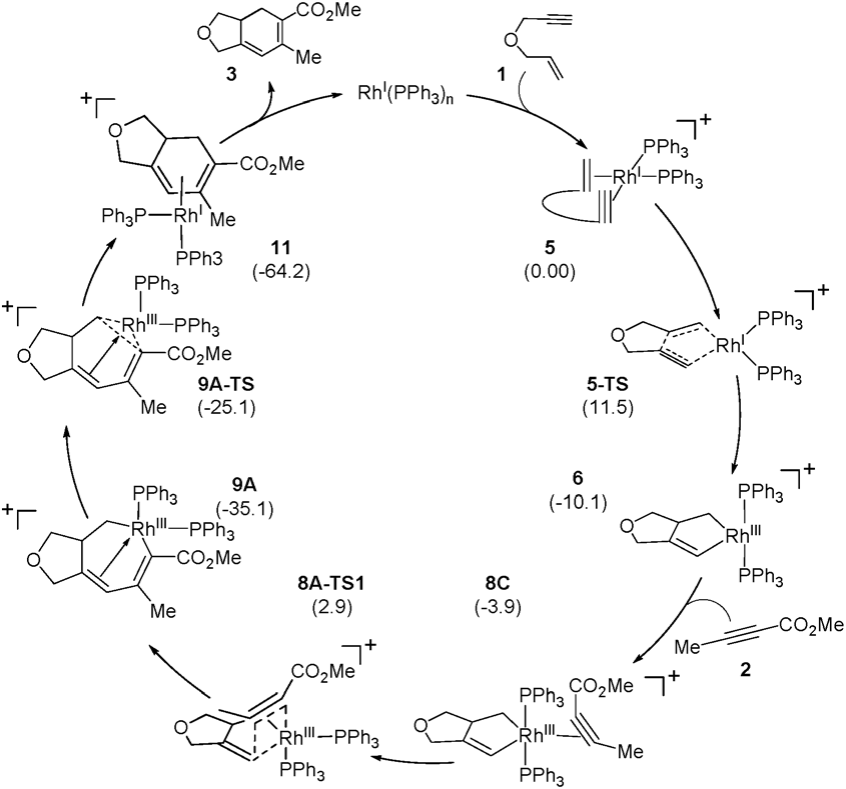


**Fig. 1:**
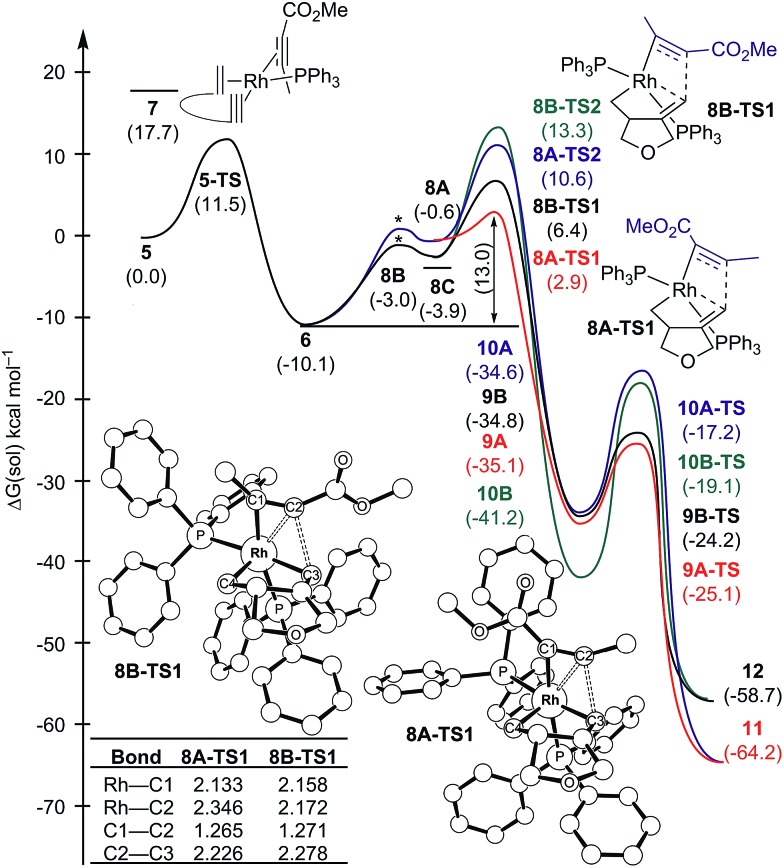
Regiodivergent reaction energy profiles for the Rh-catalyzed [(2 + 2) + 2] carbocyclization reaction with two PPh_3_ ligands on Rh. Transition states indicated by * were not explicitly located and are shown for illustration only. Inset table shows selected bond distances in Å.

The catalytic cycle continues with the binding of an alkyne molecule **2** to the rhodacyclopentene **6**, which is energetically uphill by 6.2 kcal mol^–1^ and the lowest energy structure places the two phosphine ligands in an *anti* orientation to each other, constituting the axial positions of a trigonal bipyramidal coordination geometry at the rhodium center. The alkyne substrate occupies an equatorial position in **8C**, as depicted in Scheme 2. We carefully examined the possibility of the dissociation of one of the sterically demanding phosphine ligands from **8C** and concluded after some extensive sampling of the potential energy surface, that the metal center is most effective for the insertion of the alkyne, if both phosphine groups remain bound (see the ESI† for more details). Interestingly, the insertion of the alkyne cannot take place directly from **8C**, but the phosphine ligands must be rearranged to adopt a *syn* orientation, which is easily accomplished with a barrier of 5.1 kcal mol^–1^ by a Berry-pseudo rotation of the ligands.^7^ In conformers **8A** and **8B** the two phosphine ligands adopt *syn* orientations to each other and only differ in the arrangement of the alkyne. They are also slightly higher in energy than **8C** by 3.3 and 0.9 kcal mol^–1^, respectively. At this juncture, alkyne insertion may occur either into the Rh–alkenyl or the Rh–alkyl bond of the metallacycle. Our calculations indicate in contrast to the mechanism initially postulated,^5^ that the insertion into the Rh–alkenyl bond traversing the transition state **8A-TS1** at 13.0 kcal mol^–1^ to generate the seven-membered metallacycle **9A** is most favorable with PPh_3_. The formation of **9A** is exergonic relative to **8C** by 31.2 kcal mol^–1^, suggesting that the alkyne insertion is irreversible despite the relatively low activation barrier in the forward direction. The transition state **8B-TS1** for the opposite regioisomer has a barrier of 16.5 kcal mol^–1^ and the energy difference between **8A-TS1** and **8B-TS1** is therefore 3.5 kcal mol^–1^. This result is in good agreement with the experimental outcome of **3** being the major regioisomer when PPh_3_ is used as the ligand. The C2–C3 distance of 2.226 Å between the alkyne and the alkenyl group is slightly shorter in **8A-TS1** than the distance of 2.278 Å in **8B-TS1**, as illustrated in Fig. 1, whereas Rh–C2 distance of 2.346 Å is notably longer in **8A-TS1** compared to that of 2.172 Å in **8B-TS1**. Insertion into the Rh–alkyl bond is significantly higher in energy with barriers of 20.7 and 23.4 kcal mol^–1^ through transition states **8A-TS2** and **8B-TS2**, respectively. It is reasonable to assume that the distortion energies are similar for both insertion modes, whereas the electronic interaction component will favor insertion into the Rh–alkenyl bond over the Rh–alkyl bond as a result of a stronger interaction of the p_π_ orbital of the alkenyl carbon with the alkyne substrate. The calculated activation barriers suggest that the insertion is likely rate-determining, which is surprising, as the initial oxidative addition step is assumed to be most difficult in other metal-catalyzed [2 + 2 + 2] cycloadditions.^8,9^

Given that the insertion step is responsible for the regioselectivity, it must be analyzed in greater detail: the electronic structure changes are most consistent with a heterolytic cleavage of the Rh–C(alkenyl) π-bond to form the new C(alkenyl)–C(alkyne) bond during the insertion, as illustrated in Fig. 2. In this process, electrons from the Rh–C(alkenyl) π-orbital are donated into the π* orbital of the alkyne. The presence of the electron-withdrawing ester moiety polarizes the π-bonding orbital making the MO coefficient on the α-carbon larger, whereas the π* orbital has a larger orbital coefficient on the β-carbon.^10^ Consequently, the p_π_-orbital of the alkenyl carbon in the enyne overlaps much more strongly with the π* orbital of the alkyne in **8A-TS1** compared to the alternative orientation in **8B-TS1**, which leads to a lower energy transition state in **8A-TS1**. Ultimately, this stereoelectronic effect results in C–C bond formation between the methyl-substituted rather than the ester-functionalized terminus. To determine whether this is a general finding, we located the analogous transition states for the phenyl- and isopropyl-substituted propiolates, which demonstrate similar results (Fig. S4†) that are in good accord with the experimental observations.^5^ As the alkyne moiety bends away from the metal center, the steric demands of the functional groups on the alkyne are not great enough to allow any appreciable difference when the ancillary ligand is PPh_3_.^11^

**Fig. 2:**
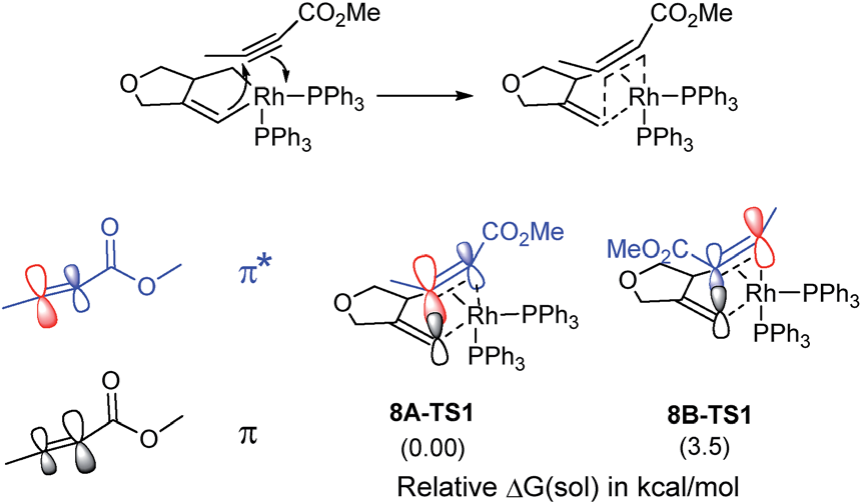
Orbital polarization during alkyne insertion with relative energies of transition states **8A-TS1** and **8B-TS1**.

The catalytic cycle completes with subsequent reductive elimination from **9A** traversing the transition state **9A-TS** with a barrier of 10.0 kcal mol^–1^ to afford the bicyclohexadiene product complex **11**, which is calculated to be 29.1 kcal mol^–1^ downhill relative to **9A**. Release of the experimentally observed regioisomer **3** affords the Rh(i)-complex that can bind new substrate and reenter the catalytic cycle. Thermodynamically, the release of **3** and binding of the enyne substrate to regenerate **5** is downhill by 4.5 kcal mol^–1^.

As mentioned above, the regiochemistry can be reversed by using the chiral bidentate (*S*)-xyl-binap instead of the monodentate PPh_3_ ligand.^5^ The computed reaction profile with (*S*)-xyl-binap is illustrated in Fig. 3. Oxidative addition of the 1,6-enyne proceeds *via* **13-TS** with a barrier of 9.6 kcal mol^–1^ to furnish the metallacycle intermediate **14** that is 4.2 kcal mol^–1^ lower in energy than the adduct **13**. Binding of the alkyne to **14** is uphill by 10.7 and 11.6 kcal mol^–1^ for **15A** and **15B**, respectively, which differ only in the orientation of the alkyne. Insertion into the Rh–alkyl bond is again found to be too high in energy with barriers of 27.9 and 31.0 kcal mol^–1^ for **15A-TS2** and **15B-TS2**, respectively (shown in blue and green in Fig. 3). Our calculations support the experimental observation that the regioselectivity is reversed from that obtained with the PPh_3_ ligand, as the transition state **15B-TS1** leading to the regioisomer **4** is associated with the lowest barrier of 18.5 kcal mol^–1^ among all the insertion transition states, as illustrated in black in Fig. 3. The transition state **15A-TS1** for the insertion *via* the methyl-substituted terminus of the alkyne, which was preferred with the monodentate PPh_3_ ligand, is 3.3 kcal mol^–1^ higher in energy compared to **15B-TS1**, mitigating the necessity to invoke the migratory insertion of the alkyl to explain the regiochemistry. Therefore, the relative energy ordering is reversed by 6.8 kcal mol^–1^ for the two insertion transition states when PPh_3_ is changed to (*S*)-xyl-binap, which correlates to a reversal of regioselectivity by a factor of ∼10 000 000. This effect can be attributed to a steric clash as the ester functionality of the alkyne is directed towards one of the xylyl groups of the binap backbone in **15A-TS1**. This steric clash is not present in **15B-TS1** and is responsible for the reversal of regiochemistry in the case of xyl-binap ligand from that seen with PPh_3_, as illustrated in Fig. 3 Thus, these two reaction pathways showcase what could be referred to as a classical example of where the regiocontrol that is mandated by the stereoelectronics of the transition state can be overridden by steric demands of the ligand scaffold that denies the access to the electronically favorable substrate arrangement.

**Fig. 3:**
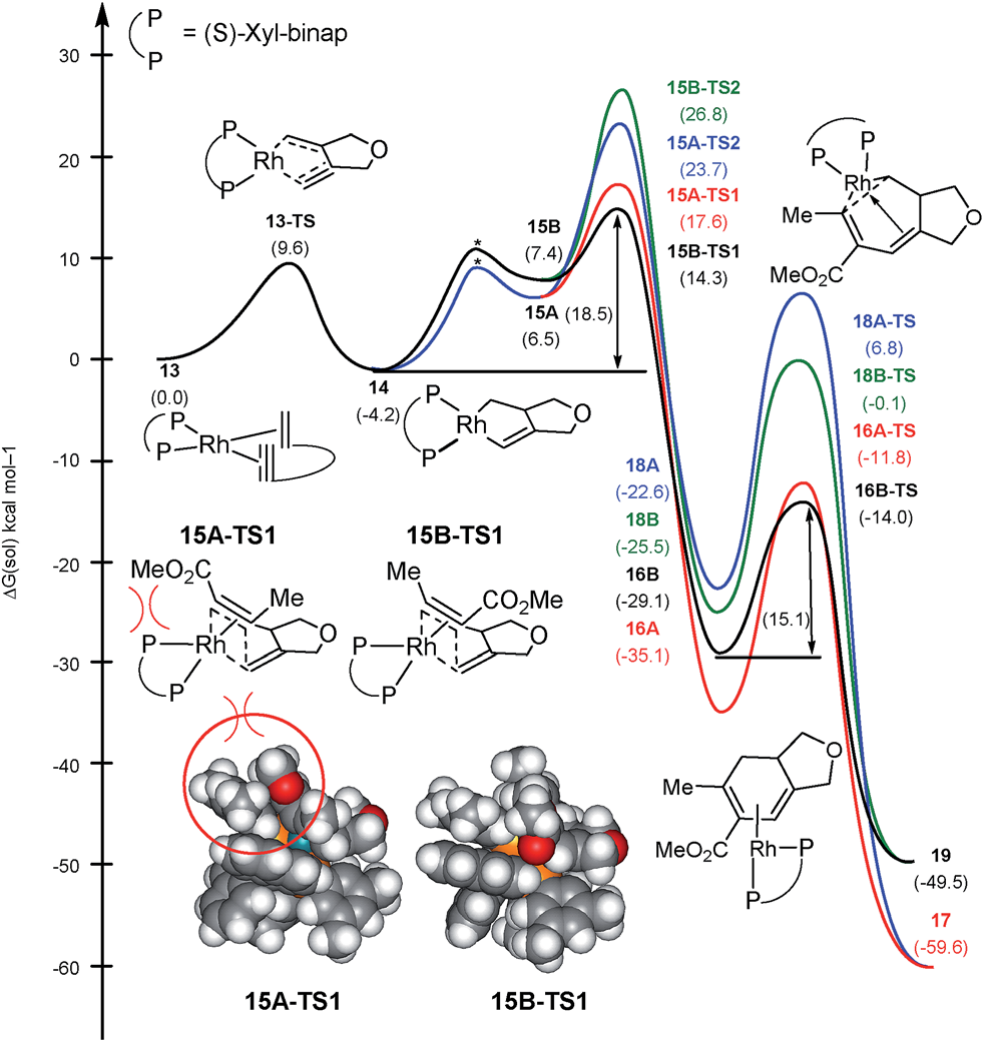
Regiodivergent reaction energy profiles for the Rh-catalyzed [(2 + 2) + 2] carbocyclization with (*S*)-xyl-binap ligand on Rh. Transition states indicated by * were not explicitly located and are shown for illustration only.

## Conclusions

In summary, the study provides a logical explanation for the regiodivergent alkyne insertion to a 1,6-enyne in the rhodium-catalyzed [(2 + 2) + 2] carbocyclization by virtue of the nature of the ligand that decorates the metal-center. We found that the PPh_3_ ligand provides access to a transition state where the electron-withdrawing ester functionality polarizes the π* orbital of the alkyne substrate such that insertion through the methyl end of the alkyne into the Rh–alkenyl bond is favored over the ester-substituted terminus. Replacing the monodentate ligand with the bulkier (*S*)-xyl-binap results in a steric clash between one of the xylyl moieties and the ester substituent forcing the insertion to happen in the opposite orientation. Hence, the strapped bidentate ligand is unable to accommodate the alkyne substrate in the orientation required for insertion *via* the most electronically favorable insertion mode and thus insertion occurs at the α-carbon of the ester-substituted alkyne. These results suggest that polar functional groups with strong inductive and resonance effects can provide powerful control over migratory insertions of alkynes in metal-catalyzed carbocyclizations and related transformations, but careful ligand design can be used to reverse these electronic biases to obtain products as desired. Our study showed that the sequence of chemical steps leading to the carbocycle is identical for both regioisomers, unlike what was previously thought.
