# Supplementary material for: The origin of the ligand-controlled regioselectivity in Rh-catalyzed [(2 + 2) + 2] carbocyclizations: steric vs. stereoelectronic effects
Source: Chem Sci. 2015 Aug 25;6(12):6896–900. doi: 10.1039/c5sc02307f (PMC5510011; doi:10.1039/c5sc02307f)
Supplement: Supplementary file 1 [file SC-006-C5SC02307F-s001.pdf]

Electronic Supplementary Material (ESI) for Chemical Science.
This journal is © The Royal Society of Chemistry 2015

**Supporting Information**

# The Origin of the Ligand-Controlled Regioselectivity in Rh-Catalyzed [(2+2)+2] Carbocyclizations: Steric vs. Stereoelectronic Effects

Douglas W. Crandell,[a] Shivnath Mazumder,[a] P. Andrew Evans[*,b] and Mu-Hyun Baik[*,a,c,d]

[a] *Department of Chemistry, Indiana University, 800 E. Kirkwood Ave., Bloomington, Indiana 47405, USA*
[b] *Department of Chemistry, Queen's University, 90 Bader Lane, Kingston, ON K7L 3N6, Canada*
[c] Department of Chemistry, Korea Advanced Institute of Science & Technology (KAIST), Daejeon, 305-701 (South Korea)
[d] Center for Catalytic Hydrocarbon Functionalizations, Institute for Basic Science (IBS), Daejeon, 305-701 (South Korea)

Email: andrew.evans@chem.queensu.ca; mbaik@indiana.edu

**Computational Details**

All calculations were carried out using DFT[1] as implemented in the Jaguar 8.1 suite of ab initio quantum chemistry programs.[2] Structures were optimized with the B3LYP[3-7] functional including Grimme's D3 dispersion correction[8] and the 6-31G** basis set. The Los Alamos LACVP[9-11] basis that includes relativistic effective core potentials was used to represent Rh. Additional single point calculations were performed on each optimized geometry using Dunning's correlation consistent triple-ζ basis set cc-pVTZ(-f)[12] that includes a double set of polarization functions to improve the electronic energy. For Rh, we used a modified version of LACVP, designated as LACV3P, in which the exponents were decontracted to match the effective core potential with triple-ζ quality. Solvation energies were evaluated by a self-consistent reaction field (SCRF)[13-15] approach based on accurate numerical solutions of the Poisson-Boltzmann equation. Solvation calculations were carried out with the 6-31G**/LACVP basis at the optimized gas-phase geometry employing the dielectric constants of $\varepsilon = 7.6$ for THF and 2.284 for benzene. Reactions using $PPh_3$ as the ligand were performed in benzene and those using xyl-binap were done in THF for solubility of the ligand.[16] As is the case for all continuum models, the solvation energies are subject to empirical parameterization of the atomic radii that are used to generate the solute surface. We employed the standard set of optimized radii in Jaguar for H (1.150 Å), C (1.900 Å), P (2.074 Å), O (1.600Å), and Rh (1.464Å). Analytical vibrational frequencies within the harmonic approximation were computed with the 6-31G**/LACVP basis to confirm that all intermediates had no imaginary frequencies except for transition states, which possessed one imaginary frequency.

The energy components have been computed with the following protocol. The change in Gibbs free energy in solution phase ΔG(Sol) has been calculated as follows:

$$\Delta G(\mathrm{Sol}) = \Delta G(\mathrm{gas}) + \Delta G^{\mathrm{solv}} \quad (1)$$
$$\Delta G(\mathrm{gas}) = \Delta H(\mathrm{gas}) - T\Delta S(\mathrm{gas}) \quad (2)$$
$$\Delta H(\mathrm{gas}) = \Delta E(\mathrm{SCF}) + \Delta \mathrm{ZPE} + \Delta C_v T + \Delta PV \quad (3)$$
$$\Delta H(\mathrm{gas}) \approx \Delta E(\mathrm{SCF}) + \Delta \mathrm{ZPE} \quad (4)$$

G(gas) is the Gibbs free energy in gas phase; G(solv) is the free energy of solvation as computed using the continuum solvation model; H(gas) is the enthalpy in gas phase; T is the temperature (333 K); S(gas) is the entropy in gas phase; E(SCF) is the self-consistent field energy, i.e. "raw" electronic energy as computed from the SCF procedure and ZPE is the zero point energy. Note that by entropy here we refer specifically to the vibrational/rotational/translational entropy of the solute(s); the entropy of the solvent is incorporated implicitly in the continuum solvation model. As the contributions from the last two terms in equation 3 are negligibly small, this equation can be essentially reduced to equation 4.

To locate transition states, the potential energy surface was first explored approximately using the linear synchronous transit (LST) method,[17] followed by a quadratic synchronous transit (QST)[18] search using the LST geometry as an initial guess.

**References**


(1) Parr, R. G.; Yang. W., *Density Functional Theory of Atoms and Molecules.* Oxford University Press: New York, 1989.
(2) Jaguar 8.1, Schrödinger, Inc., New York, NY, 2013.
(3) Slater, J.C., *Quantum Theory of Molecules and Solids, Vol. 4: The Self-Consistent Field for Molecules and Solids.* McGraw-Hill: New York, 1974.

(4) Vosko, S. H.; WIlk, L.; Nusair, M., *Can. J. Phys.* **1980**, *58,* 1200-1211.
(5) Becke, A. D., *Phys. Rev. A* **1988,** *38*, 3098-3100.
(6) Becke, A. D., *J. Chem. Phys.* **1993,** *98*, 5648-5652.
(7) Lee, C.; Yang, W.; Parr, R. G., *Phys. Rev. B* **1988,** *37*, 785-789.
(8) Grimme, S.; Antony, J.; Ehrlich, S.; Krieg, H. *J. Chem. Phys.* **2010**, *132*, 154104.
(9) Hay, P. J.; Wadt, W. R., *J. Chem. Phys.* **1985,** *82*, 270-283.
(10) Hay, P. J.; Wadt, W. R., *J. Chem. Phys.* **1985,** *82*, 299-310.
(11) Wadt, W. R.; Hay, P. J., *J. Chem. Phys.* **1985,** *82*, 284-298.
(12) Dunning, T. H., Jr., *J. Chem. Phys.* **1989,** *90*, 1007-1023.
(13) Marten, B.; Kim, K.; Cortis, C.; Friesner, R. A.; Murphy, R. B.; Ringnalda, M. N.; Sitkoff, D.; Honig, B., *J. Phys. Chem.* **1996,** *100*, 11775-11788.
(14) Friedrichs, M.; Zhou, R.; Edinger, S. R.; Friesner, R. A., *J. Phys. Chem. B* **1999,** *103*, 3057-3061.
(15) Edinger, S. R.; Cortis, C.; Shenkin, P. S.; Friesner, R. A., *J. Phys. Chem. B* **1997,** *101*, 1190-1197.
(16) Evans, P. A.; Sawyer, J. R.; Inglesby, P. A., *Angew. Chem. Int. Ed.* **2010**, *49*, 5746-5749.
(17) Halgren, T. A.; Lipscomb, W. N., *Chem. Phys. Lett.* **1977,** *49*, 225-232.
(18) Peng, C. Y. ; Schlegel, B. H. *Isr. J. Chem.* **1993**, 33, 449-454.

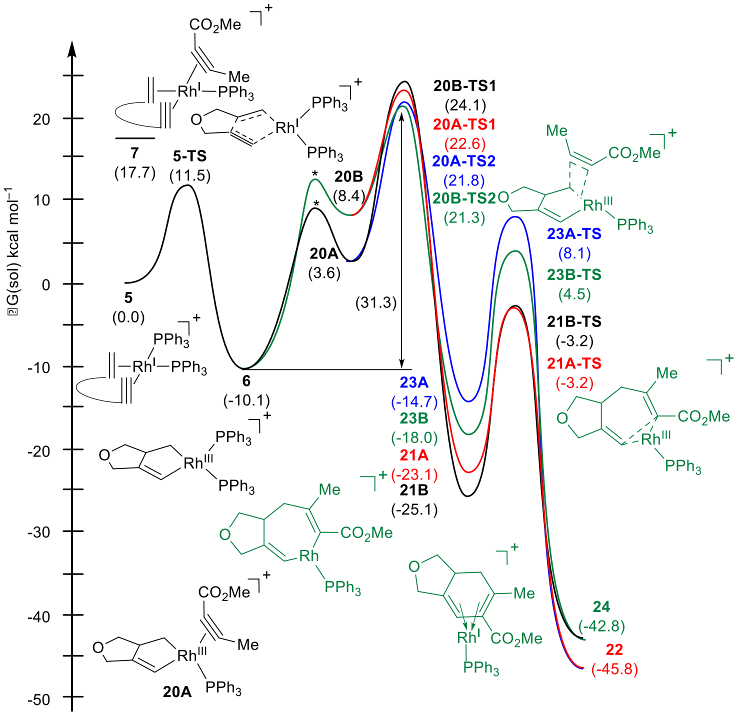


**Figure S1.** Regiodivergent reaction energy profiles for the Rh-catalyzed [2 + 2 + 2] carbocyclization with one $PPh_3$ ligand bound during alkyne insertion. Transition states indicated by * were not explicitly located and are shown for illustration only.

The reaction profile for alkyne insertion and subsequent product generation with only one monodentate phosphine ligand bound to Rh is shown in Figure S1. Our calculations indicate that the alkyne bound intermediates **20A** and **20B** with only one phosphine attached are 7.5 and 12.3 kcal $mol^{-1}$ higher in energy

than **8C**, respectively. These intermediates are still accessible under the experimental conditions and dissociation of a phosphine ligand provided a sensible hypothesis for the observed change in regioselectivity when a bulky bidentate ligand such as xyl-binap was used. The calculated activation barriers for alkyne insertion with only one phosphine ligand attached, however, are much too high to be accomplished with the lowest barrier height **20B-TS2** being 31.33 kcal mol$^{-1}$ uphill with respect to **6**. Moreover, **20B-TS2** is expected to generate the wrong regiosiomer compared to experiments with monodentate phosphine. Whereas the lowest energy pathway **20A-TS2** leading to the expected regioisomeric product is only 0.5 kcal mol$^{-1}$ higher in energy, the calculated energy differences are too small to explain the experimentally observed product ratios.

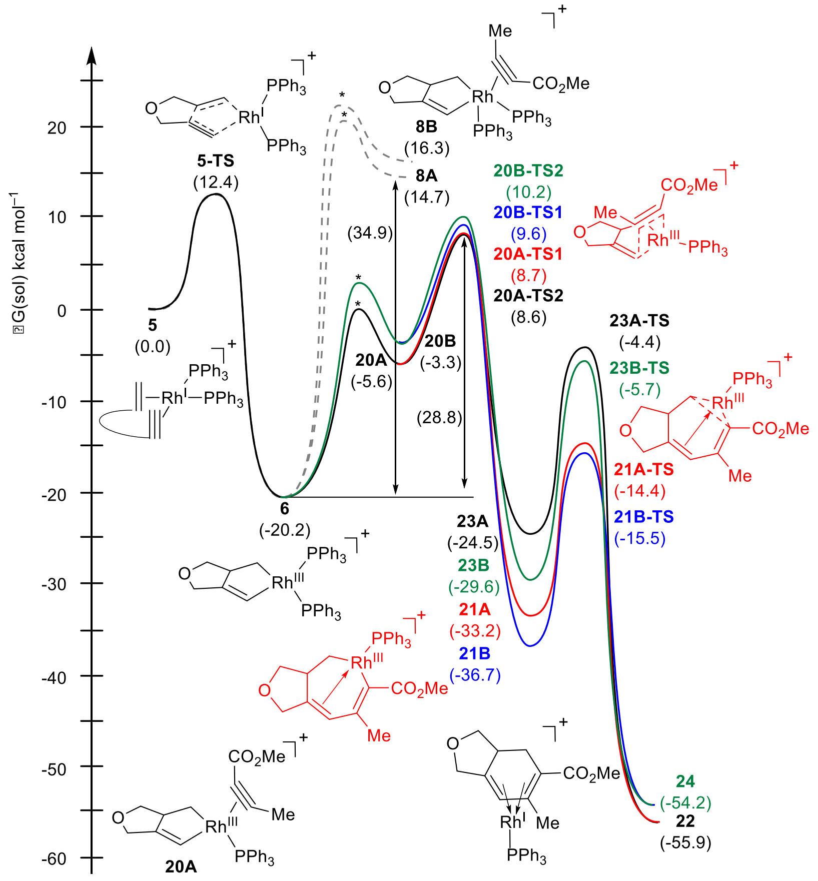


**Figure S2.** Regiodivergent reaction energy profiles for the Rh-catalyzed [2 + 2 + 2] carbocyclization calculated with B3LYP without Grimme's D3 dispersion correction.[S1] Transition states indicated by * were not explicitly located and are shown for illustration only.

When the reaction profile is computed with B3LYP without including Grimme's D3 correction to account for noncovalent interactions, dissociation of a $PPh_3$ ligand prior to alkyne binding is computed to be favorable by 5.6 kcal mol$^{-1}$. B3LYP and B3LYP-D3 give a less than 1 kcal mol$^{-1}$ energy difference (Figure 1 and Figure S1) for the barrier of **5-TS.** Intermediates **8A** and **8B** are substantially higher in energy than intermediates **20A** and **20B** where only one phosphine ligand remains bound to Rh. Surprisingly, insertion into the Rh–alkyl bond through **20A-TS2** is calculated to be essentially isoenergetic with insertion into the Rh–alkenyl bond. Transition states **20A-TS1** and **20A-TS2** both lead to the correct experimentally observed regioisomer with an activation barrier of about 28.8 kcal mol$^{-1}$.

However, when (S)-xyl-binap is used as the ancillary ligand the lowest calculated barrier to alkyne insertion increases to 37.0 kcal mol$^{-1}$. Utilizing dispersion corrections to account for noncovalent actions seems to be critical to properly assess ligand binding energies and accurately predict the correct regioisomeric products.

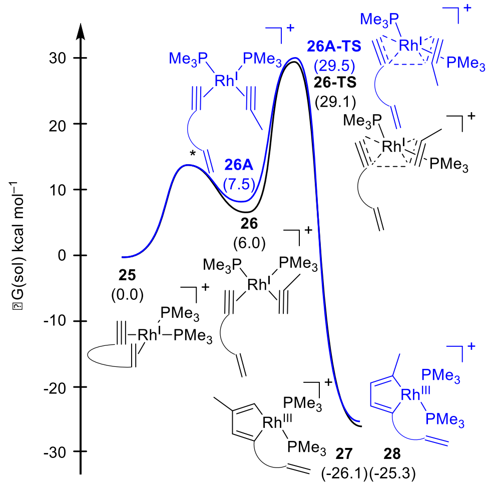


**Figure S3.** Reaction profile for coupling of alkyne of 1,6-eneyne with methyl substituted alkyne substrate to produce the rhodacyclopentadiene with a dangling tethered alkyne computed with B3LYP.

A small model was used to explore the above pathway in Figure S3, which shows coupling of the alkyne of the 1,6-eneyne with the methyl-substituted alkyne substrate. In this slightly truncated model the $PPh_3$ ligand was replaced by $PMe_3$ and the ester moiety of the alkyne substrate was replaced by hydrogen. Binding an equivalent of alkyne in place of the alkene fragment of the 1,6-eneyne is uphill by 6.0 kcal mol$^{-1}$. The oxidative addition has a barrier of 29.1 kcal mol$^{-1}$ compared to only 12.4 kcal mol$^{-1}$ for the coupling of the alkyne and alkene shown in Figure S1. As such, this pathway was not explored any further.

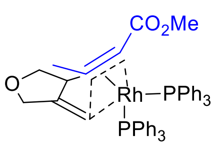

**8A-TS1**
(0.0)

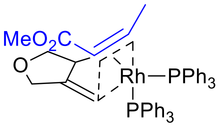

**8B-TS1**
(3.4)

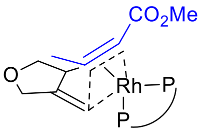

**15A-TS1**
(3.3)

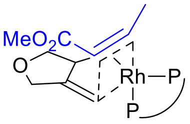

**15B-TS1**
(0.0)

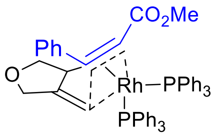

**8A-TS1'**
(0.0)

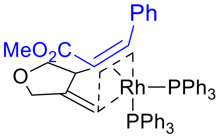

**8B-TS1'**
(3.0)

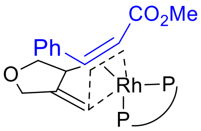

**15A-TS1'**
(1.6)

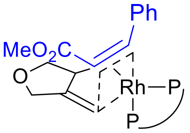

**15B-TS1'**
(0.0)

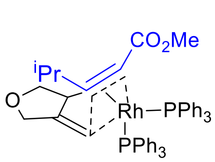

**8A-TS1''**
(0.0)

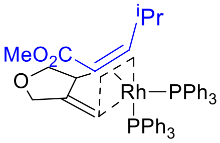

**8B-TS1''**
(2.3)

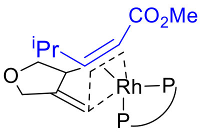

**15A-TS1''**
(1.8)

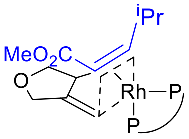

**15B-TS1''**
(0.0)

**Figure S4.** Relative energies of the key transition states using different substrates in kcal mol$^{-1}$

Unlike the ester moiety, the aryl and isopropyl fragments are able to rotate away to avoid a steric clash with the phosphine ligands bound to Rh. This flexibility of the aryl and alkyl substituents results in the change in regioselectivity being observed when the ancillary ligand is changed from L = $PPh_3$ to L = (*S*)-xyl-binap despite an increase in the steric bulk attached to the alkyne substrate.

**Table S1.** Energy components of optimized structures (L = $PPh_3$) in kcal/mol

| Structure | E(SCF) (kcal/mol) cc-pVTZ(-f) | ZPE | TS (333 K) | G (solv) ε = 2.284 | G(sol) ε = 2.284 |
|---|---|---|---|---|---|
| **1** | -216276.66 | 62.56 | 31.04 | -3.64 | -216248.79 |
| **2** | -193696.82 | 76.94 | 30.63 | -2.65 | -193653.16 |
| **$PPh_3$** | -650459.05 | 171.99 | 46.57 | -3.10 | -650336.73 |
| **3** | -410066.85 | 146.68 | 40.88 | -4.04 | -409965.09 |
| **4** | -410067.77 | 146.76 | 40.62 | -4.08 | -409965.71 |
| **5** | -1563305.48 | 426.77 | 88.09 | -21.05 | -1562987.85 |
| **5-TS** | -1563292.70 | 426.82 | 89.04 | -21.43 | -1562976.35 |
| **6** | -1129099.66 | 317.46 | 75.81 | -24.18 | -1128882.19 |
| **6-TS** | -1129081.09 | 316.66 | 75.68 | -23.82 | -1128863.94 |
| **7** | -1563313.85 | 427.03 | 89.48 | -21.60 | -1562997.90 |
| **8A** | -1779603.20 | 492.68 | 103.61 | -23.13 | -1779237.26 |
| **8B** | -1779605.27 | 492.54 | 103.51 | -23.42 | -1779239.66 |
| **8C** | -1779608.45 | 492.83 | 101.81 | -23.10 | -1779240.53 |
| **8C-BPR** | -1779601.74 | 492.14 | 103.48 | -22.40 | -1779235.48 |
| **8A-TS1** | -1779599.16 | 491.94 | 102.74 | -23.73 | -1779233.69 |
| **8A-TS2** | -1779591.57 | 491.68 | 103.29 | -22.89 | -1779226.07 |
| **8B-TS1** | -1779599.67 | 491.77 | 98.67 | -23.71 | -1779230.28 |
| **8B-TS2** | -1779589.80 | 491.59 | 102.15 | -22.93 | -1779223.29 |
| **9A** | -1779642.21 | 493.91 | 101.32 | -22.09 | -1779271.71 |
| **9B** | -1779639.10 | 494.35 | 103.62 | -23.11 | -1779271.48 |
| **11A** | -1779638.09 | 493.36 | 103.75 | -22.72 | -1779271.19 |
| **11B** | -1779648.42 | 493.80 | 101.71 | -21.55 | -1779277.88 |
| **9A-TS** | -1779632.97 | 493.98 | 100.91 | -21.88 | -1779261.78 |
| **9B-TS** | -1779633.91 | 493.83 | 98.00 | -22.76 | -1779260.84 |
| **11A-TS** | -1779623.37 | 493.28 | 101.61 | -22.17 | -1779253.88 |
| **11B-TS** | -1779624.04 | 493.32 | 103.32 | -21.74 | -1779255.78 |
| **10** | -1779677.08 | 495.97 | 97.90 | -21.78 | -1779300.79 |
| **12** | -1779671.18 | 496.16 | 98.43 | -21.85 | -1779295.30 |
| **20A** | -1129112.11 | 317.86 | 77.61 | -24.45 | -1128896.31 |
| **20B** | -1129107.71 | 317.54 | 76.64 | -24.73 | -1128891.54 |
| **20A-TS1** | -1129092.62 | 317.11 | 77.28 | -24.50 | -1128877.29 |
| **20A-TS2** | -1129095.92 | 317.63 | 76.36 | -23.47 | -1128878.12 |
| **20B-TS1** | -1129090.99 | 316.96 | 76.81 | -24.96 | -1128875.79 |
| **20B-TS2** | -1129095.58 | 317.62 | 76.60 | -24.07 | -1128878.63 |
| **21A** | -1129143.66 | 319.91 | 75.02 | -24.21 | -1128922.98 |
| **21B** | -1129143.54 | 319.58 | 76.31 | -24.70 | -1128924.97 |
| **23A** | -1129131.44 | 319.84 | 75.47 | -27.53 | -1128914.59 |
| **23B** | -1129140.38 | 320.73 | 71.77 | -26.46 | -1128917.89 |
| **21A-TS** | -1129124.40 | 319.06 | 73.99 | -23.73 | -1128903.06 |
| **21B-TS** | -1129124.15 | 319.15 | 74.02 | -24.08 | -1128903.09 |
| **23A-TS** | -1129111.30 | 318.86 | 74.86 | -24.53 | -1128891.84 |
| **23B-TS** | -1129117.25 | 319.02 | 73.57 | -23.57 | -1128895.37 |
| **22** | -1129169.53 | 321.06 | 74.19 | -23.07 | -1128945.73 |
| **24** | -1129167.34 | 321.38 | 73.70 | -23.03 | -1128942.70 |
| **25** | -841139.81 | 224.03 | 57.47 | -28.99 | -841002.24 |
| **26** | -914376.54 | 258.84 | 69.75 | -26.84 | -914214.29 |
| **26A** | -914375.27 | 258.82 | 69.56 | -26.76 | -914212.76 |
| **26-TS** | -914356.80 | 258.89 | 66.97 | -26.31 | -914191.19 |
| **26A-TS** | -914355.81 | 259.01 | 67.50 | -26.48 | -914190.78 |
| **27** | -39652.677 | 261.74 | 66.93 | -28.98 | -914246.41 |
| **28** | -39652.706 | 261.88 | 66.47 | -28.13 | -914245.63 |

**Table S2.** Energy components of optimized structures (L = Xyl-binap) in kcal/mol

| Structure | E(SCF) (kcal/mol) cc-pVTZ(-f) | ZPE | TS (333 K) | G (solv) ε = 7.6 | G(sol) ε = 7.6 |
|---|---|---|---|---|---|
| **1** | -216276.66 | 62.56 | 31.04 | -6.52 | -216251.67 |
| **13** | -1952897.71 | 609.62 | 121.97 | -31.87 | -1952441.92 |
| **13-TS** | -1952891.25 | 609.18 | 118.47 | -31.83 | -1952432.37 |
| **14** | -1952900.63 | 610.76 | 122.98 | -33.24 | -1952446.10 |
| **15A** | -2169194.75 | 675.28 | 133.53 | -34.13 | -2168687.14 |
| **15B** | -2169193.58 | 675.64 | 134.06 | -34.24 | -2168686.23 |
| **15A-TS1** | -2169184.84 | 675.70 | 132.53 | -34.34 | -2168676.01 |
| **15A-TS2** | -2169178.15 | 675.17 | 132.50 | -34.44 | -2168669.92 |
| **15B-TS1** | -2169185.90 | 675.03 | 134.75 | -33.72 | -2168679.34 |
| **15B-TS2** | -2169176.05 | 675.13 | 131.30 | -34.57 | -2168666.79 |
| **16A** | -2169239.49 | 676.82 | 131.82 | -34.18 | -2168728.67 |
| **16B** | -2169234.40 | 677.18 | 131.62 | -33.88 | -2168722.71 |
| **18A** | -2169223.44 | 676.78 | 133.35 | -36.18 | -2168716.20 |
| **18B** | -2169229.02 | 677.23 | 133.90 | -33.40 | -2168719.09 |
| **16A-TS** | -2169221.46 | 677.21 | 127.20 | -33.89 | -2168705.34 |
| **16B-TS** | -2169221.37 | 676.95 | 129.61 | -33.60 | -2168707.63 |
| **18A-TS** | -2169202.76 | 676.09 | 125.91 | -34.18 | -2168686.76 |
| **18B-TS** | -2169206.72 | 676.91 | 129.97 | -33.86 | -2168693.64 |
| **17** | -2169269.01 | 679.49 | 131.16 | -32.49 | -2168753.17 |
| **19** | -2169261.31 | 680.03 | 129.40 | -32.45 | -2168743.13 |

**Table S3.** Energy components of optimized structures with (R = $C_6H_5$ or $C_3H_7$)

| Structure | E(SCF) (kcal/mol) cc-pVTZ(-f) | ZPE | -TS (333 K) | G (solv) [a] ε = 2.284 [b] ε = 7.6 | G(sol) [a] ε = 2.284 [b] ε = 7.6 |
|---|---|---|---|---|---|
| **8A-TS1'** | -1899959.79 | 525.45 | 108.43 | -22.39[a] | -1899565.16[a] |
| **8B-TS1'** | -1899958.06 | 525.34 | 106.91 | -22.45[a] | -1899562.08[a] |
| **8A-TS1''** | -1828964.31 | 528.10 | 106.83 | -22.29[a] | -1828565.33[a] |
| **8B-TS1''** | -1828961.57 | 528.10 | 107.18 | -22.38[a] | -1828563.03[a] |
| **15A-TS1'** | -2289543.80 | 709.34 | 135.37 | -34.20[b] | -2289004.04[b] |
| **15B-TS1'** | -2289542.65 | 708.52 | 137.69 | -33.83[b] | -2289005.65[b] |
| **15A-TS1''** | -2218547.13 | 711.58 | 132.56 | -33.65[b] | -2218001.92[b] |
| **15B-TS1''** | -2218547.13 | 711.35 | 134.72 | -33.19[b] | -2218003.68[b] |

**Table S4.** Cartesian coordinates of optimized geometries

==============================

1

==============================

| | | | |
|---|---|---|---|
| C | -0.552773472 | -0.012288480 | -1.921401186 |
| C | 0.430856116 | 0.035649868 | -1.218804888 |
| C | -1.736098131 | -0.072345964 | -2.768592870 |
| H | -1.497617184 | 0.252834573 | -3.787745442 |
| H | -2.136657035 | -1.090496832 | -2.822367756 |
| H | -2.523901160 | 0.585980861 | -2.385338982 |
| C | 1.602412834 | 0.170968464 | -0.384780926 |
| O | 2.105736849 | 1.228357048 | -0.063534959 |
| O | 2.071657986 | -1.038139219 | -0.003814942 |
| C | 3.241245503 | -0.990838480 | 0.832825288 |
| H | 4.067584645 | -0.498150097 | 0.313040203 |
| H | 3.032935782 | -0.443463556 | 1.756254894 |
| H | 3.487786217 | -2.030395515 | 1.048909032 |

==============================

2

==============================

| | | | |
|---|---|---|---|
| C | -2.524300000 | -0.260700000 | -1.983900000 |
| C | -2.017800000 | -0.850000000 | -0.900400000 |
| C | 3.304800000 | -1.179100000 | 0.101800000 |
| C | 2.200100000 | -0.775100000 | -0.167900000 |
| C | 0.877800000 | -0.267800000 | -0.519500000 |
| O | -0.045700000 | -0.592300000 | 0.514100000 |
| C | -1.362500000 | -0.118400000 | 0.240600000 |
| H | 0.925800000 | 0.826300000 | -0.662200000 |
| H | 0.559400000 | -0.696000000 | -1.482900000 |
| H | -1.916900000 | -0.288900000 | 1.170400000 |
| H | -1.356600000 | 0.967200000 | 0.045800000 |
| H | -2.053700000 | -1.933800000 | -0.795800000 |
| H | 4.275600000 | -1.545300000 | 0.342000000 |
| H | -2.488500000 | 0.819000000 | -2.111500000 |
| H | -3.002200000 | -0.828600000 | -2.776300000 |

==============================

$PPh_3$

==============================

| | | | |
|---|---|---|---|
| P | -0.677200000 | -0.026500000 | -0.483300000 |
| C | 1.111100000 | -0.078100000 | -0.011000000 |
| C | 1.605200000 | 0.286000000 | 1.250800000 |
| H | 0.921500000 | 0.631900000 | 2.019700000 |
| C | 2.970900000 | 0.201400000 | 1.528400000 |
| H | 3.338800000 | 0.486900000 | 2.510400000 |
| C | 3.860700000 | -0.253400000 | 0.553900000 |
| H | 4.922700000 | -0.321200000 | 0.773400000 |
| C | 3.380400000 | -0.616200000 | -0.706300000 |
| H | 4.067300000 | -0.966800000 | -1.471700000 |
| C | 2.018200000 | -0.519800000 | -0.988200000 |
| H | 1.652900000 | -0.793700000 | -1.975200000 |
| C | -1.209500000 | -1.754100000 | -0.087500000 |
| C | -0.428300000 | -2.677600000 | 0.623500000 |
| H | 0.560700000 | -2.395100000 | 0.970200000 |
| C | -0.915700000 | -3.957800000 | 0.894400000 |
| H | -0.299200000 | -4.662300000 | 1.446500000 |
| C | -2.190400000 | -4.331200000 | 0.465900000 |
| H | -2.568700000 | -5.326800000 | 0.680600000 |
| C | -2.975700000 | -3.420700000 | -0.244700000 |
| H | -3.967400000 | -3.705500000 | -0.585600000 |
| C | -2.484700000 | -2.146500000 | -0.526400000 |
| H | -3.097900000 | -1.446600000 | -1.089300000 |
| C | -1.404900000 | 0.909100000 | 0.938000000 |
| C | -1.426300000 | 2.309800000 | 0.839900000 |
| H | -1.043500000 | 2.791100000 | -0.057100000 |
| C | -1.929900000 | 3.090500000 | 1.879900000 |
| H | -1.933400000 | 4.173400000 | 1.790300000 |
| C | -2.436900000 | 2.480700000 | 3.029400000 |
| H | -2.835700000 | 3.087100000 | 3.838000000 |
| C | -2.433100000 | 1.088700000 | 3.133000000 |
| H | -2.827800000 | 0.608200000 | 4.024300000 |
| C | -1.918300000 | 0.307100000 | 2.097000000 |
| H | -1.913500000 | -0.774300000 | 2.191600000 |

==============================

3

==============================

| | | | |
|---|---|---|---|
| C | 0.523000000 | 0.345600000 | 0.329300000 |
| C | -0.137500000 | -0.091100000 | 1.631300000 |
| C | 1.528500000 | -1.902400000 | 1.711200000 |
| C | 1.855000000 | -1.747000000 | 0.424500000 |
| C | 1.057700000 | -0.833200000 | -0.474300000 |
| C | 2.084800000 | -0.549300000 | -1.580000000 |
| C | 3.001200000 | -2.301700000 | -0.385800000 |
| O | 2.830300000 | -1.761600000 | -1.701700000 |
| H | -0.179400000 | 0.944000000 | -0.260700000 |
| H | 1.356100000 | 1.019100000 | 0.593900000 |
| H | 2.089300000 | -2.559300000 | 2.365300000 |
| H | 3.972300000 | -1.997900000 | 0.039800000 |
| H | 2.998900000 | -3.395000000 | -0.466300000 |
| H | 1.649600000 | -0.328100000 | -2.557800000 |
| H | 2.737700000 | 0.291400000 | -1.286700000 |
| H | 0.218800000 | -1.402300000 | -0.904800000 |
| C | 0.368900000 | -1.179000000 | 2.280800000 |
| C | -0.179900000 | -1.652600000 | 3.584300000 |
| C | -1.251600000 | 0.802600000 | 2.092400000 |
| H | -1.571300000 | 0.600900000 | 3.110100000 |
| H | -0.937200000 | 1.850600000 | 1.996500000 |
| H | -2.117400000 | 0.684700000 | 1.426600000 |
| O | -1.173300000 | -1.245400000 | 4.160700000 |
| O | 0.576900000 | -2.655200000 | 4.106900000 |
| C | 0.117800000 | -3.168700000 | 5.365200000 |
| H | 0.821900000 | -3.955600000 | 5.636200000 |
| H | 0.108900000 | -2.382100000 | 6.124500000 |
| H | -0.894000000 | -3.572100000 | 5.272700000 |

==============================

4

==============================

| | | | |
|---|---|---|---|
| C | 0.636769024 | 0.412923280 | 0.320490711 |
| C | -0.015420928 | 0.023534131 | 1.648308440 |
| C | 1.671439513 | -1.737511400 | 1.840061931 |
| C | 1.980559515 | -1.672892423 | 0.538838325 |
| C | 1.176083431 | -0.815518832 | -0.403263134 |
| C | 2.193207281 | -0.602918226 | -1.533738440 |
| C | 3.100857877 | -2.300387730 | -0.251197720 |
| O | 2.936819761 | -1.823909501 | -1.589625622 |
| H | -0.073298685 | 0.950412384 | -0.309102149 |
| H | 1.464261463 | 1.111394609 | 0.523851631 |
| H | 2.233145186 | -2.367126213 | 2.527232854 |
| H | 4.082019800 | -2.006944834 | 0.158054130 |
| H | 3.061519701 | -3.396571720 | -0.269797602 |
| H | 1.746272111 | -0.442071909 | -2.518013752 |
| H | 2.851300804 | 0.251752158 | -1.300443245 |
| H | 0.339454054 | -1.421190828 | -0.788240792 |
| C | 0.517340862 | -0.999669355 | 2.380152772 |

| | | | |
|---|---|---|---|
| C | 0.068316652 | -1.456478702 | 3.744664633 |
| C | -1.146050369 | 0.848017198 | 2.135663063 |
| H | -0.742560254 | -0.858236106 | 4.147905490 |
| H | -0.257245130 | -2.503592220 | 3.691533334 |
| H | 0.923399279 | -1.435896480 | 4.432868707 |
| O | -1.291917441 | 1.975168795 | 1.383628475 |
| O | -1.891640558 | 0.616133969 | 3.073641460 |
| C | -2.366757126 | 2.834847590 | 1.785395980 |
| H | -3.324263069 | 2.308782916 | 1.733395366 |
| H | -2.350642205 | 3.673051753 | 1.087900096 |
| H | -2.220881457 | 3.185163884 | 2.811182739 |

==============================

$Rh(PPh_3)_2$

==============================

| | | | |
|---|---|---|---|
| Rh | 0.437968062 | 1.626320937 | -1.317854266 |
| P | -1.311719493 | 2.737530398 | -0.277176785 |
| P | 2.009250587 | -0.061764046 | -1.576659334 |
| C | 3.311028078 | -0.122422069 | -0.302815129 |
| C | 4.060973461 | 1.045657841 | -0.086420426 |
| H | 3.851899503 | 1.939915562 | -0.669123521 |
| C | 5.069718360 | 1.064430588 | 0.874984955 |
| H | 5.650534075 | 1.969003428 | 1.033152882 |
| C | 5.327995367 | -0.078987635 | 1.637573045 |
| H | 6.108999436 | -0.062460917 | 2.392150493 |
| C | 4.584120688 | -1.242210697 | 1.427737568 |
| H | 4.786329917 | -2.131915737 | 2.017233909 |
| C | 3.581124020 | -1.269423125 | 0.457024174 |
| H | 3.006650886 | -2.177385492 | 0.299203146 |
| C | 0.830117942 | -1.420333704 | -1.225957484 |
| C | 0.345965387 | -2.264425531 | -2.238591502 |
| H | 0.783925307 | -2.228302156 | -3.231895306 |
| C | -0.695267027 | -3.153942134 | -1.969165855 |
| H | -1.057564404 | -3.808733138 | -2.756513068 |
| C | -1.269072455 | -3.207714864 | -0.695426509 |
| H | -2.078657866 | -3.902643958 | -0.492289870 |
| C | -0.789814126 | -2.371653563 | 0.318047293 |
| H | -1.226282778 | -2.408851890 | 1.312096128 |
| C | 0.249860119 | -1.480799754 | 0.058092605 |
| H | 0.612348769 | -0.833253826 | 0.850374900 |
| C | 2.822190215 | -0.424039241 | -3.165529823 |
| C | 2.226065624 | 0.054732583 | -4.343470719 |
| H | 1.305014585 | 0.632115477 | -4.290513984 |
| C | 2.810017543 | -0.208346527 | -5.582597851 |
| H | 2.344215023 | 0.164668352 | -6.489829919 |
| C | 3.997733149 | -0.940743144 | -5.652471420 |
| H | 4.456928332 | -1.139733270 | -6.616090394 |
| C | 4.598645929 | -1.411632389 | -4.481927478 |
| H | 5.524695007 | -1.976465382 | -4.535031178 |
| C | 4.016973648 | -1.156299101 | -3.240903984 |
| H | 4.492445779 | -1.519539061 | -2.334681319 |
| C | -0.557433084 | 2.052385071 | 1.243567702 |
| C | 0.833578329 | 2.289291253 | 1.389667723 |
| H | 1.324705690 | 3.055294642 | 0.788454645 |
| C | 1.568549184 | 1.598801176 | 2.362186615 |
| H | 2.638206467 | 1.761534835 | 2.444087637 |
| C | 0.922547228 | 0.707461374 | 3.215699777 |
| H | 1.487882664 | 0.179865867 | 3.977912725 |
| C | -0.460429149 | 0.497381834 | 3.097612108 |
| H | -0.962779253 | -0.184917022 | 3.777770243 |
| C | -1.194250839 | 1.144736696 | 2.107265805 |
| H | -2.254981089 | 0.943912242 | 1.994257283 |
| C | -1.500300530 | 4.529233219 | -0.057573004 |
| C | -1.479120367 | 5.350231048 | -1.196150063 |
| H | -1.323836626 | 4.914002533 | -2.179766753 |
| C | -1.662443219 | 6.725940132 | -1.066289908 |
| H | -1.649307533 | 7.358913832 | -1.948394692 |
| C | -1.857058677 | 7.287770878 | 0.197592445 |
| H | -1.996449676 | 8.360101239 | 0.297596179 |
| C | -1.870040448 | 6.475744056 | 1.334283515 |
| H | -2.020716430 | 6.914820239 | 2.315995524 |
| C | -1.693502500 | 5.098224314 | 1.211364948 |
| H | -1.707411969 | 4.467722201 | 2.095760042 |
| C | -2.971044868 | 2.011227835 | -0.445179652 |
| C | -3.057473163 | 0.630501411 | -0.706996013 |
| H | -2.156315184 | 0.023860823 | -0.762434050 |
| C | -4.302948160 | 0.030423370 | -0.872985595 |
| H | -4.364016255 | -1.036268888 | -1.067841315 |
| C | -5.466507046 | 0.800974466 | -0.792387561 |
| H | -6.437232634 | 0.333258676 | -0.928548683 |
| C | -5.383514127 | 2.171517890 | -0.539393815 |
| H | -6.288111927 | 2.769049961 | -0.477512266 |
| C | -4.140307491 | 2.780361941 | -0.364395162 |
| H | -4.080603525 | 3.846447799 | -0.167637964 |

==============================

5

==============================

| | | | |
|---|---|---|---|
| Rh | 0.458755780 | 1.145122870 | -0.499301300 |
| P | -1.443187018 | 2.432408831 | 0.234001335 |
| P | -0.567964706 | 0.183817828 | -2.445966970 |
| C | 2.579237263 | 0.532615753 | -1.176058535 |
| C | 2.038034423 | -0.579192749 | -0.569071883 |
| C | 1.573859873 | 2.331068914 | 1.040989191 |
| C | 1.242202112 | 1.274786874 | 1.590003297 |
| C | 1.082525723 | 0.074241272 | 2.433312551 |
| O | 1.273775917 | -1.132117453 | 1.702620755 |
| C | 2.401008790 | -1.076700247 | 0.820582065 |
| H | 1.801737712 | 0.139681054 | 3.265942134 |
| H | 0.071272445 | 0.043120217 | 2.849562610 |
| H | 2.761137834 | -2.106945615 | 0.732115198 |
| H | 3.209922703 | -0.476632395 | 1.257797787 |
| H | 1.548140397 | -1.321993337 | -1.190272775 |
| H | 1.959725642 | 3.312658409 | 0.856661676 |
| H | 3.278942247 | 1.165197102 | -0.637278980 |
| H | 2.554660071 | 0.648429164 | -2.253586431 |
| C | 0.599419659 | -0.686350248 | -3.577786302 |
| C | 1.377772656 | 0.082498208 | -4.460231922 |
| H | 1.255798890 | 1.162031688 | -4.492814707 |
| C | 2.287978231 | -0.532346079 | -5.316808548 |
| H | 2.878240348 | 0.071707301 | -5.999393432 |
| C | 2.434603111 | -1.922312214 | -5.303532881 |
| H | 3.141532747 | -2.401179816 | -5.974246515 |
| C | 1.664288255 | -2.692053777 | -4.433356602 |
| H | 1.766061768 | -3.773228708 | -4.425858529 |
| C | 0.749663983 | -2.079815036 | -3.573102595 |
| H | 0.145023614 | -2.692596511 | -2.912197507 |
| C | -1.693518343 | -1.173782667 | -1.910582940 |
| C | -2.806157435 | -1.589314987 | -2.656062079 |
| H | -3.079655865 | -1.076651341 | -3.569892208 |
| C | -3.599781803 | -2.646686832 | -2.210288443 |
| H | -4.460342592 | -2.953413961 | -2.797832496 |
| C | -3.297396952 | -3.302435624 | -1.014793702 |
| H | -3.918632029 | -4.123557973 | -0.670297225 |
| C | -2.194169183 | -2.891526028 | -0.263597731 |
| H | -1.952493272 | -3.388798008 | 0.671110991 |
| C | -1.396435222 | -1.834079404 | -0.704021801 |
| H | -0.560025455 | -1.513072851 | -0.088725515 |
| C | -1.445746423 | 1.250509053 | -3.655309596 |
| C | -1.607379604 | 2.619700297 | -3.419498487 |
| H | -1.227924723 | 3.061682141 | -2.509073026 |
| C | -2.242543290 | 3.435649614 | -4.357366974 |
| H | -2.359350664 | 4.495263030 | -4.150810985 |

| | | | |
|---|---|---|---|
| C | -2.719397884 | 2.892894172 | -5.549405827 |
| H | -3.215496855 | 3.525336776 | -6.279310847 |
| C | -2.534184027 | 1.531854191 | -5.811370489 |
| H | -2.877518568 | 1.104981485 | -6.748979626 |
| C | -1.891177690 | 0.719435893 | -4.881148005 |
| H | -1.706924118 | -0.322096824 | -5.124271618 |
| C | -1.743123682 | 2.181880311 | 2.041064311 |
| C | -1.050750260 | 2.955079032 | 2.988724506 |
| H | -0.397821810 | 3.757108921 | 2.665293703 |
| C | -1.197372888 | 2.707800123 | 4.353668548 |
| H | -0.658811063 | 3.322914167 | 5.068416743 |
| C | -2.030853072 | 1.680421989 | 4.799435696 |
| H | -2.143198849 | 1.487910847 | 5.861877611 |
| C | -2.728652991 | 0.911398931 | 3.866650312 |
| H | -3.392409200 | 0.118813808 | 4.199021763 |
| C | -2.588346937 | 1.158774161 | 2.500435707 |
| H | -3.155287989 | 0.554972054 | 1.803768927 |
| C | -1.061056654 | 4.221542068 | 0.075812731 |
| C | -0.037518366 | 4.638774470 | -0.789141138 |
| H | 0.556603882 | 3.895479522 | -1.315597753 |
| C | 0.227628220 | 5.997261657 | -0.970804022 |
| H | 1.020873427 | 6.305787139 | -1.645336364 |
| C | -0.522445080 | 6.954200895 | -0.285297098 |
| H | -0.315808496 | 8.010935921 | -0.424634911 |
| C | -1.537565755 | 6.547840632 | 0.585374083 |
| H | -2.121727001 | 7.288042899 | 1.123769562 |
| C | -1.807784321 | 5.191757767 | 0.765934305 |
| H | -2.599609999 | 4.886123304 | 1.443231420 |
| C | -3.109803235 | 2.241003904 | -0.524238841 |
| C | -3.676764880 | 0.960026519 | -0.605955431 |
| H | -3.138319260 | 0.096888619 | -0.238930397 |
| C | -4.930332682 | 0.771249974 | -1.185219208 |
| H | -5.341676096 | -0.231401195 | -1.243971004 |
| C | -5.633385470 | 1.858855779 | -1.702008321 |
| H | -6.606509469 | 1.712652355 | -2.161206606 |
| C | -5.076397977 | 3.136201925 | -1.631426454 |
| H | -5.613698111 | 3.988037936 | -2.037749051 |
| C | -3.826104084 | 3.329831785 | -1.046026036 |
| H | -3.408457122 | 4.329199962 | -1.016465618 |

===============================

5-TS

===============================

| | | | |
|---|---|---|---|
| C | -0.697287311 | 0.689102978 | 0.376242421 |
| C | -0.739879780 | -0.187597734 | 2.164483385 |
| Rh | 1.465772058 | 0.104814053 | 1.114809473 |
| P | 2.783380059 | -1.765780666 | 1.452468347 |
| P | 2.971531707 | 1.561346959 | 0.183405313 |
| C | 0.277587552 | 0.568431989 | -0.463583538 |
| C | 0.458913283 | -0.353427595 | 2.965544585 |
| C | -1.791736091 | 0.802155960 | 2.655014533 |
| C | -1.975575372 | 1.473522920 | 0.491036715 |
| O | -2.711696151 | 1.057120744 | 1.617019755 |
| H | 0.643286301 | -1.352972995 | 3.347725975 |
| H | 0.687137651 | 0.451515935 | 3.665536009 |
| H | 0.486658305 | 0.828164912 | -1.491524589 |
| H | -1.696589456 | 2.539288330 | 0.575357289 |
| H | -2.597228836 | 1.340480782 | -0.399259140 |
| H | -2.351808072 | 0.355274617 | 3.481357244 |
| H | -1.303413403 | 1.725270140 | 3.010753011 |
| H | -1.172746745 | -1.122315958 | 1.808193785 |
| C | 2.315269611 | 3.193222462 | 0.718492811 |
| C | 2.859747819 | 3.884021339 | 1.811074167 |
| H | 3.740229338 | 3.504635582 | 2.317788610 |
| C | 2.282617869 | 5.077944432 | 2.248568491 |
| H | 2.718570651 | 5.603384813 | 3.093239727 |
| C | 1.162746559 | 5.600221642 | 1.601242932 |
| H | 0.719379221 | 6.531024583 | 1.941512743 |
| C | 0.621764795 | 4.924436241 | 0.503126093 |
| H | -0.239433391 | 5.331980780 | -0.018525046 |
| C | 1.187890252 | 3.728469970 | 0.068565097 |
| H | 0.760229981 | 3.216024108 | -0.786091247 |
| C | 4.675929465 | 1.489488338 | 0.836788065 |
| C | 5.795588425 | 1.701419141 | 0.019134974 |
| H | 5.670842992 | 1.936692808 | -1.032130958 |
| C | 7.079341414 | 1.582656774 | 0.552392620 |
| H | 7.942431900 | 1.742360634 | -0.087433875 |
| C | 7.257551687 | 1.247267722 | 1.896754263 |
| H | 8.259721275 | 1.140930561 | 2.301870122 |
| C | 6.145836815 | 1.048782214 | 2.718875829 |
| H | 6.272821015 | 0.776364470 | 3.762325602 |
| C | 4.862143597 | 1.175178487 | 2.191390944 |
| H | 4.000755468 | 1.004542766 | 2.826561234 |
| C | 3.165180617 | 1.659093473 | -1.628996002 |
| C | 3.390045456 | 2.876200699 | -2.291249758 |
| H | 3.388543342 | 3.809549404 | -1.736725703 |
| C | 3.617135216 | 2.887940169 | -3.667909155 |
| H | 3.787809270 | 3.831964661 | -4.176934684 |
| C | 3.628370123 | 1.691549779 | -4.388455324 |
| H | 3.808912204 | 1.705373377 | -5.459429596 |
| C | 3.404465005 | 0.479137870 | -3.732527679 |
| H | 3.414553596 | -0.454978009 | -4.286298002 |
| C | 3.168291229 | 0.461019393 | -2.359258950 |
| H | 2.990421210 | -0.481822189 | -1.854624228 |
| C | 1.602895379 | -3.175836438 | 1.536088563 |
| C | 0.457703989 | -3.148851336 | 0.719424556 |
| H | 0.277077344 | -2.299214930 | 0.065156746 |
| C | -0.447425634 | -4.208766160 | 0.737360508 |
| H | -1.324182663 | -4.179049218 | 0.096852456 |
| C | -0.226489900 | -5.303354350 | 1.576568326 |
| H | -0.932744991 | -6.128101343 | 1.593167185 |
| C | 0.903260761 | -5.333422697 | 2.394146575 |
| H | 1.078927663 | -6.181335093 | 3.049801292 |
| C | 1.816431943 | -4.277602532 | 2.376235908 |
| H | 2.689597477 | -4.318026359 | 3.018352520 |
| C | 3.858686505 | -2.164697924 | 0.017780904 |
| C | 5.153358503 | -1.624945660 | -0.066465293 |
| H | 5.558885041 | -1.043366380 | 0.751724847 |
| C | 5.938855454 | -1.850183571 | -1.195186393 |
| H | 6.935672335 | -1.422063040 | -1.243565994 |
| C | 5.449775464 | -2.620629043 | -2.251294399 |
| H | 6.064837545 | -2.797101606 | -3.128808207 |
| C | 4.171537789 | -3.175402246 | -2.168168194 |
| H | 3.790739071 | -3.792533507 | -2.976878807 |
| C | 3.377987933 | -2.950068819 | -1.042765423 |
| H | 2.390435136 | -3.395197712 | -0.993700835 |
| C | 3.871179694 | -1.983247116 | 2.912988204 |
| C | 3.594511280 | -1.289674338 | 4.099871747 |
| H | 2.773034636 | -0.582979624 | 4.132021460 |
| C | 4.378813949 | -1.488513984 | 5.235460565 |
| H | 4.153797027 | -0.944173615 | 6.148042082 |
| C | 5.452599661 | -2.380563603 | 5.197361829 |
| H | 6.066873982 | -2.532005138 | 6.080148393 |

C 5.734554463 -3.077763218 4.021286607
H 6.566547132 -3.775031922 3.985838477
C 4.948062334 -2.885398432 2.885894607
H 5.177119807 -3.436708870 1.979985967
==============================
6
==============================
Rh 0.695072281 1.854162020 0.752729052
P -1.556164972 2.571997522 0.799197314
P 2.951073693 1.560153535 1.359326107
C 0.414404876 -0.081001225 0.345126117
C 0.998999379 1.998875717 -1.303222670
C 0.241536244 -0.334358020 -0.950987360
C 0.255072786 0.819347973 -1.925927230
C 0.865699912 0.118489812 -3.157068766
C 0.224027273 -1.618276165 -1.757506279
O 0.300432370 -1.191489861 -3.123149766
C -2.839165467 2.312563999 -0.464972979
C -3.611341048 3.357059191 -0.993967900
H -3.444722916 4.380581866 -0.674184289
C -4.604315774 3.078996600 -1.934415178
H -5.198387955 3.890697480 -2.344090855
C -4.836859397 1.764826344 -2.342522049
H -5.609977792 1.553443998 -3.075288498
C -4.077337465 0.720376552 -1.808374422
H -4.257944415 -0.302560486 -2.124949836
C -3.080407560 0.989503885 -0.873529436
H -2.482030526 0.179692926 -0.465079652
C -2.328157136 2.106146052 2.390084421
C -3.720684815 2.088859375 2.554982118
H -4.371548903 2.323380405 1.718212276
C -4.272043234 1.766815028 3.794796964
H -5.351207096 1.754413789 3.916154652
C -3.442444922 1.462484701 4.875916263
H -3.877438119 1.213238198 5.839360488
C -2.054701423 1.476329664 4.718023159
H -1.402032663 1.240519872 5.553222852
C -1.498563017 1.792205498 3.479828649
H -0.415050241 1.801188840 3.378661318
C -1.228118817 4.373947727 0.908191042
C -1.231111644 5.046969886 2.141559375
H -1.537800425 4.529264866 3.044931561
C -0.850036861 6.387984495 2.210643158
H -0.865609534 6.901122053 3.168320370
C -0.461428712 7.071356683 1.056928472
H -0.170381599 8.116113910 1.115077012
C -0.456314684 6.410187091 -0.175223612
H -0.166037612 6.939742292 -1.078264362
C -0.829336024 5.069254534 -0.250580648
H -0.838184280 4.569934983 -1.215577600
C 3.871344958 0.010603092 1.110106189
C 4.713096838 -0.525838091 2.097358053
H 4.817119405 -0.027745284 3.056765421
C 5.411975705 -1.707612514 1.849923880
H 6.060436459 -2.118709776 2.617927178
C 5.278594291 -2.359409377 0.621527193
H 5.822477575 -3.280259587 0.434121343
C 4.442647696 -1.827935805 -0.363800797
H 4.334407425 -2.333163182 -1.319024719
C 3.737151568 -0.650170607 -0.121540701
H 3.073294388 -0.251680267 -0.880623247
C 4.035634553 2.940892758 0.867326731
C 5.433458940 2.847238816 0.933111863
H 5.903701982 1.918862719 1.243237754
C 6.220555675 3.947083249 0.595588393
H 7.302534878 3.870746482 0.647353119
C 5.621773771 5.142947798 0.192323524
H 6.239674779 5.997042335 -0.068672735
C 4.230820313 5.240458420 0.118071513
H 3.762873419 6.167271404 -0.200678373
C 3.438547073 4.141714607 0.447402671
H 2.355588595 4.225157864 0.376965024
C 2.668984640 1.727929852 3.160760227
C 2.808547370 2.965570458 3.810569550
H 3.224368483 3.816079971 3.278967603
C 2.419410356 3.103165679 5.143122838
H 2.537448469 4.061194607 5.640937607
C 1.888102356 2.014495960 5.838446299
H 1.590200492 2.126087726 6.876907596
C 1.747566553 0.779479288 5.199220258
H 1.342743916 -0.072126858 5.738444597
C 2.128067534 0.635575845 3.865527656
H 2.019149135 -0.327813597 3.374732995
H 0.643032832 2.979886159 -1.627014072
H 2.078122938 1.942561302 -1.464346980
H 0.515878258 -0.810865438 1.148433610
H 1.079109907 -2.261320068 -1.494409985
H -0.693158313 -2.206973556 -1.638403006
H 0.601567659 0.572231209 -4.115580427
H 1.966887285 0.085142295 -3.074173439
H -0.775728319 1.106805756 -2.166395166
==============================
7
==============================
C 0.063412650 -0.022888497 -0.122268666
C 0.113152963 -0.208497282 2.389009515
Rh 1.754471528 0.016279920 1.026311535
P 4.163703732 0.000656401 1.370941884
C 1.753397646 2.060552467 0.673660090
C -0.204119896 1.202376656 -0.583692305
C 0.413094827 2.420247516 0.072123710
C 0.395289178 3.409229520 -1.121784500
C -0.949912980 1.681204539 -1.818253906
O -0.819644132 3.100034244 -1.792466842
H 2.010026310 2.564408361 1.604468813
H 2.572293006 2.162725525 -0.047764656
H -0.405078261 -0.954636485 -0.433236778
H -0.504915495 1.253244422 -2.729939039
H -2.014617696 1.425615081 -1.802336270
H 0.366910212 4.460319322 -0.828913755
H 1.271348402 3.238295977 -1.770633370
H -0.276442747 2.796139030 0.839883781
C 1.234505778 -0.033333724 2.975293764
C -1.345774104 -0.407835109 2.449370972
C 1.966866640 0.116638837 4.244383780
H -1.875792232 0.485014450 2.104742799
H -1.661484259 -1.245637156 1.821176144
H -1.628578506 -0.613828594 3.487191657
O 2.849306513 0.929136062 4.427546184
O 1.532527946 -0.768494151 5.143386599
C 2.229224130 -0.746303827 6.416272557
H 1.745561236 -1.512552289 7.019693590

| | | | |
|---|---|---|---|
| H | 3.284739797 | -0.979120129 | 6.258548895 |
| H | 2.133591357 | 0.236605361 | 6.882055185 |
| C | 4.750316187 | -0.980879148 | 2.790239747 |
| C | 5.825225333 | -0.562144851 | 3.584177713 |
| H | 6.338189883 | 0.367499786 | 3.363083676 |
| C | 6.230577800 | -1.337399887 | 4.671144997 |
| H | 7.063930229 | -1.007192555 | 5.283692240 |
| C | 5.570060881 | -2.529612709 | 4.970671816 |
| H | 5.891278506 | -3.131481214 | 5.815452658 |
| C | 4.495219258 | -2.950279502 | 4.182249686 |
| H | 3.978144842 | -3.876739750 | 4.412506123 |
| C | 4.082756920 | -2.177461100 | 3.099129114 |
| H | 3.244170651 | -2.509800857 | 2.492233064 |
| C | 4.708080387 | -0.892856455 | -0.131761309 |
| C | 4.256687096 | -0.425917526 | -1.381244179 |
| H | 3.649756004 | 0.474504897 | -1.444604808 |
| C | 4.613570362 | -1.090113945 | -2.553205653 |
| H | 4.267454759 | -0.716510467 | -3.512028457 |
| C | 5.419816517 | -2.229384168 | -2.491893559 |
| H | 5.699220815 | -2.747183729 | -3.404002503 |
| C | 5.870383643 | -2.697038516 | -1.255964456 |
| H | 6.504472768 | -3.576975983 | -1.205873049 |
| C | 5.517081744 | -2.036648618 | -0.078596650 |
| H | 5.877007001 | -2.406273435 | 0.875747417 |
| C | 5.071192246 | 1.582504730 | 1.383992056 |
| C | 4.732242513 | 2.545991777 | 2.350426120 |
| H | 3.964858298 | 2.328708273 | 3.085480041 |
| C | 5.408790962 | 3.763565605 | 2.382591929 |
| H | 5.148872508 | 4.501225429 | 3.135908790 |
| C | 6.416391660 | 4.034699116 | 1.453720820 |
| H | 6.938810850 | 4.986242467 | 1.480828059 |
| C | 6.754829377 | 3.080248654 | 0.494436905 |
| H | 7.542202800 | 3.284970347 | -0.224559766 |
| C | 6.088370583 | 1.855074518 | 0.456605892 |
| H | 6.364581381 | 1.117667629 | -0.289390538 |

==============================

7-TS

==============================

| | | | |
|---|---|---|---|
| C | -0.848558426 | 0.646185415 | 0.308229705 |
| C | -0.704548251 | -0.192353313 | 2.071859917 |
| Rh | 1.371231667 | 0.178985828 | 0.901713002 |
| P | 2.690622301 | -1.641478662 | 1.440056330 |
| C | 0.105000159 | 0.778359860 | -0.557223166 |
| C | 0.533788291 | -0.113495252 | 2.842797894 |
| C | -1.887326583 | 0.636631704 | 2.582899293 |
| C | -2.288390965 | 1.116549875 | 0.403189749 |
| O | -2.880291747 | 0.645278783 | 1.586700832 |
| H | 0.815718016 | -1.025821359 | 3.359577786 |
| H | 0.692425120 | 0.797049484 | 3.420237327 |
| H | 0.218723954 | 1.184602048 | -1.550392724 |
| H | -2.275087593 | 2.217916723 | 0.375356748 |
| H | -2.867538764 | 0.748863719 | -0.448945173 |
| H | -2.309671942 | 0.148515074 | 3.465319237 |
| H | -1.555347893 | 1.651729280 | 2.855489932 |
| H | -1.007318193 | -1.208428957 | 1.817124462 |
| C | 1.524920347 | -3.042080416 | 1.599317677 |
| C | 0.500993933 | -3.179609445 | 0.642620718 |
| H | 0.427722477 | -2.477880290 | -0.184442785 |
| C | -0.428770105 | -4.212138004 | 0.750595585 |
| H | -1.210516322 | -4.313679464 | 0.003826100 |
| C | -0.356289634 | -5.109317408 | 1.819115877 |
| H | -1.083384472 | -5.911055955 | 1.905565060 |
| C | 0.649332926 | -4.971441353 | 2.776242776 |
| H | 0.706112304 | -5.665001959 | 3.609752643 |
| C | 1.588420994 | -3.943741310 | 2.671605500 |
| H | 2.363377279 | -3.848657339 | 3.424359543 |
| C | 3.827168329 | -2.011582001 | 0.058727977 |
| C | 5.048525355 | -1.314861646 | -0.005850005 |
| H | 5.324101501 | -0.603042736 | 0.766403215 |
| C | 5.913821949 | -1.526708098 | -1.076801785 |
| H | 6.855315707 | -0.987327311 | -1.116236699 |
| C | 5.572758722 | -2.423857672 | -2.093375727 |
| H | 6.249926443 | -2.585930909 | -2.926448802 |
| C | 4.362740622 | -3.117431028 | -2.030717948 |
| H | 4.097871981 | -3.825216381 | -2.810456036 |
| C | 3.489815731 | -2.914697259 | -0.960161804 |
| H | 2.561705639 | -3.474057625 | -0.918173111 |
| C | 3.763086813 | -1.656466353 | 2.917990291 |
| C | 3.614992591 | -0.710575311 | 3.941248857 |
| H | 2.894549810 | 0.090493394 | 3.838758373 |
| C | 4.418826932 | -0.779082704 | 5.080480310 |
| H | 4.294491436 | -0.043595879 | 5.869862538 |
| C | 5.377052474 | -1.785714247 | 5.206186769 |
| H | 6.000816181 | -1.836625376 | 6.093314092 |
| C | 5.538155490 | -2.724283721 | 4.183078809 |
| H | 6.287763132 | -3.504685314 | 4.271422354 |
| C | 4.740518535 | -2.661230463 | 3.042415600 |
| H | 4.879254168 | -3.389307026 | 2.248819133 |
| C | 2.944953275 | 1.020099792 | -0.445721030 |
| C | 3.146641611 | 1.330652048 | 0.755128881 |
| C | 3.187512697 | 0.933720499 | -1.889362286 |
| C | 4.032408030 | 1.844033185 | 1.803325284 |
| H | 2.360033552 | 1.355013925 | -2.468143857 |
| H | 4.103342081 | 1.478494798 | -2.141580664 |
| H | 3.312181301 | -0.114151957 | -2.180234687 |
| O | 5.241413469 | 1.805098458 | 1.708672907 |
| O | 3.356460528 | 2.327744386 | 2.856192476 |
| C | 4.184628052 | 2.833271760 | 3.930343206 |
| H | 3.488623956 | 3.183511903 | 4.691334873 |
| H | 4.821164548 | 2.033782479 | 4.316984968 |
| H | 4.811401306 | 3.651788920 | 3.571983841 |

==============================

8A

==============================

| | | | |
|---|---|---|---|
| C | -0.307094287 | -0.294555182 | 0.423649360 |
| C | 2.266658521 | 0.180227641 | -0.176093509 |
| Rh | 1.395076806 | -1.289338945 | 1.063221222 |
| P | 3.372376282 | -2.673400098 | 2.040885212 |
| P | 0.888251499 | -2.684915407 | -0.818712753 |
| C | -0.079881174 | 0.904413140 | -0.109538374 |
| C | 1.344121197 | 1.383217856 | -0.215752892 |
| C | 1.255285658 | 2.186038143 | -1.534121273 |
| C | -0.945536628 | 1.877411479 | -0.883041552 |
| O | -0.024988862 | 2.810601788 | -1.463141319 |
| H | 3.240208160 | 0.373666325 | 0.266364333 |
| H | 2.357762288 | -0.284433525 | -1.156979739 |
| H | -1.292246779 | -0.756556549 | 0.488649686 |
| H | -1.514814339 | 1.348034713 | -1.662104333 |
| H | -1.648809694 | 2.441362850 | -0.259637965 |
| H | 2.006922825 | 2.972367216 | -1.638142839 |
| H | 1.322458365 | 1.502881761 | -2.397282154 |
| H | 1.570940270 | 2.074861176 | 0.607147437 |

| | | | |
|---|---|---|---|
| C | 1.846146164 | 0.186701371 | 2.729212316 |
| C | 0.635818416 | -0.055147600 | 2.850531856 |
| C | -0.724824767 | -0.140906208 | 3.391103079 |
| C | 3.070289539 | 0.828642112 | 3.209083307 |
| H | -1.145166214 | -1.144107718 | 3.273308807 |
| H | -0.687723386 | 0.109661160 | 4.456743825 |
| H | -1.383064329 | 0.565894680 | 2.880226224 |
| O | 4.145886404 | 0.528994240 | 2.459888589 |
| O | 3.086427224 | 1.544445046 | 4.183874829 |
| C | 5.400714977 | 1.052658861 | 2.950630660 |
| H | 6.156171854 | 0.672962617 | 2.264284784 |
| H | 5.381445006 | 2.144371664 | 2.951150612 |
| H | 5.581768263 | 0.687384333 | 3.963403414 |
| C | 0.150448527 | -1.826500558 | -2.267312122 |
| C | 0.971535632 | -1.381714991 | -3.314918882 |
| H | 2.031808965 | -1.610185736 | -3.318078941 |
| C | 0.432995852 | -0.655751888 | -4.379344127 |
| H | 1.084261039 | -0.321469137 | -5.181210205 |
| C | -0.932161379 | -0.374093564 | -4.421175701 |
| H | -1.350215470 | 0.185403742 | -5.251796767 |
| C | -1.759699353 | -0.827359173 | -3.390678726 |
| H | -2.826279211 | -0.626000931 | -3.419624772 |
| C | -1.224108392 | -1.539067966 | -2.318173276 |
| H | -1.883124449 | -1.881319924 | -1.528385673 |
| C | 2.259704016 | -3.620905327 | -1.592180520 |
| C | 3.520391673 | -3.014896484 | -1.675125737 |
| H | 3.677786538 | -2.030650100 | -1.255054353 |
| C | 4.594284084 | -3.685558301 | -2.260267951 |
| H | 5.570384140 | -3.211839038 | -2.286437458 |
| C | 4.412949116 | -4.969287505 | -2.775554743 |
| H | 5.247888975 | -5.499998412 | -3.222923496 |
| C | 3.154370369 | -5.573865331 | -2.711430984 |
| H | 3.010599068 | -6.573126741 | -3.111570257 |
| C | 2.080398454 | -4.905906558 | -2.125135085 |
| H | 1.117451950 | -5.399481594 | -2.056383662 |
| C | -0.350227809 | -3.922098400 | -0.286670278 |
| C | -0.410703068 | -4.270714518 | 1.071316695 |
| H | 0.271010737 | -3.818275879 | 1.784285249 |
| C | -1.312479603 | -5.235363640 | 1.518414033 |
| H | -1.333843845 | -5.501662639 | 2.570432230 |
| C | -2.168734915 | -5.864252748 | 0.613255650 |
| H | -2.871971987 | -6.615415884 | 0.959083344 |
| C | -2.116251475 | -5.525184453 | -0.741273014 |
| H | -2.778651268 | -6.011857010 | -1.450599665 |
| C | -1.214912235 | -4.560894725 | -1.191870973 |
| H | -1.190014176 | -4.301618363 | -2.245385135 |
| C | 3.749897909 | -2.249504517 | 3.796131253 |
| C | 2.688062579 | -2.034949602 | 4.691402376 |
| C | 2.929921769 | -1.745770958 | 6.032975920 |
| H | 2.095281476 | -1.583189095 | 6.707992494 |
| C | 4.240556452 | -1.654217959 | 6.502202208 |
| H | 4.431570018 | -1.420724111 | 7.544966427 |
| C | 5.303526847 | -1.862141401 | 5.622762463 |
| H | 6.326859010 | -1.796813796 | 5.980561271 |
| C | 5.063200803 | -2.158902035 | 4.280469120 |
| H | 5.904090442 | -2.317308053 | 3.615643740 |
| C | 2.892340386 | -4.456614786 | 2.157259193 |
| C | 2.270962039 | -4.984534048 | 3.300643571 |
| H | 2.141038959 | -4.377801402 | 4.188570932 |
| C | 1.829832728 | -6.308320692 | 3.321921734 |
| H | 1.359419960 | -6.699748116 | 4.219292539 |
| C | 2.005271887 | -7.129167974 | 2.208018333 |
| H | 1.662074681 | -8.159088510 | 2.228299157 |
| C | 2.639624109 | -6.620755963 | 1.074456403 |
| H | 2.798000231 | -7.249782911 | 0.203718321 |
| C | 3.081360736 | -5.299597448 | 1.049098827 |
| H | 3.596726388 | -4.936848803 | 0.170802012 |
| C | 5.039737875 | -2.721500588 | 1.263193688 |
| C | 5.509660530 | -1.589333375 | 0.582204696 |
| H | 4.875217799 | -0.718439589 | 0.488809239 |
| C | 6.788589881 | -1.574248123 | 0.026192669 |
| H | 7.136351833 | -0.691784995 | -0.503002859 |
| C | 7.617603001 | -2.691433665 | 0.148033420 |
| H | 8.613099830 | -2.682161884 | -0.285698345 |
| C | 7.164171433 | -3.818436958 | 0.835379552 |
| H | 7.806177492 | -4.687725365 | 0.941630477 |
| C | 5.885853738 | -3.833990854 | 1.393270750 |
| H | 5.550048172 | -4.715634025 | 1.927984119 |
| H | 1.662810767 | -2.091084295 | 4.341408685 |

==============================

8B

==============================

| | | | |
|---|---|---|---|
| C | -0.164537500 | -0.079246955 | 0.551724655 |
| C | 2.377972114 | 0.073180807 | -0.267540025 |
| Rh | 1.446435759 | -1.222730902 | 1.100617668 |
| P | 3.307149979 | -2.772894488 | 2.139032387 |
| P | 0.810768503 | -2.601833848 | -0.670521062 |
| C | 0.142638052 | 1.076578550 | -0.031778370 |
| C | 1.599063844 | 1.382662274 | -0.259059583 |
| C | 1.502249750 | 2.187200634 | -1.574542672 |
| C | -0.663490761 | 2.141293055 | -0.743859961 |
| O | 0.310033773 | 2.954474483 | -1.415923017 |
| H | 3.419660068 | 0.161617136 | 0.046297823 |
| H | 2.343953899 | -0.381084022 | -1.257769136 |
| H | -1.179258506 | -0.442318576 | 0.717207581 |
| H | -1.356261372 | 1.679095985 | -1.464820967 |
| H | -1.239202969 | 2.790084932 | -0.073802932 |
| H | 2.327727235 | 2.883317531 | -1.747299369 |
| H | 1.425811601 | 1.496761611 | -2.432331500 |
| H | 1.957688603 | 2.052229684 | 0.536527778 |
| C | 1.115348164 | 0.122826512 | 2.998166232 |
| C | 2.255195367 | 0.549862552 | 2.866119745 |
| C | 3.608499505 | 1.085013295 | 2.855961735 |
| C | -0.185616013 | -0.184224053 | 3.579115336 |
| H | 3.890938866 | 1.454033059 | 1.865715428 |
| H | 3.673323116 | 1.920512255 | 3.562141153 |
| H | 4.318316043 | 0.311297828 | 3.168200866 |
| O | -0.537931348 | -1.456531241 | 3.266868707 |
| O | -0.837660009 | 0.568655822 | 4.258103422 |
| C | -1.835395718 | -1.881944062 | 3.745998479 |
| H | -1.971558673 | -2.886074696 | 3.347024616 |
| H | -1.851883552 | -1.884335918 | 4.838446611 |
| H | -2.610134921 | -1.206704937 | 3.375267731 |
| C | 0.008490430 | -1.765231847 | -2.093976835 |
| C | 0.761333424 | -1.420985513 | -3.226975806 |
| H | 1.802194098 | -1.718495100 | -3.305758165 |
| C | 0.178353282 | -0.701405814 | -4.272782738 |
| H | 0.775998657 | -0.443705066 | -5.142384382 |
| C | -1.163423222 | -0.326316957 | -4.207955801 |
| H | -1.615870396 | 0.230436434 | -5.022906136 |
| C | -1.924706673 | -0.681107942 | -3.090295462 |
| H | -2.974061329 | -0.405718589 | -3.035715290 |

| | | | |
|---|---|---|---|
| C | -1.344336788 | -1.388267897 | -2.038729069 |
| H | -1.950609242 | -1.655908859 | -1.179982832 |
| C | 2.162123648 | -3.545612622 | -1.479787439 |
| C | 3.460405577 | -3.017292399 | -1.508209620 |
| H | 3.678313599 | -2.075367011 | -1.024122096 |
| C | 4.500744708 | -3.721300455 | -2.117288551 |
| H | 5.504695093 | -3.307595555 | -2.099619946 |
| C | 4.252067882 | -4.958382711 | -2.714041539 |
| H | 5.060547727 | -5.513539951 | -3.180462993 |
| C | 2.955146471 | -5.481822055 | -2.706805720 |
| H | 2.753648704 | -6.443528739 | -3.170211925 |
| C | 1.914822603 | -4.781846068 | -2.097050383 |
| H | 0.921626475 | -5.217738478 | -2.074188996 |
| C | -0.409621437 | -3.829386517 | -0.069665228 |
| C | -0.405957626 | -4.195880709 | 1.285039974 |
| H | 0.293037061 | -3.740119752 | 1.975823189 |
| C | -1.289633857 | -5.164996909 | 1.759861574 |
| H | -1.254671298 | -5.452108211 | 2.806904382 |
| C | -2.198381976 | -5.772762065 | 0.891510773 |
| H | -2.888218286 | -6.525692401 | 1.261236539 |
| C | -2.216654412 | -5.406952699 | -0.456685448 |
| H | -2.923178099 | -5.871618933 | -1.138334575 |
| C | -1.329793295 | -4.443472399 | -0.937397691 |
| H | -1.362754885 | -4.163179025 | -1.985682790 |
| C | 3.601189608 | -2.329037079 | 3.902753048 |
| C | 2.491245880 | -2.134716008 | 4.742942792 |
| C | 2.663664447 | -1.811438395 | 6.087145072 |
| H | 1.794669720 | -1.668192038 | 6.723356664 |
| C | 3.949953492 | -1.661863120 | 6.610310536 |
| H | 4.085958113 | -1.402413312 | 7.656158877 |
| C | 5.058554878 | -1.849391701 | 5.784766306 |
| H | 6.061832476 | -1.740793919 | 6.186959552 |
| C | 4.889157616 | -2.185025361 | 4.439873838 |
| H | 5.762835052 | -2.336432505 | 3.814721072 |
| C | 2.841313933 | -4.558935875 | 2.240927899 |
| C | 2.291027405 | -5.118588091 | 3.405211654 |
| H | 2.205937220 | -4.532806522 | 4.313038275 |
| C | 1.852983718 | -6.443805112 | 3.417655190 |
| H | 1.434346009 | -6.858548383 | 4.330621852 |
| C | 1.962201930 | -7.235092902 | 2.274341477 |
| H | 1.617901598 | -8.265250442 | 2.286510665 |
| C | 2.531764033 | -6.696261223 | 1.120220018 |
| H | 2.641213147 | -7.302493274 | 0.225590268 |
| C | 2.970842118 | -5.374316310 | 1.103460702 |
| H | 3.435583667 | -4.989344437 | 0.206063050 |
| C | 5.010015884 | -2.790626173 | 1.445307331 |
| C | 5.530799197 | -1.593122057 | 0.932679441 |
| H | 4.918812808 | -0.698596063 | 0.926654922 |
| C | 6.829425305 | -1.537206002 | 0.428354107 |
| H | 7.216722956 | -0.602742460 | 0.032167101 |
| C | 7.627639542 | -2.683454221 | 0.431303838 |
| H | 8.638024267 | -2.645219909 | 0.034374228 |
| C | 7.125140171 | -3.875899973 | 0.955522881 |
| H | 7.744756552 | -4.768035417 | 0.971676118 |
| C | 5.827625447 | -3.930336837 | 1.465570391 |
| H | 5.454901637 | -4.862796543 | 1.875369357 |
| H | 1.486938810 | -2.242768734 | 4.345402699 |

==============================

8C

==============================

| | | | |
|---|---|---|---|
| Rh | 0.620794352 | 1.646925867 | 0.826495015 |
| P | -1.709416799 | 2.247570804 | 0.485196965 |
| P | 2.974654912 | 1.180373340 | 1.168571290 |
| C | 0.328381924 | -0.158578702 | 0.019907924 |
| C | 1.104599490 | 2.126458609 | -1.166975104 |
| C | 0.312427800 | -0.213819532 | -1.310314832 |
| C | 0.487521289 | 1.065166511 | -2.081753884 |
| C | 1.305692587 | 0.530987560 | -3.273943627 |
| C | 0.330805460 | -1.351378776 | -2.303637748 |
| O | 0.722741390 | -0.748469862 | -3.545299267 |
| H | 0.763214124 | 3.143817045 | -1.381049374 |
| H | 2.192158014 | 2.109277413 | -1.261645253 |
| H | 0.268433378 | -1.016599675 | 0.693266629 |
| H | 1.044132548 | -2.134083800 | -2.004694922 |
| H | -0.654117014 | -1.814384487 | -2.448741057 |
| H | 1.225100847 | 1.129362406 | -4.185792046 |
| H | 2.369146480 | 0.436060498 | -2.997747081 |
| H | -0.490251047 | 1.391187343 | -2.458433531 |
| C | 0.898906608 | 4.451121611 | 2.486269991 |
| C | 0.622036543 | 3.384572953 | 3.001134170 |
| C | 1.209549208 | 5.736105278 | 1.887232200 |
| C | 0.319171449 | 2.280176830 | 3.888912470 |
| H | 0.946773219 | 5.744626758 | 0.825276635 |
| H | 0.637038970 | 6.526137632 | 2.382457304 |
| H | 2.278138618 | 5.950376586 | 1.986142769 |
| O | 0.122823497 | 2.365717426 | 5.074998239 |
| O | 0.313570897 | 1.103175118 | 3.198986129 |
| C | 0.232875904 | -0.096611451 | 4.012413095 |
| H | 0.322753481 | -0.925417291 | 3.312109740 |
| H | 1.059678436 | -0.105319007 | 4.723281607 |
| H | -0.724203855 | -0.122797502 | 4.534461193 |
| C | -1.813778668 | 4.079202044 | 0.491765861 |
| C | -2.206246284 | 4.801705384 | 1.629902913 |
| H | -2.486562055 | 4.281989687 | 2.539177617 |
| C | -2.240555923 | 6.196392551 | 1.607668820 |
| H | -2.562713928 | 6.735659499 | 2.493991312 |
| C | -1.867505763 | 6.893590189 | 0.457975954 |
| H | -1.906850986 | 7.978666859 | 0.439834744 |
| C | -1.436436291 | 6.187384979 | -0.667170026 |
| H | -1.134523182 | 6.719450280 | -1.564636841 |
| C | -1.397509202 | 4.792987302 | -0.647497187 |
| H | -1.066028759 | 4.263091883 | -1.534352949 |
| C | -2.657298944 | 1.686167539 | -0.995104878 |
| C | -3.295990162 | 2.550806887 | -1.895399891 |
| H | -3.249732203 | 3.624720983 | -1.763697373 |
| C | -4.027426708 | 2.038131592 | -2.968634576 |
| H | -4.514141847 | 2.722861397 | -3.657080734 |
| C | -4.140746168 | 0.661357423 | -3.151120005 |
| H | -4.705429473 | 0.266256695 | -3.990240900 |
| C | -3.540227595 | -0.207170324 | -2.236919555 |
| H | -3.643546723 | -1.281725214 | -2.356442558 |
| C | -2.812031489 | 0.298403143 | -1.163801260 |
| H | -2.363806691 | -0.388721956 | -0.457051673 |
| C | -2.835570843 | 1.611061715 | 1.789834885 |
| C | -4.058418649 | 2.228338489 | 2.098523413 |
| H | -4.334863999 | 3.166624510 | 1.628944129 |
| C | -4.938420055 | 1.629359994 | 2.998066934 |
| H | -5.877078748 | 2.120513630 | 3.237154023 |
| C | -4.625668172 | 0.397376145 | 3.576580459 |
| H | -5.316668286 | -0.067705988 | 4.273247593 |
| C | -3.432964007 | -0.243089823 | 3.240182951 |
| H | -3.197196273 | -1.215665369 | 3.663300225 |

| | | | |
|---|---|---|---|
| C | -2.543608178 | 0.361011832 | 2.351906946 |
| H | -1.630568880 | -0.149557991 | 2.071724336 |
| C | 4.036380133 | 2.679925339 | 1.101968681 |
| C | 5.329242842 | 2.645422794 | 1.652510008 |
| H | 5.681960526 | 1.750318164 | 2.155293476 |
| C | 6.164351914 | 3.756481874 | 1.561276889 |
| H | 7.159805076 | 3.717491465 | 1.993757181 |
| C | 5.724670628 | 4.913300482 | 0.913278314 |
| H | 6.378803445 | 5.777052555 | 0.839533705 |
| C | 4.444294964 | 4.955147232 | 0.360871940 |
| H | 4.098165811 | 5.851479318 | -0.146170200 |
| C | 3.602184422 | 3.845671214 | 0.457405988 |
| H | 2.606499601 | 3.894262205 | 0.033514475 |
| C | 3.390677937 | 0.494787795 | 2.825540431 |
| C | 3.340199775 | 1.374068334 | 3.922456955 |
| H | 3.130848436 | 2.427981068 | 3.765690521 |
| C | 3.574735758 | 0.912594950 | 5.215379469 |
| H | 3.524593617 | 1.605995845 | 6.049493104 |
| C | 3.868311077 | -0.436497360 | 5.436210113 |
| H | 4.050948914 | -0.798148273 | 6.443705384 |
| C | 3.942065228 | -1.311003921 | 4.352621509 |
| H | 4.192927717 | -2.356045438 | 4.510600367 |
| C | 3.705967907 | -0.851446556 | 3.054860331 |
| H | 3.800320688 | -1.545752195 | 2.229101654 |
| C | 3.727417030 | 0.052197575 | -0.065997711 |
| C | 4.696829462 | 0.512147048 | -0.969767693 |
| H | 5.058123773 | 1.533057040 | -0.913620966 |
| C | 5.210685414 | -0.340156427 | -1.949750396 |
| H | 5.958742947 | 0.032348686 | -2.643312596 |
| C | 4.771718686 | -1.660535307 | -2.036503598 |
| H | 5.174480773 | -2.321171187 | -2.798276161 |
| C | 3.807712349 | -2.128128038 | -1.140885643 |
| H | 3.455816468 | -3.153674154 | -1.203402429 |
| C | 3.277010551 | -1.275665387 | -0.175921080 |
| H | 2.501744341 | -1.648072536 | 0.482051445 |

==============================

8C-BPR

==============================

| | | | |
|---|---|---|---|
| C | 1.074541205 | 0.482752594 | 0.231309233 |
| C | 2.752070957 | -1.311007715 | -0.971195453 |
| Rh | 1.653741129 | -1.354926600 | 0.841250194 |
| P | 3.782292864 | -2.107693838 | 2.067269215 |
| P | -0.206385253 | -2.067027418 | -0.289044139 |
| C | 1.836145397 | 0.991627026 | -0.733084145 |
| C | 2.947916161 | 0.166354985 | -1.316170075 |
| C | 2.886829321 | 0.643411296 | -2.780922326 |
| C | 1.701174733 | 2.240837898 | -1.583168059 |
| O | 2.620283191 | 2.044399521 | -2.664147793 |
| H | 3.697252539 | -1.833847826 | -0.843843355 |
| H | 2.184569305 | -1.827442731 | -1.744761286 |
| H | 0.206935283 | 0.970414684 | 0.665698277 |
| H | 0.668655345 | 2.336093135 | -1.954919212 |
| H | 1.972161753 | 3.170463591 | -1.069907213 |
| H | 3.821956590 | 0.527818258 | -3.334918704 |
| H | 2.079176074 | 0.124093818 | -3.319813095 |
| H | 3.904199332 | 0.517938868 | -0.901603460 |
| C | 0.630552551 | -0.822934010 | 3.180176116 |
| C | 0.224937962 | -1.967366716 | 3.282993330 |
| C | -0.263761823 | -3.321590598 | 3.481913144 |
| C | 0.884259091 | 0.595884108 | 3.377060385 |
| H | -0.876193187 | -3.648210675 | 2.635661456 |
| H | 0.566356783 | -4.019740707 | 3.630393042 |
| H | -0.892057739 | -3.342483237 | 4.379995893 |
| O | 2.089436481 | 0.787075241 | 3.930185446 |
| O | 0.098669372 | 1.471785199 | 3.078611637 |
| C | 2.466854767 | 2.164680082 | 4.159500866 |
| H | 3.486539128 | 2.119350552 | 4.537150230 |
| H | 2.427364843 | 2.723343255 | 3.221640967 |
| H | 1.792141227 | 2.623274894 | 4.886670718 |
| C | -0.530714899 | -1.403601815 | -1.966290763 |
| C | 0.076592272 | -1.940420236 | -3.113342346 |
| C | -1.326294359 | -0.252580135 | -2.094745021 |
| C | -0.113936907 | -1.341531742 | -4.359942993 |
| C | -1.504238920 | 0.347411613 | -3.340529939 |
| C | -0.896951979 | -0.192412063 | -4.476914875 |
| H | 0.693550572 | -2.830510106 | -3.045628424 |
| H | -1.813277694 | 0.175027934 | -1.224374876 |
| H | 0.357502991 | -1.773126143 | -5.237980477 |
| H | -2.122192229 | 1.236621338 | -3.422764558 |
| H | -1.036616660 | 0.276873872 | -5.445855766 |
| C | -1.811512757 | -1.860837451 | 0.584140676 |
| C | -2.829038408 | -2.814667930 | 0.413078802 |
| C | -2.063504689 | -0.724119993 | 1.368147240 |
| C | -4.073729382 | -2.628942542 | 1.011845034 |
| C | -3.312228877 | -0.543357131 | 1.962087311 |
| C | -4.318831504 | -1.492722189 | 1.784548510 |
| H | -2.655477170 | -3.704856159 | -0.181341834 |
| H | -1.296674376 | 0.016262589 | 1.546472029 |
| H | -4.852040164 | -3.373339618 | 0.872204026 |
| H | -3.488552224 | 0.340215621 | 2.567862958 |
| H | -5.290163861 | -1.350616452 | 2.248959850 |
| C | 0.017174985 | -3.883566320 | -0.470353471 |
| C | 0.801398085 | -4.561339110 | 0.479065127 |
| C | -0.594078625 | -4.629634253 | -1.492466693 |
| C | 0.986217825 | -5.941346340 | 0.402704004 |
| C | -0.408889507 | -6.011269352 | -1.565826571 |
| C | 0.384061864 | -6.671211957 | -0.623671073 |
| H | 1.278171540 | -4.013275950 | 1.286121841 |
| H | -1.212075048 | -4.135608515 | -2.235452059 |
| H | 1.601894630 | -6.440206161 | 1.145824378 |
| H | -0.887007079 | -6.572120437 | -2.363635792 |
| H | 0.526854022 | -7.745636372 | -0.687882240 |
| C | 3.422142062 | -3.143886706 | 3.541187097 |
| C | 3.005253045 | -2.539113337 | 4.739768183 |
| C | 3.449669714 | -4.546116873 | 3.458984317 |
| C | 2.631710357 | -3.323895433 | 5.830435264 |
| C | 3.067227496 | -5.325802617 | 4.552981672 |
| C | 2.654970271 | -4.718640323 | 5.741028180 |
| H | 2.969889019 | -1.457192006 | 4.818402398 |
| H | 3.787350543 | -5.034156970 | 2.549785972 |
| H | 2.319917793 | -2.843040319 | 6.753355266 |
| H | 3.103887840 | -6.409038729 | 4.477934984 |
| H | 2.362828370 | -5.325938549 | 6.592504788 |
| C | 4.935109473 | -3.143621290 | 1.084317615 |
| C | 6.324041273 | -3.110645159 | 1.274738401 |
| C | 4.395761214 | -4.039140157 | 0.145312686 |
| C | 7.152594365 | -3.964831099 | 0.546558983 |
| C | 5.226114280 | -4.899508797 | -0.571636361 |
| C | 6.607191615 | -4.864247956 | -0.371306834 |
| H | 6.763908482 | -2.419084549 | 1.985668223 |
| H | 3.325256019 | -4.065343962 | -0.030462762 |
| H | 8.227294474 | -3.928456107 | 0.699514886 |

| | | | |
|---|---|---|---|
| H | 4.793987995 | -5.589927632 | -1.290588184 |
| H | 7.256845472 | -5.528558796 | -0.933838685 |
| C | 4.777692464 | -0.703143435 | 2.678102934 |
| C | 5.656346289 | -0.823578472 | 3.766346503 |
| C | 4.685991468 | 0.520616627 | 1.999237320 |
| C | 6.441554268 | 0.261720478 | 4.155556006 |
| C | 5.483297542 | 1.598474883 | 2.381770924 |
| C | 6.362891398 | 1.470232624 | 3.459051303 |
| H | 5.722854727 | -1.759492184 | 4.312824253 |
| H | 3.978373230 | 0.638747017 | 1.185208570 |
| H | 7.118660586 | 0.161436061 | 4.999040470 |
| H | 5.409715034 | 2.540200570 | 1.845474956 |
| H | 6.981778894 | 2.310732685 | 3.759843007 |

==============================

8A-TS1

==============================

| | | | |
|---|---|---|---|
| C | -0.037408423 | 0.043581524 | -0.189636593 |
| C | -0.401405142 | 0.314589986 | 1.989307477 |
| C | 2.608104503 | -0.170979936 | 0.546676629 |
| C | 0.613070864 | -0.016179601 | 2.668546959 |
| Rh | 0.945723220 | -1.358791965 | 1.045344166 |
| P | 1.857811392 | -3.095734182 | 2.632505410 |
| P | 1.080875372 | -2.732825941 | -0.918300401 |
| C | 0.768445634 | 1.037775633 | -0.576178830 |
| C | 2.121339141 | 1.182243806 | 0.066129797 |
| C | 2.899963908 | 1.856866502 | -1.089391622 |
| C | 0.685845740 | 2.025694510 | -1.719060087 |
| O | 1.940948749 | 2.713855204 | -1.702014840 |
| H | 3.179754292 | -0.110593245 | 1.468875810 |
| H | 3.145710949 | -0.724026317 | -0.223745584 |
| H | -0.999664966 | -0.157017577 | -0.657469218 |
| H | 0.529015558 | 1.502614486 | -2.673303300 |
| H | -0.112972236 | 2.766795012 | -1.593752624 |
| H | 3.743183150 | 2.470417918 | -0.763115414 |
| H | 3.264281031 | 1.091636802 | -1.795771386 |
| H | 2.050556915 | 1.885396177 | 0.907534479 |
| C | -1.758361652 | 0.875052929 | 1.869779791 |
| H | -2.458466998 | 0.146098708 | 1.448475431 |
| H | -2.091043762 | 1.141419239 | 2.880070817 |
| H | -1.771449073 | 1.765094164 | 1.235476323 |
| C | 1.237755733 | 0.233108804 | 3.975774220 |
| O | 0.661017886 | 0.727492243 | 4.918560116 |
| O | 2.533039409 | -0.140661647 | 3.978919973 |
| C | 3.224132961 | 0.034821000 | 5.234538987 |
| H | 3.282853617 | 1.095439682 | 5.490273000 |
| H | 2.697790204 | -0.502208611 | 6.026455625 |
| H | 4.217009543 | -0.385213578 | 5.075600935 |
| C | 0.746639255 | -1.859378756 | -2.504831616 |
| C | 1.812265153 | -1.222464688 | -3.162458751 |
| C | -0.546242282 | -1.740176273 | -3.032238381 |
| C | 1.589887547 | -0.491421515 | -4.328285356 |
| C | -0.765602953 | -1.002495779 | -4.198339707 |
| C | 0.298445251 | -0.376297348 | -4.849976336 |
| H | 2.823646278 | -1.314472295 | -2.777775943 |
| H | -1.383634694 | -2.233044703 | -2.548151967 |
| H | 2.425683902 | -0.010621062 | -4.828153971 |
| H | -1.771407767 | -0.926764966 | -4.601711730 |
| H | 0.124854605 | 0.191755703 | -5.758826968 |
| C | 2.652651187 | -3.581408108 | -1.315781474 |
| C | 2.784509868 | -4.345494010 | -2.489016144 |
| C | 3.771476678 | -3.407484793 | -0.492537492 |
| C | 3.994649221 | -4.965778929 | -2.791515510 |
| C | 4.987684202 | -4.017811661 | -0.802331481 |
| C | 5.097538597 | -4.806914964 | -1.947008950 |
| H | 1.947409863 | -4.441552467 | -3.173860980 |
| H | 3.697386547 | -2.806800006 | 0.403227380 |
| H | 4.081646223 | -5.564134475 | -3.693695597 |
| H | 5.840339499 | -3.875555928 | -0.144359632 |
| H | 6.040821953 | -5.287227006 | -2.190411775 |
| C | -0.262066311 | -3.972699816 | -0.782133710 |
| C | -1.468055566 | -3.526573065 | -0.210302638 |
| C | -0.169958675 | -5.298238459 | -1.222499301 |
| C | -2.565547823 | -4.379291578 | -0.107528514 |
| C | -1.270116717 | -6.149262508 | -1.116471473 |
| C | -2.469052982 | -5.693375248 | -0.568123398 |
| H | -1.554356230 | -2.501327214 | 0.145396937 |
| H | 0.760164926 | -5.680683086 | -1.624941982 |
| H | -3.492192966 | -4.019185760 | 0.330422486 |
| H | -1.184530741 | -7.177098917 | -1.456444200 |
| H | -3.321812068 | -6.361385587 | -0.490425653 |
| C | 1.399346949 | -4.833095451 | 2.164026220 |
| C | 0.156253062 | -5.374326475 | 2.526351607 |
| C | 2.295590693 | -5.637656755 | 1.439960247 |
| C | -0.172816869 | -6.686400968 | 2.188424788 |
| C | 1.964514698 | -6.950306665 | 1.108188518 |
| C | 0.731206319 | -7.481481621 | 1.484919126 |
| H | -0.555426826 | -4.790492598 | 3.097657185 |
| H | 3.268276822 | -5.258330972 | 1.154676236 |
| H | -1.137261730 | -7.087537117 | 2.485744294 |
| H | 2.679911172 | -7.558428916 | 0.561856072 |
| H | 0.476530652 | -8.507173948 | 1.233662369 |
| C | 3.634369092 | -3.269982904 | 3.082073077 |
| C | 4.112014492 | -4.442621729 | 3.695342672 |
| C | 4.535180310 | -2.224231847 | 2.838812904 |
| C | 5.456152135 | -4.562299226 | 4.045122321 |
| C | 5.882274411 | -2.348208106 | 3.182663831 |
| C | 6.346132904 | -3.517863529 | 3.785659663 |
| H | 3.435193424 | -5.266446108 | 3.896395819 |
| H | 4.185405686 | -1.307581697 | 2.387595277 |
| H | 5.808060908 | -5.474386674 | 4.518554044 |
| H | 6.566541037 | -1.529576113 | 2.978679699 |
| H | 7.394274183 | -3.616360915 | 4.053006282 |
| C | 0.991951229 | -2.901497567 | 4.251233540 |
| C | -0.376735542 | -2.582233187 | 4.245962581 |
| C | 1.638372468 | -3.075229814 | 5.483006280 |
| C | -1.082966320 | -2.451803489 | 5.440115748 |
| C | 0.930496866 | -2.935019016 | 6.677607851 |
| C | -0.429067918 | -2.624665835 | 6.660335646 |
| H | -0.896877513 | -2.424246050 | 3.305049158 |
| H | 2.695404272 | -3.313374373 | 5.519136578 |
| H | -2.139827860 | -2.203201975 | 5.417261668 |
| H | 1.446859002 | -3.069396945 | 7.623891136 |
| H | -0.975020671 | -2.512399895 | 7.592094086 |

==============================

8A-TS2

==============================

| | | | |
|---|---|---|---|
| C | -0.636012362 | -0.092945386 | 0.737502056 |
| C | 0.562318952 | -0.588941853 | 2.573637586 |
| Rh | 1.628626251 | -0.022949525 | 0.776870348 |
| P | 1.637346998 | 2.478907402 | 1.418598970 |
| P | 3.822978160 | 0.034408862 | -0.157016380 |
| C | 1.315075146 | -1.839276314 | -0.120130012 |

| | | | |
|---|---|---|---|
| C | 0.049655590 | -2.257384200 | -0.213185378 |
| C | -1.081836512 | -1.484414225 | 0.411186712 |
| C | -2.137310329 | -1.646164446 | -0.713545951 |
| C | -0.576335765 | -3.316289045 | -1.105253719 |
| O | -1.973510987 | -2.997418613 | -1.131531690 |
| H | -1.235478484 | 0.492126198 | 1.425227494 |
| H | -0.413921171 | 0.491622125 | -0.163648796 |
| H | 2.139651334 | -2.390766331 | -0.565654844 |
| H | -0.145682968 | -3.292266911 | -2.117174370 |
| H | -0.466401884 | -4.333569326 | -0.709478375 |
| H | -3.168099044 | -1.516439418 | -0.375331676 |
| H | -1.935720887 | -0.936244698 | -1.534230057 |
| H | -1.464588258 | -2.003636727 | 1.298851597 |
| C | 1.635155814 | -1.276958101 | 2.390201136 |
| C | 2.567098696 | -2.303571718 | 2.887211271 |
| H | 3.569089130 | -1.877819737 | 3.009978293 |
| H | 2.216597028 | -2.688288773 | 3.850657524 |
| H | 2.639659328 | -3.127835847 | 2.170834247 |
| C | -0.284852244 | -0.077437721 | 3.657445928 |
| O | -1.133574092 | 0.787361595 | 3.563185489 |
| O | 0.063445339 | -0.674825767 | 4.813716322 |
| C | -0.630136967 | -0.185644973 | 5.985205706 |
| H | -0.392215037 | 0.869906480 | 6.141090310 |
| H | -1.709809170 | -0.300980405 | 5.867075901 |
| H | -0.262959910 | -0.793159418 | 6.811699563 |
| C | 4.427890574 | -1.501521766 | -0.960976981 |
| C | 4.904057068 | -2.535834714 | -0.137217399 |
| C | 4.351520981 | -1.709317170 | -2.344785615 |
| C | 5.299560420 | -3.751619099 | -0.690044687 |
| C | 4.749530418 | -2.929886493 | -2.894025142 |
| C | 5.220531475 | -3.952292028 | -2.070814775 |
| H | 4.980486639 | -2.385189692 | 0.935921331 |
| H | 3.991505571 | -0.923136508 | -2.999778950 |
| H | 5.673435563 | -4.541289658 | -0.044803767 |
| H | 4.693139976 | -3.077162279 | -3.968664741 |
| H | 5.529144853 | -4.900226760 | -2.501529185 |
| C | 3.776314016 | 1.282575284 | -1.496809140 |
| C | 2.655805059 | 1.240659021 | -2.347926008 |
| C | 4.755341756 | 2.261283912 | -1.702321100 |
| C | 2.531065312 | 2.149210257 | -3.397849176 |
| C | 4.621415401 | 3.172596719 | -2.750739768 |
| C | 3.516821690 | 3.117553175 | -3.600642860 |
| H | 1.886756131 | 0.485031510 | -2.196928258 |
| H | 5.605254172 | 2.339667457 | -1.034866373 |
| H | 1.667485723 | 2.099911643 | -4.055243706 |
| H | 5.379829492 | 3.936060278 | -2.893437149 |
| H | 3.419730971 | 3.829928784 | -4.414641110 |
| C | 5.197737724 | 0.425931529 | 0.984475133 |
| C | 6.525305602 | 0.459165760 | 0.525386626 |
| C | 4.940612541 | 0.629042435 | 2.345535319 |
| C | 7.565021910 | 0.738604521 | 1.410821012 |
| C | 5.983785909 | 0.899287141 | 3.232537140 |
| C | 7.297246635 | 0.963037758 | 2.765047738 |
| H | 6.746199844 | 0.256571421 | -0.518690429 |
| H | 3.920528585 | 0.594547245 | 2.706805917 |
| H | 8.587061926 | 0.772838677 | 1.045041608 |
| H | 5.765715691 | 1.065327240 | 4.283559313 |
| H | 8.111170291 | 1.176488421 | 3.451286239 |
| C | 2.831542862 | 3.739729121 | 0.771278955 |
| C | 2.511666826 | 4.677488656 | -0.219308754 |
| C | 4.110194772 | 3.782628379 | 1.352596190 |
| C | 3.452444963 | 5.625573104 | -0.625525048 |
| C | 5.047841579 | 4.726343358 | 0.941502081 |
| C | 4.722408373 | 5.652447858 | -0.052100974 |
| H | 1.527803985 | 4.694119185 | -0.670824502 |
| H | 4.373602682 | 3.092023097 | 2.144842995 |
| H | 3.182774138 | 6.348431408 | -1.390414028 |
| H | 6.029512896 | 4.743133369 | 1.406604394 |
| H | 5.449688869 | 6.394750692 | -0.368535112 |
| C | 1.704753427 | 2.887020922 | 3.211402990 |
| C | 1.473226650 | 4.208327305 | 3.631337405 |
| C | 2.055991922 | 1.928785934 | 4.169358936 |
| C | 1.565324330 | 4.550339602 | 4.978398822 |
| C | 2.157399451 | 2.273413558 | 5.519552020 |
| C | 1.907539249 | 3.583313281 | 5.928848240 |
| H | 1.225474659 | 4.971158286 | 2.899629777 |
| H | 2.238111476 | 0.904746605 | 3.872640624 |
| H | 1.377679570 | 5.574894798 | 5.286218071 |
| H | 2.432304322 | 1.514248303 | 6.246479545 |
| H | 1.985591277 | 3.853941929 | 6.977677481 |
| C | 0.000027461 | 3.059859178 | 0.820963077 |
| C | -0.238520740 | 3.015820071 | -0.565820510 |
| C | -1.047508836 | 3.407711202 | 1.681488882 |
| C | -1.488318459 | 3.352780502 | -1.080058384 |
| C | -2.301713239 | 3.736168292 | 1.160904628 |
| C | -2.524334423 | 3.716701935 | -0.215260641 |
| H | 0.553456517 | 2.710503553 | -1.246126288 |
| H | -0.904107944 | 3.394718480 | 2.755192334 |
| H | -1.655890407 | 3.325929099 | -2.153259681 |
| H | -3.107787289 | 4.000475622 | 1.839024115 |
| H | -3.501197099 | 3.974629525 | -0.613874169 |

==============================

8B-TS1

==============================

| | | | |
|---|---|---|---|
| C | -0.120141609 | -0.069398005 | 0.030523525 |
| C | 0.098744317 | -0.034055055 | 2.300625790 |
| C | 2.640762051 | 0.168201168 | 0.178359589 |
| C | 1.294293373 | 0.060936000 | 2.721961877 |
| Rh | 1.350033149 | -1.258348401 | 1.014627647 |
| P | 3.131876985 | -2.608698810 | 1.971046595 |
| P | 0.823315233 | -2.690929523 | -0.952490607 |
| C | 0.420554393 | 1.056461228 | -0.434830515 |
| C | 1.839994203 | 1.425307618 | -0.078137497 |
| C | 2.223650300 | 2.278288870 | -1.313554429 |
| C | -0.060940527 | 2.040267030 | -1.482247288 |
| O | 1.020612953 | 2.959394386 | -1.651344880 |
| H | 3.466983631 | 0.294700273 | 0.878943845 |
| H | 2.980064588 | -0.302055469 | -0.742833857 |
| H | -1.125215980 | -0.412582271 | -0.186832291 |
| H | -0.291308008 | 1.515326250 | -2.421966353 |
| H | -0.947156066 | 2.605079591 | -1.172624854 |
| H | 2.993372645 | 3.027767764 | -1.114893490 |
| H | 2.557371007 | 1.622733118 | -2.134766486 |
| H | 1.832405544 | 2.069244719 | 0.812174414 |
| C | 2.194078505 | 0.686774426 | 3.710407287 |
| H | 3.128774328 | 1.021856865 | 3.253680240 |
| H | 1.676588574 | 1.542163345 | 4.156904921 |
| H | 2.461152168 | -0.016586688 | 4.503279283 |
| C | -1.285247169 | 0.302466528 | 2.656605904 |
| O | -1.532672806 | 1.148588948 | 3.488194221 |
| O | -2.206039036 | -0.451508089 | 2.031901445 |
| C | -3.577980588 | -0.184840347 | 2.398062885 |

| | | | |
|---|---|---|---|
| H | -3.720665890 | -0.327910190 | 3.471925530 |
| H | -3.847932789 | 0.841091874 | 2.136550711 |
| H | -4.170414887 | -0.898390402 | 1.826279624 |
| C | 0.711317189 | -1.704805735 | -2.495773940 |
| C | 1.887598273 | -1.181474130 | -3.059573778 |
| C | -0.517671085 | -1.399951410 | -3.095675379 |
| C | 1.837563793 | -0.388454503 | -4.204838065 |
| C | -0.565616117 | -0.603470014 | -4.242218833 |
| C | 0.608409046 | -0.097603106 | -4.800432321 |
| H | 2.854873052 | -1.403557313 | -2.619095141 |
| H | -1.440355521 | -1.785823503 | -2.675769855 |
| H | 2.757337244 | 0.001052739 | -4.631472076 |
| H | -1.525679211 | -0.380266780 | -4.698659822 |
| H | 0.567190782 | 0.522337232 | -5.690895461 |
| C | 1.748790805 | -4.204662107 | -1.450651694 |
| C | 1.808380379 | -5.241849179 | -0.507443882 |
| C | 2.360946869 | -4.381196579 | -2.698055944 |
| C | 2.503072755 | -6.414517205 | -0.788240616 |
| C | 3.053809438 | -5.560497338 | -2.977581836 |
| C | 3.137007413 | -6.573403468 | -2.022786472 |
| H | 1.296561963 | -5.141199517 | 0.442682362 |
| H | 2.298993878 | -3.612127300 | -3.459038059 |
| H | 2.542548622 | -7.204255986 | -0.043429971 |
| H | 3.524911147 | -5.686393938 | -3.948363535 |
| H | 3.681097989 | -7.487158753 | -2.243663809 |
| C | -0.843720778 | -3.412079321 | -0.682838984 |
| C | -1.389817627 | -4.289631584 | -1.635936384 |
| C | -1.556011376 | -3.152583754 | 0.493881486 |
| C | -2.633600617 | -4.875956819 | -1.418952125 |
| C | -2.798592990 | -3.749509286 | 0.713517261 |
| C | -3.339782699 | -4.608611715 | -0.241867768 |
| H | -0.837167679 | -4.517973761 | -2.542599158 |
| H | -1.157980100 | -2.477366654 | 1.241368466 |
| H | -3.049395527 | -5.548611100 | -2.163435310 |
| H | -3.337578064 | -3.543140413 | 1.633981734 |
| H | -4.306576375 | -5.073903235 | -0.072013044 |
| C | 4.499332034 | -3.229193209 | 0.927479800 |
| C | 5.560675421 | -3.953871777 | 1.499740112 |
| C | 4.538061459 | -2.935209131 | -0.438743412 |
| C | 6.609482236 | -4.407364569 | 0.704483809 |
| C | 5.591112231 | -3.384946432 | -1.234305142 |
| C | 6.624300533 | -4.127323424 | -0.664949932 |
| H | 5.575022233 | -4.146666538 | 2.568499993 |
| H | 3.737701974 | -2.359155350 | -0.881037093 |
| H | 7.421476098 | -4.970767874 | 1.154715318 |
| H | 5.599582300 | -3.157840672 | -2.296061332 |
| H | 7.446176539 | -4.478767557 | -1.281736963 |
| C | 2.250815579 | -4.054840407 | 2.682893240 |
| C | 0.992193251 | -3.817913878 | 3.263898588 |
| C | 2.749375540 | -5.365395020 | 2.649520571 |
| C | 0.248228287 | -4.870374053 | 3.797607754 |
| C | 2.001918621 | -6.414788883 | 3.183493035 |
| C | 0.751871959 | -6.171263476 | 3.757278701 |
| H | 0.595280453 | -2.806679582 | 3.313018035 |
| H | 3.703729659 | -5.576467381 | 2.180920447 |
| H | -0.722873773 | -4.673333931 | 4.242220553 |
| H | 2.396513699 | -7.426336698 | 3.148659441 |
| H | 0.171890198 | -6.992803384 | 4.167335448 |
| C | 4.074586091 | -1.932157730 | 3.392805683 |
| C | 3.795188948 | -2.297613979 | 4.716941312 |
| C | 5.084196102 | -0.989145143 | 3.138919315 |
| C | 4.502535783 | -1.713898991 | 5.770171295 |
| C | 5.786459326 | -0.409153854 | 4.193172879 |
| C | 5.492024711 | -0.764699667 | 5.512166034 |
| H | 3.030086412 | -3.036107990 | 4.932421093 |
| H | 5.337136674 | -0.722938469 | 2.115815090 |
| H | 4.280822208 | -2.007114827 | 6.792314767 |
| H | 6.566538376 | 0.317661717 | 3.986277805 |
| H | 6.039157613 | -0.311639495 | 6.333439508 |

==============================

8B-TS2

==============================

| | | | |
|---|---|---|---|
| C | 0.115223994 | 0.012558891 | 0.125330527 |
| C | 0.215731561 | 0.324677727 | 2.388609858 |
| Rh | 2.112592696 | 0.022635879 | 1.200122834 |
| P | 2.417849770 | 2.625724487 | 1.011091076 |
| P | 4.443681846 | -0.403786787 | 1.412176486 |
| C | 1.824173664 | -1.978627507 | 0.981511014 |
| C | 0.653442956 | -2.367763239 | 0.468102893 |
| C | -0.441263067 | -1.379151765 | 0.160937633 |
| C | -0.953937682 | -1.986949430 | -1.170067569 |
| C | 0.235348204 | -3.693557581 | -0.143597691 |
| O | -0.928108446 | -3.388088231 | -0.926763311 |
| H | -0.584237120 | 0.842420394 | 0.143385642 |
| H | 0.806903663 | 0.155942322 | -0.713031432 |
| H | 2.614490291 | -2.687775699 | 1.214433662 |
| H | 1.034009194 | -4.114940811 | -0.771279956 |
| H | -0.046021448 | -4.444836624 | 0.604361654 |
| H | -1.978588780 | -1.703772036 | -1.423731861 |
| H | -0.285714820 | -1.701482315 | -2.001175175 |
| H | -1.253364452 | -1.472246220 | 0.894834775 |
| C | 1.189284559 | -0.281118183 | 2.959946909 |
| C | -1.050469555 | 1.058213098 | 2.570497077 |
| H | -0.967261970 | 2.075217694 | 2.172650189 |
| H | -1.892408128 | 0.563141140 | 2.076279627 |
| H | -1.256480064 | 1.114813633 | 3.644287167 |
| C | 1.627740605 | -0.810756079 | 4.254193842 |
| O | 2.658549380 | -1.420825414 | 4.448641753 |
| O | 0.745587821 | -0.476130940 | 5.215288056 |
| C | 1.095939774 | -0.912721155 | 6.549747794 |
| H | 1.156133398 | -2.002714547 | 6.587902802 |
| H | 2.059820874 | -0.490556868 | 6.844594736 |
| H | 0.297373923 | -0.546831556 | 7.193330325 |
| C | 5.311013967 | 0.394787408 | 2.801952700 |
| C | 4.587466165 | 1.115011979 | 3.758996194 |
| C | 6.699811800 | 0.249491442 | 2.954960385 |
| C | 5.241279029 | 1.718307029 | 4.832994042 |
| C | 7.353252661 | 0.862156265 | 4.022511070 |
| C | 6.625929878 | 1.600716663 | 4.960295246 |
| H | 3.514863299 | 1.212488460 | 3.656936643 |
| H | 7.266567801 | -0.347699119 | 2.246327720 |
| H | 4.668464325 | 2.282668725 | 5.563307959 |
| H | 8.428966934 | 0.755882225 | 4.128241117 |
| H | 7.138404625 | 2.072899157 | 5.793503400 |
| C | 5.078508363 | -2.125434370 | 1.482501992 |
| C | 5.681592332 | -2.750388780 | 0.380951271 |
| C | 4.953682550 | -2.829380300 | 2.693180412 |
| C | 6.157437029 | -4.058574960 | 0.491811409 |
| C | 5.432685568 | -4.134262318 | 2.793602343 |
| C | 6.035425555 | -4.753701881 | 1.695378913 |
| H | 5.796195860 | -2.222355087 | -0.559403515 |
| H | 4.478893434 | -2.360391024 | 3.547012719 |

| | | | |
|---|---|---|---|
| H | 6.630119433 | -4.529756012 | -0.365218879 |
| H | 5.335309052 | -4.667541146 | 3.734932771 |
| H | 6.409924907 | -5.769628537 | 1.778979980 |
| C | 5.103773776 | 0.263479278 | -0.166143673 |
| C | 4.406345186 | -0.093773431 | -1.336608496 |
| C | 6.213551176 | 1.110621977 | -0.267961587 |
| C | 4.817558914 | 0.383039712 | -2.581307257 |
| C | 6.619161369 | 1.587127545 | -1.516081437 |
| C | 5.926601837 | 1.228283722 | -2.673270544 |
| H | 3.554531525 | -0.769593077 | -1.276815884 |
| H | 6.748424942 | 1.426299667 | 0.619875148 |
| H | 4.277088230 | 0.089056858 | -3.476929950 |
| H | 7.473008764 | 2.254526574 | -1.577884111 |
| H | 6.247514324 | 1.603755678 | -3.640351398 |
| C | 3.975412867 | 3.513395436 | 0.541633327 |
| C | 4.201981980 | 4.069705734 | -0.723959949 |
| C | 4.950607753 | 3.683133548 | 1.539263747 |
| C | 5.383110428 | 4.765966018 | -0.988219745 |
| C | 6.128178204 | 4.375849263 | 1.271031839 |
| C | 6.350467988 | 4.918622556 | 0.003393447 |
| H | 3.461116736 | 3.982864914 | -1.509180674 |
| H | 4.788009744 | 3.289925575 | 2.535577738 |
| H | 5.539107911 | 5.195185129 | -1.973924416 |
| H | 6.867497107 | 4.497879982 | 2.057520394 |
| H | 7.265604201 | 5.465443215 | -0.205153424 |
| C | 1.871830700 | 3.694088650 | 2.411223733 |
| C | 1.502590722 | 3.157983139 | 3.651714284 |
| C | 1.879374620 | 5.092786802 | 2.255590647 |
| C | 1.139911358 | 3.995679691 | 4.709898122 |
| C | 1.510129409 | 5.925720831 | 3.308735843 |
| C | 1.139739172 | 5.379675960 | 4.541822888 |
| H | 1.490321979 | 2.087165902 | 3.800862035 |
| H | 2.183751815 | 5.528354362 | 1.308296141 |
| H | 0.858983327 | 3.560620196 | 5.664918112 |
| H | 1.518977790 | 7.002906676 | 3.169849489 |
| H | 0.858465724 | 6.030975106 | 5.363934124 |
| C | 1.257587693 | 3.017631526 | -0.359740014 |
| C | 1.527110820 | 2.461955875 | -1.624507421 |
| C | 0.064564569 | 3.728281873 | -0.173805868 |
| C | 0.634148616 | 2.633049277 | -2.679804714 |
| C | -0.836758499 | 3.883965543 | -1.230830940 |
| C | -0.554888178 | 3.341439784 | -2.484068199 |
| H | 2.437526833 | 1.887830150 | -1.782683940 |
| H | -0.164963580 | 4.168838828 | 0.791151801 |
| H | 0.862085053 | 2.207747385 | -3.653169067 |
| H | -1.758294011 | 4.436764885 | -1.071833006 |
| H | -1.255436366 | 3.468354545 | -3.304003945 |

==============================

9A

==============================

| | | | |
|---|---|---|---|
| Rh | 0.663257432 | 1.160847339 | 0.563500252 |
| C | -0.455414611 | -0.640883570 | -0.743290678 |
| C | 0.072675051 | 2.530973183 | -0.909139060 |
| C | 0.059012123 | 0.195608797 | -1.692332135 |
| C | -0.434104225 | 1.613439363 | -2.012754057 |
| C | 0.164069927 | 1.834953673 | -3.429000272 |
| C | 1.019182964 | -0.176695926 | -2.804102601 |
| O | 0.505888054 | 0.536051887 | -3.917509681 |
| H | -0.647606393 | 3.262293395 | -0.545878241 |
| H | 1.008870854 | 3.024326881 | -1.194503327 |
| H | -0.114864280 | -1.676528350 | -0.710429720 |
| H | 2.049792193 | 0.151410513 | -2.574472173 |
| H | 1.036297834 | -1.244810732 | -3.033969093 |
| H | -0.535443686 | 2.285661660 | -4.135778953 |
| H | 1.065204712 | 2.467663183 | -3.368614090 |
| H | -1.528139730 | 1.608274797 | -2.045564779 |
| C | -1.574997318 | -0.251394012 | 0.159883717 |
| C | -1.289961226 | 0.857373002 | 0.864305182 |
| C | -2.799200729 | -1.120809773 | 0.215329435 |
| C | -2.231167927 | 1.595350656 | 1.726776611 |
| P | 1.265766103 | 2.855240217 | 2.017452287 |
| H | -3.566721542 | -0.682505477 | 0.852882094 |
| H | -3.201276993 | -1.292641595 | -0.789674023 |
| H | -2.525032147 | -2.095229594 | 0.637941576 |
| C | 1.124249042 | 2.512901843 | 3.811474554 |
| C | 2.194284587 | 2.712223224 | 4.694314351 |
| H | 3.169767808 | 3.001551549 | 4.321307829 |
| C | 2.018739897 | 2.514387015 | 6.064238508 |
| H | 2.860643542 | 2.658839385 | 6.734079930 |
| C | 0.780965911 | 2.117049143 | 6.571383524 |
| H | 0.651688603 | 1.963399998 | 7.638507263 |
| C | -0.287868994 | 1.911907645 | 5.696954483 |
| H | -1.256460468 | 1.598615386 | 6.075936218 |
| C | -0.117245882 | 2.105994114 | 4.326803717 |
| H | -0.962034287 | 1.949510071 | 3.670071809 |
| C | 3.043006150 | 3.061732833 | 1.601671868 |
| C | 3.785795950 | 4.170192987 | 2.038059380 |
| H | 3.319379028 | 4.921780721 | 2.668162554 |
| C | 5.118571859 | 4.320126047 | 1.653623501 |
| H | 5.684364220 | 5.177392923 | 2.006199354 |
| C | 5.721624984 | 3.383974792 | 0.808948622 |
| H | 6.757013220 | 3.510051079 | 0.507427796 |
| C | 4.985622946 | 2.290721119 | 0.347633368 |
| H | 5.440549271 | 1.557410184 | -0.311738848 |
| C | 3.657347760 | 2.135333322 | 0.743108715 |
| H | 3.114475362 | 1.260179802 | 0.387165396 |
| C | 0.618168516 | 4.561689855 | 1.840631212 |
| C | -0.139092390 | 5.174246254 | 2.849345574 |
| H | -0.365544758 | 4.640986921 | 3.765679474 |
| C | -0.601681535 | 6.481495485 | 2.688125611 |
| H | -1.178673184 | 6.945458733 | 3.483083411 |
| C | -0.320139605 | 7.191421319 | 1.520192680 |
| H | -0.682299647 | 8.207712938 | 1.397661820 |
| C | 0.445747478 | 6.592482701 | 0.516577059 |
| H | 0.685331477 | 7.142057981 | -0.388934353 |
| C | 0.917348447 | 5.291280024 | 0.676420211 |
| H | 1.540112023 | 4.855815963 | -0.096681443 |
| O | -2.104990474 | 2.932279388 | 1.609024503 |
| O | -3.037470294 | 1.061197042 | 2.469354920 |
| C | -3.051889754 | 3.710261827 | 2.372845696 |
| H | -4.073580846 | 3.419061554 | 2.119849072 |
| H | -2.856807219 | 4.746254257 | 2.103563084 |
| H | -2.892687239 | 3.556887296 | 3.443983809 |
| P | 1.594762667 | -0.800939394 | 1.987647993 |
| C | 2.780494776 | -1.838460313 | 1.037896388 |
| C | 3.476233558 | -2.896534342 | 1.649385336 |
| C | 3.022675758 | -1.567778563 | -0.314215748 |
| C | 4.370696317 | -3.670830830 | 0.913100841 |
| C | 3.925210980 | -2.337422269 | -1.051193679 |
| C | 4.597650504 | -3.395295676 | -0.439373147 |
| H | 3.318980517 | -3.109678272 | 2.702656532 |
| H | 2.503689403 | -0.748076843 | -0.794924889 |

H 4.896223093 -4.489224902 1.396541651
H 4.100003168 -2.109460901 -2.099026192
H 5.298361397 -3.999037895 -1.008105053
C 2.545555572 -0.582394167 3.555832651
C 2.049330055 -0.952081706 4.812337936
C 3.849513888 -0.063115545 3.468784574
C 2.841325953 -0.807280355 5.954045729
C 4.634599641 0.080770453 4.610203552
C 4.133933272 -0.292288849 5.860301052
H 1.051659520 -1.365469309 4.909319610
H 4.265662712 0.212318576 2.505203106
H 2.441240138 -1.104231625 6.919315388
H 5.642297748 0.476838995 4.520382609
H 4.748544345 -0.189014988 6.749783512
C 0.213162710 -1.918866826 2.434751032
C 0.148392993 -3.262211120 2.037816300
C -0.853779259 -1.372600552 3.167727036
C -0.951810425 -4.046249257 2.392346430
C -1.946323410 -2.158130663 3.525184909
C -1.996460330 -3.499843554 3.139584366
H 0.953588899 -3.704703910 1.460979618
H -0.837864600 -0.328511073 3.455578748
H -0.989014171 -5.087328846 2.084446947
H -2.764856468 -1.712560361 4.082035714
H -2.850517450 -4.113296741 3.411677301
===============================
9B
===============================
Rh 0.336743628 1.120461713 1.065590700
C -1.182179574 -0.592937237 -0.028767691
C 0.737520480 1.883363028 -0.829082827
C -0.088126400 -0.494186949 -0.837850808
C 0.339824918 0.706695360 -1.700558047
C 1.485305239 0.077235062 -2.531760406
C 0.749528770 -1.677708506 -1.290638308
O 1.194920654 -1.312379351 -2.592151577
H 0.191452328 2.797294144 -1.053083425
H 1.816027893 2.073346077 -0.810152501
H -1.432225729 -1.576923899 0.357791539
H 1.603425220 -1.849438645 -0.619743131
H 0.170168873 -2.602403071 -1.361118055
H 1.543797238 0.460563108 -3.552803394
H 2.450725739 0.250220169 -2.027741804
H -0.504151397 0.968953798 -2.350331440
C -2.103170202 0.511085070 0.317611576
C -1.530352974 1.697569441 0.661034895
C -3.565797773 0.235992069 0.391685287
C -2.174859984 3.030427099 0.830757688
H -2.095758843 3.387961092 1.862702942
H -1.663905246 3.774087317 0.211010307
H -3.231910184 2.986407603 0.561275636
P 1.063792106 2.958357574 2.232326402
C 0.461346972 2.870518339 3.958018905
C 1.147913187 3.488182478 5.015607378
H 2.087209805 3.999879722 4.831393833
C 0.628911119 3.444555524 6.309051673
H 1.172455198 3.916538844 7.122012153
C -0.587062063 2.803783819 6.557918940
H -0.990940067 2.777231168 7.565821695
C -1.284849425 2.201694222 5.509144241
H -2.234546022 1.708600746 5.695547645
C -0.762060697 2.230632020 4.216287446
H -1.311747498 1.755521086 3.408619549
C 2.901291849 2.929798965 2.295365378
C 3.661431892 3.480136287 1.249753916
H 3.189037086 4.061834016 0.468006914
C 5.045098066 3.305567723 1.212796844
H 5.615977391 3.743014761 0.398822372
C 5.694211073 2.583976151 2.217194240
H 6.770901409 2.447536119 2.183958885
C 4.948222173 2.050065335 3.268426683
H 5.434118811 1.490085903 4.061534103
C 3.564352243 2.218286094 3.308411873
H 3.009162894 1.783483999 4.129010112
C 0.592285780 4.650262917 1.677779552
C -0.055849492 5.531985371 2.557835662
H -0.273394239 5.231611204 3.576051192
C -0.435252470 6.806386782 2.133945201
H -0.936361009 7.471598542 2.830959036
C -0.176961943 7.223805938 0.828674988
H -0.474765167 8.215275797 0.501714134
C 0.464585170 6.354280392 -0.055808524
H 0.668890101 6.665403718 -1.076065926
C 0.840479169 5.078452255 0.361108345
H 1.317476080 4.422024034 -0.354758010
O -4.420496812 1.024285732 0.749495105
O -3.835353529 -1.038408488 0.029258897
C -5.224028888 -1.418439455 0.095393624
H -5.257929131 -2.459461781 -0.224913794
H -5.601399530 -1.313529167 1.116120415
H -5.823294380 -0.790010059 -0.567979810
P 1.708791171 -0.703251107 2.402402935
C 3.166391387 -1.233468972 1.428795773
C 3.766147195 -2.501104509 1.543115336
C 3.737979974 -0.293026279 0.556901133
C 4.873438121 -2.831615986 0.762036887
C 4.853779498 -0.622044189 -0.213145508
C 5.414663539 -1.896895500 -0.124957065
H 3.380712002 -3.225280574 2.253793463
H 3.318271870 0.705599426 0.491790462
H 5.317913559 -3.818409674 0.852018369
H 5.283040756 0.120061996 -0.879862108
H 6.275201127 -2.158719868 -0.733078565
C 2.317940279 -0.706708850 4.149667336
C 1.438084375 -0.352167216 5.185989844
C 3.647704102 -1.015822739 4.476834354
C 1.867093605 -0.337059121 6.512058249
C 4.074919492 -0.994314527 5.805917033
C 3.187968312 -0.659855946 6.829913604
H 0.413573730 -0.076366325 4.972221387
H 4.359881298 -1.270150559 3.700791423
H 1.165075935 -0.065945967 7.294754872
H 5.105999586 -1.246232899 6.037807991
H 3.522593375 -0.647716770 7.862775492
C 0.462860086 -2.068709989 2.404537058
C 0.597771742 -3.285327566 1.722557160
C -0.752571011 -1.796450879 3.060359027
C -0.448179237 -4.211857803 1.713526756
C -1.789331679 -2.725967735 3.059380856
C -1.639877691 -3.939821109 2.384382795
H 1.503590859 -3.516264723 1.176283117
H -0.909296396 -0.839019637 3.548029028

| | | | |
|---|---|---|---|
| H | -0.326230070 | -5.148401266 | 1.176830560 |
| H | -2.719415043 | -2.495477832 | 3.570620596 |
| H | -2.449639483 | -4.663150049 | 2.374356356 |

==============================

11A

==============================

| | | | |
|---|---|---|---|
| Rh | -0.863794980 | 2.297718236 | 1.285748203 |
| P | 0.900108227 | 2.313798843 | 2.862573586 |
| C | -0.810915400 | 0.363580671 | 0.822929867 |
| C | -0.306225447 | -0.265290563 | -0.231779904 |
| C | 0.498147657 | 0.294279397 | -1.391028686 |
| C | 1.158818797 | -0.994835232 | -1.919907337 |
| C | -0.394338531 | -1.781112899 | -0.446773821 |
| O | 0.186453553 | -2.014816787 | -1.722903211 |
| C | 0.369432757 | 1.121286594 | 4.149030125 |
| C | -0.116193344 | 1.541596213 | 5.396442544 |
| H | -0.104489696 | 2.593352898 | 5.662978220 |
| C | -0.619779462 | 0.606412472 | 6.304177513 |
| H | -0.989655336 | 0.943269252 | 7.268361223 |
| C | -0.640378051 | -0.751540850 | 5.980940673 |
| H | -1.033146521 | -1.475057868 | 6.689063850 |
| C | -0.142251453 | -1.177575921 | 4.745628562 |
| H | -0.146384541 | -2.233340974 | 4.490865626 |
| C | 0.354092209 | -0.250112808 | 3.832305005 |
| H | 0.721488682 | -0.588282155 | 2.868129103 |
| C | 2.666411172 | 2.030162160 | 2.516803598 |
| C | 3.413790010 | 1.037164375 | 3.166897841 |
| H | 2.956397975 | 0.413370646 | 3.927477454 |
| C | 4.758289834 | 0.849342494 | 2.840536858 |
| H | 5.327986584 | 0.076432484 | 3.347971577 |
| C | 5.368881673 | 1.650393203 | 1.874913199 |
| H | 6.414379433 | 1.499029849 | 1.624128752 |
| C | 4.630972564 | 2.649345665 | 1.233274752 |
| H | 5.100001341 | 3.277746829 | 0.482097638 |
| C | 3.287062404 | 2.838901789 | 1.546471194 |
| H | 2.719343294 | 3.607189292 | 1.031344384 |
| C | 0.783013660 | 3.977181138 | 3.622743263 |
| C | 1.912770551 | 4.706911688 | 4.018555466 |
| H | 2.904052045 | 4.278921196 | 3.911204727 |
| C | 1.767998381 | 5.991252494 | 4.546805036 |
| H | 2.650954423 | 6.546138942 | 4.849853072 |
| C | 0.501715823 | 6.563078851 | 4.683782247 |
| H | 0.397554222 | 7.565198630 | 5.088447561 |
| C | -0.630477853 | 5.840498868 | 4.298190499 |
| H | -1.621153457 | 6.275341938 | 4.392692648 |
| C | -0.491204636 | 4.557388102 | 3.774453231 |
| H | -1.386954082 | 4.010498429 | 3.486308861 |
| H | -1.367547118 | -0.175862471 | 1.595422006 |
| H | 0.159567730 | -2.315004780 | 0.344851582 |
| H | -1.421568315 | -2.160427579 | -0.459475170 |
| H | 1.406516183 | -0.956742668 | -2.983512652 |
| H | 2.080736929 | -1.208928876 | -1.349900386 |
| H | -0.182267829 | 0.671551808 | -2.167622084 |
| C | 0.122734052 | 3.303424151 | -0.085909026 |
| C | 0.989481795 | 2.819927322 | -0.999775568 |
| C | 1.506907500 | 1.389258605 | -1.017122603 |
| C | -0.288513903 | 4.728436461 | 0.160977721 |
| C | 1.482996036 | 3.770298853 | -2.044995618 |
| H | 1.916174583 | 1.149588107 | -0.031171255 |
| H | 2.339741988 | 1.344200946 | -1.720413615 |
| H | -0.955071186 | 4.828728414 | 1.028963759 |
| H | 0.593794715 | 5.346933351 | 0.343911953 |
| H | -0.808186123 | 5.144118541 | -0.706324447 |
| O | 1.338651988 | 4.981899361 | -2.041197234 |
| O | 2.116560760 | 3.120614538 | -3.050548779 |
| C | 2.611379238 | 3.960249798 | -4.109973576 |
| H | 3.083376430 | 3.283704661 | -4.822471648 |
| H | 1.790444750 | 4.506920067 | -4.581127038 |
| H | 3.336181731 | 4.681050029 | -3.722475645 |
| P | -3.162182414 | 2.439841603 | 0.556636894 |
| C | -3.994358860 | 1.382762954 | 1.804284333 |
| C | -5.184327508 | 0.694294505 | 1.537042908 |
| C | -3.387324866 | 1.238342791 | 3.065688503 |
| C | -5.756958797 | -0.115187005 | 2.519123747 |
| C | -3.957744416 | 0.426068359 | 4.042895791 |
| C | -5.147798018 | -0.251302534 | 3.768160858 |
| H | -5.661361800 | 0.784939303 | 0.566018717 |
| H | -2.458704234 | 1.758160214 | 3.302051105 |
| H | -6.681495744 | -0.643444499 | 2.305367727 |
| H | -3.466775206 | 0.319821835 | 5.005625956 |
| H | -5.598093936 | -0.886919420 | 4.524874738 |
| C | -3.709023432 | 4.152036656 | 0.915774141 |
| C | -4.072714438 | 4.534397772 | 2.216481194 |
| C | -3.619294144 | 5.130285666 | -0.089143566 |
| C | -4.337220770 | 5.874002650 | 2.504949418 |
| C | -3.888640483 | 6.465263216 | 0.204704484 |
| C | -4.241644841 | 6.841116602 | 1.503103439 |
| H | -4.166473867 | 3.789873676 | 3.001021917 |
| H | -3.344778600 | 4.847874771 | -1.101369806 |
| H | -4.631436928 | 6.158646895 | 3.511349993 |
| H | -3.821021321 | 7.212374597 | -0.580496416 |
| H | -4.449573595 | 7.882605089 | 1.729699689 |
| C | -3.873824790 | 2.000121338 | -1.060111093 |
| C | -3.225325618 | 1.037855405 | -1.847646245 |
| C | -5.085698578 | 2.557111911 | -1.504243809 |
| C | -3.778862979 | 0.639905559 | -3.064283187 |
| C | -5.630819972 | 2.156713332 | -2.723729830 |
| C | -4.979674851 | 1.198973929 | -3.505772737 |
| H | -2.302085459 | 0.595365208 | -1.497188509 |
| H | -5.598808674 | 3.302694898 | -0.904454998 |
| H | -3.269225075 | -0.107069719 | -3.665695700 |
| H | -6.566144530 | 2.592837612 | -3.062013156 |
| H | -5.407826654 | 0.890919107 | -4.454937575 |

==============================

11B

==============================

| | | | |
|---|---|---|---|
| Rh | -0.690332312 | 2.599389798 | 1.510917244 |
| P | 1.070829781 | 2.292536057 | 3.103472238 |
| C | -1.131295967 | 0.677615086 | 1.404893389 |
| C | -0.894448772 | -0.222348726 | 0.456340266 |
| C | -0.055912709 | -0.062520970 | -0.798597191 |
| C | 0.101335670 | -1.538375745 | -1.229826805 |
| C | -1.408475669 | -1.659250325 | 0.467912421 |
| O | -1.091656606 | -2.184267708 | -0.817254848 |
| C | 0.888019950 | 0.852719778 | 4.216178854 |
| C | 0.805361755 | 1.009875284 | 5.608506788 |
| H | 0.868701844 | 1.997715491 | 6.052546232 |
| C | 0.643175508 | -0.105853867 | 6.431471735 |
| H | 0.579203427 | 0.028686445 | 7.507301641 |
| C | 0.567776524 | -1.385728795 | 5.879663187 |
| H | 0.442028126 | -2.250780088 | 6.523847249 |
| C | 0.652484505 | -1.548831192 | 4.494326396 |

| | | | |
|---|---|---|---|
| H | 0.594021289 | -2.541229287 | 4.056386516 |
| C | 0.803768167 | -0.438876201 | 3.664914578 |
| H | 0.838504710 | -0.573501215 | 2.589540165 |
| C | 2.779753254 | 2.247544908 | 2.449436966 |
| C | 3.570155178 | 1.091620928 | 2.485682455 |
| H | 3.210961961 | 0.197389685 | 2.984644718 |
| C | 4.832852002 | 1.084629085 | 1.887296824 |
| H | 5.437140823 | 0.182974075 | 1.926452441 |
| C | 5.320462237 | 2.227528513 | 1.252960863 |
| H | 6.303747735 | 2.219050360 | 0.792180903 |
| C | 4.542890566 | 3.389793305 | 1.228923870 |
| H | 4.920537079 | 4.289443102 | 0.751532801 |
| C | 3.280852691 | 3.400183305 | 1.817902458 |
| H | 2.691028002 | 4.311577419 | 1.809069421 |
| C | 0.998180404 | 3.767379730 | 4.187696602 |
| C | 2.140519127 | 4.319555372 | 4.785707058 |
| H | 3.117640656 | 3.884325861 | 4.602463100 |
| C | 2.026369689 | 5.435858320 | 5.615059138 |
| H | 2.916448741 | 5.857794407 | 6.072598364 |
| C | 0.776182186 | 6.007391860 | 5.858630031 |
| H | 0.693019126 | 6.877727230 | 6.503072596 |
| C | -0.367009056 | 5.457763918 | 5.274614196 |
| H | -1.344133561 | 5.894407566 | 5.460416590 |
| C | -0.256404038 | 4.344666132 | 4.444510831 |
| H | -1.156062997 | 3.935508472 | 3.991675068 |
| H | -1.701791490 | 0.384858924 | 2.296982339 |
| H | -0.917821126 | -2.247193076 | 1.265017301 |
| H | -2.492138682 | -1.723767054 | 0.607947515 |
| H | 0.212812666 | -1.672751640 | -2.309207613 |
| H | 0.984255585 | -1.978912412 | -0.730529078 |
| H | -0.638243882 | 0.468923991 | -1.565337067 |
| C | 0.592867916 | 2.952743255 | 0.006548928 |
| C | 1.346803269 | 2.135777579 | -0.752046220 |
| C | 1.311546308 | 0.627155414 | -0.607283778 |
| C | 0.585384358 | 4.414035058 | 0.118798461 |
| C | 2.351417267 | 2.631291501 | -1.765885983 |
| H | 1.703339981 | 0.365724658 | 0.384942793 |
| H | 2.016657515 | 0.206531249 | -1.333528984 |
| H | 3.349033884 | 2.279795694 | -1.470725471 |
| H | 2.134650149 | 2.185967622 | -2.745391894 |
| H | 2.377069237 | 3.712100910 | -1.868779977 |
| O | 1.110204196 | 5.183156541 | -0.824195079 |
| O | 0.036898859 | 4.887392510 | 1.135286914 |
| C | 0.944447448 | 6.613886780 | -0.655076774 |
| H | 1.396581800 | 6.941867629 | 0.283429609 |
| H | -0.119210188 | 6.860580875 | -0.661372832 |
| H | 1.450579228 | 7.060904217 | -1.509331597 |
| P | -2.669499019 | 3.206526087 | 0.264960384 |
| C | -2.353051308 | 4.231528454 | -1.214594875 |
| C | -1.579901382 | 3.686390400 | -2.254619876 |
| C | -2.819534361 | 5.549355668 | -1.324705787 |
| C | -1.284907599 | 4.445007824 | -3.384964814 |
| C | -2.514606574 | 6.307145534 | -2.456917812 |
| C | -1.748028848 | 5.758696101 | -3.486355111 |
| H | -1.203885747 | 2.672257748 | -2.174902807 |
| H | -3.420015033 | 5.984409395 | -0.532706678 |
| H | -0.688312355 | 4.012850639 | -4.182954276 |
| H | -2.883367843 | 7.325849489 | -2.535455128 |
| H | -1.513975385 | 6.351653607 | -4.365761748 |
| C | -3.642254829 | 4.262992033 | 1.414898268 |
| C | -2.964335915 | 5.261659169 | 2.140884936 |

| | | | |
|---|---|---|---|
| C | -5.026674742 | 4.111165691 | 1.588672661 |
| C | -3.663908217 | 6.086440756 | 3.021308216 |
| C | -5.716718695 | 4.937135119 | 2.477798507 |
| C | -5.040008764 | 5.923568269 | 3.197837960 |
| H | -1.899195302 | 5.408138937 | 2.002952764 |
| H | -5.568641264 | 3.354313784 | 1.031701560 |
| H | -3.129134078 | 6.859411280 | 3.566371432 |
| H | -6.788207056 | 4.810672492 | 2.602491799 |
| H | -5.582634026 | 6.564469654 | 3.886366562 |
| C | -3.796413579 | 1.865498679 | -0.252883264 |
| C | -4.183717584 | 1.667219742 | -1.584690574 |
| C | -4.264356355 | 0.979946833 | 0.733970775 |
| C | -5.011471079 | 0.593665725 | -1.922957736 |
| C | -5.101095141 | -0.079798297 | 0.394063106 |
| C | -5.469625088 | -0.282500665 | -0.939742907 |
| H | -3.848154896 | 2.345246081 | -2.361914870 |
| H | -3.985483078 | 1.126084015 | 1.773141834 |
| H | -5.300852445 | 0.448911981 | -2.959689097 |
| H | -5.463083269 | -0.749396011 | 1.168880920 |
| H | -6.113258489 | -1.114939517 | -1.207249071 |

==============================

9A-TS

==============================

| | | | |
|---|---|---|---|
| C | -0.493124319 | 0.262189742 | -0.140336567 |
| C | -0.174517785 | -0.279495137 | 1.878056151 |
| Rh | 1.540840703 | 0.041117631 | 0.802031807 |
| C | 0.805438412 | -2.227884112 | 0.972776282 |
| C | 0.696817252 | -1.898288256 | -0.357242261 |
| C | -0.453239976 | -1.047541892 | -0.897989342 |
| C | -0.071474334 | -0.971805100 | -2.391161721 |
| C | 1.409979955 | -2.507876839 | -1.549562098 |
| O | 0.569469880 | -2.210996163 | -2.663582682 |
| H | -1.461541803 | 0.564962175 | 0.245750737 |
| H | -0.056564094 | 1.100703281 | -0.687907290 |
| H | 1.482676166 | -3.025199356 | 1.268862034 |
| H | 2.410362764 | -2.072397981 | -1.678919094 |
| H | 1.521947276 | -3.593649975 | -1.475126748 |
| H | -0.928440868 | -0.876251655 | -3.061481386 |
| H | 0.612392465 | -0.121994785 | -2.562514190 |
| H | -1.386228279 | -1.613274895 | -0.782565653 |
| C | -0.027104081 | -1.621202648 | 2.032564996 |
| P | 1.890215353 | 2.321284192 | 1.123887740 |
| C | 3.097823010 | 2.732392805 | 2.432511016 |
| C | 4.216370075 | 3.551084717 | 2.235121528 |
| H | 4.442140511 | 3.951248775 | 1.254044533 |
| C | 5.074571662 | 3.824904601 | 3.300040567 |
| H | 5.945226451 | 4.452395674 | 3.135028114 |
| C | 4.829602260 | 3.283653320 | 4.562463110 |
| H | 5.504037954 | 3.497840358 | 5.386598482 |
| C | 3.719128421 | 2.461152494 | 4.762296756 |
| H | 3.522446745 | 2.031441355 | 5.740164926 |
| C | 2.855310795 | 2.185370876 | 3.704324271 |
| H | 1.979933610 | 1.565932198 | 3.874547365 |
| C | 2.409147001 | 3.131180775 | -0.442119329 |
| C | 2.601764974 | 4.522106107 | -0.493502228 |
| H | 2.477561806 | 5.121243532 | 0.403804629 |
| C | 2.933889385 | 5.142833663 | -1.696287361 |
| H | 3.087394192 | 6.217714590 | -1.721068351 |
| C | 3.053183228 | 4.390099569 | -2.868474228 |
| H | 3.302234262 | 4.878627589 | -3.805669686 |
| C | 2.835172110 | 3.012009010 | -2.831782607 |

| | | | |
|---|---|---|---|
| H | 2.911010033 | 2.422150269 | -3.740707973 |
| C | 2.514476200 | 2.386422642 | -1.625153069 |
| H | 2.346836254 | 1.312582957 | -1.596613611 |
| C | 0.429098776 | 3.375149382 | 1.536521559 |
| C | 0.274862760 | 4.002478205 | 2.781346407 |
| H | 0.994859636 | 3.838492508 | 3.573798602 |
| C | -0.796003755 | 4.870089563 | 3.006010051 |
| H | -0.890186536 | 5.361695353 | 3.970223914 |
| C | -1.733820276 | 5.109386790 | 2.002271580 |
| H | -2.562912572 | 5.788092040 | 2.180385569 |
| C | -1.594812210 | 4.480949057 | 0.762575661 |
| H | -2.313032363 | 4.669846846 | -0.029925580 |
| C | -0.518939827 | 3.628571239 | 0.528745121 |
| H | -0.398983209 | 3.194299418 | -0.458205265 |
| P | 3.834124144 | -0.621414400 | 1.179500233 |
| C | 3.936573037 | -1.350663807 | 2.863015378 |
| C | 2.912385300 | -1.148668256 | 3.798484935 |
| C | 5.056405028 | -2.118233771 | 3.233663850 |
| C | 3.008243816 | -1.693006159 | 5.080365743 |
| C | 5.150540904 | -2.656420740 | 4.515234206 |
| C | 4.126820394 | -2.443154410 | 5.442346780 |
| H | 2.032082400 | -0.577621145 | 3.531532541 |
| H | 5.850546385 | -2.302208057 | 2.516318066 |
| H | 2.204318414 | -1.526016436 | 5.791532576 |
| H | 6.021183463 | -3.245433205 | 4.788425998 |
| H | 4.201242759 | -2.865919192 | 6.440152666 |
| C | 4.381458711 | -2.018719288 | 0.098279810 |
| C | 5.033865671 | -1.784731890 | -1.122287886 |
| C | 4.069357112 | -3.343665336 | 0.447087233 |
| C | 5.351387053 | -2.843824517 | -1.973479678 |
| C | 4.390481213 | -4.400332337 | -0.403750444 |
| C | 5.026076851 | -4.153910522 | -1.621643018 |
| H | 5.315984175 | -0.779403447 | -1.411969840 |
| H | 3.601024546 | -3.561293396 | 1.401229820 |
| H | 5.859806777 | -2.641054154 | -2.911547020 |
| H | 4.149140063 | -5.417788544 | -0.109445147 |
| H | 5.274305018 | -4.976187406 | -2.285763958 |
| C | 5.232256590 | 0.565015513 | 1.066171893 |
| C | 6.200459273 | 0.702747198 | 2.070180391 |
| C | 5.350519036 | 1.340838862 | -0.098095714 |
| C | 7.277688199 | 1.571418095 | 1.893129643 |
| C | 6.427853517 | 2.206751682 | -0.272924792 |
| C | 7.400913714 | 2.318559267 | 0.721437320 |
| H | 6.113928541 | 0.151631649 | 2.998783037 |
| H | 4.593255124 | 1.286235810 | -0.870143849 |
| H | 8.019082003 | 1.667961879 | 2.681000838 |
| H | 6.498256152 | 2.797316297 | -1.181941840 |
| H | 8.242896682 | 2.991739522 | 0.588831405 |
| C | -0.434461158 | -2.444612503 | 3.223274269 |
| H | -1.098485421 | -3.265787104 | 2.929359922 |
| H | 0.456761596 | -2.890213937 | 3.682828432 |
| H | -0.932830504 | -1.837562040 | 3.981114447 |
| C | -0.834918522 | 0.608647672 | 2.869153526 |
| O | -0.366728029 | 0.797846520 | 3.978785897 |
| O | -1.985247671 | 1.132196033 | 2.432690183 |
| C | -2.723750027 | 1.927975299 | 3.390069438 |
| H | -3.141801138 | 1.277155939 | 4.162387326 |
| H | -2.071301660 | 2.671714495 | 3.845376604 |
| H | -3.516144050 | 2.407659182 | 2.818149531 |

==============================

9B-TS

==============================

| | | | |
|---|---|---|---|
| C | -0.522989493 | 0.239660435 | -0.085087439 |
| C | -0.199743581 | -0.302112473 | 1.917512989 |
| Rh | 1.514587141 | 0.056547894 | 0.851115902 |
| C | 0.845732291 | -2.229844437 | 0.932708002 |
| C | 0.721719009 | -1.880041414 | -0.389189953 |
| C | -0.427443393 | -1.023884277 | -0.916788719 |
| C | 0.002084243 | -0.849955902 | -2.387416484 |
| C | 1.462448796 | -2.432497256 | -1.592498784 |
| O | 0.662080960 | -2.064794742 | -2.715426500 |
| H | -1.511845942 | 0.454228464 | 0.311506965 |
| H | -0.167035607 | 1.138443199 | -0.591578248 |
| H | 1.543094805 | -3.010893459 | 1.216301659 |
| H | 2.471505343 | -2.007162606 | -1.670937847 |
| H | 1.556184936 | -3.522683823 | -1.572417744 |
| H | -0.832154785 | -0.716830091 | -3.079824582 |
| H | 0.683542401 | 0.014486828 | -2.479086780 |
| H | -1.349939326 | -1.616693795 | -0.870735518 |
| C | -0.000924432 | -1.655783657 | 2.000147758 |
| C | -0.292922931 | -2.425835985 | 3.229915064 |
| C | -1.015292305 | 0.551854162 | 2.829701396 |
| H | -0.461427526 | 0.725748745 | 3.758880548 |
| H | -1.253768778 | 1.516767249 | 2.385559055 |
| H | -1.935131709 | 0.033945150 | 3.115341308 |
| P | 1.853484043 | 2.328962068 | 1.193192671 |
| C | 2.985293564 | 2.725923324 | 2.575271793 |
| C | 4.016542381 | 3.670173998 | 2.507709702 |
| H | 4.236779176 | 4.182374770 | 1.578886202 |
| C | 4.798767946 | 3.928563303 | 3.633375230 |
| H | 5.605685859 | 4.652045633 | 3.567363444 |
| C | 4.560357722 | 3.255250254 | 4.832289989 |
| H | 5.175042885 | 3.461119701 | 5.703630196 |
| C | 3.538521216 | 2.307364904 | 4.904758634 |
| H | 3.351793030 | 1.768461834 | 5.829090129 |
| C | 2.759040889 | 2.042585911 | 3.780973897 |
| H | 1.968598333 | 1.302448151 | 3.839973978 |
| C | 2.465062329 | 3.122140707 | -0.345691362 |
| C | 2.628831699 | 4.515492307 | -0.423363251 |
| H | 2.401985927 | 5.139191804 | 0.436363552 |
| C | 3.062662084 | 5.108257057 | -1.608588482 |
| H | 3.194693382 | 6.185434044 | -1.653287848 |
| C | 3.312817961 | 4.323859731 | -2.738355744 |
| H | 3.643942787 | 4.790495836 | -3.661239067 |
| C | 3.118460955 | 2.942351804 | -2.680364323 |
| H | 3.292578653 | 2.328238187 | -3.559218221 |
| C | 2.694726574 | 2.345618979 | -1.491316783 |
| H | 2.545393429 | 1.269174277 | -1.446146463 |
| C | 0.369785697 | 3.358360473 | 1.547446851 |
| C | 0.069144120 | 3.818813109 | 2.838185388 |
| H | 0.740462999 | 3.614306127 | 3.664621499 |
| C | -1.097419460 | 4.547625714 | 3.071951619 |
| H | -1.315089012 | 4.901250920 | 4.075604486 |
| C | -1.977255674 | 4.824857757 | 2.025213934 |
| H | -2.884707121 | 5.391731485 | 2.211352838 |
| C | -1.679128392 | 4.382785290 | 0.734236151 |
| H | -2.349215628 | 4.610759283 | -0.089535418 |
| C | -0.512722396 | 3.659095922 | 0.494973032 |
| H | -0.276244513 | 3.355759267 | -0.520509819 |
| O | -0.758106845 | -1.961577899 | 4.257380385 |
| O | 0.082996340 | -3.717597906 | 3.094372118 |
| C | -0.063701951 | -4.534662581 | 4.271794901 |

H 0.189233346 -5.547431903 3.958823822
H 0.620899225 -4.193549968 5.053481732
H -1.088325737 -4.488100478 4.647285465
P 3.817504805 -0.591782427 1.271900524
C 3.873487749 -1.502197469 2.868857836
C 2.817553591 -1.408618812 3.783604399
C 4.983769860 -2.303893699 3.194677802
C 2.854193028 -2.107131878 4.990923407
C 5.023722955 -2.994557301 4.404001010
C 3.957767965 -2.900568161 5.303354586
H 1.951415503 -0.806519363 3.547761395
H 5.812920281 -2.393501929 2.499231627
H 2.012745267 -2.030534713 5.673165170
H 5.886252937 -3.610473634 4.642020650
H 3.990744024 -3.446795938 6.241740403
C 4.425039519 -1.846868391 0.052873490
C 5.045008222 -1.455774902 -1.145183778
C 4.185427454 -3.215853091 0.262680771
C 5.407460992 -2.403057484 -2.104056267
C 4.555893904 -4.160439564 -0.694554167
C 5.161725879 -3.759122739 -1.886083633
H 5.266134163 -0.413425283 -1.337754755
H 3.727645591 -3.557483190 1.184583269
H 5.888591153 -2.076236901 -3.021492842
H 4.371384399 -5.213803504 -0.503971009
H 5.446584342 -4.494381868 -2.632278946
C 5.199654016 0.620857720 1.374575954
C 6.044201851 0.694850305 2.491868268
C 5.439216877 1.484937707 0.292994265
C 7.121971774 1.580542790 2.504304427
C 6.519721271 2.363977300 0.305630024
C 7.372071790 2.407542866 1.409689195
H 5.864508834 0.073965630 3.361092150
H 4.775375158 1.494515891 -0.561145040
H 7.765789492 1.622940661 3.378038371
H 6.687569632 3.017329920 -0.545796212
H 8.216830741 3.090150162 1.422272851
==============================
11A-TS
==============================
C 0.022389582 -0.208179722 -0.190547093
C 0.229793959 0.036207664 2.212731090
Rh 1.679972268 -0.158811209 1.088332619
P 2.331541784 2.032178448 0.326708281
C -0.595210843 -1.372797353 -0.387220241
C -0.457757745 -2.637019012 0.446762790
C -1.583695353 -3.515753631 -0.144200879
C -1.576366150 -1.694420620 -1.510240533
O -1.695049397 -3.111337169 -1.503248378
C 4.012204597 2.251605350 -0.369416208
C 4.854672717 3.311529317 -0.004070083
H 4.533717161 4.040040358 0.731790359
C 6.126296828 3.419350517 -0.566890616
H 6.773482270 4.241258749 -0.273901626
C 6.572469670 2.472802534 -1.492006555
H 7.568611786 2.555026824 -1.917193926
C 5.735043302 1.420495829 -1.868596735
H 6.073050368 0.673163569 -2.579830857
C 4.461540029 1.314309655 -1.311503761
H 3.823306302 0.487779927 -1.602048547
C 1.241034407 2.809611612 -0.935796460
C 1.625361561 2.914993226 -2.280041922
H 2.610780619 2.591427939 -2.596404782
C 0.751253175 3.458837691 -3.222547959
H 1.066391834 3.542083155 -4.258621409
C -0.512581181 3.904814390 -2.837260456
H -1.187312022 4.333666612 -3.572339173
C -0.904359022 3.803930085 -1.500328962
H -1.883445008 4.156799003 -1.189999416
C -0.036658958 3.256108115 -0.557639742
H -0.349315031 3.197671265 0.480070470
C 2.245907180 3.194030725 1.747388972
C 2.116895510 4.578140026 1.532597005
H 2.016616646 4.966769271 0.523853719
C 2.114799693 5.458987989 2.612582981
H 2.014028840 6.525977101 2.435996370
C 2.242444502 4.971677074 3.915894860
H 2.240971335 5.661159307 4.755314165
C 2.366810983 3.599998663 4.136076417
H 2.464602345 3.211934036 5.145686489
C 2.365053796 2.712970009 3.059766409
H 2.441245867 1.649641835 3.252951693
H -0.201501252 0.674436964 -0.782867053
H -2.550534073 -1.213352589 -1.313215615
H -1.230018646 -1.376409766 -2.497597423
H -1.369451086 -4.586792192 -0.123263177
H -2.529429967 -3.335767452 0.397105379
H 0.510952705 -3.106130806 0.227505470
C 0.376843513 -1.341892878 2.522471628
C -0.566448546 -2.407527857 1.960782770
C -0.595265560 1.123073155 2.755686825
C 0.979095799 -1.506134512 3.872108669
H -1.604104872 -2.137000973 2.201878605
H -0.357323598 -3.341780115 2.486027359
H -0.498208154 1.100506639 3.848294590
H -0.319279435 2.108597934 2.384608138
H -1.645482959 0.909309585 2.510080581
O 0.728587875 -2.700447668 4.423499861
O 1.626406430 -0.627728040 4.437164604
C 1.358560800 -2.947365013 5.698802196
H 1.007626365 -3.931345943 6.008416960
H 2.444004737 -2.946161984 5.579637408
H 1.065870383 -2.183629592 6.423055920
P 3.891166777 -0.880065357 1.893164756
C 3.913133945 -2.436675583 2.875605367
C 4.884261617 -2.697294730 3.853563619
C 3.034893843 -3.460880620 2.495840360
C 4.955822523 -3.955994631 4.452325948
C 3.112148207 -4.717720591 3.091795638
C 4.071349918 -4.967612222 4.074992662
H 5.589059876 -1.928092190 4.148435898
H 2.284445169 -3.273596345 1.734160829
H 5.709003112 -4.145162456 5.211812460
H 2.420048481 -5.499171616 2.792320334
H 4.131024960 -5.946279602 4.542387624
C 4.942749415 0.302455284 2.798800933
C 4.684698337 0.506059878 4.167462546
C 5.904793732 1.092261605 2.153290180
C 5.400453835 1.467637795 4.876535487
C 6.611117167 2.057454474 2.871840699
C 6.365994732 2.244507812 4.231724695
H 3.913488035 -0.071701543 4.667370081

| | | | |
|---|---|---|---|
| H | 6.102533619 | 0.970871292 | 1.094775840 |
| H | 5.199190110 | 1.614043014 | 5.933899860 |
| H | 7.351244272 | 2.664647330 | 2.359345813 |
| H | 6.920615650 | 2.995273393 | 4.787321060 |
| C | 4.799922492 | -1.471091793 | 0.411038762 |
| C | 6.191918323 | -1.629281761 | 0.382424046 |
| C | 4.041823319 | -1.898401156 | -0.690724035 |
| C | 6.815135030 | -2.155577950 | -0.749129671 |
| C | 4.665276554 | -2.433559508 | -1.817945385 |
| C | 6.056981288 | -2.551510183 | -1.854222447 |
| H | 6.792575764 | -1.342259677 | 1.239712287 |
| H | 2.954722479 | -1.811486534 | -0.678633539 |
| H | 7.895808340 | -2.264252327 | -0.763502373 |
| H | 4.066319087 | -2.755634184 | -2.664886311 |
| H | 6.546353029 | -2.965374720 | -2.730800593 |

==============================

11B-TS

==============================

| | | | |
|---|---|---|---|
| C | 0.706572772 | -0.998346687 | 0.214972616 |
| C | -0.028703393 | 0.609878045 | 1.407109281 |
| Rh | 1.978103125 | 0.063684599 | 1.463580092 |
| P | 3.075438012 | 1.170759268 | -0.176469633 |
| C | 0.397525836 | -2.135811830 | 0.838700764 |
| C | 0.334373035 | -2.180659101 | 2.359440406 |
| C | 0.203161129 | -3.703061652 | 2.625326035 |
| C | 0.160128839 | -3.543868241 | 0.341923154 |
| O | 0.638985766 | -4.344518607 | 1.426421658 |
| C | 4.640141304 | 0.309472836 | -0.631663880 |
| C | 5.788154170 | 1.001054289 | -1.047441126 |
| H | 5.795250124 | 2.084036123 | -1.086087601 |
| C | 6.948514145 | 0.300252131 | -1.378236055 |
| H | 7.831833895 | 0.849115280 | -1.692406971 |
| C | 6.984889009 | -1.093821088 | -1.290186310 |
| H | 7.895918371 | -1.632327409 | -1.533779538 |
| C | 5.843807157 | -1.789435180 | -0.884608332 |
| H | 5.859862397 | -2.872011155 | -0.799986656 |
| C | 4.678812443 | -1.092423932 | -0.563474242 |
| H | 3.800317296 | -1.638512390 | -0.236698748 |
| C | 2.182093970 | 1.260752975 | -1.775052516 |
| C | 2.299076741 | 0.215416242 | -2.707109827 |
| H | 2.948396116 | -0.630096028 | -2.505394314 |
| C | 1.600527525 | 0.265179354 | -3.912326379 |
| H | 1.705592881 | -0.545404678 | -4.627654468 |
| C | 0.783785149 | 1.358931641 | -4.205177221 |
| H | 0.248011760 | 1.400956521 | -5.148903560 |
| C | 0.668374113 | 2.403335534 | -3.286356973 |
| H | 0.046609375 | 3.263726889 | -3.518023940 |
| C | 1.355931542 | 2.356721300 | -2.074132436 |
| H | 1.257806952 | 3.170619350 | -1.365432229 |
| C | 3.525045976 | 2.894705757 | 0.216352882 |
| C | 3.958017658 | 3.771658896 | -0.794027134 |
| H | 3.975510800 | 3.448568941 | -1.830397466 |
| C | 4.348593044 | 5.070496335 | -0.473549148 |
| H | 4.678644404 | 5.742105686 | -1.260676972 |
| C | 4.308798751 | 5.507043117 | 0.852406508 |
| H | 4.613373841 | 6.519992925 | 1.099320669 |
| C | 3.863926753 | 4.646117185 | 1.855702893 |
| H | 3.818517332 | 4.984228661 | 2.886468916 |
| C | 3.465809567 | 3.349739116 | 1.537731795 |
| H | 3.090568717 | 2.694311669 | 2.312974362 |
| H | 0.656797578 | -0.799283228 | -0.850191718 |
| H | -0.909496143 | -3.738653617 | 0.152714899 |
| H | 0.726470716 | -3.801072144 | -0.556381877 |
| H | 0.829772246 | -4.063717693 | 3.443760749 |
| H | -0.846931188 | -3.959497537 | 2.841523794 |
| H | 1.277741655 | -1.821894675 | 2.810086069 |
| C | -0.875119514 | 0.101914673 | 2.334451284 |
| C | -0.795412685 | -1.299410245 | 2.906883150 |
| C | -0.311937660 | 1.921105100 | 0.750992943 |
| C | -2.043440836 | 0.893248844 | 2.881785729 |
| H | -1.766817977 | -1.778824546 | 2.710401844 |
| H | -0.707524913 | -1.239173889 | 4.000305251 |
| H | -1.987010546 | 0.933483174 | 3.976493199 |
| H | -2.989289407 | 0.399066785 | 2.626198763 |
| H | -2.085800058 | 1.916547701 | 2.508482932 |
| O | -1.444204737 | 1.860364040 | 0.021968631 |
| O | 0.370365384 | 2.925252477 | 0.848048139 |
| C | -1.878808019 | 3.098250454 | -0.577783786 |
| H | -1.160489410 | 3.433819535 | -1.327561501 |
| H | -1.988637590 | 3.873263603 | 0.185100239 |
| H | -2.837964936 | 2.874214752 | -1.043783003 |
| P | 3.885387058 | -0.238249581 | 2.897551479 |
| C | 2.995334816 | -0.238300435 | 4.507571908 |
| C | 2.041793145 | 0.768966021 | 4.750594765 |
| C | 3.199072259 | -1.237896532 | 5.470540194 |
| C | 1.315346626 | 0.778819822 | 5.941065580 |
| C | 2.463084913 | -1.226762306 | 6.655713321 |
| C | 1.522059475 | -0.222089745 | 6.892850864 |
| H | 1.854888818 | 1.538441201 | 4.005734949 |
| H | 3.926871783 | -2.023941142 | 5.296389707 |
| H | 0.585410628 | 1.563258816 | 6.120061534 |
| H | 2.626980965 | -2.004088100 | 7.396514586 |
| H | 0.951122983 | -0.219566711 | 7.816982573 |
| C | 4.607162052 | -1.916781176 | 2.755321882 |
| C | 5.863272849 | -2.219275646 | 3.304559481 |
| C | 3.853804695 | -2.945477658 | 2.165156842 |
| C | 6.352804351 | -3.525208089 | 3.255784023 |
| C | 4.339383415 | -4.250562189 | 2.131256410 |
| C | 5.592708648 | -4.542435247 | 2.674628243 |
| H | 6.461079418 | -1.439909626 | 3.766077581 |
| H | 2.889559453 | -2.741911378 | 1.714890989 |
| H | 7.328140927 | -3.747387829 | 3.679305648 |
| H | 3.730554129 | -5.029062734 | 1.680690192 |
| H | 5.976926056 | -5.557940222 | 2.645274521 |
| C | 5.297160084 | 0.901618426 | 3.100999094 |
| C | 5.281535691 | 1.908562941 | 4.078016715 |
| C | 6.375085317 | 0.829377224 | 2.203835009 |
| C | 6.319749234 | 2.837028415 | 4.143087859 |
| C | 7.407364721 | 1.762425491 | 2.273222351 |
| C | 7.379904342 | 2.771332905 | 3.237770186 |
| H | 4.464121535 | 1.971654054 | 4.788985388 |
| H | 6.416533782 | 0.042026489 | 1.460856580 |
| H | 6.300245656 | 3.610735995 | 4.905268330 |
| H | 8.233265552 | 1.697247896 | 1.570815909 |
| H | 8.184804838 | 3.498754744 | 3.288799036 |

==============================

10

==============================

| | | | |
|---|---|---|---|
| C | 0.614980944 | -0.304813913 | -0.439140705 |
| C | 0.141545738 | -0.267720376 | 1.014534034 |
| Rh | 1.873947775 | -0.466473381 | 2.333438860 |
| C | 1.114204498 | -2.390085715 | 1.653145863 |

| | | | |
|---|---|---|---|
| C | 2.141713381 | -1.932762245 | 0.760937876 |
| C | 1.714749544 | -1.363828483 | -0.585646525 |
| C | 3.060077039 | -0.960808220 | -1.189109304 |
| C | 3.488960125 | -2.553712276 | 0.503767681 |
| O | 4.024105388 | -1.877448216 | -0.639681077 |
| H | -0.230716521 | -0.511320969 | -1.104051427 |
| H | 0.986748777 | 0.686417629 | -0.709247148 |
| H | 1.170299401 | -3.302319835 | 2.234016826 |
| H | 4.180698295 | -2.467080858 | 1.344819148 |
| H | 3.372752596 | -3.628033684 | 0.284181027 |
| H | 3.088987821 | -1.042088861 | -2.279616625 |
| H | 3.317554970 | 0.068088280 | -0.910923841 |
| H | 1.335283763 | -2.228884918 | -1.150577622 |
| C | 0.008000732 | -1.509607675 | 1.765516197 |
| C | -1.141435042 | -1.846000855 | 2.674939653 |
| C | -0.806880137 | 0.815743843 | 1.410033633 |
| P | 2.862717878 | 1.712724865 | 2.054636504 |
| H | -1.503517530 | -0.972043820 | 3.215086545 |
| H | -1.966322487 | -2.225498401 | 2.057501946 |
| H | -0.865347335 | -2.618779212 | 3.395122869 |
| C | 2.551521195 | 2.865352310 | 3.451015178 |
| C | 2.687158155 | 4.256995220 | 3.297211644 |
| H | 2.937385801 | 4.676865068 | 2.327740646 |
| C | 2.481745394 | 5.110039375 | 4.380400552 |
| H | 2.590028507 | 6.182460929 | 4.245827333 |
| C | 2.132650134 | 4.591482606 | 5.630321355 |
| H | 1.970788932 | 5.259199980 | 6.471256740 |
| C | 1.980955118 | 3.213711185 | 5.787797351 |
| H | 1.702338829 | 2.798197020 | 6.751688347 |
| C | 2.185065037 | 2.359179164 | 4.704735421 |
| H | 2.067586753 | 1.293418344 | 4.848998813 |
| C | 4.687296927 | 1.683661406 | 1.809254938 |
| C | 5.261728842 | 0.574179002 | 1.170524940 |
| H | 4.646540692 | -0.248881760 | 0.834779849 |
| C | 6.639611213 | 0.507460720 | 0.960396544 |
| H | 7.058665502 | -0.359720862 | 0.458402856 |
| C | 7.465264190 | 1.545550947 | 1.396626585 |
| H | 8.539150297 | 1.493640644 | 1.243665008 |
| C | 6.899078460 | 2.657136116 | 2.026624879 |
| H | 7.533008802 | 3.471845195 | 2.365340493 |
| C | 5.520390746 | 2.730581967 | 2.230071609 |
| H | 5.105189509 | 3.592473816 | 2.740332225 |
| C | 2.291662225 | 2.678105864 | 0.596364146 |
| C | 3.055989436 | 2.749904719 | -0.577943650 |
| H | 4.040007020 | 2.294660528 | -0.618955812 |
| C | 2.568789241 | 3.427342532 | -1.696973753 |
| H | 3.171039896 | 3.474806496 | -2.599718312 |
| C | 1.322468787 | 4.051687766 | -1.652103765 |
| H | 0.946395085 | 4.582227721 | -2.521936188 |
| C | 0.567081360 | 4.003931312 | -0.478623462 |
| H | -0.395957384 | 4.503665787 | -0.433207911 |
| C | 1.042983372 | 3.319265364 | 0.637433107 |
| H | 0.452528626 | 3.293025436 | 1.548159166 |
| O | -1.461719442 | 1.328581703 | 0.355750259 |
| O | -0.978720380 | 1.199808293 | 2.557778064 |
| C | -2.431719245 | 2.353246726 | 0.652187905 |
| H | -3.222599532 | 1.951992921 | 1.290582194 |
| H | -2.832812144 | 2.662599074 | -0.312278332 |
| H | -1.959683953 | 3.194416942 | 1.164163306 |
| P | 3.181828040 | -1.369917862 | 4.192269004 |
| C | 1.839023766 | -1.852337894 | 5.353930841 |
| C | 0.669328276 | -1.072711222 | 5.378777608 |
| C | 1.974419372 | -2.903549343 | 6.274245555 |
| C | -0.344917695 | -1.342935794 | 6.296412803 |
| C | 0.953890125 | -3.176766110 | 7.185412205 |
| C | -0.205299891 | -2.397780110 | 7.199740776 |
| H | 0.528294791 | -0.254470126 | 4.675518967 |
| H | 2.876763141 | -3.506814190 | 6.282960963 |
| H | -1.243269769 | -0.732435519 | 6.299160494 |
| H | 1.068771426 | -3.995075414 | 7.890339383 |
| H | -0.995176746 | -2.611281841 | 7.913950575 |
| C | 4.138665850 | -2.912188062 | 3.893649383 |
| C | 5.536647264 | -2.895039552 | 3.774033620 |
| C | 3.457682494 | -4.123079812 | 3.671553150 |
| C | 6.230307721 | -4.053734811 | 3.419228829 |
| C | 4.154459627 | -5.278332948 | 3.320254317 |
| C | 5.544321721 | -5.245278239 | 3.182245360 |
| H | 6.093974292 | -1.985298776 | 3.965370655 |
| H | 2.380741427 | -4.171046767 | 3.796077961 |
| H | 7.312665504 | -4.022078882 | 3.334687459 |
| H | 3.611416686 | -6.205150309 | 3.159143315 |
| H | 6.087583268 | -6.143397009 | 2.904637396 |
| C | 4.267060768 | -0.312770979 | 5.240946410 |
| C | 3.986432610 | -0.102685532 | 6.601232344 |
| C | 5.359398706 | 0.356568212 | 4.665549407 |
| C | 4.780546373 | 0.755970421 | 7.360260445 |
| C | 6.151209361 | 1.213365399 | 5.428676951 |
| C | 5.863632881 | 1.414945804 | 6.778702446 |
| H | 3.147706328 | -0.599785342 | 7.074509363 |
| H | 5.595611654 | 0.220437156 | 3.618749849 |
| H | 4.548340897 | 0.909828789 | 8.410237117 |
| H | 6.984166118 | 1.726150940 | 4.957599579 |
| H | 6.475414324 | 2.087865941 | 7.372557513 |

==============================

12

==============================

| | | | |
|---|---|---|---|
| C | -0.305607766 | 0.006773210 | 0.728868002 |
| C | -0.123874996 | 0.572005608 | 2.135584801 |
| Rh | 2.057355492 | -0.549874004 | 2.646021186 |
| C | 0.283674968 | -1.675436651 | 3.127399765 |
| C | 0.531460410 | -2.124133507 | 1.832811581 |
| C | -0.219499320 | -1.515503137 | 0.670442579 |
| C | 0.420848785 | -2.236992924 | -0.516870626 |
| C | 1.025449812 | -3.468205488 | 1.348044145 |
| O | 0.627641551 | -3.562274920 | -0.019983944 |
| H | -1.284291468 | 0.341719629 | 0.359311377 |
| H | 0.432764737 | 0.471296533 | 0.069696716 |
| H | 0.325308954 | -2.318667351 | 3.994209655 |
| H | 2.115384014 | -3.527282252 | 1.431063346 |
| H | 0.589566358 | -4.308006472 | 1.903353165 |
| H | -0.220560851 | -2.306254123 | -1.398582308 |
| H | 1.369236230 | -1.761447125 | -0.799145194 |
| H | -1.231954830 | -1.937384793 | 0.776260021 |
| C | -0.074762702 | -0.263932346 | 3.262693209 |
| C | -0.249992552 | 0.231287248 | 4.674433572 |
| C | -0.382102710 | 2.055017311 | 2.203427501 |
| H | -0.227356594 | 2.481752277 | 3.189228165 |
| H | 0.257489413 | 2.576454399 | 1.485316502 |
| H | -1.418566144 | 2.247537348 | 1.895249811 |
| P | 3.476885589 | 1.275165111 | 1.872976682 |
| C | 3.288656613 | 2.882186651 | 2.728449478 |
| C | 3.993664301 | 4.016102642 | 2.285157789 |

| | | | |
|---|---|---|---|
| H | 4.667293185 | 3.941329037 | 1.436092940 |
| C | 3.836885819 | 5.236944976 | 2.937521006 |
| H | 4.380809776 | 6.108427841 | 2.584601284 |
| C | 2.988214770 | 5.339626939 | 4.045136719 |
| H | 2.871450980 | 6.292546682 | 4.552997867 |
| C | 2.298666976 | 4.214292444 | 4.496410121 |
| H | 1.637741550 | 4.280129817 | 5.355860542 |
| C | 2.448413293 | 2.989969821 | 3.841445846 |
| H | 1.894111746 | 2.130617735 | 4.197336929 |
| C | 5.312996393 | 1.225701716 | 1.641719551 |
| C | 5.924714755 | 0.956036907 | 0.407578713 |
| H | 5.329833829 | 0.789986175 | -0.481839875 |
| C | 7.315877635 | 0.909222554 | 0.299534930 |
| H | 7.767396077 | 0.704656941 | -0.667251741 |
| C | 8.120598459 | 1.130387494 | 1.417842108 |
| H | 9.202344906 | 1.090261788 | 1.331399033 |
| C | 7.520525711 | 1.425385168 | 2.644561773 |
| H | 8.130029435 | 1.614313361 | 3.523021247 |
| C | 6.132203121 | 1.478598568 | 2.755872713 |
| H | 5.691297465 | 1.720703803 | 3.715246833 |
| C | 2.859638872 | 1.508721320 | 0.160525683 |
| C | 2.283678537 | 2.700241392 | -0.300352748 |
| H | 2.206628731 | 3.560164365 | 0.356721987 |
| C | 1.808550757 | 2.789635402 | -1.611359032 |
| H | 1.358410309 | 3.716421399 | -1.955333482 |
| C | 1.917042297 | 1.701905846 | -2.478007481 |
| H | 1.553185692 | 1.779270697 | -3.498308106 |
| C | 2.493426290 | 0.511372346 | -2.028056089 |
| H | 2.578038351 | -0.341598984 | -2.695377688 |
| C | 2.949841182 | 0.410413944 | -0.714921255 |
| H | 3.372972340 | -0.527010480 | -0.361116916 |
| O | 0.051116121 | 1.326888551 | 5.108025688 |
| O | -0.779099349 | -0.739294663 | 5.441554080 |
| C | -0.952577907 | -0.407219292 | 6.837922152 |
| H | -1.390696575 | -1.294601135 | 7.292979658 |
| H | 0.013712979 | -0.172267628 | 7.292210742 |
| H | -1.616792452 | 0.453044548 | 6.944511024 |
| P | 3.734986362 | -1.893323848 | 3.557894363 |
| C | 3.142647251 | -3.117315001 | 4.813654162 |
| C | 2.117878910 | -2.710003068 | 5.684551233 |
| C | 3.781148295 | -4.342349559 | 5.058883932 |
| C | 1.706050982 | -3.524563398 | 6.736780186 |
| C | 3.362194946 | -5.160090942 | 6.111194298 |
| C | 2.318847809 | -4.761930824 | 6.947042737 |
| H | 1.649041191 | -1.741073516 | 5.554231817 |
| H | 4.616447667 | -4.662413740 | 4.446638588 |
| H | 0.909137217 | -3.189003565 | 7.394454926 |
| H | 3.863873147 | -6.108522869 | 6.280199448 |
| H | 1.997445286 | -5.401026392 | 7.763761051 |
| C | 4.799909879 | -0.869086072 | 4.654706923 |
| C | 4.190903708 | 0.205089465 | 5.323742187 |
| C | 6.139001711 | -1.173690840 | 4.931903332 |
| C | 4.918464421 | 0.986680193 | 6.220263271 |
| C | 6.866520942 | -0.384929335 | 5.824247697 |
| C | 6.264736861 | 0.702145818 | 6.462290604 |
| H | 3.147939244 | 0.442866297 | 5.133962770 |
| H | 6.622700022 | -2.014621407 | 4.445853809 |
| H | 4.436212542 | 1.824775017 | 6.714877972 |
| H | 7.908556969 | -0.621666159 | 6.019497445 |
| H | 6.837385717 | 1.315559909 | 7.151481774 |
| C | 4.823792404 | -2.801448574 | 2.392110537 |
| C | 4.482429206 | -4.086633577 | 1.929643485 |
| C | 5.928273416 | -2.153901551 | 1.812891356 |
| C | 5.222930109 | -4.700578243 | 0.919140821 |
| C | 6.663984758 | -2.772095336 | 0.802115415 |
| C | 6.314324337 | -4.044405438 | 0.347350623 |
| H | 3.648360720 | -4.625554898 | 2.365340998 |
| H | 6.223338060 | -1.168799397 | 2.147386256 |
| H | 4.943631238 | -5.694451354 | 0.581969879 |
| H | 7.511961685 | -2.247273811 | 0.372535059 |
| H | 6.888255417 | -4.522756009 | -0.440471469 |

==============================

13

==============================

| | | | |
|---|---|---|---|
| C | 1.488389845 | 5.497343958 | 10.780622588 |
| C | 1.134248994 | 6.830348934 | 10.523618665 |
| H | 1.111294248 | 7.552952380 | 11.331397643 |
| C | 0.805807603 | 7.262623434 | 9.234529653 |
| C | 0.479288971 | 8.715463338 | 8.979389770 |
| C | 0.804262445 | 6.324435214 | 8.198427901 |
| H | 0.537685674 | 6.644723567 | 7.193093211 |
| C | 1.130696607 | 4.981708061 | 8.422233107 |
| C | 1.123654037 | 3.992185478 | 7.280025478 |
| C | 1.471196537 | 4.579547646 | 9.717502124 |
| H | 1.724259324 | 3.539919648 | 9.888638651 |
| C | 2.062671119 | 3.138163644 | 12.312480696 |
| C | 3.183513973 | 2.521660754 | 11.752691836 |
| H | 4.049421951 | 3.114729606 | 11.472191863 |
| C | 3.211789407 | 1.132816208 | 11.558227066 |
| C | 4.449412683 | 0.475816096 | 10.996460399 |
| C | 2.085080296 | 0.385013831 | 11.914896730 |
| H | 2.094987391 | -0.692192106 | 11.760900165 |
| C | 0.938908470 | 0.984808636 | 12.457543228 |
| C | -0.269945322 | 0.150792053 | 12.810649470 |
| C | 0.939815464 | 2.367337782 | 12.655532865 |
| H | 0.059636333 | 2.850425212 | 13.076494899 |
| C | 3.549694922 | 5.724025593 | 12.910704962 |
| C | 3.814618707 | 7.003470473 | 12.341581682 |
| H | 3.225229486 | 7.347566922 | 11.502998411 |
| C | 4.826606269 | 7.803036966 | 12.806704924 |
| H | 5.017160725 | 8.766549964 | 12.340625494 |
| C | 5.637610814 | 7.388795707 | 13.890104593 |
| C | 6.667717695 | 8.216106263 | 14.409159774 |
| H | 6.825263119 | 9.194676691 | 13.962772299 |
| C | 7.457588327 | 7.783964414 | 15.449448429 |
| H | 8.247247633 | 8.420352161 | 15.838230687 |
| C | 7.243751659 | 6.503638805 | 16.011463968 |
| H | 7.867104728 | 6.168450112 | 16.835708686 |
| C | 6.256683311 | 5.677751442 | 15.525320673 |
| H | 6.101873197 | 4.704909930 | 15.975383415 |
| C | 5.421209059 | 6.088435624 | 14.448577431 |
| C | 4.382703204 | 5.242777807 | 13.922281863 |
| C | 4.321650972 | 3.834846653 | 14.435333592 |
| C | 5.393315302 | 2.953900019 | 14.050988823 |
| C | 6.413615950 | 3.346096541 | 13.136686800 |
| H | 6.382953977 | 4.335515743 | 12.696181279 |
| C | 7.438165019 | 2.489500509 | 12.802993226 |
| H | 8.204103670 | 2.813510015 | 12.104248527 |
| C | 7.506033696 | 1.191766042 | 13.363249434 |
| H | 8.326208292 | 0.530264477 | 13.099698664 |
| C | 6.526128582 | 0.770424892 | 14.231328347 |
| H | 6.558316100 | -0.227738404 | 14.659274588 |

| | | | |
|---|---|---|---|
| C | 5.451277510 | 1.627482766 | 14.586657746 |
| C | 4.432325971 | 1.208456496 | 15.473074773 |
| H | 4.477217523 | 0.208242374 | 15.896570120 |
| C | 3.400207262 | 2.047580275 | 15.804658125 |
| H | 2.646138157 | 1.693764053 | 16.493694140 |
| C | 3.321200761 | 3.369965907 | 15.284166715 |
| C | 0.958906397 | 3.432688303 | 16.957685488 |
| C | 0.757242484 | 3.781521646 | 18.293300811 |
| H | 1.228041323 | 4.668872129 | 18.705768194 |
| C | -0.052757825 | 2.989115366 | 19.126503643 |
| C | -0.248042526 | 3.378693985 | 20.572873205 |
| C | -0.651015662 | 1.849701509 | 18.590436265 |
| H | -1.278033849 | 1.229553605 | 19.227556627 |
| C | -0.473138456 | 1.480256414 | 17.246176936 |
| C | -1.127293520 | 0.230940670 | 16.706042743 |
| C | 0.323929860 | 2.286731626 | 16.436790683 |
| H | 0.460438093 | 2.023705770 | 15.390555787 |
| C | 2.771868246 | 5.752933107 | 16.757593648 |
| C | 3.624576605 | 5.382560959 | 17.808415025 |
| H | 3.701612303 | 4.338705615 | 18.102541250 |
| C | 4.400735276 | 6.339944185 | 18.465184153 |
| C | 5.367473610 | 5.944045984 | 19.555137616 |
| C | 4.323185724 | 7.670384950 | 18.034336698 |
| H | 4.947592880 | 8.417542046 | 18.520852758 |
| C | 3.498268251 | 8.062574344 | 16.976075735 |
| C | 3.526092794 | 9.484528778 | 16.470516421 |
| C | 2.707418106 | 7.090436715 | 16.352513153 |
| H | 2.073144453 | 7.366454679 | 15.512542250 |
| P | 1.917422165 | 4.951383703 | 12.488656729 |
| P | 1.910051835 | 4.469828380 | 15.782784972 |
| Rh | 0.399839442 | 5.468408864 | 14.198995242 |
| C | -0.703040327 | 6.987378415 | 13.048494440 |
| C | -1.238824909 | 5.915967842 | 12.739819063 |
| H | -0.470467737 | 8.032608517 | 13.088388597 |
| C | -2.150926556 | 4.820886869 | 12.368646295 |
| O | -2.535214760 | 4.040139928 | 13.497785609 |
| H | -3.038996150 | 5.252902560 | 11.879273713 |
| H | -1.663965315 | 4.142023861 | 11.660708579 |
| C | -1.008551695 | 6.153973316 | 15.998232034 |
| C | -1.516288569 | 4.973953054 | 15.517600217 |
| H | -0.321848123 | 6.169270506 | 16.838961429 |
| H | -1.447039804 | 7.107927544 | 15.716045952 |
| H | -1.175570334 | 4.039682142 | 15.949286102 |
| C | -2.765877119 | 4.824304786 | 14.673875072 |
| H | -3.501249665 | 4.264925812 | 15.263533214 |
| H | -3.208771488 | 5.796974276 | 14.424070782 |
| H | 2.667519654 | 9.703292974 | 15.829021870 |
| H | 3.531862264 | 10.206353898 | 17.293535292 |
| H | 4.435440251 | 9.651657378 | 15.879708090 |
| H | 5.392017003 | 6.684743499 | 20.360635702 |
| H | 5.114095006 | 4.973433465 | 19.990802544 |
| H | 6.385162230 | 5.870950349 | 19.150672466 |
| H | -1.088044359 | 0.194715071 | 15.614668574 |
| H | -0.627117862 | -0.666945009 | 17.088783799 |
| H | -2.177080926 | 0.168881300 | 17.011481644 |
| H | -0.972738846 | 2.730216244 | 21.072216565 |
| H | 0.696320648 | 3.314491422 | 21.126205770 |
| H | -0.602118053 | 4.412034198 | 20.659707316 |
| H | 4.249421053 | -0.547129019 | 10.665168119 |
| H | 4.846820301 | 1.038502023 | 10.145066290 |
| H | 5.239197379 | 0.438874238 | 11.754894503 |
| H | -1.077536087 | 0.767582534 | 13.216165105 |
| H | -0.659146061 | -0.372476034 | 11.929921337 |
| H | -0.018088480 | -0.616434203 | 13.552192999 |
| H | 1.308017048 | 2.972012529 | 7.628187225 |
| H | 0.162599614 | 4.001479907 | 6.753912188 |
| H | 1.896466915 | 4.239092243 | 6.542706713 |
| H | -0.090465836 | 8.843468076 | 8.055043768 |
| H | -0.105747606 | 9.144649730 | 9.799795179 |
| H | 1.395389325 | 9.311703923 | 8.885832278 |

==============================

13-TS

==============================

| | | | |
|---|---|---|---|
| C | 1.449165290 | 5.613263163 | 11.105344953 |
| C | 0.795334699 | 6.823960656 | 11.397250039 |
| H | 0.713641934 | 7.157061495 | 12.429201797 |
| C | 0.244694080 | 7.604667439 | 10.379146130 |
| C | -0.436899995 | 8.917606064 | 10.684348331 |
| C | 0.349331313 | 7.147682950 | 9.058097680 |
| H | -0.080669989 | 7.746002879 | 8.256924351 |
| C | 0.983956810 | 5.943752784 | 8.740595596 |
| C | 1.054216411 | 5.464821674 | 7.309421332 |
| C | 1.534637036 | 5.179259481 | 9.778755978 |
| H | 2.019586353 | 4.238813433 | 9.539721632 |
| C | 2.686247860 | 3.042169838 | 11.864533875 |
| C | 3.768281615 | 3.027477344 | 10.970702068 |
| H | 4.214553969 | 3.962655407 | 10.641743506 |
| C | 4.297631668 | 1.818186777 | 10.514501306 |
| C | 5.487364605 | 1.791325445 | 9.586937623 |
| C | 3.739246085 | 0.624898196 | 10.990865960 |
| H | 4.161167875 | -0.321450726 | 10.658687999 |
| C | 2.673923941 | 0.613050491 | 11.895647382 |
| C | 2.153913161 | -0.684432745 | 12.464205117 |
| C | 2.143969592 | 1.836930603 | 12.319523436 |
| H | 1.335881948 | 1.851507382 | 13.044326400 |
| C | 3.595199914 | 5.498073152 | 13.108517775 |
| C | 3.859570344 | 6.818120526 | 12.655386994 |
| H | 3.230793515 | 7.261024722 | 11.892915145 |
| C | 4.912611534 | 7.539008013 | 13.160545811 |
| H | 5.110511994 | 8.543716659 | 12.795195271 |
| C | 5.760925744 | 6.988848696 | 14.150551138 |
| C | 6.847588562 | 7.729030736 | 14.685810802 |
| H | 7.013487111 | 8.742079379 | 14.328547834 |
| C | 7.680034389 | 7.173500401 | 15.628482277 |
| H | 8.514582779 | 7.743920611 | 16.025673349 |
| C | 7.450724306 | 5.852248135 | 16.081570964 |
| H | 8.113257130 | 5.416472708 | 16.824104723 |
| C | 6.395319438 | 5.115799656 | 15.594377271 |
| H | 6.227092755 | 4.110814697 | 15.962464323 |
| C | 5.520588704 | 5.653167490 | 14.608801021 |
| C | 4.421378164 | 4.909308865 | 14.066533544 |
| C | 4.213244935 | 3.497998836 | 14.535401849 |
| C | 5.147466467 | 2.494178142 | 14.114902487 |
| C | 6.243537999 | 2.784088359 | 13.254725490 |
| H | 6.377618279 | 3.794341554 | 12.888865851 |
| C | 7.136238856 | 1.803231089 | 12.888629095 |
| H | 7.975429010 | 2.052281567 | 12.245211035 |
| C | 6.971417829 | 0.472594062 | 13.341491757 |
| H | 7.682667258 | -0.291937605 | 13.043016856 |
| C | 5.915598924 | 0.153154844 | 14.162405983 |
| H | 5.781422952 | -0.864168246 | 14.519544313 |
| C | 4.987735747 | 1.145839738 | 14.573063034 |

| | | | |
|---|---|---|---|
| C | 3.914927338 | 0.840907285 | 15.444258694 |
| H | 3.806646525 | -0.176138218 | 15.811385048 |
| C | 3.025617079 | 1.810566271 | 15.835910062 |
| H | 2.218650653 | 1.549672167 | 16.509641358 |
| C | 3.158771540 | 3.149418310 | 15.380279215 |
| C | 0.835670741 | 3.614236767 | 17.126858858 |
| C | 0.862950131 | 4.043667223 | 18.456110099 |
| H | 1.541285110 | 4.832563168 | 18.764719914 |
| C | 0.000992253 | 3.476539178 | 19.408520644 |
| C | 0.055900012 | 3.937717224 | 20.845947764 |
| C | -0.885112941 | 2.477468563 | 19.001440065 |
| H | -1.561532537 | 2.039145295 | 19.732435578 |
| C | -0.936210888 | 2.028409500 | 17.673210559 |
| C | -1.895386645 | 0.934532522 | 17.268219844 |
| C | -0.076216337 | 2.612514742 | 16.742832040 |
| H | -0.117717689 | 2.290681747 | 15.704625931 |
| C | 2.746013662 | 5.787044693 | 16.535156686 |
| C | 3.690098654 | 5.571606223 | 17.551328949 |
| H | 3.866821747 | 4.566533315 | 17.926267940 |
| C | 4.427372430 | 6.637350637 | 18.071551339 |
| C | 5.473403483 | 6.418741612 | 19.136648243 |
| C | 4.216884090 | 7.917136288 | 17.539988291 |
| H | 4.802532748 | 8.748698093 | 17.926848849 |
| C | 3.296132106 | 8.153505551 | 16.515996471 |
| C | 3.152884797 | 9.523150242 | 15.898308131 |
| C | 2.553256431 | 7.073657805 | 16.026051400 |
| H | 1.852415286 | 7.225911087 | 15.211630726 |
| P | 2.076706586 | 4.630855833 | 12.518724652 |
| P | 1.850050830 | 4.379711972 | 15.802320025 |
| Rh | 0.390186123 | 4.685481299 | 14.060477982 |
| C | -0.732791495 | 3.612636238 | 12.751868029 |
| C | -1.752999368 | 4.224226170 | 13.260553107 |
| H | -0.541728813 | 3.016222965 | 11.871279271 |
| C | -3.155456302 | 4.550862291 | 12.810369708 |
| O | -3.889161730 | 5.135557123 | 13.860709751 |
| H | -3.087066692 | 5.239881953 | 11.951743660 |
| H | -3.672876460 | 3.641895436 | 12.490458457 |
| C | -0.616305276 | 6.017598253 | 15.390099410 |
| C | -1.773018526 | 5.226070655 | 14.992987676 |
| H | -0.312563940 | 5.937021926 | 16.429447879 |
| H | -0.566451896 | 7.031985251 | 14.993220307 |
| H | -2.011685185 | 4.401613685 | 15.664823320 |
| C | -3.013590496 | 5.998894677 | 14.547374723 |
| H | -3.543656871 | 6.367286512 | 15.430296297 |
| H | -2.715709045 | 6.860229417 | 13.924805242 |
| H | -1.397242998 | 8.996344234 | 10.164183183 |
| H | -0.621067368 | 9.037061503 | 11.756163620 |
| H | 0.177588691 | 9.764047897 | 10.355521662 |
| H | 1.748301062 | 4.627249536 | 7.198770179 |
| H | 0.070527040 | 5.129057707 | 6.960575498 |
| H | 1.378817289 | 6.266013376 | 6.637579246 |
| H | 5.432282654 | 0.953190408 | 8.885552031 |
| H | 5.574065163 | 2.716718342 | 9.010226837 |
| H | 6.410221743 | 1.673564475 | 10.167124212 |
| H | 1.098829879 | -0.609980764 | 12.744463860 |
| H | 2.263703664 | -1.510716819 | 11.756207194 |
| H | 2.714554980 | -0.946831063 | 13.369687525 |
| H | -1.994420175 | 0.867916836 | 16.180911286 |
| H | -1.550667955 | -0.040659703 | 17.632544771 |
| H | -2.891229739 | 1.102347525 | 17.691083959 |
| H | -0.860014444 | 3.683249958 | 21.386116920 |
| H | 0.893216186 | 3.465529735 | 21.374329622 |
| H | 0.200667649 | 5.020855545 | 20.911910069 |
| H | 2.139933614 | 9.691065838 | 15.519231921 |
| H | 3.385937661 | 10.317230803 | 16.613270251 |
| H | 3.840826872 | 9.627541360 | 15.049906003 |
| H | 5.438446116 | 7.203427299 | 19.899192272 |
| H | 5.350594586 | 5.452744049 | 19.634325039 |
| H | 6.474643056 | 6.439689872 | 18.689866234 |

==============================

14

==============================

| | | | |
|---|---|---|---|
| C | 1.544423783 | 4.986412988 | 10.631220841 |
| C | 0.504535120 | 5.792506571 | 10.175157769 |
| H | -0.150462409 | 6.280138534 | 10.887952297 |
| C | 0.311492142 | 5.987414462 | 8.796193292 |
| C | -0.825350413 | 6.859148530 | 8.315690636 |
| C | 1.184040184 | 5.364192690 | 7.905172982 |
| H | 1.044343335 | 5.511266736 | 6.836164219 |
| C | 2.240624292 | 4.547014975 | 8.343478744 |
| C | 3.164046252 | 3.894914213 | 7.341561889 |
| C | 2.407643054 | 4.358976652 | 9.712529102 |
| H | 3.210987658 | 3.718334870 | 10.066436178 |
| C | 1.670562309 | 2.815727248 | 12.391698413 |
| C | 2.718402722 | 1.905542290 | 12.245998056 |
| H | 3.746229256 | 2.244528657 | 12.223288954 |
| C | 2.451407231 | 0.534620731 | 12.122640458 |
| C | 3.593925519 | -0.441407547 | 11.976434679 |
| C | 1.119293870 | 0.109477626 | 12.117560793 |
| H | 0.903942346 | -0.952152308 | 12.016540063 |
| C | 0.049262056 | 1.010779748 | 12.220310056 |
| C | -1.380636387 | 0.531395163 | 12.140871615 |
| C | 0.339579118 | 2.367250850 | 12.368370256 |
| H | -0.476616205 | 3.079480743 | 12.451335626 |
| C | 3.522913121 | 5.339327860 | 12.745382485 |
| C | 3.728797315 | 6.580204124 | 12.061333798 |
| H | 3.054508322 | 6.857939996 | 11.259237908 |
| C | 4.753840925 | 7.421128629 | 12.402011760 |
| H | 4.895002017 | 8.354597732 | 11.864021478 |
| C | 5.635950121 | 7.092956019 | 13.459855263 |
| C | 6.687268960 | 7.967296653 | 13.839035284 |
| H | 6.802610414 | 8.905069725 | 13.302291741 |
| C | 7.544616225 | 7.635211236 | 14.861244608 |
| H | 8.347875936 | 8.309609640 | 15.142435008 |
| C | 7.382088295 | 6.407927241 | 15.546961753 |
| H | 8.064676477 | 6.146329925 | 16.350379488 |
| C | 6.372461150 | 5.538980645 | 15.202285840 |
| H | 6.272216921 | 4.602300366 | 15.736389843 |
| C | 5.462372321 | 5.847638086 | 14.149414808 |
| C | 4.394395232 | 4.958227903 | 13.775438067 |
| C | 4.362952552 | 3.626557025 | 14.467853993 |
| C | 5.400713483 | 2.692222289 | 14.118838553 |
| C | 6.305280650 | 2.928950282 | 13.044646677 |
| H | 6.228250648 | 3.848886659 | 12.476141406 |
| C | 7.268981128 | 2.003814758 | 12.711641175 |
| H | 7.943640940 | 2.203381766 | 11.884257671 |
| C | 7.392973085 | 0.797900255 | 13.440718570 |
| H | 8.163665361 | 0.081467541 | 13.172752388 |
| C | 6.535674729 | 0.536016127 | 14.484604688 |
| H | 6.618952606 | -0.388140210 | 15.050208397 |
| C | 5.520179832 | 1.461980120 | 14.841438085 |
| C | 4.616667002 | 1.199582150 | 15.900308009 |

| | | | |
|---|---|---|---|
| H | 4.723772357 | 0.281110319 | 16.470640893 |
| C | 3.612244230 | 2.082752567 | 16.198157213 |
| H | 2.920085920 | 1.852037683 | 16.998987954 |
| C | 3.457114492 | 3.299440469 | 15.473827184 |
| C | 0.840268810 | 3.223480045 | 16.679617262 |
| C | 0.337876308 | 3.439587764 | 17.963383699 |
| H | 0.669072900 | 4.295581103 | 18.544789470 |
| C | -0.584799381 | 2.541242863 | 18.526746536 |
| C | -1.110944619 | 2.779432737 | 19.923035645 |
| C | -0.978527401 | 1.430714626 | 17.779298200 |
| H | -1.681106380 | 0.722462775 | 18.213501779 |
| C | -0.483356838 | 1.191375468 | 16.487200813 |
| C | -0.923623054 | -0.026219073 | 15.709291083 |
| C | 0.418702575 | 2.102551720 | 15.941753813 |
| H | 0.820334560 | 1.923626397 | 14.948887014 |
| C | 2.559830228 | 5.574125082 | 17.115616606 |
| C | 3.832230865 | 5.548954232 | 17.687812827 |
| H | 4.521973883 | 4.746717118 | 17.445486503 |
| C | 4.236268036 | 6.561956487 | 18.570147891 |
| C | 5.637316373 | 6.556077478 | 19.132621490 |
| C | 3.331555619 | 7.579959920 | 18.879293539 |
| H | 3.636956898 | 8.367833800 | 19.564889319 |
| C | 2.041289980 | 7.624170413 | 18.329345173 |
| C | 1.092004287 | 8.740381430 | 18.695560448 |
| C | 1.671248424 | 6.617489208 | 17.437419879 |
| H | 0.673410524 | 6.638834536 | 17.001695791 |
| P | 1.859945351 | 4.627615974 | 12.397230540 |
| P | 2.006573037 | 4.369869564 | 15.855273159 |
| Rh | 1.010626571 | 5.767341941 | 14.110463857 |
| C | -0.900832603 | 5.062440543 | 14.422907946 |
| C | -1.855701034 | 5.741726259 | 13.458100279 |
| C | -1.274058861 | 7.073931947 | 13.056554443 |
| C | 0.059161314 | 7.236863693 | 13.092291991 |
| H | 0.552664677 | 8.156611999 | 12.765775715 |
| C | -2.433756002 | 8.020918738 | 12.818557081 |
| H | -2.003447826 | 5.124490500 | 12.558826197 |
| C | -3.242770448 | 6.188539472 | 13.970141486 |
| H | -0.922307145 | 3.972176808 | 14.431651501 |
| H | -1.040311797 | 5.434762392 | 15.450000812 |
| H | -2.376903746 | 8.894766295 | 13.487231408 |
| H | -2.491031649 | 8.387528294 | 11.786148817 |
| H | -4.017726575 | 5.420045375 | 13.915803752 |
| H | -3.166090520 | 6.544921934 | 15.012514139 |
| O | -3.608154919 | 7.245617859 | 13.084080025 |
| H | 0.140256677 | 8.654064630 | 18.163380676 |
| H | 0.877882029 | 8.737347736 | 19.770410090 |
| H | 1.524361631 | 9.718372068 | 18.456289675 |
| H | 5.740389723 | 7.244779765 | 19.975340906 |
| H | 5.926879189 | 5.555971139 | 19.472318998 |
| H | 6.356182448 | 6.858029395 | 18.361010195 |
| H | -1.795114585 | 1.986880727 | 20.237666416 |
| H | -0.291574128 | 2.824703719 | 20.649640634 |
| H | -1.648971301 | 3.732447538 | 19.985012308 |
| H | -0.260920832 | -0.216386460 | 14.860966117 |
| H | -0.934081696 | -0.918918970 | 16.343188672 |
| H | -1.939785737 | 0.103105535 | 15.317742406 |
| H | 3.253504547 | -1.476140763 | 12.067684761 |
| H | 4.074939393 | -0.333435374 | 10.997056766 |
| H | 4.364069538 | -0.263386469 | 12.733486489 |
| H | -2.058218479 | 1.199034368 | 12.681625115 |
| H | -1.716921782 | 0.493787853 | 11.097874587 |
| H | -1.491481832 | -0.474362404 | 12.556211988 |
| H | 3.907877244 | 3.259304208 | 7.830036550 |
| H | 2.601601250 | 3.274372227 | 6.634970716 |
| H | 3.699599780 | 4.648451661 | 6.752706258 |
| H | -0.830427878 | 6.954749959 | 7.226670413 |
| H | -1.793736727 | 6.446150410 | 8.621294554 |
| H | -0.756086024 | 7.866701831 | 8.741818144 |

==============================

15A

==============================

| | | | |
|---|---|---|---|
| C | 0.519968038 | -0.193796466 | -0.543269868 |
| C | 0.255356174 | -0.135681580 | 2.143967547 |
| C | 2.675163462 | 0.016242759 | 4.332216147 |
| C | 1.594297724 | 0.228848335 | 5.194408734 |
| H | 0.823121445 | 0.945659836 | 4.938228848 |
| C | 1.496110878 | -0.464968629 | 6.405119201 |
| C | 0.303439139 | -0.255239583 | 7.308896575 |
| C | 2.509686727 | -1.367990085 | 6.741401033 |
| H | 2.445929266 | -1.908522643 | 7.683827696 |
| C | 3.610416807 | -1.589024487 | 5.905030859 |
| C | 4.693349809 | -2.563612340 | 6.306495973 |
| C | 3.685255117 | -0.886045447 | 4.698997161 |
| H | 4.542720011 | -1.044310974 | 4.052607364 |
| C | 3.523727063 | 2.592788962 | 3.681365032 |
| C | 4.772950712 | 2.601145425 | 4.305093371 |
| H | 5.444744447 | 1.754608003 | 4.197451796 |
| C | 5.178895500 | 3.703881560 | 5.066445655 |
| C | 6.552256341 | 3.723026578 | 5.693144528 |
| C | 4.285794280 | 4.767842886 | 5.226441088 |
| H | 4.584744341 | 5.620597267 | 5.832752154 |
| C | 3.008940577 | 4.760667273 | 4.650470347 |
| C | 2.048375329 | 5.895024000 | 4.914183231 |
| C | 2.641644699 | 3.667012692 | 3.862044064 |
| H | 1.657232575 | 3.640202045 | 3.406278051 |
| C | 4.047701001 | 0.218344050 | 1.757925679 |
| C | 3.692288723 | -1.148838124 | 1.529632327 |
| H | 2.888116812 | -1.597596184 | 2.099309583 |
| C | 4.345854037 | -1.900322710 | 0.587936433 |
| H | 4.054438157 | -2.934153996 | 0.428577287 |
| C | 5.375215528 | -1.334755682 | -0.200550654 |
| C | 6.063828348 | -2.102555544 | -1.176265850 |
| H | 5.809611587 | -3.153689159 | -1.285279008 |
| C | 7.029735797 | -1.528209005 | -1.969005351 |
| H | 7.551647577 | -2.123844947 | -2.712346073 |
| C | 7.336821753 | -0.153437148 | -1.826529203 |
| H | 8.081412461 | 0.300029990 | -2.473999353 |
| C | 6.700346279 | 0.613268078 | -0.879576674 |
| H | 6.938763337 | 1.665565183 | -0.791482409 |
| C | 5.712388368 | 0.049476812 | -0.024296259 |
| C | 5.037205771 | 0.823921255 | 0.976922429 |
| C | 5.405938019 | 2.264970147 | 1.150396336 |
| C | 6.680126623 | 2.587654351 | 1.727606010 |
| C | 7.585686948 | 1.591256504 | 2.190553496 |
| H | 7.309710985 | 0.545541870 | 2.112681158 |
| C | 8.807421044 | 1.935427634 | 2.725217423 |
| H | 9.482730383 | 1.156728552 | 3.067713397 |
| C | 9.195463879 | 3.293285998 | 2.829127073 |
| H | 10.165686858 | 3.549326203 | 3.243916820 |
| C | 8.336530467 | 4.282745880 | 2.407368860 |
| H | 8.617301261 | 5.329639530 | 2.487910293 |
| C | 7.066300765 | 3.960853385 | 1.858520257 |

| | | | |
|---|---|---|---|
| C | 6.169152209 | 4.965658705 | 1.423343125 |
| H | 6.461183968 | 6.008621652 | 1.516632843 |
| C | 4.952559891 | 4.640383288 | 0.877819481 |
| H | 4.298399142 | 5.433904663 | 0.540268116 |
| C | 4.551590471 | 3.284315805 | 0.733619117 |
| C | 2.135169624 | 4.464578721 | -0.434114138 |
| C | 1.747965644 | 4.869287206 | -1.716431062 |
| H | 2.009324090 | 4.267758670 | -2.581018095 |
| C | 1.041505309 | 6.065220181 | -1.919014943 |
| C | 0.644462403 | 6.474709307 | -3.318615815 |
| C | 0.736833428 | 6.858618107 | -0.811892965 |
| H | 0.201189739 | 7.793928219 | -0.958421746 |
| C | 1.121965089 | 6.488788902 | 0.484778498 |
| C | 0.776923379 | 7.369598340 | 1.662027723 |
| C | 1.811489350 | 5.286193547 | 0.662247546 |
| H | 2.133748194 | 5.005249620 | 1.661004560 |
| C | 3.651866441 | 2.350017144 | -1.756194984 |
| C | 4.477184365 | 3.238757583 | -2.463076935 |
| H | 4.671020068 | 4.231318975 | -2.064395410 |
| C | 5.056359720 | 2.864914245 | -3.677684210 |
| C | 5.912286896 | 3.840118891 | -4.452336082 |
| C | 4.808587637 | 1.574556536 | -4.166910466 |
| H | 5.259919194 | 1.272308661 | -5.109809611 |
| C | 4.012502044 | 0.660836320 | -3.471983564 |
| C | 3.806673717 | -0.747187196 | -3.976592450 |
| C | 3.438980098 | 1.063501396 | -2.259415530 |
| H | 2.841203950 | 0.355611149 | -1.698675460 |
| P | 2.872642915 | 1.099240068 | 2.866790295 |
| P | 2.960347083 | 2.847243158 | -0.126222125 |
| Rh | 1.463948856 | 1.127230225 | 0.999979116 |
| C | -0.102623802 | 2.441218774 | 1.484348959 |
| C | -0.721425621 | 2.005517938 | 2.795435461 |
| C | -0.670057429 | 0.502445675 | 2.854440206 |
| H | 0.375112715 | -1.215255168 | 2.124614204 |
| C | -1.833001403 | 0.044805823 | 3.709623206 |
| H | -0.169225062 | 2.432456455 | 3.647525150 |
| C | -2.225614715 | 2.234977551 | 3.046617475 |
| H | 0.314383625 | 3.448378850 | 1.471651016 |
| H | -0.813625119 | 2.319499607 | 0.664304410 |
| H | -2.474564750 | -0.672499171 | 3.175016032 |
| H | -1.517616644 | -0.416111035 | 4.654539580 |
| H | -2.476603567 | 3.213575562 | 3.464888396 |
| H | -2.796124970 | 2.087852452 | 2.113144869 |
| O | -2.555002523 | 1.246152736 | 4.023770175 |
| C | 0.356770856 | 0.902179086 | -1.095096809 |
| C | 0.009836072 | 1.968402995 | -2.030516970 |
| C | 0.473297895 | -1.659530886 | -0.504596784 |
| H | -0.216465899 | 2.908504635 | -1.520843247 |
| H | 0.851396098 | 2.144412463 | -2.707995092 |
| H | -0.859335440 | 1.659747053 | -2.620660744 |
| O | 1.154100145 | -2.374988586 | 0.201504851 |
| O | -0.420216021 | -2.101128583 | -1.403629063 |
| C | -0.547940278 | -3.539684315 | -1.483461690 |
| H | -1.311725065 | -3.719350274 | -2.238822542 |
| H | 0.403617059 | -3.990032128 | -1.777000599 |
| H | -0.853792961 | -3.946200327 | -0.516333980 |
| H | 5.513004265 | -2.579348365 | 5.582414625 |
| H | 5.113097073 | -2.305711435 | 7.285363338 |
| H | 4.296637676 | -3.582393994 | 6.384720957 |
| H | 0.549245563 | -0.464956630 | 8.353961843 |
| H | -0.072684789 | 0.770595689 | 7.244766688 |

| | | | |
|---|---|---|---|
| H | -0.522142944 | -0.921317358 | 7.028000153 |
| H | 6.682388307 | 4.583818690 | 6.354937988 |
| H | 6.730474747 | 2.814492677 | 6.279066089 |
| H | 7.327424583 | 3.768129628 | 4.920952702 |
| H | 1.131740817 | 5.796156606 | 4.318702631 |
| H | 1.757408763 | 5.919960801 | 5.975480883 |
| H | 2.507072707 | 6.868389688 | 4.684656272 |
| H | 2.807596691 | -1.119131803 | -3.725884730 |
| H | 3.931507820 | -0.810741558 | -5.061387357 |
| H | 4.534614631 | -1.424216656 | -3.514673235 |
| H | 6.584270033 | 3.323282909 | -5.143506389 |
| H | 5.291178479 | 4.521374692 | -5.047210413 |
| H | 6.520460367 | 4.456694368 | -3.782716295 |
| H | 0.122125421 | 7.435043159 | -3.326226875 |
| H | 1.523642957 | 6.564831920 | -3.967055221 |
| H | -0.017054977 | 5.729637275 | -3.776117529 |
| H | 1.544616176 | 7.263948460 | 2.478762238 |
| H | 0.724417023 | 8.451231838 | 1.345626620 |
| H | -0.235258570 | 7.082793437 | 2.088261834 |

==============================

15B

==============================

| | | | |
|---|---|---|---|
| C | 0.828553357 | 0.557544975 | -1.204721043 |
| C | 0.359890398 | -0.373386448 | 1.938313809 |
| C | 2.390896592 | 0.345311828 | 4.566581726 |
| C | 1.122983414 | 0.295680553 | 5.147833175 |
| H | 0.257448482 | 0.640217589 | 4.600682297 |
| C | 0.957345620 | -0.195125299 | 6.451509156 |
| C | -0.423844958 | -0.281511259 | 7.058202575 |
| C | 2.085645844 | -0.621094224 | 7.156007927 |
| H | 1.966558970 | -0.995233023 | 8.170953767 |
| C | 3.371757612 | -0.572208237 | 6.597812249 |
| C | 4.571490474 | -1.029147887 | 7.394733771 |
| C | 3.511375462 | -0.084398191 | 5.299322083 |
| H | 4.503712741 | -0.031650536 | 4.858970827 |
| C | 3.375523100 | 2.753093044 | 3.585843965 |
| C | 4.706656500 | 2.958681625 | 3.963711897 |
| H | 5.454796781 | 2.195155345 | 3.784065300 |
| C | 5.092790671 | 4.160466222 | 4.564780359 |
| C | 6.539980688 | 4.388817478 | 4.933010473 |
| C | 4.109377116 | 5.124448477 | 4.833894516 |
| H | 4.400233980 | 6.054390586 | 5.317649898 |
| C | 2.763797895 | 4.920349046 | 4.506939955 |
| C | 1.701844330 | 5.947005289 | 4.818276984 |
| C | 2.411514105 | 3.732113554 | 3.855696460 |
| H | 1.377201379 | 3.577674318 | 3.557671897 |
| C | 3.921025954 | 0.052611924 | 2.114887019 |
| C | 3.658277585 | -1.345053383 | 2.294944039 |
| H | 2.976568012 | -1.651092285 | 3.079754880 |
| C | 4.250316454 | -2.293300235 | 1.503855480 |
| H | 4.057978335 | -3.348457314 | 1.679582524 |
| C | 5.106351747 | -1.912070552 | 0.436426109 |
| C | 5.678517921 | -2.872774885 | -0.436669838 |
| H | 5.452814324 | -3.923390102 | -0.274079777 |
| C | 6.503689870 | -2.486187742 | -1.469206256 |
| H | 6.937188764 | -3.229539514 | -2.131190340 |
| C | 6.787479899 | -1.114041020 | -1.666667162 |
| H | 7.433301629 | -0.809952641 | -2.485145172 |
| C | 6.252563627 | -0.158059156 | -0.831508126 |
| H | 6.470109809 | 0.888370384 | -1.006114043 |
| C | 5.397054123 | -0.521788141 | 0.246143843 |

| | | | |
|---|---|---|---|
| C | 4.824798042 | 0.464532475 | 1.129171919 |
| C | 5.379502559 | 1.854228380 | 1.034020327 |
| C | 6.738141023 | 2.015669037 | 1.491233924 |
| C | 7.434893092 | 0.988608556 | 2.189603837 |
| H | 6.947634789 | 0.037484940 | 2.369829899 |
| C | 8.716364991 | 1.191536483 | 2.652657638 |
| H | 9.225455527 | 0.398202783 | 3.191896871 |
| C | 9.377640400 | 2.421648271 | 2.431194638 |
| H | 10.390591458 | 2.564913841 | 2.795238334 |
| C | 8.734545443 | 3.434437860 | 1.756787178 |
| H | 9.231094068 | 4.385163644 | 1.581144808 |
| C | 7.408696319 | 3.263025238 | 1.282268007 |
| C | 6.732291427 | 4.296414538 | 0.585927371 |
| H | 7.259869352 | 5.221361925 | 0.368203454 |
| C | 5.431641445 | 4.134580993 | 0.192969166 |
| H | 4.933483889 | 4.934338556 | -0.342216932 |
| C | 4.715363879 | 2.932676835 | 0.467827194 |
| C | 2.282271265 | 4.545802353 | 0.276964489 |
| C | 1.103906691 | 4.916659047 | -0.386470125 |
| H | 0.625399550 | 4.226588738 | -1.072552516 |
| C | 0.519995819 | 6.171180242 | -0.171556243 |
| C | -0.747189709 | 6.546129676 | -0.904030543 |
| C | 1.145370178 | 7.058086603 | 0.708536612 |
| H | 0.709376819 | 8.041977765 | 0.871277435 |
| C | 2.321766664 | 6.714350475 | 1.385708994 |
| C | 3.018130760 | 7.720580995 | 2.271829262 |
| C | 2.866333185 | 5.441206380 | 1.187368106 |
| H | 3.752480800 | 5.164056127 | 1.745535759 |
| C | 3.213503753 | 2.779987878 | -1.864759769 |
| C | 3.187672416 | 3.905194566 | -2.697886254 |
| H | 2.904767215 | 4.874066003 | -2.298902849 |
| C | 3.528759201 | 3.795768548 | -4.050797837 |
| C | 3.475329490 | 5.005201557 | -4.955696566 |
| C | 3.891070496 | 2.539295698 | -4.553380982 |
| H | 4.154867051 | 2.447519848 | -5.605162455 |
| C | 3.918915298 | 1.399668173 | -3.742120738 |
| C | 4.243151911 | 0.037592863 | -4.305371497 |
| C | 3.588509814 | 1.537332289 | -2.390675922 |
| H | 3.614795243 | 0.661439803 | -1.749386836 |
| P | 2.722158362 | 1.168534322 | 2.962965775 |
| P | 2.940447232 | 2.856469586 | -0.048411149 |
| Rh | 1.576881153 | 1.001446861 | 0.944488199 |
| C | -0.128602252 | 2.208624713 | 1.227582114 |
| C | -0.956772137 | 1.644539586 | 2.361279709 |
| C | -0.750674564 | 0.155094440 | 2.445668236 |
| H | 0.591340132 | -1.436580353 | 2.004416229 |
| C | -1.997283283 | -0.431400914 | 3.076876309 |
| H | -0.655067362 | 2.104893976 | 3.317011892 |
| C | -2.495009786 | 1.695895954 | 2.283927767 |
| H | 0.257113338 | 3.211260724 | 1.408454959 |
| H | -0.636607837 | 2.172395003 | 0.265006046 |
| H | -2.425553192 | -1.235495160 | 2.459016363 |
| H | -1.822991930 | -0.832608747 | 4.084409738 |
| H | -2.940188829 | 2.636327846 | 2.619065556 |
| H | -2.832112442 | 1.486205418 | 1.254504201 |
| O | -2.908807410 | 0.670828804 | 3.191874111 |
| C | 1.047045920 | -0.567280418 | -0.744038808 |
| C | 1.223113995 | -2.005257357 | -0.546315884 |
| C | 0.364610079 | 1.468267816 | -2.249941712 |
| H | 2.120059384 | -2.218599784 | 0.041725984 |
| H | 0.357993626 | -2.436756881 | -0.035726080 |
| H | 1.325053642 | -2.476739412 | -1.529281777 |
| O | -0.197447682 | 2.529469098 | -2.059112498 |
| O | 0.625662988 | 0.952984230 | -3.458089729 |
| C | 0.261375563 | 1.796081504 | -4.576184354 |
| H | 0.562489073 | 1.239765810 | -5.463500438 |
| H | -0.815629452 | 1.981368247 | -4.577708849 |
| H | 0.800208236 | 2.744573917 | -4.512786664 |
| H | 5.504066892 | -0.878371784 | 6.843539567 |
| H | 4.647010451 | -0.479667758 | 8.339758529 |
| H | 4.497576532 | -2.093413648 | 7.645950305 |
| H | -0.392041112 | -0.181950265 | 8.147241879 |
| H | -1.085046603 | 0.495386556 | 6.661851041 |
| H | -0.888510448 | -1.249980171 | 6.833446828 |
| H | 6.729347207 | 5.435773029 | 5.185346032 |
| H | 6.823438161 | 3.782215119 | 5.801397124 |
| H | 7.205388372 | 4.108229551 | 4.109960570 |
| H | 1.119831251 | 6.185530033 | 3.921520921 |
| H | 1.002549497 | 5.568315132 | 5.572599264 |
| H | 2.134758454 | 6.875516421 | 5.199019514 |
| H | 3.319259111 | -0.529963197 | -4.473821767 |
| H | 4.770108714 | 0.107856235 | -5.261179349 |
| H | 4.860861584 | -0.542409378 | -3.612294925 |
| H | 4.209081567 | 4.934287734 | -5.764217084 |
| H | 2.486453541 | 5.101833318 | -5.421003266 |
| H | 3.665142502 | 5.929885521 | -4.402447618 |
| H | -1.208510651 | 7.442796013 | -0.478102595 |
| H | -0.542382492 | 6.748005347 | -1.963797538 |
| H | -1.479992824 | 5.731428274 | -0.868337215 |
| H | 3.690322315 | 7.231088512 | 2.980872090 |
| H | 3.617783455 | 8.413092184 | 1.668503016 |
| H | 2.301058211 | 8.323150951 | 2.837608416 |

==============================

15A-TS1

==============================

| | | | |
|---|---|---|---|
| C | 0.857199312 | 0.543455429 | -0.879661720 |
| C | 0.419049123 | -0.448733724 | 1.771340067 |
| C | 2.403631765 | 0.412152319 | 4.582879526 |
| C | 1.101389560 | 0.465331477 | 5.083789630 |
| H | 0.312815517 | 0.918249370 | 4.494995054 |
| C | 0.803516909 | -0.043736920 | 6.354984203 |
| C | -0.611486442 | -0.010672680 | 6.883807511 |
| C | 1.839823705 | -0.602488339 | 7.107887667 |
| H | 1.620624717 | -0.995525588 | 8.099039550 |
| C | 3.157653858 | -0.662903563 | 6.631035815 |
| C | 4.251168830 | -1.262664437 | 7.483759911 |
| C | 3.428448505 | -0.145558947 | 5.363363017 |
| H | 4.447140192 | -0.169585590 | 4.984391191 |
| C | 3.562633882 | 2.789067255 | 3.709879985 |
| C | 4.904818033 | 2.949890340 | 4.062475250 |
| H | 5.627775516 | 2.171107308 | 3.846213328 |
| C | 5.336618944 | 4.129745148 | 4.679313695 |
| C | 6.799229846 | 4.316827078 | 5.005666917 |
| C | 4.385619298 | 5.110830041 | 4.994359749 |
| H | 4.711806046 | 6.021354653 | 5.491730194 |
| C | 3.025948838 | 4.945568950 | 4.700433658 |
| C | 1.995882834 | 5.970904637 | 5.108839214 |
| C | 2.630689934 | 3.786000788 | 4.026845868 |
| H | 1.585482771 | 3.662253153 | 3.750934557 |
| C | 3.984701211 | 0.136861365 | 2.140325486 |
| C | 3.691149218 | -1.258345312 | 2.281569729 |
| H | 3.041288601 | -1.578289776 | 3.087799758 |

| | | | |
|---|---|---|---|
| C | 4.214849452 | -2.186980756 | 1.423188689 |
| H | 3.994637942 | -3.242738286 | 1.561593244 |
| C | 5.045097612 | -1.791373165 | 0.343058935 |
| C | 5.571164462 | -2.737802926 | -0.573028533 |
| H | 5.316032120 | -3.786459965 | -0.443095888 |
| C | 6.394148099 | -2.339474398 | -1.601807362 |
| H | 6.795839776 | -3.071995454 | -2.295683364 |
| C | 6.716521017 | -0.970733861 | -1.757272126 |
| H | 7.360518539 | -0.658746022 | -2.574487338 |
| C | 6.219658841 | -0.028567252 | -0.884383420 |
| H | 6.464515071 | 1.016410916 | -1.027326489 |
| C | 5.374000488 | -0.405853287 | 0.195645431 |
| C | 4.849001594 | 0.560934449 | 1.125857253 |
| C | 5.394713405 | 1.955663080 | 1.039418900 |
| C | 6.768960443 | 2.120262601 | 1.441900056 |
| C | 7.509398389 | 1.081089990 | 2.072287531 |
| H | 7.037994086 | 0.121288777 | 2.249122080 |
| C | 8.812074575 | 1.279391588 | 2.473249568 |
| H | 9.353545872 | 0.473664691 | 2.960543395 |
| C | 9.453054807 | 2.520934684 | 2.254791328 |
| H | 10.483204230 | 2.661351556 | 2.568766025 |
| C | 8.766545950 | 3.547940431 | 1.648133984 |
| H | 9.245056240 | 4.508747863 | 1.476857922 |
| C | 7.418219220 | 3.378352769 | 1.239289616 |
| C | 6.698883972 | 4.430241208 | 0.618323564 |
| H | 7.204401945 | 5.371021105 | 0.415826165 |
| C | 5.383616980 | 4.267456938 | 0.278835370 |
| H | 4.855691818 | 5.081232899 | -0.203259478 |
| C | 4.698400679 | 3.043152486 | 0.531052630 |
| C | 2.226980156 | 4.614153409 | 0.310486079 |
| C | 1.075416198 | 4.966308740 | -0.410550261 |
| H | 0.658286909 | 4.274451057 | -1.136384088 |
| C | 0.457050032 | 6.204862771 | -0.206960335 |
| C | -0.765244970 | 6.591207457 | -1.006183268 |
| C | 1.018861867 | 7.089904491 | 0.719328996 |
| H | 0.556022615 | 8.063934348 | 0.869310229 |
| C | 2.164781843 | 6.761651144 | 1.452506203 |
| C | 2.796387864 | 7.772562965 | 2.381137932 |
| C | 2.747846420 | 5.502808260 | 1.263204742 |
| H | 3.618304807 | 5.237748363 | 1.850696609 |
| C | 3.214232512 | 2.846667523 | -1.808169650 |
| C | 3.246528563 | 3.984492519 | -2.624277418 |
| H | 3.009633381 | 4.959939038 | -2.212466347 |
| C | 3.565670634 | 3.878337906 | -3.980399283 |
| C | 3.569393206 | 5.096008690 | -4.874161816 |
| C | 3.858731578 | 2.611481945 | -4.505543546 |
| H | 4.104718769 | 2.521504531 | -5.562089196 |
| C | 3.842109435 | 1.462601691 | -3.709594382 |
| C | 4.106088197 | 0.092948679 | -4.285331340 |
| C | 3.527347147 | 1.596565479 | -2.350932521 |
| H | 3.517920887 | 0.709662208 | -1.724159715 |
| P | 2.831623164 | 1.267106466 | 3.024525211 |
| P | 2.928894208 | 2.947178222 | 0.005277772 |
| Rh | 1.663300208 | 1.143671870 | 0.946069108 |
| C | -0.076500080 | 2.298809221 | 1.155974652 |
| C | -1.015227167 | 1.561134859 | 2.097123567 |
| C | -0.703498335 | 0.099345573 | 2.252650339 |
| H | 0.703096746 | -1.472040749 | 2.004288547 |
| C | -1.897071691 | -0.493723913 | 2.962300009 |
| H | -0.885730754 | 1.988329668 | 3.107259735 |
| C | -2.529643118 | 1.480341700 | 1.859595008 |
| H | 0.258697226 | 3.253495909 | 1.557934788 |
| H | -0.479654305 | 2.447093954 | 0.154100708 |
| H | -2.174630528 | -1.465820845 | 2.528854824 |
| H | -1.732455833 | -0.637355542 | 4.039879311 |
| H | -3.082231403 | 2.393852494 | 2.094808598 |
| H | -2.741790816 | 1.191075657 | 0.817001591 |
| O | -2.951797181 | 0.465990525 | 2.785701995 |
| C | 0.625516507 | -0.565766893 | -0.293239176 |
| C | 0.387821097 | -2.016107128 | -0.454096363 |
| C | 0.553771149 | 1.186754085 | -2.164849838 |
| H | 1.213606394 | -2.593403254 | -0.023005891 |
| H | -0.545752026 | -2.340606783 | 0.013028831 |
| H | 0.349674745 | -2.215657300 | -1.530375126 |
| O | 0.181503042 | 2.338189431 | -2.282436955 |
| O | 0.694397135 | 0.330892780 | -3.192352509 |
| C | 0.420895071 | 0.892852549 | -4.497515340 |
| H | 0.552581629 | 0.069748424 | -5.199088830 |
| H | -0.599182499 | 1.281096094 | -4.540277817 |
| H | 1.129029178 | 1.698346905 | -4.706312225 |
| H | 5.227499097 | -1.195632887 | 6.995534254 |
| H | 4.321526155 | -0.749419452 | 8.449426951 |
| H | 4.052076995 | -2.319734991 | 7.694528793 |
| H | -0.634023847 | 0.198900429 | 7.957935366 |
| H | -1.214003774 | 0.748627427 | 6.375635368 |
| H | -1.106434639 | -0.978318563 | 6.734803836 |
| H | 7.005305992 | 5.329193825 | 5.363371033 |
| H | 7.120287630 | 3.615766450 | 5.784870608 |
| H | 7.424403662 | 4.129931408 | 4.125938621 |
| H | 1.246423318 | 6.112379348 | 4.323043467 |
| H | 1.466703210 | 5.643513358 | 6.011715027 |
| H | 2.451249470 | 6.940355189 | 5.326513076 |
| H | 3.184087498 | -0.501539944 | -4.281197962 |
| H | 4.472301835 | 0.147103065 | -5.314342415 |
| H | 4.841402938 | -0.452077134 | -3.684363965 |
| H | 4.451387787 | 5.111211528 | -5.523023358 |
| H | 2.687680247 | 5.104408553 | -5.526335463 |
| H | 3.559345246 | 6.022239761 | -4.293011723 |
| H | -1.447279325 | 7.215471075 | -0.420245763 |
| H | -0.481875520 | 7.167277003 | -1.895524430 |
| H | -1.312162990 | 5.708866751 | -1.349822791 |
| H | 3.546470440 | 7.308315988 | 3.026610844 |
| H | 3.294668761 | 8.561742083 | 1.805081594 |
| H | 2.049560271 | 8.260320694 | 3.015537463 |

==============================

15A-TS2

==============================

| | | | |
|---|---|---|---|
| C | 0.205689580 | 1.040996453 | -0.362370768 |
| C | -0.223424589 | 0.697724007 | 2.598738743 |
| C | 2.845236666 | -0.195272670 | 4.373440059 |
| C | 1.753299481 | -0.044496249 | 5.234616146 |
| H | 0.956926109 | 0.651450245 | 4.987824039 |
| C | 1.687865927 | -0.758761777 | 6.438881494 |
| C | 0.491825542 | -0.608548069 | 7.349687561 |
| C | 2.742622766 | -1.615223671 | 6.764547152 |
| H | 2.706003365 | -2.167520614 | 7.701457885 |
| C | 3.856031073 | -1.773096705 | 5.927262675 |
| C | 4.988483141 | -2.690169323 | 6.326267448 |
| C | 3.897428669 | -1.053854375 | 4.731725403 |
| H | 4.763816421 | -1.157469463 | 4.085468220 |
| C | 3.748264389 | 2.385455666 | 3.788489157 |
| C | 5.026370751 | 2.329306290 | 4.348671699 |

| | | | |
|---|---|---|---|
| H | 5.646527603 | 1.448503260 | 4.208142339 |
| C | 5.532001426 | 3.413358379 | 5.073332692 |
| C | 6.943070646 | 3.380802659 | 5.607207669 |
| C | 4.712207729 | 4.532479652 | 5.259722989 |
| H | 5.093198781 | 5.375012678 | 5.833551998 |
| C | 3.412783428 | 4.596772170 | 4.743286724 |
| C | 2.535320882 | 5.794517938 | 5.018348664 |
| C | 2.942716962 | 3.512779310 | 3.995595486 |
| H | 1.935169960 | 3.542558967 | 3.584762588 |
| C | 4.159732486 | 0.121290180 | 1.778598223 |
| C | 3.866450485 | -1.259512220 | 1.556610296 |
| H | 3.161416516 | -1.763196498 | 2.209148885 |
| C | 4.475001767 | -1.963139484 | 0.550462148 |
| H | 4.258769412 | -3.019577158 | 0.412050898 |
| C | 5.382423150 | -1.323191718 | -0.331414009 |
| C | 5.990784872 | -2.023939601 | -1.404408451 |
| H | 5.773760417 | -3.082071775 | -1.525656211 |
| C | 6.831569982 | -1.375106793 | -2.279775950 |
| H | 7.287621969 | -1.917250921 | -3.102844587 |
| C | 7.095340644 | 0.004126993 | -2.113031409 |
| H | 7.741778206 | 0.516601611 | -2.819412052 |
| C | 6.541507645 | 0.703948756 | -1.065788483 |
| H | 6.746169663 | 1.762210541 | -0.958837738 |
| C | 5.674288303 | 0.065410104 | -0.136783163 |
| C | 5.070357518 | 0.780513612 | 0.957053833 |
| C | 5.478470473 | 2.212150691 | 1.157565937 |
| C | 6.803850427 | 2.471580972 | 1.647760197 |
| C | 7.684527284 | 1.429642772 | 2.054100835 |
| H | 7.349228257 | 0.399897815 | 1.997494708 |
| C | 8.952562758 | 1.708670850 | 2.513890612 |
| H | 9.605230100 | 0.895305053 | 2.817432607 |
| C | 9.417749251 | 3.043906413 | 2.590627353 |
| H | 10.423229887 | 3.248624113 | 2.945769504 |
| C | 8.589258125 | 4.076604013 | 2.214192136 |
| H | 8.929926427 | 5.107422543 | 2.269182667 |
| C | 7.272866716 | 3.821719172 | 1.744950566 |
| C | 6.409666378 | 4.871697448 | 1.351323178 |
| H | 6.767336748 | 5.897039431 | 1.402278212 |
| C | 5.140851635 | 4.609073957 | 0.901314615 |
| H | 4.511988507 | 5.434390568 | 0.593880083 |
| C | 4.650265638 | 3.276988662 | 0.811770773 |
| C | 2.186870511 | 4.607356679 | -0.084132223 |
| C | 1.536633958 | 4.926820309 | -1.279267128 |
| H | 1.562890951 | 4.242010878 | -2.118184404 |
| C | 0.805889793 | 6.116704364 | -1.404686689 |
| C | 0.098851529 | 6.422166222 | -2.703391156 |
| C | 0.759290480 | 6.992546601 | -0.318918937 |
| H | 0.207775505 | 7.925842790 | -0.411995967 |
| C | 1.406479916 | 6.702657599 | 0.890738216 |
| C | 1.336306201 | 7.673610618 | 2.047209249 |
| C | 2.098645110 | 5.494093431 | 1.006164855 |
| H | 2.595025996 | 5.257202968 | 1.943548825 |
| C | 3.362704242 | 2.359191249 | -1.535651864 |
| C | 4.044491774 | 3.207529243 | -2.424146261 |
| H | 4.285569963 | 4.224022702 | -2.124777266 |
| C | 4.403685101 | 2.764938125 | -3.696906320 |
| C | 5.103891066 | 3.680524825 | -4.673070701 |
| C | 4.080065077 | 1.449206461 | -4.065167501 |
| H | 4.354294110 | 1.098174653 | -5.058293029 |
| C | 3.429700905 | 0.577473103 | -3.191059362 |
| C | 3.120200027 | -0.850298638 | -3.571978224 |
| C | 3.081424577 | 1.047626380 | -1.916851431 |
| H | 2.598195977 | 0.375489256 | -1.220393745 |
| P | 3.003147923 | 0.945384723 | 2.948074016 |
| P | 2.949012772 | 2.953137523 | 0.148974190 |
| Rh | 1.236617667 | 1.569823060 | 1.442367836 |
| C | -0.477367025 | 2.865555833 | 0.863247110 |
| C | -1.714692737 | 2.373414456 | 1.563881930 |
| C | -1.446934695 | 1.220396220 | 2.490581876 |
| H | -0.040168733 | -0.222036641 | 3.151254961 |
| C | -2.808256904 | 0.696859958 | 2.883914191 |
| H | -2.083147810 | 3.221305875 | 2.170388009 |
| C | -2.925437414 | 1.807392291 | 0.812119808 |
| H | 0.095018897 | 3.577277406 | 1.473456786 |
| H | -0.595186362 | 3.269585983 | -0.138135016 |
| H | -2.819324815 | -0.399676166 | 2.939110583 |
| H | -3.169392387 | 1.093163151 | 3.842609998 |
| H | -3.562041308 | 2.565262169 | 0.346828384 |
| H | -2.602658206 | 1.089196792 | 0.042205093 |
| O | -3.688872624 | 1.163965068 | 1.842489855 |
| C | 0.505587844 | -0.003658881 | 0.321416411 |
| C | 0.509836325 | -1.472210101 | 0.418062339 |
| C | -0.076166922 | 1.435421991 | -1.745396110 |
| H | 1.536591898 | -1.845270218 | 0.347580774 |
| H | 0.089629055 | -1.808535546 | 1.370474722 |
| H | -0.082143210 | -1.888257404 | -0.403664539 |
| O | -0.268240333 | 2.563694888 | -2.157388918 |
| O | -0.066838975 | 0.338074416 | -2.529386195 |
| C | -0.246390346 | 0.594951497 | -3.940670852 |
| H | -0.269506694 | -0.387149176 | -4.411419269 |
| H | -1.179763120 | 1.134013625 | -4.117043354 |
| H | 0.593611630 | 1.184788334 | -4.316812936 |
| H | 5.793336922 | -2.685237357 | 5.585823331 |
| H | 5.415604947 | -2.390065089 | 7.289881700 |
| H | 4.639670311 | -3.723234524 | 6.436654448 |
| H | 0.744295562 | -0.847287986 | 8.386677811 |
| H | 0.095805950 | 0.411606132 | 7.322746575 |
| H | -0.319288511 | -1.282005420 | 7.045827385 |
| H | 7.078139491 | 4.085962712 | 6.432261579 |
| H | 7.212369377 | 2.381562877 | 5.963408638 |
| H | 7.654522635 | 3.646064684 | 4.816652119 |
| H | 1.652399805 | 5.805871400 | 4.373421896 |
| H | 2.186931902 | 5.792198929 | 6.057947064 |
| H | 3.079899958 | 6.732278198 | 4.864686366 |
| H | 2.140955666 | -1.151535349 | -3.185153404 |
| H | 3.120022734 | -0.988850141 | -4.657150607 |
| H | 3.868948471 | -1.529717459 | -3.148562079 |
| H | 5.934986706 | 3.170112680 | -5.171038362 |
| H | 4.415163044 | 4.016107931 | -5.457766960 |
| H | 5.500388902 | 4.570870257 | -4.177170465 |
| H | -0.604173076 | 7.252382529 | -2.595921048 |
| H | 0.816242365 | 6.692167002 | -3.487271636 |
| H | -0.453279490 | 5.545208037 | -3.056484128 |
| H | 2.234757434 | 7.622854297 | 2.668340499 |
| H | 1.224948101 | 8.703614471 | 1.695164352 |
| H | 0.477559537 | 7.458310661 | 2.695064138 |

==============================

15B-TS1

==============================

| | | | |
|---|---|---|---|
| C | 0.249589264 | -0.235437200 | -0.033941156 |
| C | 0.157059726 | -0.181297861 | 1.964679238 |
| C | 2.688906757 | -0.065377318 | 4.289849300 |

| | | | |
|---|---|---|---|
| C | 1.575711673 | 0.173707838 | 5.105453952 |
| H | 0.864821486 | 0.948269273 | 4.839266437 |
| C | 1.382154529 | -0.547078192 | 6.286839201 |
| C | 0.175276487 | -0.295754321 | 7.160682565 |
| C | 2.331749266 | -1.513641908 | 6.639141904 |
| H | 2.196246471 | -2.076442400 | 7.561003876 |
| C | 3.459025696 | -1.766307617 | 5.849294571 |
| C | 4.469220376 | -2.809340559 | 6.267739433 |
| C | 3.630214524 | -1.030234338 | 4.670850072 |
| H | 4.509183326 | -1.210726069 | 4.058888981 |
| C | 3.658931834 | 2.512046575 | 3.751629406 |
| C | 4.853840740 | 2.461533859 | 4.472146941 |
| H | 5.474297727 | 1.570147386 | 4.440030230 |
| C | 5.270223470 | 3.560887915 | 5.232510905 |
| C | 6.593748361 | 3.525086265 | 5.958091903 |
| C | 4.447232323 | 4.691687538 | 5.279676127 |
| H | 4.757993798 | 5.545072110 | 5.879451892 |
| C | 3.230384231 | 4.755572690 | 4.588850368 |
| C | 2.359564952 | 5.986321901 | 4.681088555 |
| C | 2.847936045 | 3.653256851 | 3.821219676 |
| H | 1.905785756 | 3.671889779 | 3.281447667 |
| C | 4.119624510 | 0.210068678 | 1.736953795 |
| C | 3.776830394 | -1.149338674 | 1.443445305 |
| H | 2.985379355 | -1.637284243 | 2.000782756 |
| C | 4.424167694 | -1.839100029 | 0.450567117 |
| H | 4.149272968 | -2.869613891 | 0.243617049 |
| C | 5.435959890 | -1.219262027 | -0.325816144 |
| C | 6.117495664 | -1.919847253 | -1.354949640 |
| H | 5.879173262 | -2.968010004 | -1.516517727 |
| C | 7.056771160 | -1.286045494 | -2.136497027 |
| H | 7.569434149 | -1.832720114 | -2.922562433 |
| C | 7.350737223 | 0.082338459 | -1.923729055 |
| H | 8.078130773 | 0.581699343 | -2.556647842 |
| C | 6.722669792 | 0.783119493 | -0.919795954 |
| H | 6.950740182 | 1.831720569 | -0.771535125 |
| C | 5.757074106 | 0.156524625 | -0.082577523 |
| C | 5.080303311 | 0.865089885 | 0.964804211 |
| C | 5.413660073 | 2.306779073 | 1.184128933 |
| C | 6.667893865 | 2.631610788 | 1.796089930 |
| C | 7.562932335 | 1.631924129 | 2.271683621 |
| H | 7.295521124 | 0.586326126 | 2.166969023 |
| C | 8.760620706 | 1.979027441 | 2.856686428 |
| H | 9.431245059 | 1.201960289 | 3.211869916 |
| C | 9.131058583 | 3.339358027 | 2.997933216 |
| H | 10.082976284 | 3.596166758 | 3.453013873 |
| C | 8.282106511 | 4.330009514 | 2.558020856 |
| H | 8.552323731 | 5.377494765 | 2.663804551 |
| C | 7.036553762 | 4.006105528 | 1.956982888 |
| C | 6.148092540 | 5.007924047 | 1.496247261 |
| H | 6.433226390 | 6.051947695 | 1.598302438 |
| C | 4.945061593 | 4.679403082 | 0.922022374 |
| H | 4.298364568 | 5.471565573 | 0.567220815 |
| C | 4.552083967 | 3.321625132 | 0.770820512 |
| C | 2.102648444 | 4.458385298 | -0.431643107 |
| C | 1.597732120 | 4.707491174 | -1.713448988 |
| H | 1.801138582 | 4.014907572 | -2.523707528 |
| C | 0.845038024 | 5.859205503 | -1.979700361 |
| C | 0.282988567 | 6.098743640 | -3.361284492 |
| C | 0.616429820 | 6.763890487 | -0.940284286 |
| H | 0.047512493 | 7.669310984 | -1.141076229 |
| C | 1.108373691 | 6.542707730 | 0.352465670 |
| C | 0.831932637 | 7.534610314 | 1.457352892 |
| C | 1.841431298 | 5.378401272 | 0.598721583 |
| H | 2.234780051 | 5.204701200 | 1.596402745 |
| C | 3.594463080 | 2.292492202 | -1.680155527 |
| C | 4.402833856 | 3.155171307 | -2.438896311 |
| H | 4.615800438 | 4.156873641 | -2.074768373 |
| C | 4.930371977 | 2.744848437 | -3.663343450 |
| C | 5.784264059 | 3.669820287 | -4.498079821 |
| C | 4.640989604 | 1.448498424 | -4.115805669 |
| H | 5.043803509 | 1.122606019 | -5.072837714 |
| C | 3.864596459 | 0.561588772 | -3.368896239 |
| C | 3.598474699 | -0.847279532 | -3.841263473 |
| C | 3.352461593 | 0.996711160 | -2.138769041 |
| H | 2.768939427 | 0.305660014 | -1.539187810 |
| P | 2.961899323 | 1.069684317 | 2.883080517 |
| P | 2.950740768 | 2.877998500 | -0.061701998 |
| Rh | 1.517020344 | 1.255725272 | 0.987581131 |
| C | -0.027412699 | 2.606061409 | 1.448769611 |
| C | -0.714592279 | 2.051389219 | 2.677006839 |
| C | -0.685812277 | 0.548304014 | 2.704481197 |
| H | 0.301228387 | -1.243311765 | 2.116837909 |
| C | -1.753344786 | 0.126728630 | 3.690774887 |
| H | -0.159521334 | 2.386467082 | 3.570849391 |
| C | -2.204736076 | 2.300434527 | 2.966994133 |
| H | 0.426226283 | 3.583678414 | 1.597503439 |
| H | -0.687670160 | 2.646645034 | 0.580413815 |
| H | -2.397405227 | -0.653741366 | 3.258290022 |
| H | -1.346827570 | -0.251834879 | 4.637209215 |
| H | -2.433398089 | 3.283230223 | 3.387763266 |
| H | -2.807527649 | 2.148576494 | 2.055993386 |
| O | -2.503180359 | 1.319412846 | 3.967744299 |
| C | 0.387191339 | 0.831038946 | -0.742442048 |
| C | 0.034423483 | 1.481645161 | -2.019022335 |
| C | 0.233105183 | -1.692038550 | -0.284802592 |
| H | -0.354409355 | 2.492668918 | -1.843440916 |
| H | 0.920435998 | 1.587852994 | -2.652549085 |
| H | -0.717297073 | 0.891223172 | -2.551481384 |
| O | 0.829599882 | -2.533960297 | 0.356979699 |
| O | -0.492948588 | -1.947541512 | -1.387485827 |
| C | -0.537571885 | -3.337932829 | -1.781655137 |
| H | -1.182827651 | -3.368808146 | -2.658759032 |
| H | 0.465678680 | -3.697711691 | -2.023972225 |
| H | -0.950023653 | -3.948027278 | -0.974278602 |
| H | 5.362054475 | -2.783758569 | 5.636545885 |
| H | 4.784750637 | -2.659510132 | 7.306024767 |
| H | 4.043488295 | -3.817403344 | 6.202291589 |
| H | 0.460609333 | -0.199903421 | 8.213663064 |
| H | -0.352731627 | 0.616488448 | 6.867744426 |
| H | -0.536590280 | -1.127671120 | 7.096335200 |
| H | 6.644251095 | 4.284664556 | 6.743375929 |
| H | 6.770210141 | 2.547007941 | 6.417270076 |
| H | 7.415775056 | 3.709028856 | 5.256944295 |
| H | 1.425693463 | 5.862320705 | 4.125070808 |
| H | 2.101551262 | 6.212856318 | 5.721665996 |
| H | 2.876531124 | 6.867103892 | 4.281187660 |
| H | 2.613941898 | -1.197609857 | -3.513172169 |
| H | 3.640815731 | -0.922218106 | -4.931620525 |
| H | 4.347028026 | -1.533109367 | -3.428826819 |
| H | 6.707146939 | 3.173386367 | -4.817153261 |
| H | 5.255242339 | 3.979964203 | -5.406975299 |
| H | 6.059091370 | 4.573510030 | -3.947074721 |

H 0.124352205 7.163815415 -3.552314900
H 0.947457781 5.707601917 -4.137961281
H -0.685780468 5.597820092 -3.478203715
H 1.583248516 7.475488300 2.249036677
H 0.816705975 8.560884657 1.077563757
H -0.146438823 7.344868873 1.916052627

==============================

15B-TS2

==============================

C 0.676159234 -0.133637652 0.187531126
C -0.282266704 0.841175458 2.547241191
C 2.733108732 -0.457650310 4.088948261
C 1.670886773 -0.220391376 4.973011882
H 0.974830598 0.589162433 4.779071792
C 1.511051482 -0.989752081 6.128304843
C 0.350219291 -0.746682428 7.063263960
C 2.443904426 -1.999015778 6.395440929
H 2.332068641 -2.600218618 7.295531192
C 3.529688802 -2.237104248 5.546990997
C 4.525593569 -3.329824927 5.858549745
C 3.670778692 -1.451496101 4.395780594
H 4.529152032 -1.618528760 3.753156212
C 3.587099092 2.166424101 3.675851771
C 4.829292631 2.112667226 4.313249361
H 5.472734233 1.247045218 4.179985833
C 5.264350634 3.173531302 5.112793940
C 6.635022075 3.143138210 5.743312704
C 4.416714934 4.276182976 5.279121497
H 4.745985116 5.103387825 5.904763805
C 3.155216666 4.341355214 4.677630266
C 2.272406828 5.553544551 4.859025170
C 2.746892067 3.268289491 3.878420448
H 1.752774745 3.283085222 3.434187384
C 4.226245748 0.057400510 1.580736959
C 4.018079865 -1.314536609 1.243713886
H 3.266240858 -1.883972440 1.774879226
C 4.747550855 -1.925124370 0.257939477
H 4.585534737 -2.976233944 0.031558443
C 5.710009979 -1.200675373 -0.486095665
C 6.455956996 -1.812632945 -1.526091137
H 6.302615089 -2.870548301 -1.723872044
C 7.350544162 -1.081289227 -2.273002040
H 7.915174590 -1.556216294 -3.070075707
C 7.528500818 0.296103761 -2.006675003
H 8.220347889 0.875303237 -2.611488080
C 6.834977909 0.911495660 -0.990045671
H 6.980082071 1.968955625 -0.807608041
C 5.913419524 0.184623305 -0.185623261
C 5.170841164 0.807836127 0.880181878
C 5.450091620 2.257318386 1.158623523
C 6.710075197 2.615700303 1.746825201
C 7.659989892 1.642917422 2.167710809
H 7.423250707 0.590312648 2.058072435
C 8.872602919 2.018349133 2.702015257
H 9.582364476 1.256700434 3.011692176
C 9.206588734 3.386662326 2.849070105
H 10.170586554 3.668172751 3.262000818
C 8.302938213 4.353665303 2.470675963
H 8.541795239 5.408030953 2.584137562
C 7.041741263 3.998868154 1.921398969
C 6.101924297 4.981478941 1.526631721
H 6.352928854 6.031654970 1.653059902
C 4.894685950 4.623766709 0.980575535
H 4.201417768 5.398584626 0.677060696
C 4.554304113 3.257446480 0.786595324
C 2.088768299 4.374734486 -0.350325275
C 1.660253176 4.734903967 -1.633955571
H 1.952881885 4.143106812 -2.495513081
C 0.849702032 5.863967620 -1.826524634
C 0.425447953 6.266598916 -3.219543090
C 0.467121740 6.616176212 -0.711720697
H -0.169029985 7.487512306 -0.853178480
C 0.876857173 6.276474871 0.584910961
C 0.465360049 7.117023651 1.770527040
C 1.682267248 5.147197855 0.751991743
H 2.002822099 4.868311072 1.752817088
C 3.518960637 2.174256155 -1.630191039
C 4.306081933 2.998058875 -2.451760063
H 4.539462300 4.011941786 -2.136391723
C 4.799530816 2.527862384 -3.669579583
C 5.647860555 3.403714855 -4.561357894
C 4.501983951 1.209549230 -4.046194303
H 4.887516470 0.832603882 -4.991868698
C 3.745309516 0.361475176 -3.234563291
C 3.491922097 -1.075333835 -3.623123079
C 3.258013998 0.859164145 -2.019102510
H 2.697193309 0.203327232 -1.365655266
P 2.949450268 0.743633183 2.719609290
P 2.947180860 2.790298766 -0.005124862
Rh 1.199954324 1.460206347 1.287582684
C -0.642506535 2.527488962 0.320572338
C -1.018033250 3.030198670 1.711273241
C -1.145735881 1.843989922 2.668877906
H -0.298867864 -0.113688596 3.062446903
C -2.380359975 2.082831351 3.521716169
H -0.304707447 3.783478754 2.069275111
C -2.447814447 3.632803427 1.823883050
H -0.286467872 3.281075887 -0.378368262
H -1.496796761 1.979346119 -0.072679989
H -3.159721640 1.336407048 3.295631704
H -2.187429279 2.064818766 4.598512202
H -2.489592167 4.708423177 1.635028494
H -3.130949503 3.117007905 1.126690926
O -2.815783178 3.400219899 3.174473891
C 0.194135242 0.732557311 -0.627490261
C -0.140501451 1.022113558 -2.034344046
C 0.866263794 -1.574765220 0.361376722
H -1.148757628 1.425590461 -2.163637569
H 0.576361417 1.740595890 -2.446440886
H -0.052743000 0.088757481 -2.599874085
O 0.960947941 -2.138945510 1.432122231
O 0.938469733 -2.187159500 -0.838980940
C 1.135062143 -3.618407573 -0.785775016
H 1.145071458 -3.949448358 -1.823458591
H 2.084909058 -3.848435925 -0.296675072
H 0.322983973 -4.095474017 -0.231651419
H 5.477372456 -3.165956086 5.344580382
H 4.726275002 -3.393325448 6.932792659
H 4.143717655 -4.307855150 5.541088462
H 0.662750969 -0.805054196 8.110829128
H -0.099368016 0.236948690 6.898403948
H -0.433273801 -1.499745771 6.916165181

| | | | |
|---|---|---|---|
| H | 6.683555947 | 3.780088684 | 6.631162259 |
| H | 6.919469988 | 2.127265834 | 6.034245205 |
| H | 7.387216579 | 3.501280281 | 5.031104732 |
| H | 1.215541728 | 5.306621047 | 4.716766222 |
| H | 2.384853282 | 5.989495763 | 5.856453337 |
| H | 2.530410242 | 6.334924268 | 4.132650125 |
| H | 2.656511875 | -1.498745847 | -3.056762119 |
| H | 3.272842034 | -1.172458329 | -4.691353267 |
| H | 4.379435731 | -1.682620464 | -3.410988482 |
| H | 6.617175470 | 2.933560685 | -4.763629169 |
| H | 5.163059004 | 3.570109906 | -5.530497329 |
| H | 5.836021442 | 4.380995943 | -4.107948447 |
| H | -0.484281746 | 6.873884589 | -3.204295110 |
| H | 1.208943335 | 6.860631331 | -3.706819410 |
| H | 0.242051974 | 5.391932860 | -3.852239235 |
| H | 0.385542785 | 6.517635631 | 2.681635106 |
| H | 1.202657239 | 7.905371437 | 1.963583188 |
| H | -0.496523272 | 7.608393161 | 1.599090259 |

==============================

16A

==============================

| | | | |
|---|---|---|---|
| C | 2.399611877 | 6.270557399 | 11.432043708 |
| C | 2.467306404 | 7.562929484 | 11.980067909 |
| H | 2.707658895 | 7.694291460 | 13.033957200 |
| C | 2.238228592 | 8.694379380 | 11.191620371 |
| C | 2.325247772 | 10.076007239 | 11.796014822 |
| C | 1.940806101 | 8.509300138 | 9.835818897 |
| H | 1.749475250 | 9.380192452 | 9.211839755 |
| C | 1.884976373 | 7.234103847 | 9.258182771 |
| C | 1.574597875 | 7.073395737 | 7.788182904 |
| C | 2.118168251 | 6.116072821 | 10.070277970 |
| H | 2.063341586 | 5.124244517 | 9.633260452 |
| C | 2.506768215 | 3.314336977 | 11.721845417 |
| C | 3.399183643 | 3.069716263 | 10.669133652 |
| H | 4.083559434 | 3.849385568 | 10.343473966 |
| C | 3.419619444 | 1.824046329 | 10.033018162 |
| C | 4.392489937 | 1.542736974 | 8.912185132 |
| C | 2.549631844 | 0.825202735 | 10.489083519 |
| H | 2.566150190 | -0.148418438 | 10.002893664 |
| C | 1.672822613 | 1.033410295 | 11.559114306 |
| C | 0.806206038 | -0.085724712 | 12.082293127 |
| C | 1.657998713 | 2.293509284 | 12.162976250 |
| H | 1.006378882 | 2.471458074 | 13.009113477 |
| C | 4.216739275 | 5.006656706 | 13.285793473 |
| C | 5.168070749 | 5.881092846 | 12.691741111 |
| H | 4.866255729 | 6.528296975 | 11.877380905 |
| C | 6.470890475 | 5.898648473 | 13.121217101 |
| H | 7.194314960 | 6.557604362 | 12.648195075 |
| C | 6.900055203 | 5.040767673 | 14.164402480 |
| C | 8.257671415 | 5.000184582 | 14.578648860 |
| H | 8.972098907 | 5.653502131 | 14.084500682 |
| C | 8.666643234 | 4.143360588 | 15.574763806 |
| H | 9.709893735 | 4.111606811 | 15.874941744 |
| C | 7.723059062 | 3.307139868 | 16.217241453 |
| H | 8.047392362 | 2.643462830 | 17.013525944 |
| C | 6.399193305 | 3.326351312 | 15.840916916 |
| H | 5.690341146 | 2.676208216 | 16.341042686 |
| C | 5.946099630 | 4.174967688 | 14.792195536 |
| C | 4.585807245 | 4.170622008 | 14.331631269 |
| C | 3.639651510 | 3.161727976 | 14.918929739 |
| C | 3.875451551 | 1.789412690 | 14.560341726 |
| C | 4.837560264 | 1.425245697 | 13.573672336 |
| H | 5.403477860 | 2.198765199 | 13.070389404 |
| C | 5.031289759 | 0.108301758 | 13.229503946 |
| H | 5.758378127 | -0.145377925 | 12.463428895 |
| C | 4.292910218 | -0.922118290 | 13.860050861 |
| H | 4.468895708 | -1.958560638 | 13.586706442 |
| C | 3.351091614 | -0.607212891 | 14.810915770 |
| H | 2.773578041 | -1.387545749 | 15.298865132 |
| C | 3.110341102 | 0.744792886 | 15.171744522 |
| C | 2.132370601 | 1.095708296 | 16.130967491 |
| H | 1.574991313 | 0.309280383 | 16.633686645 |
| C | 1.876192734 | 2.412140659 | 16.422475811 |
| H | 1.119649388 | 2.642327015 | 17.160361013 |
| C | 2.598718987 | 3.472139781 | 15.802996955 |
| C | 1.015853842 | 5.175244530 | 17.632152885 |
| C | 1.570051687 | 5.831613899 | 18.735470327 |
| H | 2.499429718 | 6.381008883 | 18.637248913 |
| C | 0.933983195 | 5.802320841 | 19.985939304 |
| C | 1.564757530 | 6.508557737 | 21.162814815 |
| C | -0.272110445 | 5.111213784 | 20.111091666 |
| H | -0.772193680 | 5.084312573 | 21.077186217 |
| C | -0.864006289 | 4.460968836 | 19.018521043 |
| C | -2.184629982 | 3.745508840 | 19.175565059 |
| C | -0.211738173 | 4.506608343 | 17.787539551 |
| H | -0.666412781 | 3.998102060 | 16.949638675 |
| C | 3.232425672 | 6.380527509 | 16.224511885 |
| C | 4.411548661 | 6.041043949 | 16.895194845 |
| H | 4.561751193 | 5.027440365 | 17.255211135 |
| C | 5.410831245 | 6.998148485 | 17.104386444 |
| C | 6.699936299 | 6.619170635 | 17.791146475 |
| C | 5.197183888 | 8.297669432 | 16.631756009 |
| H | 5.968167786 | 9.049093996 | 16.792184832 |
| C | 4.014936582 | 8.666466352 | 15.977760277 |
| C | 3.810935089 | 10.079424052 | 15.485783790 |
| C | 3.029711974 | 7.695296605 | 15.782647651 |
| H | 2.078832415 | 7.981737765 | 15.344422234 |
| P | 2.498615518 | 4.891730770 | 12.636325293 |
| P | 1.886554781 | 5.175019058 | 16.009462475 |
| Rh | 0.676117309 | 5.649630193 | 14.100825457 |
| H | -0.008867817 | 0.293836582 | 12.706311218 |
| H | 0.368839061 | -0.671578454 | 11.267791220 |
| H | 1.404188498 | -0.766851166 | 12.699400557 |
| H | 3.972658486 | 0.843221927 | 8.182911672 |
| H | 4.676189399 | 2.457543749 | 8.383653567 |
| H | 5.311940365 | 1.090996759 | 9.304188753 |
| H | 1.485773890 | 6.020495838 | 7.506607153 |
| H | 0.636221069 | 7.574308799 | 7.524952312 |
| H | 2.361358491 | 7.520433907 | 7.169620634 |
| H | 1.702263703 | 10.792707039 | 11.253003544 |
| H | 2.010444752 | 10.072748542 | 12.844418348 |
| H | 3.355606631 | 10.450334240 | 11.767528967 |
| H | 0.890004183 | 6.543716463 | 22.022248071 |
| H | 2.482504791 | 5.998834349 | 21.479332818 |
| H | 1.838542935 | 7.536652968 | 20.902918433 |
| H | -2.510370337 | 3.286761328 | 18.237684973 |
| H | -2.120698221 | 2.956793519 | 19.933737707 |
| H | -2.969799728 | 4.438735539 | 19.498540087 |
| H | 2.754551020 | 10.283314835 | 15.287672324 |
| H | 4.166021159 | 10.812920969 | 16.216657454 |
| H | 4.366375954 | 10.253264088 | 14.556149049 |
| H | 7.167690122 | 7.482684813 | 18.272990677 |

| | | | |
|---|---|---|---|
| H | 6.538081991 | 5.848808761 | 18.551241544 |
| H | 7.411421757 | 6.211634693 | 17.065004441 |
| C | -0.766134585 | 4.171265832 | 14.325359344 |
| C | -0.655539124 | 6.984624585 | 14.816562829 |
| C | -1.671483437 | 4.508373277 | 13.156100720 |
| C | -1.115373214 | 7.585939987 | 13.692540030 |
| C | -0.976561454 | 5.519302054 | 12.226490427 |
| C | -0.820303559 | 6.860850772 | 12.436815500 |
| H | -0.472184175 | 7.458381354 | 11.592225739 |
| C | -0.828358509 | 4.827418722 | 10.884891335 |
| C | -1.794149921 | 8.929853300 | 13.596107933 |
| C | -1.980775418 | 3.347958234 | 12.170388104 |
| H | -2.592665294 | 4.973236344 | 13.525173869 |
| C | -0.824473304 | 7.497780199 | 16.189347063 |
| H | -0.260814975 | 3.206483464 | 14.269488051 |
| H | -1.266190478 | 4.294757845 | 15.280117154 |
| O | -1.922828559 | 3.916261420 | 10.866442352 |
| H | -0.916227235 | 5.508450920 | 10.034843407 |
| H | 0.125684071 | 4.286582864 | 10.807132167 |
| H | -1.230886890 | 2.548087195 | 12.275772849 |
| H | -2.975222270 | 2.917768934 | 12.309547987 |
| H | -1.235456580 | 9.592559756 | 12.922595277 |
| H | -2.806608221 | 8.833401514 | 13.186103709 |
| H | -1.862021046 | 9.415992856 | 14.572399330 |
| O | 0.012220313 | 8.147597524 | 16.790847517 |
| O | -2.013335001 | 7.141111988 | 16.713474074 |
| C | -2.250052841 | 7.595334920 | 18.063702374 |
| H | -3.231740730 | 7.206621339 | 18.333749470 |
| H | -1.481386504 | 7.205401997 | 18.732335880 |
| H | -2.244950147 | 8.687615527 | 18.103885221 |

==============================

16B

==============================

| | | | |
|---|---|---|---|
| C | 1.788798589 | 5.460072192 | 10.626412085 |
| C | 1.608926134 | 6.827327555 | 10.374228283 |
| H | 1.626908070 | 7.533302429 | 11.199831213 |
| C | 1.404836451 | 7.306797632 | 9.074966811 |
| C | 1.235238764 | 8.787833745 | 8.828631735 |
| C | 1.373819104 | 6.382603068 | 8.026412427 |
| H | 1.206911033 | 6.738577608 | 7.011684277 |
| C | 1.548558730 | 5.009411836 | 8.245650332 |
| C | 1.507483138 | 4.041579323 | 7.086505042 |
| C | 1.754387980 | 4.558151195 | 9.553482795 |
| H | 1.883448062 | 3.495714938 | 9.732232686 |
| C | 1.762775342 | 3.097902266 | 12.249634418 |
| C | 2.743391406 | 2.139073045 | 11.997182271 |
| H | 3.777975816 | 2.436230011 | 11.861528020 |
| C | 2.404306401 | 0.779192408 | 11.918296798 |
| C | 3.484274518 | -0.251584568 | 11.688932479 |
| C | 1.063396089 | 0.415181911 | 12.069352620 |
| H | 0.791956461 | -0.636986677 | 12.007550645 |
| C | 0.052835097 | 1.367383144 | 12.275762483 |
| C | -1.392900719 | 0.943966494 | 12.390667728 |
| C | 0.416285184 | 2.711198257 | 12.367959998 |
| H | -0.357347146 | 3.466765076 | 12.479046849 |
| C | 3.815124191 | 5.340588968 | 12.725705766 |
| C | 4.352885981 | 6.452423496 | 12.012280757 |
| H | 3.851061495 | 6.812468343 | 11.124598797 |
| C | 5.515069233 | 7.065094128 | 12.403585583 |
| H | 5.911924464 | 7.896577739 | 11.826298819 |
| C | 6.212185097 | 6.629882571 | 13.554586300 |
| C | 7.395168507 | 7.279048037 | 13.996473743 |
| H | 7.764057194 | 8.131344670 | 13.431353499 |
| C | 8.060971883 | 6.838089419 | 15.116222437 |
| H | 8.964975569 | 7.340023499 | 15.448776967 |
| C | 7.562717460 | 5.728816915 | 15.839811286 |
| H | 8.080168893 | 5.395401329 | 16.734700879 |
| C | 6.428033389 | 5.070829023 | 15.425774989 |
| H | 6.054161276 | 4.233714969 | 16.001676721 |
| C | 5.715759051 | 5.495031680 | 14.268854010 |
| C | 4.523566786 | 4.830832237 | 13.816107542 |
| C | 4.175757031 | 3.533013234 | 14.475122513 |
| C | 5.062829198 | 2.424863468 | 14.225116152 |
| C | 6.138554077 | 2.507061278 | 13.294776167 |
| H | 6.293509806 | 3.423858699 | 12.738023661 |
| C | 6.982116526 | 1.438986087 | 13.088330015 |
| H | 7.794025275 | 1.526533232 | 12.371989171 |
| C | 6.805338898 | 0.230089719 | 13.802269916 |
| H | 7.486707227 | -0.599479759 | 13.637565326 |
| C | 5.765353476 | 0.107451785 | 14.694236713 |
| H | 5.610775753 | -0.818636064 | 15.241731483 |
| C | 4.869245364 | 1.186286733 | 14.915798348 |
| C | 3.785023017 | 1.079867430 | 15.817039449 |
| H | 3.637290545 | 0.149698848 | 16.359899915 |
| C | 2.927595435 | 2.130495183 | 16.016207420 |
| H | 2.123222970 | 2.007117137 | 16.725995968 |
| C | 3.099784672 | 3.375411995 | 15.341702149 |
| C | 0.780637933 | 4.013585652 | 16.901928172 |
| C | 0.544913536 | 4.422305984 | 18.214659840 |
| H | 1.138969269 | 5.216554686 | 18.656118286 |
| C | -0.459096112 | 3.807718218 | 18.984605717 |
| C | -0.690889662 | 4.254971555 | 20.409000994 |
| C | -1.212264443 | 2.783805109 | 18.409236243 |
| H | -1.990847721 | 2.303434006 | 18.998213768 |
| C | -0.992084473 | 2.351069497 | 17.090846512 |
| C | -1.819473443 | 1.233764079 | 16.499587425 |
| C | 0.004346364 | 2.976679758 | 16.347717681 |
| H | 0.189213651 | 2.648362283 | 15.327273981 |
| C | 3.155692255 | 5.810669738 | 16.794774579 |
| C | 3.762788958 | 5.253012117 | 17.928864241 |
| H | 3.493373690 | 4.250983987 | 18.253505718 |
| C | 4.725295057 | 5.971040155 | 18.645569685 |
| C | 5.351488262 | 5.381439844 | 19.887646529 |
| C | 5.088257304 | 7.243925229 | 18.186865251 |
| H | 5.848653982 | 7.802417908 | 18.728837083 |
| C | 4.520952958 | 7.810841317 | 17.040922261 |
| C | 4.983657846 | 9.147335909 | 16.513343775 |
| C | 3.543383068 | 7.081983490 | 16.355408209 |
| H | 3.092719319 | 7.512225640 | 15.464595336 |
| P | 2.049949110 | 4.893730672 | 12.355897124 |
| P | 1.995840181 | 4.810198972 | 15.792503622 |
| Rh | 0.857713915 | 6.088720484 | 14.194080964 |
| H | -2.050875455 | 1.803768480 | 12.546614229 |
| H | -1.726269527 | 0.429235014 | 11.482489968 |
| H | -1.537918064 | 0.246267794 | 13.224188849 |
| H | 3.063475311 | -1.236103779 | 11.466323326 |
| H | 4.135620239 | 0.036615250 | 10.856461813 |
| H | 4.123940724 | -0.346697923 | 12.574214406 |
| H | 1.714468609 | 3.017414925 | 7.408615582 |
| H | 0.523653122 | 4.049571361 | 6.603250511 |
| H | 2.244579910 | 4.311475104 | 6.321852872 |
| H | 0.804588323 | 8.986022713 | 7.843520277 |

| | | | |
|---|---|---|---|
| H | 0.583382828 | 9.246471070 | 9.580748758 |
| H | 2.200331134 | 9.306619899 | 8.877777339 |
| H | -1.571700903 | 3.776118915 | 20.844853209 |
| H | 0.170551452 | 4.009474895 | 21.041534709 |
| H | -0.832519105 | 5.340081082 | 20.465210381 |
| H | -1.563373923 | 1.057501885 | 15.450917401 |
| H | -1.664400433 | 0.296273537 | 17.046144055 |
| H | -2.889713280 | 1.463501325 | 16.552927013 |
| H | 4.155020341 | 9.712448168 | 16.074014152 |
| H | 5.433437340 | 9.758487499 | 17.301217676 |
| H | 5.740160727 | 9.000171563 | 15.732532161 |
| H | 6.286475653 | 5.887408224 | 20.144268247 |
| H | 4.679413535 | 5.475637814 | 20.749396784 |
| H | 5.565628411 | 4.315285902 | 19.758279617 |
| C | -1.013425730 | 5.266893563 | 14.645824722 |
| C | 0.264931626 | 7.578179086 | 15.450262365 |
| C | -1.947511764 | 6.208947429 | 13.920412007 |
| C | -0.015966159 | 8.540985841 | 14.524970677 |
| C | -1.086097319 | 6.961689995 | 12.897498625 |
| C | -0.268020147 | 8.030019718 | 13.156897265 |
| H | 0.137472288 | 8.586017142 | 12.314764900 |
| C | -1.489873403 | 6.431357663 | 11.533971701 |
| C | -0.021889261 | 10.001448975 | 14.796348628 |
| C | -2.955991067 | 5.496195236 | 12.998776382 |
| H | -2.412849742 | 6.915089487 | 14.616066960 |
| C | 0.244786639 | 7.717376754 | 16.933366338 |
| H | -0.979656190 | 4.269894405 | 14.210014235 |
| H | -1.160960058 | 5.222010371 | 15.721277391 |
| O | -2.226576703 | 5.240245239 | 11.798163423 |
| H | -2.128969130 | 7.184914924 | 11.038979178 |
| H | -0.662326064 | 6.191292904 | 10.869295791 |
| H | -3.311144304 | 4.540367987 | 13.392534768 |
| H | -3.823398655 | 6.145513949 | 12.796871713 |
| O | 0.207009608 | 10.520902224 | 15.872582016 |
| O | -0.291033186 | 10.707621114 | 13.670821418 |
| C | -0.284124519 | 12.140195577 | 13.831853665 |
| H | 0.694008218 | 12.482175052 | 14.181104169 |
| H | -1.042414908 | 12.446100340 | 14.557299822 |
| H | -0.507159398 | 12.548374482 | 12.846158332 |
| H | -0.256712695 | 6.850695534 | 17.378042284 |
| H | 1.260573332 | 7.736869045 | 17.340046914 |
| H | -0.258339955 | 8.636313714 | 17.241662673 |

==============================

18A

==============================

| | | | |
|---|---|---|---|
| C | -1.276964682 | 3.153338386 | 1.733763441 |
| C | -0.087609886 | 0.212182111 | 1.313408686 |
| C | 2.268687487 | 0.403413582 | 3.819514633 |
| C | 1.546829315 | 0.927793405 | 4.901433762 |
| H | 1.142429094 | 1.933224913 | 4.854101643 |
| C | 1.361467853 | 0.188175981 | 6.073630745 |
| C | 0.532099919 | 0.755103593 | 7.200822963 |
| C | 1.942641509 | -1.079785185 | 6.159613574 |
| H | 1.815293733 | -1.661228472 | 7.070339490 |
| C | 2.706146231 | -1.612243529 | 5.113939101 |
| C | 3.349202476 | -2.972265726 | 5.252701305 |
| C | 2.861847261 | -0.862443276 | 3.944883193 |
| H | 3.480660709 | -1.267863466 | 3.154668448 |
| C | 3.024063893 | 3.029437661 | 3.158252237 |
| C | 4.069387741 | 2.989425610 | 4.091248636 |
| H | 4.599293262 | 2.060388689 | 4.281857460 |
| C | 4.440207528 | 4.144434566 | 4.781276119 |
| C | 5.608461676 | 4.121448669 | 5.737218024 |
| C | 3.724448183 | 5.326195577 | 4.540372497 |
| H | 4.000159955 | 6.226634907 | 5.085403966 |
| C | 2.666854876 | 5.382809743 | 3.627778961 |
| C | 1.930861514 | 6.669726320 | 3.345373997 |
| C | 2.320998083 | 4.215650632 | 2.939671346 |
| H | 1.493531149 | 4.226274400 | 2.239710961 |
| C | 3.707739629 | 0.698668404 | 1.237786349 |
| C | 3.361276537 | -0.631428934 | 0.837472515 |
| H | 2.464863596 | -1.092818187 | 1.236896686 |
| C | 4.142746062 | -1.328184822 | -0.047850383 |
| H | 3.869128584 | -2.339269899 | -0.337166397 |
| C | 5.313508505 | -0.746542704 | -0.590795157 |
| C | 6.155074445 | -1.471996180 | -1.472422849 |
| H | 5.895464212 | -2.499509669 | -1.712928646 |
| C | 7.282432770 | -0.891210809 | -2.004174528 |
| H | 7.926876921 | -1.457234289 | -2.670221852 |
| C | 7.603643902 | 0.448843692 | -1.685865100 |
| H | 8.490982942 | 0.905294223 | -2.114535235 |
| C | 6.798437860 | 1.184301880 | -0.847815019 |
| H | 7.054758340 | 2.212794456 | -0.626936990 |
| C | 5.637662090 | 0.610757998 | -0.256483599 |
| C | 4.794609683 | 1.345716522 | 0.643913061 |
| C | 5.056604076 | 2.809134666 | 0.838647233 |
| C | 6.196540011 | 3.231451311 | 1.591945912 |
| C | 7.051230806 | 2.308811192 | 2.255498750 |
| H | 6.833563450 | 1.247634950 | 2.194798647 |
| C | 8.149289084 | 2.744985857 | 2.961604884 |
| H | 8.792819456 | 2.024584194 | 3.458225727 |
| C | 8.450058364 | 4.126229977 | 3.045122936 |
| H | 9.324597399 | 4.455166515 | 3.598844842 |
| C | 7.631067679 | 5.047108302 | 2.432889481 |
| H | 7.847656193 | 6.110027268 | 2.500292112 |
| C | 6.489294902 | 4.629835758 | 1.699515216 |
| C | 5.630361366 | 5.559977739 | 1.066562293 |
| H | 5.855206252 | 6.620727191 | 1.143392019 |
| C | 4.525680115 | 5.138880532 | 0.368254378 |
| H | 3.878040394 | 5.870744978 | -0.100208166 |
| C | 4.222386824 | 3.757261100 | 0.244792524 |
| C | 1.971799427 | 4.808090053 | -1.260556696 |
| C | 2.250503166 | 5.342511255 | -2.524564110 |
| H | 2.904042950 | 4.812898595 | -3.210556785 |
| C | 1.686780515 | 6.560753577 | -2.926488683 |
| C | 2.019603098 | 7.135983577 | -4.283110512 |
| C | 0.830678991 | 7.230649739 | -2.044102596 |
| H | 0.382583029 | 8.171973319 | -2.354336733 |
| C | 0.525574103 | 6.714141852 | -0.778877590 |
| C | -0.423445835 | 7.417333753 | 0.161878996 |
| C | 1.105931465 | 5.500039882 | -0.400398402 |
| H | 0.842348766 | 5.073868119 | 0.560548241 |
| C | 3.375798660 | 2.345527195 | -2.096219170 |
| C | 4.538958835 | 2.799118895 | -2.734922639 |
| H | 5.014227366 | 3.718741995 | -2.403090324 |
| C | 5.130233730 | 2.056276239 | -3.759800748 |
| C | 6.386177260 | 2.540641004 | -4.444099399 |
| C | 4.542050696 | 0.838656811 | -4.123009527 |
| H | 5.013844130 | 0.236450456 | -4.896749617 |
| C | 3.384791912 | 0.358363449 | -3.502639022 |
| C | 2.833009766 | -1.002019803 | -3.853084578 |
| C | 2.799480568 | 1.131946453 | -2.493463630 |

| | | | |
|---|---|---|---|
| H | 1.911881474 | 0.755689965 | -1.989123487 |
| P | 2.476392302 | 1.492235738 | 2.358478032 |
| P | 2.687206377 | 3.232853441 | -0.650773412 |
| Rh | 1.103722406 | 1.680673666 | 0.572245149 |
| C | -0.978169110 | 2.657767787 | 3.131830809 |
| C | -1.371367328 | 1.188789052 | 3.349676979 |
| C | -0.972630909 | 0.154305087 | 2.313386919 |
| H | -0.001166160 | -0.662552032 | 0.652351416 |
| C | -1.933838412 | -1.011815495 | 2.537589417 |
| H | -0.939087727 | 0.864907354 | 4.308729002 |
| C | -2.878507917 | 0.894197696 | 3.442037581 |
| H | 0.087170813 | 2.779718151 | 3.330221786 |
| H | -1.512573670 | 3.273612753 | 3.860701031 |
| H | -2.361687698 | -1.355900219 | 1.583471275 |
| H | -1.442013293 | -1.869132421 | 3.017356006 |
| H | -3.336069291 | 1.244267923 | 4.371116145 |
| H | -3.418978499 | 1.350014081 | 2.597859317 |
| O | -2.953908864 | -0.531234711 | 3.413741033 |
| C | -0.538990836 | 2.756883834 | 0.688521359 |
| C | -0.832860617 | 2.758770726 | -0.786066792 |
| C | -2.518739135 | 3.953377069 | 1.586627666 |
| H | -0.674852269 | 3.742170084 | -1.233514484 |
| H | -0.163356744 | 2.058498571 | -1.333199825 |
| H | -1.854074285 | 2.416364554 | -0.974369167 |
| O | -3.353617978 | 4.072815262 | 2.463562975 |
| O | -2.625285958 | 4.564444322 | 0.379942994 |
| C | -3.839032953 | 5.316333988 | 0.178403133 |
| H | -3.761407356 | 5.729051950 | -0.827524204 |
| H | -4.712606061 | 4.665197959 | 0.263776934 |
| H | -3.921636922 | 6.115921437 | 0.918848570 |
| H | 4.028483705 | -3.185878171 | 4.422606250 |
| H | 3.919783146 | -3.047387581 | 6.184613751 |
| H | 2.589934364 | -3.762655924 | 5.274985767 |
| H | 0.731833389 | 0.243055115 | 8.145790669 |
| H | 0.728278347 | 1.822851488 | 7.342709359 |
| H | -0.538200277 | 0.646023888 | 6.985391301 |
| H | 5.537899248 | 4.922683508 | 6.478230243 |
| H | 5.671111937 | 3.167454943 | 6.270082589 |
| H | 6.547396988 | 4.253965702 | 5.187947956 |
| H | 0.858125152 | 6.495302091 | 3.212365095 |
| H | 2.060709522 | 7.398424762 | 4.149984220 |
| H | 2.303567099 | 7.123488119 | 2.418364418 |
| H | 1.763582689 | -1.075696463 | -3.633777711 |
| H | 2.981151541 | -1.238168386 | -4.911072397 |
| H | 3.345012037 | -1.778802277 | -3.270096682 |
| H | 7.040342869 | 1.704140438 | -4.709059034 |
| H | 6.149623880 | 3.076308983 | -5.371733196 |
| H | 6.952150045 | 3.223875268 | -3.803755504 |
| H | 1.357780358 | 7.966133136 | -4.543424260 |
| H | 3.049134052 | 7.513335737 | -4.304793243 |
| H | 1.937581029 | 6.376502454 | -5.067730335 |
| H | 0.100935527 | 7.780783613 | 1.053505409 |
| H | -0.904876725 | 8.275036069 | -0.315160736 |
| H | -1.203787624 | 6.726674849 | 0.498870393 |

==============================

18B

==============================

| | | | |
|---|---|---|---|
| C | -1.289224007 | 3.135866127 | 1.754472166 |
| C | -0.085370788 | 0.172809824 | 1.287390948 |
| C | 2.250142259 | 0.379084064 | 3.866821112 |
| C | 1.537190927 | 0.928125235 | 4.943159699 |
| H | 1.188355961 | 1.954727615 | 4.900376758 |
| C | 1.287367559 | 0.187041972 | 6.101905110 |
| C | 0.460731706 | 0.779866293 | 7.218328369 |
| C | 1.796523834 | -1.112203217 | 6.182929729 |
| H | 1.617210580 | -1.697631606 | 7.082437365 |
| C | 2.551728797 | -1.671313370 | 5.145746572 |
| C | 3.108964789 | -3.069158217 | 5.273322867 |
| C | 2.774163901 | -0.915776770 | 3.989726224 |
| H | 3.384148559 | -1.345053970 | 3.204972923 |
| C | 3.059419148 | 2.991992931 | 3.218635730 |
| C | 4.102840691 | 2.950567959 | 4.152273118 |
| H | 4.630232778 | 2.019755742 | 4.341434262 |
| C | 4.475281243 | 4.104056597 | 4.844123441 |
| C | 5.642885156 | 4.080249036 | 5.800134561 |
| C | 3.762129585 | 5.287591479 | 4.603260984 |
| H | 4.040141575 | 6.187677293 | 5.147993614 |
| C | 2.702808100 | 5.345198501 | 3.692918190 |
| C | 1.968662286 | 6.634257682 | 3.415694123 |
| C | 2.356361511 | 4.178908207 | 3.003266547 |
| H | 1.528756535 | 4.188175206 | 2.302385183 |
| C | 3.749884213 | 0.671447071 | 1.307959952 |
| C | 3.387667948 | -0.647005542 | 0.882804869 |
| H | 2.490299546 | -1.109738992 | 1.278661888 |
| C | 4.154346226 | -1.331104484 | -0.026214394 |
| H | 3.863123099 | -2.329708986 | -0.340897782 |
| C | 5.332234549 | -0.754594306 | -0.557344230 |
| C | 6.157786082 | -1.473261941 | -1.462498835 |
| H | 5.874215896 | -2.487573860 | -1.731032982 |
| C | 7.296492834 | -0.903775078 | -1.982439345 |
| H | 7.926387041 | -1.463705873 | -2.667141394 |
| C | 7.644756755 | 0.420551571 | -1.623591670 |
| H | 8.541447331 | 0.870885256 | -2.039303956 |
| C | 6.854023905 | 1.150657905 | -0.766607132 |
| H | 7.131856482 | 2.167538579 | -0.519586414 |
| C | 5.679437866 | 0.587912042 | -0.190851826 |
| C | 4.841064983 | 1.317792471 | 0.718466275 |
| C | 5.099192596 | 2.784227311 | 0.906346931 |
| C | 6.227403321 | 3.222847696 | 1.667178045 |
| C | 7.080460745 | 2.313474109 | 2.350846296 |
| H | 6.867523651 | 1.250665440 | 2.301337375 |
| C | 8.171009954 | 2.764870961 | 3.059258617 |
| H | 8.814555540 | 2.054794263 | 3.570636765 |
| C | 8.464534972 | 4.149016866 | 3.125188511 |
| H | 9.333510710 | 4.490110400 | 3.680495352 |
| C | 7.646993527 | 5.057369115 | 2.492357307 |
| H | 7.859214377 | 6.122116313 | 2.544868155 |
| C | 6.513875890 | 4.624530787 | 1.754554531 |
| C | 5.660259650 | 5.541609573 | 1.094943219 |
| H | 5.880708348 | 6.604379795 | 1.157303392 |
| C | 4.570072555 | 5.103861297 | 0.383814406 |
| H | 3.927834172 | 5.824616897 | -0.109555917 |
| C | 4.274615828 | 3.719146415 | 0.279334131 |
| C | 2.007344019 | 4.688743692 | -1.283433625 |
| C | 2.138499087 | 5.087501912 | -2.620263420 |
| H | 2.740170837 | 4.498720899 | -3.305945063 |
| C | 1.501506285 | 6.244147249 | -3.088202356 |
| C | 1.681479751 | 6.685530584 | -4.522215902 |
| C | 0.718987960 | 6.987391257 | -2.196369572 |
| H | 0.211498957 | 7.880515533 | -2.555362148 |
| C | 0.566218134 | 6.609332535 | -0.857205096 |
| C | -0.330814054 | 7.380861701 | 0.080977313 |

| | | | |
|---|---|---|---|
| C | 1.221442106 | 5.457723165 | -0.412946736 |
| H | 1.094357169 | 5.146429728 | 0.617250115 |
| C | 3.484238426 | 2.300942453 | -2.085465946 |
| C | 4.677214620 | 2.754762768 | -2.665096567 |
| H | 5.170761108 | 3.638185061 | -2.268464719 |
| C | 5.265091391 | 2.062122741 | -3.726765190 |
| C | 6.551489030 | 2.550397236 | -4.348623609 |
| C | 4.639606185 | 0.899178669 | -4.190985015 |
| H | 5.103726534 | 0.338515346 | -5.000134531 |
| C | 3.445454792 | 0.427935219 | -3.636179116 |
| C | 2.830424067 | -0.865201713 | -4.115742402 |
| C | 2.866370045 | 1.147266656 | -2.584777134 |
| H | 1.934773546 | 0.797864216 | -2.151505077 |
| P | 2.498378094 | 1.466474863 | 2.407415503 |
| P | 2.768899468 | 3.153707657 | -0.631445900 |
| Rh | 1.158823848 | 1.610226158 | 0.588514258 |
| C | -1.008754485 | 2.609294836 | 3.142397281 |
| C | -1.374591874 | 1.123642637 | 3.337854261 |
| C | -0.968659311 | 0.104414537 | 2.286544548 |
| H | -0.036492400 | -0.679434872 | 0.595861622 |
| C | -1.916947099 | -1.074573820 | 2.505625302 |
| H | -0.930226322 | 0.805826588 | 4.292161543 |
| C | -2.875498191 | 0.801574763 | 3.443398916 |
| H | 0.052917417 | 2.737502430 | 3.357191956 |
| H | -1.553036734 | 3.199469441 | 3.889954781 |
| H | -2.361743607 | -1.403950011 | 1.554658693 |
| H | -1.416940908 | -1.936511917 | 2.966339388 |
| H | -3.333458618 | 1.140650228 | 4.377992891 |
| H | -3.431170655 | 1.243131750 | 2.597714742 |
| O | -2.929204293 | -0.618626159 | 3.410465558 |
| C | -0.469626129 | 2.792804500 | 0.745666896 |
| C | -0.696690116 | 2.830264701 | -0.695036153 |
| C | -2.523446917 | 3.989383955 | 1.592811309 |
| H | 3.827253409 | -3.289621637 | 4.479209920 |
| H | 3.612346217 | -3.210761462 | 6.235431616 |
| H | 2.307593431 | -3.815178495 | 5.215008796 |
| H | 0.665274373 | 0.291596193 | 8.175182068 |
| H | 0.653320437 | 1.851391413 | 7.333650072 |
| H | -0.610482992 | 0.660322228 | 7.010893268 |
| H | 5.554832485 | 4.860041259 | 6.562037625 |
| H | 5.725886584 | 3.113883102 | 6.306906145 |
| H | 6.578616086 | 4.247759208 | 5.254903064 |
| H | 0.901564502 | 6.456726464 | 3.244603634 |
| H | 2.067483399 | 7.346177012 | 4.239642735 |
| H | 2.367690910 | 7.111533236 | 2.511833082 |
| H | 1.756808910 | -0.902267501 | -3.908382842 |
| H | 2.975694972 | -1.007651170 | -5.191151501 |
| H | 3.295071747 | -1.720911235 | -3.608621196 |
| H | 7.147241031 | 1.718219730 | -4.736084680 |
| H | 6.351902543 | 3.227957008 | -5.187444569 |
| H | 7.162793141 | 3.096256666 | -3.623406608 |
| H | 0.871582528 | 7.345708928 | -4.845522073 |
| H | 2.622221904 | 7.236330536 | -4.645128674 |
| H | 1.717091730 | 5.829015305 | -5.203047356 |
| H | 0.120750535 | 7.481051089 | 1.073286549 |
| H | -0.546576519 | 8.384067033 | -0.296516346 |
| H | -1.287625554 | 6.859471264 | 0.208986591 |
| O | -1.466985369 | 3.739555126 | -1.264946943 |
| O | -0.073496231 | 1.960509322 | -1.347887016 |
| C | -1.493359708 | 3.748046427 | -2.717260556 |
| H | -0.486790143 | 3.939126676 | -3.094716564 |
| H | -1.862538799 | 2.792671870 | -3.095317565 |
| H | -2.168031863 | 4.560323661 | -2.982585389 |
| H | -3.394212059 | 3.479974753 | 2.022568367 |
| H | -2.737409354 | 4.246698132 | 0.556919510 |
| H | -2.396136247 | 4.918995277 | 2.163955497 |

==============================

16A-TS

==============================

| | | | |
|---|---|---|---|
| C | 1.597223377 | 3.541404796 | 12.058811401 |
| C | 0.991428347 | 4.409875489 | 11.136408474 |
| H | 0.836628910 | 5.451646873 | 11.403358103 |
| C | 0.600600124 | 3.966243900 | 9.871648296 |
| C | -0.061258187 | 4.899100548 | 8.886178482 |
| C | 0.810505404 | 2.619611668 | 9.544845776 |
| H | 0.500422538 | 2.258333876 | 8.566383248 |
| C | 1.402560754 | 1.727971204 | 10.443206924 |
| C | 1.630912701 | 0.285888816 | 10.056512575 |
| C | 1.795924476 | 2.202751136 | 11.704485802 |
| H | 2.248404026 | 1.512018859 | 12.407939751 |
| C | 2.784830836 | 2.892881850 | 14.677234622 |
| C | 3.910771672 | 2.183858773 | 14.232823812 |
| H | 4.335703745 | 2.398552402 | 13.255459603 |
| C | 4.516883470 | 1.225745875 | 15.048015856 |
| C | 5.759624798 | 0.498307327 | 14.598234902 |
| C | 3.991902297 | 1.009896317 | 16.328947407 |
| H | 4.478147766 | 0.286867178 | 16.980647731 |
| C | 2.884970786 | 1.717254044 | 16.804808574 |
| C | 2.407601862 | 1.548092162 | 18.226309288 |
| C | 2.276740321 | 2.649958162 | 15.957218275 |
| H | 1.435888670 | 3.235211611 | 16.321359641 |
| C | 3.568343260 | 5.252688418 | 13.230350614 |
| C | 3.948471337 | 5.399017571 | 11.868323467 |
| H | 3.407314166 | 4.869073150 | 11.096220452 |
| C | 5.005498412 | 6.196392775 | 11.509773579 |
| H | 5.288592414 | 6.288585049 | 10.464096600 |
| C | 5.745470677 | 6.901419490 | 12.487357625 |
| C | 6.835051840 | 7.738875239 | 12.130367582 |
| H | 7.088648444 | 7.843705545 | 11.078612040 |
| C | 7.563278286 | 8.394968035 | 13.094716221 |
| H | 8.402034156 | 9.025040021 | 12.812918388 |
| C | 7.219166723 | 8.248215725 | 14.460285081 |
| H | 7.793530561 | 8.773772728 | 15.217701143 |
| C | 6.162738936 | 7.450416212 | 14.836061825 |
| H | 5.909418218 | 7.352908036 | 15.885436875 |
| C | 5.395368244 | 6.746341555 | 13.866108511 |
| C | 4.296934113 | 5.897321743 | 14.224463295 |
| C | 4.029155513 | 5.662170332 | 15.679147606 |
| C | 4.982354604 | 4.849609773 | 16.389854959 |
| C | 6.105042438 | 4.256323474 | 15.744539295 |
| H | 6.248743056 | 4.406117454 | 14.681822361 |
| C | 7.010137698 | 3.496259213 | 16.449574000 |
| H | 7.865919217 | 3.067742915 | 15.935368422 |
| C | 6.838538238 | 3.267256144 | 17.834657718 |
| H | 7.561271947 | 2.664926315 | 18.377121939 |
| C | 5.760887934 | 3.815310194 | 18.489696696 |
| H | 5.619377493 | 3.655176908 | 19.555285717 |
| C | 4.819332413 | 4.615146257 | 17.792203995 |
| C | 3.727039769 | 5.221552811 | 18.454030732 |
| H | 3.615949233 | 5.088074391 | 19.527089294 |
| C | 2.824412198 | 5.988145630 | 17.764195964 |
| H | 2.027493219 | 6.459422139 | 18.317941517 |

| | | | |
|---|---|---|---|
| C | 2.937992408 | 6.207072510 | 16.361511619 |
| C | 0.780342376 | 8.165460918 | 16.754094261 |
| C | 0.849359788 | 9.559798185 | 16.620355824 |
| H | 1.291100439 | 10.005704127 | 15.737638570 |
| C | 0.353741581 | 10.409605964 | 17.616708114 |
| C | 0.416062718 | 11.908839086 | 17.434877052 |
| C | -0.224206876 | 9.845755186 | 18.759396555 |
| H | -0.602583596 | 10.498539454 | 19.542994762 |
| C | -0.351349802 | 8.458955753 | 18.903527816 |
| C | -1.068017252 | 7.850163501 | 20.083008062 |
| C | 0.149696279 | 7.634283296 | 17.889470648 |
| H | 0.017859841 | 6.561359054 | 17.987650265 |
| C | 2.252062027 | 8.156038964 | 14.252249921 |
| C | 3.248549001 | 9.068730546 | 14.637797857 |
| H | 3.588418086 | 9.095638527 | 15.669462491 |
| C | 3.817528657 | 9.937728819 | 13.706713529 |
| C | 4.884470168 | 10.929562285 | 14.103124457 |
| C | 3.397297750 | 9.852202364 | 12.370732992 |
| H | 3.855141483 | 10.508941241 | 11.633517419 |
| C | 2.425881407 | 8.938437019 | 11.957004796 |
| C | 2.056688380 | 8.787024462 | 10.500959199 |
| C | 1.840872691 | 8.107044233 | 12.920350068 |
| H | 1.084885930 | 7.388480293 | 12.620302087 |
| P | 2.077682755 | 4.252305856 | 13.681111819 |
| P | 1.507183901 | 7.032343153 | 15.485285315 |
| H | 0.990837546 | 8.567732681 | 10.374674435 |
| H | 2.287474439 | 9.687251744 | 9.925091178 |
| H | 2.618084944 | 7.954805498 | 10.057063873 |
| H | 4.600616741 | 11.948622659 | 13.816859739 |
| H | 5.068552566 | 10.917247280 | 15.180674417 |
| H | 5.828724485 | 10.696631649 | 13.600040773 |
| H | -0.506359134 | 7.008214595 | 20.502729326 |
| H | -1.230434116 | 8.581813956 | 20.879191625 |
| H | -2.042549477 | 7.465469631 | 19.760158031 |
| H | 0.501845540 | 12.426639757 | 18.394888427 |
| H | 1.266349290 | 12.203108674 | 16.811811423 |
| H | -0.492139502 | 12.280729956 | 16.943333985 |
| H | 5.769029936 | -0.539114647 | 14.946499474 |
| H | 5.857537419 | 0.495338709 | 13.508908597 |
| H | 6.648689367 | 0.990386308 | 15.010926269 |
| H | 1.339441874 | 1.766597814 | 18.324175958 |
| H | 2.585582283 | 0.534457073 | 18.597058746 |
| H | 2.945611473 | 2.241266779 | 18.885258763 |
| H | 1.901390260 | -0.325543252 | 10.921763565 |
| H | 0.736686675 | -0.149166995 | 9.598130356 |
| H | 2.442788394 | 0.203052121 | 9.323873764 |
| H | 0.368389560 | 4.789530108 | 7.885266265 |
| H | -1.132182945 | 4.678039299 | 8.802953292 |
| H | 0.043731233 | 5.945378982 | 9.186916413 |
| C | -1.632306038 | 6.070857599 | 13.662511691 |
| C | -1.544771223 | 5.680472098 | 15.819445534 |
| Rh | 0.174767601 | 5.285518281 | 14.721225980 |
| C | -1.274925966 | 3.406530947 | 15.153819866 |
| C | -1.610512637 | 3.596013039 | 13.836129865 |
| C | -2.376715653 | 4.808702247 | 13.310056983 |
| C | -2.491111351 | 4.465095944 | 11.808417483 |
| C | -1.590039525 | 2.557248539 | 12.737959890 |
| O | -2.500314216 | 3.042651752 | 11.751122557 |
| H | -2.228286690 | 6.933503403 | 13.929801257 |
| H | -0.879909391 | 6.356947276 | 12.919268177 |
| H | -0.886331766 | 2.436569595 | 15.461860423 |
| H | -0.588146350 | 2.412731077 | 12.322667198 |
| H | -1.954687167 | 1.587355656 | 13.096454236 |
| H | -3.415418540 | 4.827779248 | 11.352719288 |
| H | -1.634606334 | 4.878630390 | 11.258422040 |
| H | -3.374994063 | 4.800042924 | 13.769353244 |
| C | -1.671465604 | 4.367611118 | 16.195870155 |
| C | -2.154715479 | 3.836639786 | 17.520900710 |
| C | -2.232702760 | 6.790499353 | 16.517091492 |
| H | -1.895169452 | 4.508750463 | 18.338886687 |
| H | -3.248177612 | 3.757522884 | 17.513757382 |
| H | -1.745809295 | 2.838023692 | 17.705327499 |
| O | -2.157989797 | 7.955596684 | 15.844969170 |
| O | -2.860359612 | 6.658923199 | 17.556853477 |
| C | -2.879119997 | 9.061300397 | 16.422066319 |
| H | -2.493983741 | 9.286596588 | 17.416952111 |
| H | -2.707562678 | 9.899947818 | 15.747737103 |
| H | -3.944855040 | 8.827252078 | 16.487911928 |

==============================

16B-TS

==============================

| | | | |
|---|---|---|---|
| C | 2.006977500 | 3.624871294 | 12.023758081 |
| C | 1.604632124 | 4.508866797 | 11.007783754 |
| H | 1.426239823 | 5.553454170 | 11.245534473 |
| C | 1.422098122 | 4.072817705 | 9.693535942 |
| C | 0.953306752 | 5.012732865 | 8.610031079 |
| C | 1.637337848 | 2.718638543 | 9.407731518 |
| H | 1.493091310 | 2.364740626 | 8.388976167 |
| C | 2.018516190 | 1.808959338 | 10.397236256 |
| C | 2.263333673 | 0.357533797 | 10.056721326 |
| C | 2.197272713 | 2.274084101 | 11.708556015 |
| H | 2.489274147 | 1.568607435 | 12.478442369 |
| C | 2.811871563 | 2.866086488 | 14.710504622 |
| C | 4.034470749 | 2.257351729 | 14.389188327 |
| H | 4.604495447 | 2.608094442 | 13.532576366 |
| C | 4.534010487 | 1.210073978 | 15.165588282 |
| C | 5.862062264 | 0.567812708 | 14.846808867 |
| C | 3.797363015 | 0.802542151 | 16.286466863 |
| H | 4.192036903 | 0.003777998 | 16.911418607 |
| C | 2.583269955 | 1.400758028 | 16.635524052 |
| C | 1.854764535 | 1.006812366 | 17.896434707 |
| C | 2.089616185 | 2.428148706 | 15.823205831 |
| H | 1.153432488 | 2.918671469 | 16.082578751 |
| C | 3.621862215 | 5.449144830 | 13.625214098 |
| C | 4.038207842 | 5.952885710 | 12.361808240 |
| H | 3.626370844 | 5.537330419 | 11.453163361 |
| C | 4.985708366 | 6.940248495 | 12.260493987 |
| H | 5.295545025 | 7.297398447 | 11.281229771 |
| C | 5.588429394 | 7.489435709 | 13.415898463 |
| C | 6.565195988 | 8.515884900 | 13.327616550 |
| H | 6.827340579 | 8.901797880 | 12.345796939 |
| C | 7.178469911 | 9.001416885 | 14.458172588 |
| H | 7.933725253 | 9.778159501 | 14.380593012 |
| C | 6.827807030 | 8.482419071 | 15.727341419 |
| H | 7.317977221 | 8.864730189 | 16.618328632 |
| C | 5.869529879 | 7.501904228 | 15.845167668 |
| H | 5.604997181 | 7.127679688 | 16.826869002 |
| C | 5.221306921 | 6.967342594 | 14.695898977 |
| C | 4.232852597 | 5.929638144 | 14.782943900 |
| C | 3.953855995 | 5.347666422 | 16.133948908 |
| C | 4.972567361 | 4.524930263 | 16.723982908 |
| C | 6.186381253 | 4.215006523 | 16.047087787 |

| | | | |
|---|---|---|---|
| H | 6.349999975 | 4.596364857 | 15.046443438 |
| C | 7.155446100 | 3.443500487 | 16.646470004 |
| H | 8.079061889 | 3.232100583 | 16.114995088 |
| C | 6.959874052 | 2.918874971 | 17.946647491 |
| H | 7.733516115 | 2.310381683 | 18.405993636 |
| C | 5.792903697 | 3.183748232 | 18.623630935 |
| H | 5.631398759 | 2.789528623 | 19.623720519 |
| C | 4.783263082 | 3.992498763 | 18.039047825 |
| C | 3.598723866 | 4.321903664 | 18.736733063 |
| H | 3.463841931 | 3.957996777 | 19.752470685 |
| C | 2.632898949 | 5.106003620 | 18.158012221 |
| H | 1.753629792 | 5.349888158 | 18.736102237 |
| C | 2.781073399 | 5.616719250 | 16.837219976 |
| C | 0.312276353 | 6.928107793 | 17.505071975 |
| C | 0.164287822 | 8.201784739 | 18.055186845 |
| H | 0.686012340 | 9.050950643 | 17.626382815 |
| C | -0.681337502 | 8.413910432 | 19.157195199 |
| C | -0.857246299 | 9.808629145 | 19.709819540 |
| C | -1.362549238 | 7.324731998 | 19.702195616 |
| H | -2.017175557 | 7.479650286 | 20.556858277 |
| C | -1.231693969 | 6.031759609 | 19.169557235 |
| C | -2.017684735 | 4.882621886 | 19.752072729 |
| C | -0.398537753 | 5.851427716 | 18.067743601 |
| H | -0.309128503 | 4.860577394 | 17.629791765 |
| C | 2.126643646 | 8.131161463 | 15.503931940 |
| C | 2.895710520 | 8.904705878 | 16.387987616 |
| H | 3.024829872 | 8.585279657 | 17.418837465 |
| C | 3.520439669 | 10.073088337 | 15.948384334 |
| C | 4.374036769 | 10.901211569 | 16.876515180 |
| C | 3.387262388 | 10.436932054 | 14.600976620 |
| H | 3.891699627 | 11.333925589 | 14.247224095 |
| C | 2.648456028 | 9.671432614 | 13.696095389 |
| C | 2.597914573 | 10.026076355 | 12.229748724 |
| C | 2.006846457 | 8.520645789 | 14.167687609 |
| H | 1.451706105 | 7.897901530 | 13.476107675 |
| P | 2.181903814 | 4.273647553 | 13.733929782 |
| P | 1.379703983 | 6.561410904 | 16.053539730 |
| H | 1.638774731 | 9.749578606 | 11.779900962 |
| H | 2.755308314 | 11.095383480 | 12.064574376 |
| H | 3.382579122 | 9.485458367 | 11.685797349 |
| H | 4.169698832 | 11.970396868 | 16.759955091 |
| H | 4.211545901 | 10.634108918 | 17.924190787 |
| H | 5.435221285 | 10.744077477 | 16.651589839 |
| H | -1.617046904 | 3.917119524 | 19.426360465 |
| H | -2.007129025 | 4.904435190 | 20.846461213 |
| H | -3.060091464 | 4.940892488 | 19.420609732 |
| H | -1.322229446 | 9.795403348 | 20.699045357 |
| H | 0.102974885 | 10.328474987 | 19.791952291 |
| H | -1.495174119 | 10.410258826 | 19.051169120 |
| H | 5.849856461 | -0.505975803 | 15.057420264 |
| H | 6.138662394 | 0.708172494 | 13.797712517 |
| H | 6.652045136 | 1.015408658 | 15.460258454 |
| H | 0.770905706 | 1.119196466 | 17.789030931 |
| H | 2.065310594 | -0.027790217 | 18.182530892 |
| H | 2.169573618 | 1.652807271 | 18.725074867 |
| H | 2.263070613 | -0.271530404 | 10.951333654 |
| H | 1.502821657 | -0.025695234 | 9.369120747 |
| H | 3.236101052 | 0.232956772 | 9.565439270 |
| H | 1.464813788 | 4.815889038 | 7.662692373 |
| H | -0.120973044 | 4.875899897 | 8.440712696 |
| H | 1.124140440 | 6.060333410 | 8.876422929 |
| C | -1.049776056 | 6.651786948 | 13.148539066 |
| C | -1.680110093 | 6.055972352 | 15.159861846 |
| Rh | 0.157726695 | 5.332001149 | 14.484182405 |
| C | -1.766839930 | 3.848766237 | 14.206562265 |
| C | -1.582955895 | 4.249260636 | 12.907956085 |
| C | -1.918233096 | 5.659470474 | 12.413256391 |
| C | -1.679503412 | 5.526762007 | 10.894617746 |
| C | -1.412772536 | 3.354753711 | 11.702587131 |
| O | -1.829276900 | 4.144208582 | 10.584571324 |
| H | -1.504887186 | 7.603510549 | 13.406820737 |
| H | -0.090187183 | 6.829216804 | 12.653240038 |
| H | -1.727089832 | 2.791355138 | 14.454081049 |
| H | -0.386568291 | 2.997809699 | 11.580130290 |
| H | -2.072234869 | 2.480389004 | 11.765049413 |
| H | -2.408777242 | 6.082834303 | 10.299924840 |
| H | -0.676006288 | 5.884845994 | 10.632481599 |
| H | -2.981423159 | 5.844176342 | 12.616519505 |
| C | -2.237859401 | 4.804785930 | 15.217754402 |
| C | -3.199061473 | 4.394622643 | 16.277550918 |
| C | -2.191060060 | 7.305492096 | 15.796282754 |
| H | -2.436667535 | 7.139252553 | 16.846658145 |
| H | -1.477887818 | 8.126705839 | 15.712855545 |
| H | -3.125746360 | 7.599597475 | 15.303838452 |
| O | -3.810288162 | 5.158425181 | 17.002256049 |
| O | -3.278016985 | 3.048668749 | 16.369942720 |
| C | -4.174103595 | 2.545337599 | 17.381874956 |
| H | -4.179777857 | 1.463151103 | 17.254731863 |
| H | -3.811973084 | 2.812575335 | 18.377846927 |
| H | -5.176057373 | 2.958624741 | 17.244644405 |

==============================

18A-TS

==============================

| | | | |
|---|---|---|---|
| C | 2.152140011 | 7.491797124 | 11.655172369 |
| C | 1.030933251 | 8.330214829 | 11.642822510 |
| H | 0.247609868 | 8.182363973 | 12.380647542 |
| C | 0.899904127 | 9.358663827 | 10.702533783 |
| C | -0.306524342 | 10.268186170 | 10.715078293 |
| C | 1.914550645 | 9.525353686 | 9.754350411 |
| H | 1.815374963 | 10.307431913 | 9.003282939 |
| C | 3.048743424 | 8.704950114 | 9.737778443 |
| C | 4.126753720 | 8.910575551 | 8.698210261 |
| C | 3.157601581 | 7.690668958 | 10.694656210 |
| H | 4.032549929 | 7.051047157 | 10.678156492 |
| C | 3.580282750 | 5.091399838 | 12.129298796 |
| C | 4.936693844 | 5.213832483 | 12.435614163 |
| H | 5.259878465 | 5.861998691 | 13.241751213 |
| C | 5.898369175 | 4.519528770 | 11.687087365 |
| C | 7.356800036 | 4.601781057 | 12.068887647 |
| C | 5.469464214 | 3.759579459 | 10.595221465 |
| H | 6.210906259 | 3.245579033 | 9.985812056 |
| C | 4.114520142 | 3.654643249 | 10.242229424 |
| C | 3.714392340 | 2.910113271 | 8.989900902 |
| C | 3.169065042 | 4.305598896 | 11.038305412 |
| H | 2.113793186 | 4.237355167 | 10.782109764 |
| C | 2.839199476 | 6.904321540 | 14.484059959 |
| C | 2.553310300 | 8.293309251 | 14.669136574 |
| H | 2.295383790 | 8.895115697 | 13.806031041 |
| C | 2.593460635 | 8.873830500 | 15.909503857 |
| H | 2.391645706 | 9.935960843 | 16.022067475 |
| C | 2.863227494 | 8.097158969 | 17.065535220 |
| C | 2.814347277 | 8.653809035 | 18.369543027 |

| | | | |
|---|---|---|---|
| H | 2.569123747 | 9.706985591 | 18.479023244 |
| C | 3.061150476 | 7.872941067 | 19.476366659 |
| H | 3.013171598 | 8.307064407 | 20.471073465 |
| C | 3.374751927 | 6.501131643 | 19.322500746 |
| H | 3.560064155 | 5.889262946 | 20.200750163 |
| C | 3.448941879 | 5.936540720 | 18.068988980 |
| H | 3.683479267 | 4.883599846 | 17.962096864 |
| C | 3.189530246 | 6.711841270 | 16.904863264 |
| C | 3.234088832 | 6.130455707 | 15.590696739 |
| C | 3.900761622 | 4.787873449 | 15.457727220 |
| C | 5.331744751 | 4.772375930 | 15.631576325 |
| C | 6.095598065 | 5.953692838 | 15.852092006 |
| H | 5.598039772 | 6.915657487 | 15.889051268 |
| C | 7.461434697 | 5.896087079 | 16.020576863 |
| H | 8.020605553 | 6.811549541 | 16.191380221 |
| C | 8.142352335 | 4.657274601 | 15.979158991 |
| H | 9.218288090 | 4.625379221 | 16.123241298 |
| C | 7.437876862 | 3.497736303 | 15.750748415 |
| H | 7.948982506 | 2.539174935 | 15.710744856 |
| C | 6.030824639 | 3.525468141 | 15.565090964 |
| C | 5.301409173 | 2.335495141 | 15.317491704 |
| H | 5.836674060 | 1.391313916 | 15.267621029 |
| C | 3.943538891 | 2.369454428 | 15.152452021 |
| H | 3.404705144 | 1.448558190 | 14.964536120 |
| C | 3.228340100 | 3.597417042 | 15.223262021 |
| C | 0.955895466 | 2.133637857 | 14.150787125 |
| C | -0.137077505 | 1.322298869 | 14.481737054 |
| H | -0.675680828 | 1.479763066 | 15.411562997 |
| C | -0.533432039 | 0.267949838 | 13.647958522 |
| C | -1.701476214 | -0.610630063 | 14.030710769 |
| C | 0.198895235 | 0.028399446 | 12.482292935 |
| H | -0.090873255 | -0.795571663 | 11.832693566 |
| C | 1.309784587 | 0.808782229 | 12.137548228 |
| C | 2.092077351 | 0.510442953 | 10.881403956 |
| C | 1.669441610 | 1.871000701 | 12.971565118 |
| H | 2.526991582 | 2.480659200 | 12.707425381 |
| C | 1.014916247 | 3.062164153 | 16.891536232 |
| C | 1.268720496 | 1.740384562 | 17.300483852 |
| H | 1.628111218 | 1.007138798 | 16.586030766 |
| C | 1.047418833 | 1.344512694 | 18.618363065 |
| C | 1.318866248 | -0.073514548 | 19.064140536 |
| C | 0.571183926 | 2.296372591 | 19.535303332 |
| H | 0.387103551 | 1.990910544 | 20.563773258 |
| C | 0.331652649 | 3.619758281 | 19.164630817 |
| C | -0.163707092 | 4.653499047 | 20.146758787 |
| C | 0.565882088 | 3.990175918 | 17.830360562 |
| H | 0.397074966 | 5.015179423 | 17.536470912 |
| P | 2.293856708 | 6.130097590 | 12.891344737 |
| P | 1.389658786 | 3.599094206 | 15.171921304 |
| H | -1.103400081 | 5.099225689 | 19.802563048 |
| H | -0.327128024 | 4.223683807 | 21.138886351 |
| H | 0.563338236 | 5.468750110 | 20.244626132 |
| H | 0.455846979 | -0.495818010 | 19.590726758 |
| H | 1.550098688 | -0.725784929 | 18.217462973 |
| H | 2.167811928 | -0.111570666 | 19.757142090 |
| H | 3.082018436 | 0.971419221 | 10.913084044 |
| H | 2.219164562 | -0.568335320 | 10.741485201 |
| H | 1.572945253 | 0.891681924 | 9.992886529 |
| H | -2.139199015 | -1.098837021 | 13.155270303 |
| H | -1.384976239 | -1.400175700 | 14.722844718 |
| H | -2.488328773 | -0.036598073 | 14.530394165 |
| H | 8.010176191 | 4.348969866 | 11.228950376 |
| H | 7.621040450 | 5.603852331 | 12.423111858 |
| H | 7.576804037 | 3.908542319 | 12.890151801 |
| H | 2.628454162 | 2.839832539 | 8.889367543 |
| H | 4.097027776 | 3.427456549 | 8.102714332 |
| H | 4.126361122 | 1.895897778 | 8.971257712 |
| H | 4.920191078 | 8.163874866 | 8.787213697 |
| H | 3.714354795 | 8.845493667 | 7.685015253 |
| H | 4.585335988 | 9.901091787 | 8.796713296 |
| H | -0.613306407 | 10.537651359 | 9.699956394 |
| H | -1.160242862 | 9.796926019 | 11.211902518 |
| H | -0.089550961 | 11.202392534 | 11.247369750 |
| C | -1.009661207 | 6.952952151 | 13.927364829 |
| C | -0.973934647 | 6.197286682 | 15.835699263 |
| Rh | 0.427547130 | 5.676001018 | 14.318806063 |
| C | -2.097701696 | 4.273086242 | 14.798527778 |
| C | -1.326089127 | 4.763949370 | 13.582714517 |
| C | -1.905222234 | 6.111445421 | 13.096697974 |
| C | -1.642817581 | 5.998449818 | 11.565332452 |
| C | -1.215547547 | 3.887276604 | 12.331762484 |
| O | -1.869279730 | 4.623682251 | 11.294588471 |
| H | -1.280775586 | 7.968386255 | 14.225788004 |
| H | -3.149362979 | 4.087240992 | 14.534667579 |
| H | -0.166900649 | 3.689620228 | 12.072315836 |
| H | -1.723097353 | 2.923208224 | 12.442811427 |
| H | -2.338819020 | 6.586451459 | 10.963092701 |
| H | -0.612042280 | 6.288661298 | 11.323963812 |
| H | -2.985270275 | 6.237150323 | 13.263590798 |
| C | -1.974562188 | 5.277567609 | 15.917336469 |
| C | -2.823696187 | 5.104229920 | 17.135103835 |
| C | -0.613579360 | 7.289436359 | 16.800666798 |
| H | 0.329130149 | 7.056414165 | 17.308159552 |
| H | -0.425340865 | 8.224003479 | 16.264461618 |
| H | -1.384203679 | 7.445730815 | 17.556148729 |
| O | -2.797704182 | 5.799854055 | 18.135155481 |
| O | -3.622275234 | 4.026199627 | 17.010885339 |
| C | -4.441113612 | 3.727098982 | 18.159086953 |
| H | -4.991654883 | 2.824086227 | 17.897347061 |
| H | -3.813420770 | 3.559205004 | 19.038764803 |
| H | -5.126292185 | 4.552813608 | 18.364278462 |
| H | -1.706776238 | 3.313416005 | 15.150641664 |

==============================

18B-TS

==============================

| | | | |
|---|---|---|---|
| C | 2.058394700 | 5.864652974 | 11.125247797 |
| C | 1.423430530 | 7.093907117 | 10.885136486 |
| H | 0.997435342 | 7.651871755 | 11.715439068 |
| C | 1.315009168 | 7.613270549 | 9.590884232 |
| C | 0.671550662 | 8.959670086 | 9.352299858 |
| C | 1.828476807 | 6.862364466 | 8.526027580 |
| H | 1.733094635 | 7.245704929 | 7.512210445 |
| C | 2.450580970 | 5.626079701 | 8.731635206 |
| C | 2.997488829 | 4.839411998 | 7.563651573 |
| C | 2.564664822 | 5.137400316 | 10.040313822 |
| H | 3.037535821 | 4.174368164 | 10.199733566 |
| C | 2.689324335 | 3.518902428 | 12.782481731 |
| C | 3.960860611 | 3.156973829 | 12.321842627 |
| H | 4.671373898 | 3.924709518 | 12.026065773 |
| C | 4.348026407 | 1.812314748 | 12.276946105 |
| C | 5.743708194 | 1.428507662 | 11.850099203 |
| C | 3.432398851 | 0.841001341 | 12.696345259 |

| | | | |
|---|---|---|---|
| H | 3.724451435 | -0.206889832 | 12.665591906 |
| C | 2.158521267 | 1.177220278 | 13.170413762 |
| C | 1.217857687 | 0.109939916 | 13.677084779 |
| C | 1.799088921 | 2.525308399 | 13.209609330 |
| H | 0.828840686 | 2.807542994 | 13.603767116 |
| C | 3.563127173 | 6.204151017 | 13.656463717 |
| C | 3.813699779 | 7.510988870 | 13.146470848 |
| H | 3.358456297 | 7.814148613 | 12.211958962 |
| C | 4.646859554 | 8.385329178 | 13.792963698 |
| H | 4.844517460 | 9.366421973 | 13.368503437 |
| C | 5.254076353 | 8.029308026 | 15.020237484 |
| C | 6.083415648 | 8.943039254 | 15.723622569 |
| H | 6.260920678 | 9.923392019 | 15.288711403 |
| C | 6.648593110 | 8.598384927 | 16.929500792 |
| H | 7.281259057 | 9.302745929 | 17.461130027 |
| C | 6.399582931 | 7.317259745 | 17.476009608 |
| H | 6.834753533 | 7.048390183 | 18.434426273 |
| C | 5.612166984 | 6.405893247 | 16.809592364 |
| H | 5.428003462 | 5.436095668 | 17.253335527 |
| C | 5.019138242 | 6.722276566 | 15.554227458 |
| C | 4.191457227 | 5.785854195 | 14.834087687 |
| C | 4.139474886 | 4.385902884 | 15.389282269 |
| C | 5.349786766 | 3.610421004 | 15.303022741 |
| C | 6.488944246 | 4.055105142 | 14.573870899 |
| H | 6.446959269 | 5.000366702 | 14.046398463 |
| C | 7.644154242 | 3.307550230 | 14.532753981 |
| H | 8.500879325 | 3.672669196 | 13.973622072 |
| C | 7.727977902 | 2.068767609 | 15.212266299 |
| H | 8.648985002 | 1.494158118 | 15.179487987 |
| C | 6.638813668 | 1.595703889 | 15.905984151 |
| H | 6.684786644 | 0.642068929 | 16.425271564 |
| C | 5.434105152 | 2.344060669 | 15.966792064 |
| C | 4.313609230 | 1.880321030 | 16.694556557 |
| H | 4.381161479 | 0.933768858 | 17.224687776 |
| C | 3.155427173 | 2.611931305 | 16.738973588 |
| H | 2.321796376 | 2.234549754 | 17.315609018 |
| C | 3.044177312 | 3.866726507 | 16.077630095 |
| C | 0.318749511 | 3.772352252 | 17.167444898 |
| C | -0.346370070 | 4.302235587 | 18.272086140 |
| H | -0.150763343 | 5.316912242 | 18.599064425 |
| C | -1.329921660 | 3.554827515 | 18.940348388 |
| C | -2.078676057 | 4.186329039 | 20.088171742 |
| C | -1.610150764 | 2.263412392 | 18.493940507 |
| H | -2.368656749 | 1.676548478 | 19.008100035 |
| C | -0.957356395 | 1.706913362 | 17.380971901 |
| C | -1.291811535 | 0.306963567 | 16.922429230 |
| C | -0.006520365 | 2.478545232 | 16.715234448 |
| H | 0.494204665 | 2.065008496 | 15.843584697 |
| C | 1.910704562 | 6.264545166 | 17.124069904 |
| C | 2.443860392 | 6.134636391 | 18.414050298 |
| H | 2.480483114 | 5.156913298 | 18.887691243 |
| C | 2.945592617 | 7.247568488 | 19.092718280 |
| C | 3.573375463 | 7.111071485 | 20.458255209 |
| C | 2.914624455 | 8.493041793 | 18.451543958 |
| H | 3.321544096 | 9.360838487 | 18.966997167 |
| C | 2.402904277 | 8.650136532 | 17.160896597 |
| C | 2.454811951 | 9.989348720 | 16.466498195 |
| C | 1.893917095 | 7.521454126 | 16.509563616 |
| H | 1.510499506 | 7.634152963 | 15.497474155 |
| P | 2.180103970 | 5.267153230 | 12.864480275 |
| P | 1.440531807 | 4.791939130 | 16.142966215 |
| H | 1.805215836 | 10.010713150 | 15.586940233 |
| H | 2.152541451 | 10.801565722 | 17.135583681 |
| H | 3.476927003 | 10.201419123 | 16.132216972 |
| H | 3.267753835 | 7.924574635 | 21.124027477 |
| H | 3.307394470 | 6.163465259 | 20.934185445 |
| H | 4.666790879 | 7.149500645 | 20.378077251 |
| H | -0.792900456 | 0.059218233 | 15.981450638 |
| H | -0.982851746 | -0.434684484 | 17.668282721 |
| H | -2.370734074 | 0.184382420 | 16.776953884 |
| H | -2.899064441 | 3.553219656 | 20.436419099 |
| H | -1.413768140 | 4.375600452 | 20.938902416 |
| H | -2.490695101 | 5.152462598 | 19.776148039 |
| H | 5.769227813 | 0.424209381 | 11.417429283 |
| H | 6.147866292 | 2.129897374 | 11.113905416 |
| H | 6.416011924 | 1.438085385 | 12.715677501 |
| H | 0.196138723 | 0.489509799 | 13.774100849 |
| H | 1.193763973 | -0.757234158 | 13.009241721 |
| H | 1.535008439 | -0.248407322 | 14.664807657 |
| H | 3.290828284 | 3.828735948 | 7.860001171 |
| H | 2.257889234 | 4.755786218 | 6.760708773 |
| H | 3.880232158 | 5.332798251 | 7.140246339 |
| H | 0.186090156 | 9.004445739 | 8.372546973 |
| H | -0.079384457 | 9.188783220 | 10.115861275 |
| H | 1.421587330 | 9.760140485 | 9.380489251 |
| C | -1.091388734 | 6.804361691 | 13.268042335 |
| C | -1.490801624 | 5.994127980 | 15.424135715 |
| Rh | 0.217844556 | 5.753647830 | 14.133190452 |
| C | -2.091630258 | 3.949770225 | 14.267459744 |
| C | -1.292930018 | 4.590153590 | 13.147393407 |
| C | -1.988937591 | 5.854141721 | 12.585532263 |
| C | -1.872069933 | 5.662768341 | 11.045019011 |
| C | -0.973125018 | 3.756076538 | 11.897069454 |
| O | -1.864317812 | 4.246964973 | 10.896808147 |
| H | -1.261629120 | 7.880113051 | 13.354727014 |
| H | -3.027819498 | 3.541112031 | 13.851701355 |
| H | 0.064583308 | 3.869610337 | 11.562627049 |
| H | -1.175281372 | 2.689458602 | 12.038391799 |
| H | -2.731124619 | 6.060606339 | 10.501718287 |
| H | -0.948727761 | 6.105706437 | 10.649664508 |
| H | -3.047651426 | 5.950015752 | 12.861396878 |
| C | -2.358072341 | 4.958734369 | 15.360353602 |
| C | -3.517397044 | 4.693623025 | 16.275849685 |
| C | -1.544988262 | 7.073817666 | 16.428157016 |
| H | -3.664051333 | 5.494702514 | 16.997575774 |
| H | -4.434562183 | 4.544734676 | 15.692361625 |
| H | -3.333468241 | 3.765394305 | 16.830546938 |
| O | -1.087844723 | 8.250654397 | 15.926960143 |
| O | -1.918619422 | 6.958340998 | 17.583312807 |
| C | -1.062486013 | 9.343837624 | 16.866028343 |
| H | -0.394444779 | 9.108733098 | 17.698201396 |
| H | -0.688617073 | 10.201720336 | 16.306975455 |
| H | -2.065073259 | 9.542160585 | 17.252879997 |
| H | -1.554330770 | 3.101346269 | 14.714011380 |

==============================

17

==============================

| | | | |
|---|---|---|---|
| C | 1.421953956 | 5.229945372 | 10.695807516 |
| C | 0.993466477 | 6.534331315 | 10.417090664 |
| H | 0.867335900 | 7.244917164 | 11.229669217 |
| C | 0.740694949 | 6.951044407 | 9.101255039 |
| C | 0.311859176 | 8.371084265 | 8.818766526 |

| | | | |
|---|---|---|---|
| C | 0.882912486 | 6.014942338 | 8.074030159 |
| H | 0.683019871 | 6.321617972 | 7.049462326 |
| C | 1.272503204 | 4.692788445 | 8.324174201 |
| C | 1.404661450 | 3.707787571 | 7.186289375 |
| C | 1.546829814 | 4.312195860 | 9.639849405 |
| H | 1.867930360 | 3.295828356 | 9.834705300 |
| C | 2.098658362 | 2.946276025 | 12.295788658 |
| C | 3.285429235 | 2.366418828 | 11.846169671 |
| H | 4.146017139 | 2.989609587 | 11.621078698 |
| C | 3.386006404 | 0.976008538 | 11.696196551 |
| C | 4.690986120 | 0.360106754 | 11.253882925 |
| C | 2.263563694 | 0.193895505 | 11.979698581 |
| H | 2.329069003 | -0.885643303 | 11.859698332 |
| C | 1.050726784 | 0.756098891 | 12.404473889 |
| C | -0.141824922 | -0.129443095 | 12.676653783 |
| C | 0.982248048 | 2.140702062 | 12.565867967 |
| H | 0.056923800 | 2.604219424 | 12.900675527 |
| C | 3.477007036 | 5.606704251 | 12.776617597 |
| C | 3.717567356 | 6.834450319 | 12.097173865 |
| H | 3.138899508 | 7.081427955 | 11.217408820 |
| C | 4.686250583 | 7.709129594 | 12.517534660 |
| H | 4.858907674 | 8.632224180 | 11.971150333 |
| C | 5.466399828 | 7.429830172 | 13.663580128 |
| C | 6.433118979 | 8.347939635 | 14.149993883 |
| H | 6.570799218 | 9.288226187 | 13.622331950 |
| C | 7.179083154 | 8.054627610 | 15.267603728 |
| H | 7.913918425 | 8.764390007 | 15.635942195 |
| C | 6.986430382 | 6.825562272 | 15.941817757 |
| H | 7.566866515 | 6.605775748 | 16.833068823 |
| C | 6.070287030 | 5.908489068 | 15.483214185 |
| H | 5.924275705 | 4.980186135 | 16.020449605 |
| C | 5.278628458 | 6.177009601 | 14.331778212 |
| C | 4.296587492 | 5.246442947 | 13.848694552 |
| C | 4.260058779 | 3.881968351 | 14.468149530 |
| C | 5.376545820 | 3.014007602 | 14.204204533 |
| C | 6.444130172 | 3.383439675 | 13.336288928 |
| H | 6.414901343 | 4.341873032 | 12.833372663 |
| C | 7.516286442 | 2.546248545 | 13.129145530 |
| H | 8.318946966 | 2.855209760 | 12.465853073 |
| C | 7.584719511 | 1.288885954 | 13.773926113 |
| H | 8.442357688 | 0.643057702 | 13.610976561 |
| C | 6.557372232 | 0.885360262 | 14.594424064 |
| H | 6.589173520 | -0.083886686 | 15.083804430 |
| C | 5.433940324 | 1.723464634 | 14.820478979 |
| C | 4.363662600 | 1.319489997 | 15.651528807 |
| H | 4.400938905 | 0.341495608 | 16.124311526 |
| C | 3.292353911 | 2.146334393 | 15.870526892 |
| H | 2.497350127 | 1.807428523 | 16.519692595 |
| C | 3.223606550 | 3.441513663 | 15.284070098 |
| C | 0.792404154 | 3.512493980 | 16.825829635 |
| C | 0.584601917 | 3.785476472 | 18.177400547 |
| H | 1.025826243 | 4.661124225 | 18.636019849 |
| C | -0.242958803 | 2.959699649 | 18.954819256 |
| C | -0.485645298 | 3.304771373 | 20.404528154 |
| C | -0.833752206 | 1.849278010 | 18.354489132 |
| H | -1.473351862 | 1.201005910 | 18.949865319 |
| C | -0.633718824 | 1.546437885 | 16.997823927 |
| C | -1.269057015 | 0.316843627 | 16.393939415 |
| C | 0.168685227 | 2.394992780 | 16.239005046 |
| H | 0.330246338 | 2.179471176 | 15.186949899 |
| C | 2.708738439 | 5.765683040 | 16.790104656 |
| C | 3.427824860 | 5.312446355 | 17.910576988 |
| H | 3.407032638 | 4.259766755 | 18.178586082 |
| C | 4.178571598 | 6.200141768 | 18.679357586 |
| C | 4.954907686 | 5.730828862 | 19.887455551 |
| C | 4.220413810 | 7.549831924 | 18.292126364 |
| H | 4.810916601 | 8.247685934 | 18.882653216 |
| C | 3.548807285 | 8.015615608 | 17.161885292 |
| C | 3.647529819 | 9.453300160 | 16.713876353 |
| C | 2.784461531 | 7.106281968 | 16.416487704 |
| H | 2.254008807 | 7.449991091 | 15.533898075 |
| P | 1.870824145 | 4.749603789 | 12.419381838 |
| P | 1.814804592 | 4.568937581 | 15.730952571 |
| H | 2.674195211 | 9.827273694 | 16.378125578 |
| H | 4.008985966 | 10.104617572 | 17.514649569 |
| H | 4.344910468 | 9.540806268 | 15.872089180 |
| H | 4.680564820 | 6.301424595 | 20.781840345 |
| H | 4.778243544 | 4.672070014 | 20.096042880 |
| H | 6.032348391 | 5.867929046 | 19.737500662 |
| H | -1.277527263 | 0.362426232 | 15.302818077 |
| H | -0.721605558 | -0.588734626 | 16.683331998 |
| H | -2.300912120 | 0.191712473 | 16.737827113 |
| H | -1.170293494 | 2.598153328 | 20.881102892 |
| H | 0.451201803 | 3.302474653 | 20.973757444 |
| H | -0.915326151 | 4.309054727 | 20.491838155 |
| H | 4.578709074 | -0.701826258 | 11.018794213 |
| H | 5.086909766 | 0.865464207 | 10.366264529 |
| H | 5.444891111 | 0.456318680 | 12.042271258 |
| H | -1.039270143 | 0.457674951 | 12.892153504 |
| H | -0.361386663 | -0.771115397 | 11.816435341 |
| H | 0.043736067 | -0.790261270 | 13.531324662 |
| H | 1.745217042 | 2.729627794 | 7.536626172 |
| H | 0.445721667 | 3.567173677 | 6.674634917 |
| H | 2.119593082 | 4.066023318 | 6.437026628 |
| H | 0.250717609 | 8.563592282 | 7.744746040 |
| H | -0.667678801 | 8.575331311 | 9.263042364 |
| H | 1.022043516 | 9.087438885 | 9.247569020 |
| C | -0.731475029 | 8.157395164 | 14.560066135 |
| C | -0.847054207 | 6.893477807 | 15.436051848 |
| Rh | 0.466092066 | 5.523353815 | 14.121921601 |
| C | -1.841433145 | 5.656814674 | 13.560907992 |
| C | -1.854394957 | 6.776403222 | 12.790414071 |
| C | -1.709676597 | 8.145746650 | 13.391194024 |
| C | -1.373856024 | 8.983440575 | 12.150401795 |
| C | -2.181410430 | 6.944672367 | 11.335655837 |
| O | -2.090673444 | 8.353547346 | 11.082977991 |
| H | -0.896870396 | 9.037356374 | 15.185296970 |
| H | 0.296166970 | 8.253422873 | 14.177299293 |
| H | -2.143330692 | 4.693359321 | 13.157673379 |
| H | -1.493499361 | 6.380418590 | 10.693971360 |
| H | -3.205032962 | 6.617581065 | 11.110041522 |
| H | -1.707632025 | 10.022085805 | 12.208767141 |
| H | -0.288461301 | 8.975058384 | 11.958863893 |
| H | -2.710777911 | 8.440061539 | 13.742321000 |
| C | -1.550509744 | 5.741537460 | 14.997964718 |
| C | -2.188905002 | 4.684768667 | 15.861419486 |
| C | -0.507042376 | 7.081927381 | 16.882930940 |
| H | -1.706827601 | 4.574727906 | 16.825157944 |
| H | -3.234934641 | 4.973584500 | 16.031952775 |
| H | -2.189520946 | 3.723664637 | 15.341777839 |
| O | 0.091138132 | 8.275830381 | 17.095679465 |
| O | -0.720384277 | 6.298913046 | 17.790112172 |

| | | | |
|---|---|---|---|
| C | 0.467904956 | 8.539132447 | 18.461755181 |
| H | -0.405375866 | 8.483965225 | 19.115893923 |
| H | 0.886641395 | 9.545097314 | 18.457427194 |
| H | 1.217966017 | 7.816996224 | 18.791077426 |

==============================

19

==============================

| | | | |
|---|---|---|---|
| C | 1.146564083 | 4.937365648 | 10.581213116 |
| C | 0.453148319 | 6.110372915 | 10.266696668 |
| H | 0.250093024 | 6.827649758 | 11.049676284 |
| C | 0.015070590 | 6.371154537 | 8.963159155 |
| C | -0.712499509 | 7.656747077 | 8.645495736 |
| C | 0.277396135 | 5.418050319 | 7.975644657 |
| H | -0.073038647 | 5.595772185 | 6.960697714 |
| C | 0.972159741 | 4.233552126 | 8.257378346 |
| C | 1.236335156 | 3.226828527 | 7.162137266 |
| C | 1.402786413 | 4.001568475 | 9.566482270 |
| H | 1.923514157 | 3.077667804 | 9.792879332 |
| C | 1.888577503 | 2.815804708 | 12.305578467 |
| C | 3.101266013 | 2.197672291 | 11.992907568 |
| H | 3.996781670 | 2.789678477 | 11.841333754 |
| C | 3.174860918 | 0.803735444 | 11.861419433 |
| C | 4.507673076 | 0.145881396 | 11.593174985 |
| C | 1.997075448 | 0.058020903 | 11.986685820 |
| H | 2.039348378 | -1.021725988 | 11.855655090 |
| C | 0.759368511 | 0.663006810 | 12.252212507 |
| C | -0.516836390 | -0.145812000 | 12.275089373 |
| C | 0.721006112 | 2.047361923 | 12.438410388 |
| H | -0.233097392 | 2.523011488 | 12.649234062 |
| C | 3.144563618 | 5.612850058 | 12.700459538 |
| C | 3.225271542 | 6.877671443 | 12.039506169 |
| H | 2.592570836 | 7.065528385 | 11.180943080 |
| C | 4.088282093 | 7.852690944 | 12.461053272 |
| H | 4.143891103 | 8.797390374 | 11.927593067 |
| C | 4.912818722 | 7.653966019 | 13.596798585 |
| C | 5.781474083 | 8.671227821 | 14.067846453 |
| H | 5.799085692 | 9.624573227 | 13.546571229 |
| C | 6.586573066 | 8.454892208 | 15.162537846 |
| H | 7.251198494 | 9.238024037 | 15.515430388 |
| C | 6.542186692 | 7.211211578 | 15.836417648 |
| H | 7.164954112 | 7.051535710 | 16.711716354 |
| C | 5.712827640 | 6.203945481 | 15.398486469 |
| H | 5.683506051 | 5.262686965 | 15.934386624 |
| C | 4.875879863 | 6.387952330 | 14.261255335 |
| C | 4.005719138 | 5.346881815 | 13.777878912 |
| C | 4.236928254 | 3.984230670 | 14.366045814 |
| C | 5.455284665 | 3.323560866 | 13.974142563 |
| C | 6.292066311 | 3.823935814 | 12.937140066 |
| H | 6.015488775 | 4.737168837 | 12.422573174 |
| C | 7.440963518 | 3.159484637 | 12.570286121 |
| H | 8.061052182 | 3.557720126 | 11.772417692 |
| C | 7.824094403 | 1.966527549 | 13.226051879 |
| H | 8.738244641 | 1.458470826 | 12.933738301 |
| C | 7.036300202 | 1.452735763 | 14.230483166 |
| H | 7.319215400 | 0.535842559 | 14.739889534 |
| C | 5.838237431 | 2.105748814 | 14.621099485 |
| C | 5.014605194 | 1.591182496 | 15.653598080 |
| H | 5.323936102 | 0.689595985 | 16.176009621 |
| C | 3.843359045 | 2.215909855 | 15.991612446 |
| H | 3.231866805 | 1.801362435 | 16.783763778 |
| C | 3.422218754 | 3.403611737 | 15.327436418 |
| C | 0.837768190 | 2.878935777 | 16.537377682 |
| C | -0.032105283 | 3.126254628 | 17.605459266 |
| H | -0.017935258 | 4.089020101 | 18.110289426 |
| C | -0.911891797 | 2.132372453 | 18.062167611 |
| C | -1.831434399 | 2.414656943 | 19.227462052 |
| C | -0.895212103 | 0.885129067 | 17.434438985 |
| H | -1.564438889 | 0.103595539 | 17.788323019 |
| C | -0.030042456 | 0.607712397 | 16.365472442 |
| C | -0.008276103 | -0.764193600 | 15.735216547 |
| C | 0.816036494 | 1.619408186 | 15.910286748 |
| H | 1.461860140 | 1.420366854 | 15.061884329 |
| C | 2.415973742 | 5.260334062 | 17.223640482 |
| C | 2.683204313 | 4.699775562 | 18.483214205 |
| H | 2.461177453 | 3.653464550 | 18.672411602 |
| C | 3.227765270 | 5.476520169 | 19.506968934 |
| C | 3.525508829 | 4.880383133 | 20.862715820 |
| C | 3.523449170 | 6.822937459 | 19.243752381 |
| H | 3.953201294 | 7.431867810 | 20.037043901 |
| C | 3.293166583 | 7.401395955 | 17.993008112 |
| C | 3.661999921 | 8.835993707 | 17.699474765 |
| C | 2.722215240 | 6.604924145 | 16.988634478 |
| H | 2.540553640 | 7.049229527 | 16.014457349 |
| P | 1.633119596 | 4.616886321 | 12.322897785 |
| P | 1.845020689 | 4.224943983 | 15.816286641 |
| H | 2.862886793 | 9.341614609 | 17.149316608 |
| H | 3.865736027 | 9.395015427 | 18.617449089 |
| H | 4.561633670 | 8.873901968 | 17.074993805 |
| H | 3.225494607 | 5.556626928 | 21.669936001 |
| H | 3.008158936 | 3.927787601 | 21.007514667 |
| H | 4.600284885 | 4.695946888 | 20.979929463 |
| H | 0.714164870 | -0.813774855 | 14.916856663 |
| H | 0.262188583 | -1.527771139 | 16.473548104 |
| H | -0.993021954 | -1.033402349 | 15.337655922 |
| H | -2.556979825 | 1.609992637 | 19.372818488 |
| H | -1.261911655 | 2.522011233 | 20.158289875 |
| H | -2.386207855 | 3.348028424 | 19.080219264 |
| H | 4.391523102 | -0.898417801 | 11.290034687 |
| H | 5.057906931 | 0.671886970 | 10.805556661 |
| H | 5.138241875 | 0.173297452 | 12.489796983 |
| H | -1.336377234 | 0.419944825 | 12.727343447 |
| H | -0.820115632 | -0.404957716 | 11.253200816 |
| H | -0.395258215 | -1.085956516 | 12.820917706 |
| H | 1.728691737 | 2.330240165 | 7.548607501 |
| H | 0.302837618 | 2.917539913 | 6.678611045 |
| H | 1.877393956 | 3.653551589 | 6.382089707 |
| H | -1.331641380 | 7.557795950 | 7.749569811 |
| H | -1.361531634 | 7.965410183 | 9.472130208 |
| H | -0.004018197 | 8.474783241 | 8.466350168 |
| C | -1.780371915 | 7.010781580 | 12.908438434 |
| C | -0.509566008 | 7.414184395 | 13.661392871 |
| Rh | 0.645868815 | 5.618127989 | 14.210598341 |
| C | -0.994540915 | 5.970438581 | 15.568554503 |
| C | -1.412560581 | 5.096413250 | 14.503040801 |
| C | -2.302354725 | 5.662878950 | 13.409371548 |
| C | -2.355667265 | 4.477115734 | 12.443361353 |
| C | -1.814147589 | 3.647254147 | 14.588880123 |
| O | -2.302072488 | 3.305778706 | 13.281153974 |
| H | -2.537285586 | 7.799259082 | 13.027594338 |
| H | -1.565554213 | 6.959546327 | 11.838612641 |
| H | -0.968507999 | 5.695581474 | 16.615262044 |
| H | -1.001283970 | 2.974657831 | 14.858464401 |

H -2.619101243 3.518363068 15.331765333
H -3.276048887 4.435414019 11.853310518
H -1.495726437 4.493538126 11.759831095
H -3.299969640 5.775129583 13.861709423
C -0.531979107 7.231957801 15.114287143
C 0.028747264 8.208859261 16.103553490
C 0.216596355 8.615187218 13.088905505
H 1.256263886 8.666912484 13.415807482
H 0.193993629 8.590053017 11.996281988
H -0.262256707 9.550066298 13.407997587
O 0.812902323 9.100491165 15.845114894
O -0.477112621 7.977197371 17.328381939
C -0.017063387 8.840128970 18.391052544
H -0.713044480 8.683666460 19.214901597
H 0.993974143 8.550964564 18.685035621
H -0.024447175 9.882884409 18.067119124
==============================
20A
==============================
Rh 0.446301916 1.559407960 0.819480790
P 2.668060646 1.786374815 1.309559955
C 0.807695002 -0.303533411 0.210066488
C 0.773064714 2.020145329 -1.182427040
C 0.745077247 -0.463235940 -1.113619819
C 0.455700982 0.747244566 -1.968429709
C 1.315423020 0.402472490 -3.203528789
C 1.123381115 -1.597202498 -2.045439592
O 1.141457795 -1.006790077 -3.349803974
H 0.104050942 2.860262198 -1.393190800
H 1.810228692 2.323765492 -1.330834387
H 1.095608158 -1.069332960 0.931813606
H 2.114319608 -1.996960548 -1.779080068
H 0.409910572 -2.429022397 -2.059563246
H 0.990412964 0.877171621 -4.132398172
H 2.370663851 0.662048690 -3.013388167
H -0.601318984 0.740970675 -2.269883063
C -2.219142753 1.061948665 0.292409954
C -1.860136304 2.100833182 0.831954263
C -2.648833444 -0.169421133 -0.341200288
C -1.772760925 3.400547768 1.486270248
H -1.784175045 -0.821666326 -0.510593574
H -3.368433905 -0.690961444 0.299069400
H -3.130024700 0.044686795 -1.301466283
C 3.051255593 3.557273388 1.584308091
C 3.765321967 3.972250676 2.719733143
H 4.097634616 3.244735871 3.452898023
C 4.052566028 5.325222305 2.911298328
H 4.604929668 5.635505874 3.793111620
C 3.638602540 6.274177883 1.974121368
H 3.866723366 7.324792604 2.125735883
C 2.930479756 5.866966136 0.840180668
H 2.606151209 6.599933167 0.107496659
C 2.630008472 4.518858052 0.647931301
H 2.062186234 4.221210484 -0.226948254
C 3.028878618 0.934729102 2.879766761
C 4.273127866 0.341722728 3.144298448
H 5.055354097 0.350991628 2.392553783
C 4.507500165 -0.261880030 4.380037394
H 5.472041187 -0.718942067 4.579496613
C 3.509578756 -0.277321809 5.356250797
H 3.696812885 -0.750027718 6.315673288
C 2.271390169 0.317796682 5.102165253
H 1.496161917 0.311396646 5.862232035
C 2.031659759 0.922765024 3.870208919
H 1.071227034 1.402474656 3.689109927
C 3.859739803 1.167916894 0.073530042
C 4.535420841 2.043638397 -0.788722848
H 4.431478634 3.117535246 -0.676528768
C 5.358843209 1.537292287 -1.796664762
H 5.877665896 2.223274519 -2.459324760
C 5.521343556 0.160305196 -1.946444512
H 6.162806719 -0.230218529 -2.730537290
C 4.863942059 -0.717057741 -1.079378970
H 4.997416338 -1.789590597 -1.183902032
C 4.032003718 -0.219735603 -0.079684782
H 3.525433383 -0.910217023 0.585243576
O -2.676953949 4.154759445 1.720215873
O -0.460915602 3.624882587 1.804633064
C -0.166041559 4.862394002 2.511642717
H 0.878894362 4.787711269 2.802736490
H -0.322179152 5.710753379 1.843229278
H -0.821013560 4.948074683 3.379976694
==============================
20B
==============================
Rh 0.450924351 1.506695302 0.833662786
P 2.632416646 1.518326231 1.516718528
C 0.651047519 -0.233034751 -0.102628482
C 1.033259427 2.312018128 -1.001165675
C 0.642885240 -0.115168434 -1.427295747
C 0.585716544 1.267903845 -2.031961299
C 1.451832856 1.035434609 -3.288336009
C 0.882568962 -1.096516498 -2.557200606
O 1.087596816 -0.278101364 -3.713987804
H 0.543636381 3.284735435 -1.117388707
H 2.113807734 2.461935260 -1.043519044
H 0.779606753 -1.134669763 0.495140728
H 1.766600883 -1.721229929 -2.351602614
H 0.033666863 -1.760937052 -2.754609889
H 1.249702686 1.721495700 -4.114648223
H 2.521095824 1.092508278 -3.024070892
H -0.444467983 1.467683763 -2.356829931
C -2.067355601 2.300190966 0.134768793
C -1.899186818 1.514951730 1.057978615
C -2.365425748 3.242183207 -0.932835428
C -2.090235295 0.537099829 2.123935752
H -1.550393655 3.956807668 -1.082683628
H -2.539715806 2.710636918 -1.874359011
H -3.273640691 3.801391003 -0.681624290
C 2.965991559 3.173713620 2.220649391
C 3.835129312 3.330920285 3.313967723
H 4.299797775 2.464419018 3.773054417
C 4.105192009 4.603352328 3.814548347
H 4.779823627 4.716167302 4.658171512
C 3.512817749 5.727895499 3.234844141
H 3.724062372 6.716841372 3.630306034
C 2.652616988 5.580435743 2.145181128
H 2.195555503 6.453219356 1.688534442
C 2.380807259 4.310470424 1.636675421
H 1.726297692 4.210904143 0.776364922
C 2.879406439 0.356733104 2.904032831
C 3.736463886 -0.750045535 2.826422747

| | | | |
|---|---|---|---|
| H | 4.306910443 | -0.942908781 | 1.924543364 |
| C | 3.879798183 | -1.599105523 | 3.925434391 |
| H | 4.549236346 | -2.451565821 | 3.859713822 |
| C | 3.181290733 | -1.346819075 | 5.106726080 |
| H | 3.304044329 | -2.004664610 | 5.961936249 |
| C | 2.330157399 | -0.240787096 | 5.191739502 |
| H | 1.791118102 | -0.035603216 | 6.112107146 |
| C | 2.171399985 | 0.602805126 | 4.094496001 |
| H | 1.511002862 | 1.462631757 | 4.167998848 |
| C | 3.943911206 | 1.205664289 | 0.286341511 |
| C | 4.951557494 | 2.156675445 | 0.059911029 |
| H | 4.972281147 | 3.081431789 | 0.627003710 |
| C | 5.934804658 | 1.917313267 | -0.901687440 |
| H | 6.709824568 | 2.659330937 | -1.071617071 |
| C | 5.923568845 | 0.733425156 | -1.640103883 |
| H | 6.689039885 | 0.551660901 | -2.388585240 |
| C | 4.924037293 | -0.218180145 | -1.416796513 |
| H | 4.909680352 | -1.140394986 | -1.990576562 |
| C | 3.935127483 | 0.015883387 | -0.463909476 |
| H | 3.151565723 | -0.717798536 | -0.316568314 |
| O | -3.147328669 | 0.094674678 | 2.487360798 |
| O | -0.875951835 | 0.184107539 | 2.623862210 |
| C | -0.890816662 | -0.899854603 | 3.593650237 |
| H | 0.152113228 | -1.159867056 | 3.753469834 |
| H | -1.354244852 | -0.555769911 | 4.520787857 |
| H | -1.457171088 | -1.741578002 | 3.191405810 |

==============================

20A-TS1

==============================

| | | | |
|---|---|---|---|
| C | 0.163677715 | 0.112595736 | -0.120738196 |
| C | -0.314576304 | 0.443682054 | 1.864561668 |
| Rh | 1.933422712 | 0.050330533 | 0.896419898 |
| P | 3.181486763 | -1.133046408 | -0.761345796 |
| C | 2.275291028 | 1.887385488 | -0.008683892 |
| C | 0.004929830 | 1.305646940 | -0.741471715 |
| C | 0.906809359 | 2.449003929 | -0.372061511 |
| C | 0.783780057 | 3.325453814 | -1.645506736 |
| C | -0.839750879 | 1.732160673 | -1.915677266 |
| O | -0.562107624 | 3.119557769 | -2.071898681 |
| H | 2.813472524 | 2.465858056 | 0.748892166 |
| H | 2.891393112 | 1.733238600 | -0.895350481 |
| H | -0.362085417 | -0.796654519 | -0.405890826 |
| H | -0.547383741 | 1.158103525 | -2.810821694 |
| H | -1.915600623 | 1.598158222 | -1.755137868 |
| H | 0.923737858 | 4.392730885 | -1.465781044 |
| H | 1.501383603 | 2.985535568 | -2.410645571 |
| H | 0.465594152 | 2.984489417 | 0.480861288 |
| C | 0.746938892 | 0.893276936 | 2.400374621 |
| C | -1.699725655 | -0.051145754 | 1.957637671 |
| C | 1.305412626 | 1.538810655 | 3.595516194 |
| H | -2.366063263 | 0.503083399 | 1.291001751 |
| H | -1.764427954 | -1.115217694 | 1.709361573 |
| H | -2.028480472 | 0.087300538 | 2.993930089 |
| O | 0.694128346 | 2.267129254 | 4.340487616 |
| O | 2.613830810 | 1.221059933 | 3.712058543 |
| C | 3.293255226 | 1.791017729 | 4.858140542 |
| H | 4.317050179 | 1.424634391 | 4.799057872 |
| H | 3.264672349 | 2.881968854 | 4.808258908 |
| H | 2.815414197 | 1.459506071 | 5.782492927 |
| C | 4.997686877 | -0.955035066 | -0.643644057 |
| C | 5.540248258 | 0.191197735 | -0.040617485 |
| H | 4.886451994 | 0.954913717 | 0.371667425 |
| C | 6.923728191 | 0.362240526 | 0.028072764 |
| H | 7.335070069 | 1.252833782 | 0.493868565 |
| C | 7.774967157 | -0.611484607 | -0.497659090 |
| H | 8.851307125 | -0.480401567 | -0.441626255 |
| C | 7.240562706 | -1.758368972 | -1.091518379 |
| H | 7.900706033 | -2.519942348 | -1.496039021 |
| C | 5.859709459 | -1.932740204 | -1.165731593 |
| H | 5.453600843 | -2.830451904 | -1.621920221 |
| C | 2.845813633 | -2.925051201 | -0.658331209 |
| C | 2.675704758 | -3.480871526 | 0.621038088 |
| H | 2.737459359 | -2.847815016 | 1.504863670 |
| C | 2.447494280 | -4.847440475 | 0.772612344 |
| H | 2.319949766 | -5.268117567 | 1.765577868 |
| C | 2.384600827 | -5.671695494 | -0.353271726 |
| H | 2.203586816 | -6.736041111 | -0.237084137 |
| C | 2.556266684 | -5.127912032 | -1.627878416 |
| H | 2.512369871 | -5.768923940 | -2.503431687 |
| C | 2.788225472 | -3.760936191 | -1.784452614 |
| H | 2.920379630 | -3.348010968 | -2.779427266 |
| C | 2.721536909 | -0.607305350 | -2.450931122 |
| C | 3.518432068 | 0.299574536 | -3.165370331 |
| H | 4.484320724 | 0.603158404 | -2.772473295 |
| C | 3.080385026 | 0.805515988 | -4.391789735 |
| H | 3.707504104 | 1.503165110 | -4.938412090 |
| C | 1.850911437 | 0.408329030 | -4.919474473 |
| H | 1.515633129 | 0.798727637 | -5.875234666 |
| C | 1.056832369 | -0.503120146 | -4.216839707 |
| H | 0.106942190 | -0.830435283 | -4.630100350 |
| C | 1.481636200 | -0.999383737 | -2.985084308 |
| H | 0.858520876 | -1.709667798 | -2.449601828 |

==============================

20A-TS2

==============================

| | | | |
|---|---|---|---|
| C | -0.161487218 | -0.066312725 | -0.040574695 |
| C | 0.015214855 | 0.046617195 | 2.225481198 |
| Rh | 1.823487450 | 0.040044079 | 1.065464314 |
| P | 4.113534419 | 0.412300465 | 1.359223506 |
| C | 2.116020476 | -1.776149014 | 0.348544050 |
| C | 1.055506459 | -2.244390776 | -0.318721512 |
| C | -0.271936149 | -1.526665215 | -0.351705939 |
| C | -0.749724080 | -1.925674565 | -1.775269365 |
| C | 0.959383434 | -3.400864779 | -1.304421770 |
| O | -0.341708999 | -3.283643801 | -1.878259417 |
| H | -1.098338835 | 0.435410911 | 0.161962453 |
| H | 0.443606866 | 0.508912272 | -0.756899226 |
| H | 3.075040984 | -2.279824167 | 0.436727596 |
| H | 1.743830853 | -3.320118328 | -2.072713185 |
| H | 1.042051263 | -4.383909283 | -0.828600224 |
| H | -1.833097976 | -1.880427200 | -1.899052142 |
| H | -0.266947604 | -1.293430986 | -2.539231106 |
| H | -0.959596328 | -1.994136617 | 0.364233192 |
| C | 1.111745780 | -0.083928435 | 2.909123615 |
| C | 1.668039974 | -0.214043834 | 4.269567290 |
| C | -1.426022853 | 0.060388326 | 2.570119056 |
| H | 2.020887091 | -1.236032149 | 4.441965094 |
| H | 2.514189386 | 0.464505944 | 4.412029291 |
| H | 0.883309002 | 0.016882527 | 4.997188067 |
| C | 4.835104763 | 1.059350125 | -0.188470129 |
| C | 5.645755319 | 2.203866908 | -0.212632724 |
| H | 5.846999124 | 2.752216642 | 0.702087638 |

| | | | |
|---|---|---|---|
| C | 6.198253570 | 2.637964268 | -1.418455003 |
| H | 6.827030880 | 3.523217827 | -1.432980520 |
| C | 5.947050409 | 1.937903064 | -2.599792790 |
| H | 6.379184793 | 2.280124644 | -3.535381512 |
| C | 5.139996954 | 0.797369565 | -2.579947007 |
| H | 4.944949446 | 0.251109287 | -3.497965177 |
| C | 4.582357031 | 0.359152162 | -1.380932383 |
| H | 3.957301183 | -0.530243508 | -1.369931020 |
| C | 4.300656818 | 1.724293076 | 2.612262417 |
| C | 3.545419453 | 2.900138237 | 2.454103095 |
| H | 2.918411928 | 3.033083396 | 1.574918495 |
| C | 3.611976362 | 3.908994968 | 3.412149001 |
| H | 3.031852087 | 4.817497955 | 3.281186971 |
| C | 4.426112912 | 3.750222519 | 4.537584150 |
| H | 4.476372911 | 4.536131103 | 5.285191676 |
| C | 5.177041959 | 2.584997281 | 4.698848205 |
| H | 5.813669662 | 2.463369068 | 5.570143351 |
| C | 5.117763183 | 1.570604472 | 3.740674591 |
| H | 5.706002289 | 0.667810826 | 3.870808959 |
| C | 5.181118800 | -0.968997164 | 1.876321773 |
| C | 6.543675573 | -0.990133471 | 1.538756111 |
| H | 6.965100877 | -0.204444928 | 0.918970598 |
| C | 7.358057550 | -2.023908607 | 1.999150514 |
| H | 8.411411714 | -2.037029009 | 1.735932067 |
| C | 6.821724262 | -3.040248813 | 2.793296806 |
| H | 7.459304939 | -3.844567613 | 3.147929682 |
| C | 5.465485564 | -3.027589151 | 3.125903834 |
| H | 5.045869671 | -3.821620272 | 3.736326353 |
| C | 4.645832485 | -1.997170350 | 2.667403368 |
| H | 3.588310151 | -1.995419401 | 2.911234381 |
| O | -2.345737205 | -0.240641386 | 1.838017517 |
| O | -1.559455845 | 0.435578976 | 3.852821633 |
| C | -2.919616572 | 0.448141012 | 4.354825225 |
| H | -2.841928266 | 0.795700767 | 5.383662375 |
| H | -3.534440031 | 1.125688482 | 3.758926324 |
| H | -3.344945630 | -0.556985897 | 4.312925308 |

==============================

20B-TS1

==============================

| | | | |
|---|---|---|---|
| C | 0.116776787 | -0.124578693 | -0.090506964 |
| C | -0.122415748 | 0.099439719 | 1.838285749 |
| C | 2.864260636 | -0.103247398 | 0.253221752 |
| C | 0.947601927 | 0.044161018 | 2.547811133 |
| Rh | 1.369746783 | -1.336904461 | 1.002445866 |
| P | 1.574207854 | -3.014365944 | -0.716313398 |
| C | 0.823840122 | 0.873575279 | -0.667017764 |
| C | 2.194994403 | 1.196593993 | -0.155647512 |
| C | 2.776365847 | 1.977179021 | -1.365080490 |
| C | 0.558933686 | 1.719370732 | -1.888066057 |
| O | 1.643872026 | 2.642344335 | -1.918594890 |
| H | 3.571999912 | -0.011274356 | 1.083723154 |
| H | 3.305553172 | -0.621485934 | -0.598513883 |
| H | -0.825627355 | -0.522016590 | -0.454514119 |
| H | 0.544772800 | 1.087640053 | -2.791166629 |
| H | -0.382822831 | 2.277143824 | -1.839083570 |
| H | 3.515086263 | 2.733032430 | -1.092498487 |
| H | 3.221978983 | 1.274897448 | -2.088534690 |
| H | 2.095139290 | 1.868972031 | 0.710708905 |
| C | 1.649567706 | 0.401862047 | 3.797252380 |
| C | -1.593225994 | -0.005566642 | 2.017761312 |
| H | 2.390256018 | 1.186844217 | 3.604802585 |
| H | 0.935712691 | 0.778020048 | 4.538220962 |
| H | 2.180758432 | -0.457408941 | 4.217001447 |
| O | -2.307169371 | -0.769325409 | 1.400672893 |
| O | -1.984754409 | 0.825525178 | 2.985156853 |
| C | -3.397193959 | 0.775966959 | 3.315833596 |
| H | -3.533238922 | 1.520433414 | 4.098401303 |
| H | -3.996633737 | 1.019283873 | 2.435971966 |
| H | -3.663812350 | -0.221002107 | 3.673596610 |
| C | 2.737063754 | -4.314864610 | -0.173046412 |
| C | 3.893937791 | -3.899099959 | 0.512301108 |
| H | 4.082246251 | -2.839151851 | 0.675223844 |
| C | 4.819276488 | -4.835497387 | 0.970429047 |
| H | 5.712506356 | -4.504270998 | 1.491611446 |
| C | 4.590424352 | -6.197056410 | 0.761619380 |
| H | 5.306538404 | -6.928953005 | 1.122653664 |
| C | 3.438209506 | -6.617204647 | 0.094376083 |
| H | 3.257096586 | -7.676271962 | -0.062354556 |
| C | 2.512294153 | -5.683884915 | -0.372937140 |
| H | 1.616451743 | -6.023104721 | -0.882193864 |
| C | -0.016840279 | -3.792816758 | -1.146259278 |
| C | -1.138315453 | -3.577044295 | -0.330274343 |
| H | -1.079970924 | -2.930815485 | 0.541592453 |
| C | -2.362481514 | -4.166018146 | -0.650081400 |
| H | -3.225287872 | -3.982193439 | -0.017443592 |
| C | -2.476104191 | -4.973959288 | -1.782250251 |
| H | -3.429032392 | -5.431313139 | -2.030527366 |
| C | -1.365573236 | -5.184184968 | -2.605658299 |
| H | -1.454381249 | -5.803461130 | -3.493220484 |
| C | -0.142340857 | -4.592849976 | -2.296235512 |
| H | 0.710741440 | -4.741934721 | -2.952074021 |
| C | 2.214694436 | -2.342870085 | -2.295414312 |
| C | 1.328437468 | -1.661998509 | -3.147992420 |
| H | 0.263711656 | -1.656078028 | -2.933888762 |
| C | 1.806493205 | -1.006941524 | -4.282364837 |
| H | 1.110734118 | -0.496255901 | -4.941892560 |
| C | 3.173772521 | -1.009870044 | -4.574061479 |
| H | 3.544289370 | -0.495403560 | -5.455275000 |
| C | 4.057332781 | -1.689931127 | -3.734641486 |
| H | 5.118824143 | -1.710719453 | -3.963055788 |
| C | 3.583492703 | -2.355209752 | -2.600858272 |
| H | 4.282048386 | -2.889372268 | -1.964026309 |

==============================

20B-TS2

==============================

| | | | |
|---|---|---|---|
| C | -0.213899808 | -0.127039776 | 0.033517850 |
| C | -0.017610923 | 0.001137792 | 2.323890093 |
| Rh | 1.824371013 | -0.015037807 | 1.052670851 |
| P | 4.131151951 | 0.281011567 | 1.361513387 |
| C | 2.085182540 | -1.831334040 | 0.333111604 |
| C | 1.007529453 | -2.300173694 | -0.302597749 |
| C | -0.325733125 | -1.589127465 | -0.290533565 |
| C | -0.840172874 | -1.983282265 | -1.701856864 |
| C | 0.883515547 | -3.458353019 | -1.282771873 |
| O | -0.432751886 | -3.338463875 | -1.825145819 |
| H | -1.147871939 | 0.381139964 | 0.245935378 |
| H | 0.337931469 | 0.448827122 | -0.723679263 |
| H | 3.044994925 | -2.335689714 | 0.403453172 |
| H | 1.647719977 | -3.383850770 | -2.070993216 |
| H | 0.973459237 | -4.440249036 | -0.806030474 |
| H | -1.926637933 | -1.940744164 | -1.802463921 |
| H | -0.378432800 | -1.342255435 | -2.471350647 |

| | | | |
|---|---|---|---|
| H | -0.977007757 | -2.090736037 | 0.438904929 |
| C | 1.136779241 | -0.143586176 | 2.884832565 |
| C | -1.459417108 | 0.100074417 | 2.635807188 |
| C | 1.787135737 | -0.249658216 | 4.206839406 |
| H | -1.887393842 | 1.038916631 | 2.269653668 |
| H | -2.026188275 | -0.727764839 | 2.195811458 |
| H | -1.571382311 | 0.064533133 | 3.724374323 |
| C | 4.442172796 | 1.389112767 | 2.773762067 |
| C | 5.373091712 | 1.072413468 | 3.770611228 |
| H | 5.951826320 | 0.157335159 | 3.706444400 |
| C | 5.546749489 | 1.931929311 | 4.856300988 |
| H | 6.272780094 | 1.684321463 | 5.625009255 |
| C | 4.792378469 | 3.102207130 | 4.956190669 |
| H | 4.933088485 | 3.768815883 | 5.802013801 |
| C | 3.859776617 | 3.420786182 | 3.964289556 |
| H | 3.275850460 | 4.333571488 | 4.035714114 |
| C | 3.683154989 | 2.567452800 | 2.877274591 |
| H | 2.965828035 | 2.827311149 | 2.101571670 |
| C | 5.232419178 | -1.159448547 | 1.536929243 |
| C | 4.855029295 | -2.221751233 | 2.375683357 |
| H | 3.913626578 | -2.178795275 | 2.911310439 |
| C | 5.710470414 | -3.310000420 | 2.542267024 |
| H | 5.418362019 | -4.126875319 | 3.195441122 |
| C | 6.937229396 | -3.349637636 | 1.875583666 |
| H | 7.599522204 | -4.200275496 | 2.007401499 |
| C | 7.313336098 | -2.296110677 | 1.040404866 |
| H | 8.267196300 | -2.323929774 | 0.522490211 |
| C | 6.465885967 | -1.202322455 | 0.867387300 |
| H | 6.765938575 | -0.387407719 | 0.216722370 |
| C | 4.702571604 | 1.158532467 | -0.137419720 |
| C | 5.439719894 | 2.349489194 | -0.081735923 |
| H | 5.691611718 | 2.792897651 | 0.876043231 |
| C | 5.858625610 | 2.963345518 | -1.263180256 |
| H | 6.432863523 | 3.883718822 | -1.215147321 |
| C | 5.547766719 | 2.396570581 | -2.500211809 |
| H | 5.877300699 | 2.878072482 | -3.415897444 |
| C | 4.815937111 | 1.207541709 | -2.561209847 |
| H | 4.578901242 | 0.760997641 | -3.522258663 |
| C | 4.392151150 | 0.590568816 | -1.386458418 |
| H | 3.834286431 | -0.341868100 | -1.438102414 |
| O | 2.846388615 | -0.806958628 | 4.402413987 |
| O | 1.061151184 | 0.379535935 | 5.138026958 |
| C | 1.654601317 | 0.412082203 | 6.461482774 |
| H | 0.949528408 | 0.966052985 | 7.078994675 |
| H | 1.792585410 | -0.603457319 | 6.839668589 |
| H | 2.621022181 | 0.919776654 | 6.417639362 |

==============================

21A

==============================

| | | | |
|---|---|---|---|
| Rh | 0.559018909 | 1.354067581 | 0.975842941 |
| C | -0.479022585 | -0.539612224 | -0.290857704 |
| C | 0.752363575 | 2.495277634 | -0.718325770 |
| C | 0.488862164 | 0.039209046 | -1.066505481 |
| C | 0.458323851 | 1.425499650 | -1.754831555 |
| C | 1.548082706 | 1.237069139 | -2.850389033 |
| C | 1.654011115 | -0.710520531 | -1.684646180 |
| O | 1.733863612 | -0.166336318 | -2.991992243 |
| H | 0.041965681 | 3.320101454 | -0.681207228 |
| H | 1.789424906 | 2.848438660 | -0.725917475 |
| H | -0.382337288 | -1.606252813 | -0.080687668 |
| H | 2.585837485 | -0.531284774 | -1.117657165 |
| H | 1.491160165 | -1.788968579 | -1.757110371 |
| H | 1.256980410 | 1.640318631 | -3.822205965 |
| H | 2.487572077 | 1.719369409 | -2.532902236 |
| H | -0.535942140 | 1.572857438 | -2.190815449 |
| C | -1.684627983 | 0.136238443 | 0.249561777 |
| C | -1.397807599 | 1.326416294 | 0.830417926 |
| C | -3.016771534 | -0.553958338 | 0.145254414 |
| C | -2.361211979 | 2.323372268 | 1.355398386 |
| P | 1.049076564 | 2.930410707 | 2.555131970 |
| H | -3.800253118 | 0.024642471 | 0.633138740 |
| H | -3.279400806 | -0.721637144 | -0.905811143 |
| H | -2.959513575 | -1.540358386 | 0.622422849 |
| C | 0.617197343 | 2.356737868 | 4.228665550 |
| C | 1.530399272 | 2.499618761 | 5.287016669 |
| H | 2.512554110 | 2.926569551 | 5.108728153 |
| C | 1.174831310 | 2.090475659 | 6.570625373 |
| H | 1.883083595 | 2.202417767 | 7.386042255 |
| C | -0.087025527 | 1.539198737 | 6.806719268 |
| H | -0.359474337 | 1.220357273 | 7.808306261 |
| C | -0.997220403 | 1.395794872 | 5.757908405 |
| H | -1.978037169 | 0.967189407 | 5.939574344 |
| C | -0.649624505 | 1.799781915 | 4.469283094 |
| H | -1.369240628 | 1.681796211 | 3.665784766 |
| C | 2.860301571 | 2.746091414 | 2.392703359 |
| C | 3.695330178 | 3.770383936 | 1.922346491 |
| H | 3.303226797 | 4.770206084 | 1.769170026 |
| C | 5.041456939 | 3.508864588 | 1.668093129 |
| H | 5.685697372 | 4.309486666 | 1.317166556 |
| C | 5.563945205 | 2.228528171 | 1.868309821 |
| H | 6.612596505 | 2.032749850 | 1.666135805 |
| C | 4.741239726 | 1.204415946 | 2.341816163 |
| H | 5.147709060 | 0.213029795 | 2.517631951 |
| C | 3.395210124 | 1.457943226 | 2.605535133 |
| H | 2.770525565 | 0.667269845 | 3.015656399 |
| C | 0.639834991 | 4.700667417 | 2.467603086 |
| C | 0.162366188 | 5.375854179 | 3.600781055 |
| H | 0.023148961 | 4.845753549 | 4.536831513 |
| C | -0.135257699 | 6.737324057 | 3.528246257 |
| H | -0.501776322 | 7.253476155 | 4.410342351 |
| C | 0.037735559 | 7.432023178 | 2.330752879 |
| H | -0.193601644 | 8.491639965 | 2.278202541 |
| C | 0.508835086 | 6.762433856 | 1.197429915 |
| H | 0.644317489 | 7.299157105 | 0.263266745 |
| C | 0.804545885 | 5.402601223 | 1.261662961 |
| H | 1.168553679 | 4.896273287 | 0.376025485 |
| O | -1.918042143 | 3.581660191 | 1.185211869 |
| O | -3.413213091 | 2.036916784 | 1.894600154 |
| C | -2.771740393 | 4.626025077 | 1.704885831 |
| H | -3.725809428 | 4.633758410 | 1.172948700 |
| H | -2.221497616 | 5.551400583 | 1.542831379 |
| H | -2.955636545 | 4.467167842 | 2.769851645 |

==============================

21B

==============================

| | | | |
|---|---|---|---|
| Rh | 0.429295715 | 1.322757464 | 1.066960889 |
| C | -0.980007080 | -0.325519325 | -0.163502171 |
| C | 0.932478680 | 2.307623000 | -0.659846652 |
| C | 0.115599090 | -0.034265347 | -0.927773874 |
| C | 0.445989486 | 1.283744372 | -1.669885620 |
| C | 1.514815741 | 0.790870053 | -2.688996812 |
| C | 1.085000797 | -1.072144381 | -1.461489173 |

| | | | |
|---|---|---|---|
| O | 1.360058624 | -0.619653876 | -2.777534052 |
| H | 0.439860131 | 3.279071056 | -0.706187800 |
| H | 2.021599516 | 2.421749913 | -0.621510782 |
| H | -1.152114586 | -1.369286441 | 0.093939334 |
| H | 2.006348071 | -1.109274740 | -0.851363769 |
| H | 0.660504623 | -2.078236746 | -1.512179722 |
| H | 1.384013785 | 1.209990342 | -3.688485448 |
| H | 2.524913445 | 1.045997870 | -2.326884226 |
| H | -0.461801281 | 1.633078770 | -2.173617794 |
| C | -1.983390395 | 0.656102127 | 0.314158923 |
| C | -1.478495032 | 1.776056646 | 0.891769771 |
| C | -3.441487644 | 0.340881911 | 0.213693198 |
| C | -2.148865899 | 3.032101956 | 1.321881997 |
| H | -2.050807526 | 3.192827544 | 2.400323542 |
| H | -1.680435386 | 3.895294115 | 0.836447866 |
| H | -3.210650358 | 2.993199239 | 1.065187167 |
| P | 1.199346474 | 2.881077839 | 2.568721108 |
| C | 0.858291462 | 2.290527819 | 4.260686849 |
| C | 1.792691814 | 2.487499390 | 5.290197898 |
| H | 2.732437782 | 2.991377279 | 5.086053052 |
| C | 1.516085732 | 2.025880326 | 6.575750754 |
| H | 2.240988668 | 2.178192260 | 7.369615271 |
| C | 0.313616609 | 1.365803197 | 6.841743504 |
| H | 0.105261840 | 1.003696551 | 7.844004941 |
| C | -0.618603567 | 1.166809754 | 5.821660935 |
| H | -1.552385507 | 0.652525074 | 6.027542136 |
| C | -0.349106140 | 1.625168039 | 4.532433502 |
| H | -1.079325926 | 1.465385469 | 3.743133613 |
| C | 3.012408918 | 2.815534423 | 2.370793175 |
| C | 3.817287107 | 3.946212415 | 2.180244886 |
| H | 3.381631195 | 4.939443128 | 2.195066179 |
| C | 5.190710286 | 3.794570191 | 1.978349958 |
| H | 5.811477929 | 4.673981308 | 1.836022152 |
| C | 5.766563492 | 2.523429924 | 1.963076647 |
| H | 6.834835560 | 2.413082837 | 1.803322649 |
| C | 4.970655392 | 1.391981091 | 2.162571877 |
| H | 5.417444447 | 0.402349931 | 2.165313543 |
| C | 3.600591283 | 1.535857751 | 2.367433480 |
| H | 2.992919921 | 0.651674999 | 2.556915078 |
| C | 0.667455193 | 4.617271929 | 2.463911503 |
| C | -0.031923749 | 5.219250901 | 3.519806429 |
| H | -0.213935070 | 4.670737806 | 4.438368296 |
| C | -0.497122576 | 6.528984334 | 3.390740920 |
| H | -1.038106784 | 6.988333087 | 4.212378244 |
| C | -0.267454892 | 7.245535177 | 2.215549272 |
| H | -0.631174305 | 8.263774044 | 2.119174626 |
| C | 0.437274175 | 6.652403537 | 1.163674853 |
| H | 0.627220363 | 7.209791370 | 0.251388646 |
| C | 0.899547474 | 5.343324410 | 1.282011617 |
| H | 1.454607579 | 4.896800020 | 0.463078931 |
| O | -4.343969221 | 1.075796853 | 0.560822438 |
| O | -3.623879682 | -0.887445149 | -0.306312746 |
| C | -5.000312157 | -1.312455726 | -0.428701904 |
| H | -4.958778648 | -2.310890303 | -0.861879467 |
| H | -5.478546395 | -1.333210403 | 0.553558181 |
| H | -5.549861347 | -0.628141700 | -1.079100636 |

==============================

23A

==============================

| | | | |
|---|---|---|---|
| Rh | -0.568687356 | 1.994153272 | 1.000605922 |
| P | 0.795487008 | 2.247131181 | 2.787598270 |
| C | -0.095595233 | 0.120854759 | 0.653693154 |
| C | 0.351971817 | -0.362083138 | -0.506870590 |
| C | 0.975824271 | 0.356211888 | -1.691566026 |
| C | 1.703313331 | -0.820832033 | -2.378082049 |
| C | 0.393817273 | -1.859037674 | -0.834264611 |
| O | 0.859309283 | -1.944115808 | -2.170961153 |
| C | 0.071971617 | 1.427104459 | 4.248997330 |
| C | 0.562461825 | 1.789846922 | 5.516813820 |
| H | 1.343877023 | 2.538687374 | 5.606658152 |
| C | 0.044030036 | 1.191639200 | 6.661842482 |
| H | 0.430950198 | 1.472445653 | 7.636672011 |
| C | -0.974844156 | 0.240335663 | 6.556973080 |
| H | -1.380698974 | -0.219562478 | 7.452839460 |
| C | -1.475313596 | -0.113151302 | 5.303872053 |
| H | -2.272565997 | -0.845153709 | 5.220239928 |
| C | -0.953472211 | 0.475179879 | 4.150780317 |
| H | -1.360285677 | 0.187985236 | 3.187193918 |
| C | 2.502397087 | 1.656147598 | 2.602756197 |
| C | 2.974911357 | 0.584121825 | 3.374114250 |
| H | 2.330650274 | 0.102000450 | 4.101412978 |
| C | 4.285869974 | 0.136148783 | 3.209874308 |
| H | 4.646966107 | -0.694009516 | 3.809187414 |
| C | 5.129241286 | 0.752503689 | 2.285161841 |
| H | 6.149708518 | 0.402129081 | 2.163385846 |
| C | 4.660318644 | 1.818387438 | 1.512285288 |
| H | 5.312386056 | 2.296192933 | 0.787522692 |
| C | 3.349727022 | 2.265151178 | 1.660647266 |
| H | 2.983488575 | 3.075652454 | 1.038162164 |
| C | 0.824452091 | 4.010289254 | 3.249216125 |
| C | 1.987977302 | 4.792047493 | 3.267354283 |
| H | 2.952676735 | 4.356605778 | 3.035463044 |
| C | 1.909995731 | 6.143633389 | 3.607917516 |
| H | 2.814784883 | 6.743502239 | 3.623503632 |
| C | 0.681386351 | 6.719957792 | 3.932067916 |
| H | 0.627772810 | 7.772093235 | 4.194379084 |
| C | -0.479358516 | 5.940425470 | 3.929779503 |
| H | -1.434946655 | 6.382182856 | 4.195002547 |
| C | -0.410460358 | 4.592852201 | 3.590317824 |
| H | -1.314229404 | 3.988158603 | 3.605050740 |
| H | -0.389910523 | -0.554529386 | 1.462039595 |
| H | 1.081905175 | -2.364873111 | -0.134695995 |
| H | -0.583082857 | -2.348045495 | -0.771091108 |
| H | 1.835731240 | -0.681366355 | -3.453168493 |
| H | 2.694090071 | -0.974695007 | -1.914788813 |
| H | 0.180522807 | 0.702944843 | -2.365866846 |
| C | 0.380460368 | 3.194719578 | -0.207029317 |
| C | 1.264261963 | 2.879409332 | -1.171747209 |
| C | 1.917429319 | 1.515804280 | -1.330854910 |
| C | -0.319959876 | 4.490852156 | 0.093908601 |
| C | 1.588804495 | 3.959343058 | -2.168780381 |
| H | 2.435366434 | 1.251265137 | -0.403817643 |
| H | 2.677251073 | 1.597811894 | -2.109716768 |
| H | -1.161582437 | 4.350443256 | 0.799593265 |
| H | 0.363777570 | 5.204668793 | 0.560911269 |
| H | -0.723936735 | 4.938636581 | -0.817355159 |
| O | 1.396186333 | 5.149496223 | -1.999784231 |
| O | 2.112248377 | 3.446090932 | -3.298139295 |
| C | 2.434547452 | 4.407763376 | -4.325963912 |
| H | 2.838588527 | 3.824409139 | -5.152499802 |
| H | 1.535841328 | 4.947549824 | -4.633682245 |
| H | 3.171826653 | 5.124959943 | -3.957491905 |

==============================
23B
==============================
Rh -0.328000000 2.229100000 0.983800000
P 0.996700000 2.280400000 2.812800000
C -0.220700000 0.304800000 0.603700000
C 0.227300000 -0.307100000 -0.496600000
C 1.184100000 0.184600000 -1.565800000
C 1.752000000 -1.160400000 -2.061500000
C -0.107500000 -1.754800000 -0.864100000
O 0.643000000 -2.046200000 -2.038100000
C 0.061100000 1.378200000 4.095700000
C -0.476600000 2.047200000 5.207600000
H -0.319200000 3.111300000 5.340400000
C -1.207300000 1.338000000 6.160200000
H -1.616800000 1.862200000 7.018100000
C -1.405300000 -0.035700000 6.017200000
H -1.974800000 -0.582400000 6.762400000
C -0.867000000 -0.708900000 4.917000000
H -1.011600000 -1.779100000 4.806900000
C -0.143200000 -0.008200000 3.956300000
H 0.276800000 -0.539100000 3.109000000
C 2.670300000 1.571700000 2.698100000
C 3.089000000 0.503700000 3.504600000
H 2.409900000 0.045400000 4.213600000
C 4.399300000 0.031700000 3.409800000
H 4.714700000 -0.797500000 4.035500000
C 5.300800000 0.626100000 2.527400000
H 6.319300000 0.256500000 2.459700000
C 4.894300000 1.705100000 1.737500000
H 5.595600000 2.178900000 1.057400000
C 3.586900000 2.175400000 1.818400000
H 3.277400000 3.014400000 1.204200000
C 1.227500000 3.990000000 3.419000000
C 2.474000000 4.433200000 3.892600000
H 3.332800000 3.772600000 3.889700000
C 2.615200000 5.732600000 4.379100000
H 3.582800000 6.065200000 4.742100000
C 1.520400000 6.597100000 4.404900000
H 1.635700000 7.608400000 4.783100000
C 0.274200000 6.158000000 3.950100000
H -0.583400000 6.823200000 3.976600000
C 0.125300000 4.863000000 3.458200000
H -0.845200000 4.538800000 3.101500000
H -0.857400000 -0.252300000 1.302200000
H 0.171500000 -2.425400000 -0.034800000
H -1.168700000 -1.909200000 -1.086300000
H 2.139500000 -1.121400000 -3.082600000
H 2.559100000 -1.501900000 -1.389200000
H 0.597800000 0.617200000 -2.390000000
C 0.922700000 3.125600000 -0.281500000
C 1.841100000 2.630900000 -1.135500000
C 2.262300000 1.173700000 -1.098500000
C 0.392900000 4.480300000 -0.080000000
C 2.509000000 3.457600000 -2.207700000
H 2.560400000 0.898300000 -0.082900000
H 3.145800000 1.059100000 -1.736300000
H 3.590900000 3.486400000 -2.022600000
H 2.374300000 2.962700000 -3.177400000
H 2.137800000 4.477400000 -2.272700000
O 0.988200000 5.532300000 -0.613000000
O -0.599500000 4.568100000 0.670700000
C 0.429900000 6.832800000 -0.277900000
H 0.459300000 6.977500000 0.803900000
H -0.600300000 6.900400000 -0.632200000
H 1.066300000 7.553200000 -0.788500000
==============================
21A-TS
==============================
C -0.336425994 0.229028048 0.102433452
C -0.008296671 -0.015671624 2.117656258
Rh 1.694568737 0.009382272 1.056100558
C 0.847150749 -2.143226167 1.544158942
C 0.830780393 -1.958704339 0.175442417
C -0.212632599 -1.125376281 -0.572560221
C 0.402155985 -1.134366875 -1.987460461
C 1.611403038 -2.742220816 -0.864913082
O 1.008799222 -2.414431681 -2.112449263
H -1.337350402 0.470634604 0.445179174
H 0.036390140 1.090074967 -0.452954021
H 1.445777271 -2.955431601 1.957396959
H 2.676128110 -2.462327030 -0.849468799
H 1.538731647 -3.825902591 -0.717825525
H -0.333984269 -1.028529476 -2.786993861
H 1.151675623 -0.328522732 -2.077413181
H -1.165113375 -1.671446670 -0.582207301
C 0.068949975 -1.335760627 2.500495036
C -0.404692449 -1.957060848 3.783130808
C -0.593824005 1.117124936 2.877618669
P 2.577265143 2.092250015 1.145485759
H -0.775997160 -1.205277824 4.476416777
H -1.203044302 -2.680309256 3.574553896
H 0.414186909 -2.515504230 4.252206852
C 3.200614637 2.790208817 2.697622137
C 4.330829129 3.626244634 2.697527307
H 4.847601567 3.848660681 1.767774844
C 4.786058132 4.175641351 3.893999220
H 5.660312837 4.819598924 3.893882428
C 4.118610086 3.897673817 5.090656636
H 4.475052063 4.330358279 6.020647845
C 3.000069009 3.062491791 5.094959455
H 2.485754050 2.842219144 6.025429694
C 2.540418670 2.503131914 3.902120470
H 1.679594267 1.843002287 3.915282589
C 3.888997582 1.145161787 0.301525312
C 4.291335884 1.303902278 -1.040118331
H 4.027909066 2.206568168 -1.581305995
C 5.031632535 0.308237041 -1.664504563
H 5.353013453 0.445587140 -2.692698940
C 5.366794844 -0.874180130 -0.982922293
H 5.938284100 -1.645647956 -1.489359831
C 4.998086118 -1.038526509 0.346610535
H 5.294681633 -1.927732817 0.894580431
C 4.261296774 -0.035522679 1.003141912
H 4.104979583 -0.105213295 2.079247050
C 1.981636493 3.451848828 0.104556320
C 1.870622444 4.756885860 0.609476440
H 2.195135261 4.983106740 1.619479727
C 1.343503489 5.772136441 -0.189245359
H 1.265222956 6.779861453 0.207393603
C 0.922544690 5.497701244 -1.491095619
H 0.514733067 6.291702381 -2.109317077

C 1.024576788 4.199993845 -1.998239308
H 0.697695217 3.982694182 -3.010595804
C 1.543281763 3.179301594 -1.204731761
H 1.616014685 2.173534973 -1.607468074
O -0.595152054 2.253538151 2.149130142
O -0.980386549 1.058408137 4.029246218
C -1.057563273 3.436960163 2.834863436
H -2.086893380 3.305900372 3.176128889
H -0.986869861 4.238455066 2.100427238
H -0.417332969 3.645297526 3.696102185
==============================
21B-TS
==============================
C -0.696811262 0.227366114 0.437428350
C -0.045150363 -0.288298875 2.296642363
Rh 1.465300605 0.194299605 1.061159955
C 1.021072261 -2.120262963 1.181630661
C 0.778616386 -1.681961891 -0.105647540
C -0.484312516 -0.925415805 -0.528999065
C -0.104134697 -0.557947146 -1.977810248
C 1.502587546 -2.095842244 -1.373452364
O 0.666472345 -1.658676496 -2.441786007
H -1.652274308 0.195400574 0.955877848
H -0.570048651 1.234941350 0.038982288
H 1.781605301 -2.877943879 1.356768021
H 2.499730289 -1.631978461 -1.431978482
H 1.624062967 -3.182979486 -1.450373801
H -0.963288440 -0.441860318 -2.641870917
H 0.488699272 0.373687347 -1.988477199
H -1.333189088 -1.621425855 -0.536207500
C 0.266282321 -1.631790603 2.342291364
C 0.045960688 -2.465738389 3.551446248
C -0.770383275 0.528381007 3.316834022
H -0.267794929 0.431593920 4.283720902
H -0.812810496 1.579923600 3.023873995
H -1.787261122 0.151430232 3.470120778
P 2.171029740 2.340161403 1.383188035
C 3.015805085 2.893387376 2.888235220
C 4.223112694 3.608011819 2.809532558
H 4.666083999 3.829303254 1.842427653
C 4.854798268 4.030223147 3.977800503
H 5.789288031 4.579666763 3.916422643
C 4.288228678 3.745466612 5.223927013
H 4.784001164 4.075959009 6.131481516
C 3.089961044 3.032594933 5.304016207
H 2.653107148 2.807183804 6.271983942
C 2.453746610 2.601579350 4.139660675
H 1.527995666 2.038196033 4.202046671
C 3.406228004 1.604512207 0.259846994
C 3.568027654 1.926030985 -1.104531512
H 3.111553264 2.824582187 -1.506514536
C 4.321233273 1.098236649 -1.925458758
H 4.457842380 1.359648584 -2.970663818
C 4.907973812 -0.074360948 -1.418624648
H 5.490241971 -0.712155377 -2.076670905
C 4.764616139 -0.404611251 -0.077227529
H 5.243399413 -1.291202783 0.327006646
C 4.015155663 0.427080381 0.774453050
H 4.021023870 0.238324573 1.846835688
C 1.372818349 3.768455476 0.602586087
C 1.377412583 5.031027824 1.214931328
H 1.894153494 5.180614418 2.157290763
C 0.716821624 6.100135957 0.609134282
H 0.724793611 7.074886644 1.086976057
C 0.050823361 5.920949596 -0.604119404
H -0.461064088 6.756875268 -1.071137946
C 0.040213242 4.665570180 -1.217756019
H -0.475778343 4.523612140 -2.162550701
C 0.690481192 3.591186495 -0.615976431
H 0.683274615 2.619860442 -1.101185608
O -0.579597127 -2.099663259 4.528238713
O 0.632978872 -3.672107699 3.432062701
C 0.478226648 -4.550496205 4.568999395
H 0.997407539 -5.469453973 4.300073173
H 0.921479596 -4.099421013 5.460122394
H -0.580885680 -4.742449173 4.755989491
==============================
23A-TS
==============================
C -0.194387178 -0.028394008 0.070470186
C 0.086515421 -0.016852101 2.094489241
Rh 1.713165760 0.046949293 0.819096358
P 2.416395363 2.139312613 0.844604388
C -0.282674475 -1.291982810 -0.358425963
C 0.538176185 -2.352059170 0.368269966
C 0.345346594 -3.587407422 -0.557858860
C -1.018008754 -1.987735729 -1.479048776
O -0.164356830 -3.086184062 -1.787873908
C 3.970957965 1.298779176 0.398943889
C 5.027855849 1.110395349 1.314082060
H 5.004126376 1.605958793 2.279188090
C 6.108495388 0.306267419 0.967377035
H 6.929285933 0.179067861 1.666322349
C 6.144167663 -0.333933414 -0.277542031
H 6.992757890 -0.959203004 -0.538106573
C 5.100710003 -0.165789768 -1.187563604
H 5.134782522 -0.652675420 -2.157257832
C 4.014604304 0.649747489 -0.861305907
H 3.232271963 0.827911423 -1.594037565
C 1.967349940 3.308554485 -0.463188464
C 2.951158976 3.873846517 -1.291017657
H 3.995913812 3.607539776 -1.163423845
C 2.583274394 4.783711747 -2.281886571
H 3.345433078 5.219605446 -2.920870788
C 1.242201235 5.133189001 -2.449908435
H 0.960036375 5.841268989 -3.223130672
C 0.261600253 4.574911513 -1.625102057
H -0.780828472 4.848890195 -1.755187562
C 0.619169327 3.663202854 -0.634350319
H -0.148040973 3.235855035 0.004576124
C 2.615031014 3.052960809 2.393049880
C 2.651377384 4.456326217 2.408266120
H 2.534858190 5.018781360 1.487319563
C 2.839300806 5.129547602 3.615620348
H 2.867531620 6.214850410 3.625639323
C 2.987814251 4.413519582 4.805252027
H 3.129852799 4.943195322 5.742303496
C 2.945166657 3.015505089 4.793778331
H 3.048998871 2.457840373 5.719335829
C 2.754244895 2.333885507 3.594394388
H 2.698937827 1.248286861 3.587137292
H -0.762970218 0.834793275 -0.258400331

| | | | |
|---|---|---|---|
| H | -2.012234361 | -2.332657761 | -1.144253905 |
| H | -1.142536730 | -1.378023205 | -2.376672791 |
| H | 1.266537759 | -4.132942220 | -0.772004609 |
| H | -0.373224020 | -4.279127198 | -0.088829343 |
| H | 1.629889404 | -2.096821477 | 0.397405144 |
| C | -0.136520468 | -1.264458877 | 2.603360363 |
| C | 0.053348484 | -2.547582645 | 1.817012671 |
| C | -0.432567709 | 1.266598836 | 2.689844935 |
| C | -0.723240410 | -1.405990395 | 3.968672081 |
| H | -0.912883617 | -3.069143135 | 1.812014150 |
| H | 0.742458013 | -3.223858913 | 2.335165767 |
| H | 0.180117192 | 1.578850197 | 3.537799909 |
| H | -0.427276654 | 2.070595852 | 1.951172141 |
| H | -1.450325397 | 1.140677201 | 3.066884589 |
| O | -1.043648257 | -2.692671419 | 4.230208329 |
| O | -0.876315099 | -0.506329991 | 4.775231977 |
| C | -1.593717764 | -2.937641964 | 5.540397774 |
| H | -1.785691593 | -4.009322711 | 5.580534340 |
| H | -0.880991538 | -2.641275997 | 6.313962105 |
| H | -2.518773854 | -2.372593297 | 5.677014078 |

==============================

23B-TS

==============================

| | | | |
|---|---|---|---|
| C | -0.137816331 | -0.028189601 | 0.021014276 |
| C | -0.063284927 | 0.028486806 | 2.034464589 |
| Rh | 1.700759558 | 0.015720227 | 0.931872548 |
| P | 2.385654605 | 2.113031725 | 0.900852805 |
| C | -0.176392445 | -1.296903605 | -0.401264816 |
| C | 0.451063051 | -2.364687285 | 0.488987373 |
| C | 0.354897728 | -3.625398683 | -0.416253216 |
| C | -0.725018768 | -1.998590627 | -1.621241658 |
| O | 0.110831078 | -3.148730930 | -1.733390152 |
| C | 3.974058499 | 1.272192358 | 0.605552878 |
| C | 5.008619354 | 1.203044917 | 1.561882110 |
| H | 4.953033671 | 1.801543116 | 2.465046291 |
| C | 6.107876330 | 0.383473573 | 1.334474994 |
| H | 6.912968532 | 0.350907072 | 2.062212937 |
| C | 6.184173242 | -0.395721194 | 0.172206406 |
| H | 7.047392338 | -1.033263613 | 0.006836538 |
| C | 5.165600745 | -0.345782946 | -0.777133950 |
| H | 5.232581569 | -0.935538443 | -1.685939099 |
| C | 4.061649280 | 0.489343349 | -0.574736383 |
| H | 3.310016522 | 0.588579837 | -1.353466500 |
| C | 2.027945916 | 3.223529516 | -0.483243379 |
| C | 3.073369032 | 3.698324506 | -1.293768035 |
| H | 4.098744182 | 3.396446332 | -1.104772812 |
| C | 2.791666733 | 4.570028493 | -2.344667884 |
| H | 3.600339085 | 4.939130292 | -2.968164093 |
| C | 1.476204218 | 4.968005073 | -2.590722513 |
| H | 1.260855357 | 5.646555353 | -3.410740605 |
| C | 0.437856723 | 4.498691999 | -1.782524067 |
| H | -0.583936400 | 4.813124644 | -1.972063810 |
| C | 0.704173660 | 3.627214864 | -0.727712277 |
| H | -0.098245532 | 3.274642786 | -0.089235135 |
| C | 2.473864754 | 3.081547010 | 2.425897659 |
| C | 2.416065135 | 4.483951247 | 2.399695648 |
| H | 2.278115363 | 5.008807073 | 1.460096839 |
| C | 2.536445014 | 5.202122615 | 3.589059756 |
| H | 2.492312698 | 6.286635443 | 3.568884774 |
| C | 2.716805418 | 4.530117013 | 4.800392096 |
| H | 2.813759738 | 5.094805309 | 5.722940772 |
| C | 2.764905282 | 3.133566849 | 4.830134888 |
| H | 2.892344054 | 2.611099104 | 5.773249311 |
| C | 2.633319592 | 2.406779047 | 3.649269492 |
| H | 2.641602463 | 1.320976454 | 3.673134412 |
| H | -0.625216950 | 0.853663569 | -0.374375347 |
| H | -1.781552051 | -2.285714908 | -1.476121476 |
| H | -0.644325813 | -1.417586238 | -2.542628938 |
| H | 1.264851704 | -4.228554458 | -0.438281068 |
| H | -0.477974515 | -4.258636152 | -0.068106008 |
| H | 1.531782907 | -2.168353532 | 0.717394992 |
| C | -0.545034595 | -1.142272035 | 2.537882892 |
| C | -0.302967668 | -2.457653479 | 1.828887725 |
| C | -0.444652080 | 1.388423861 | 2.513460678 |
| C | -1.470000326 | -1.260805123 | 3.723829196 |
| H | -1.285041690 | -2.924746392 | 1.660926568 |
| H | 0.236078098 | -3.138672438 | 2.501002405 |
| H | -1.042624203 | -1.948875260 | 4.463656815 |
| H | -2.424126084 | -1.699744233 | 3.403180974 |
| H | -1.663150920 | -0.311073099 | 4.216088916 |
| O | -0.376189226 | 1.526505956 | 3.846751947 |
| O | -0.744184485 | 2.309358530 | 1.770489750 |
| C | -0.724958648 | 2.838029809 | 4.353247539 |
| H | -1.756139383 | 3.082836649 | 4.089481690 |
| H | -0.052412085 | 3.590759689 | 3.937629909 |
| H | -0.606053653 | 2.768547029 | 5.433439312 |

==============================

22

==============================

| | | | |
|---|---|---|---|
| C | 0.067344073 | -0.012906702 | 0.044778950 |
| C | 0.027496114 | -0.019349106 | 1.578193726 |
| Rh | 2.065409657 | -0.029276315 | 2.181782171 |
| C | 1.342039311 | -2.073485384 | 1.757035264 |
| C | 1.882418810 | -1.739376125 | 0.529291264 |
| C | 1.110690209 | -0.988445001 | -0.527226617 |
| C | 2.279577854 | -0.424171737 | -1.348881841 |
| C | 3.160989987 | -2.203453460 | -0.122264499 |
| O | 3.252149794 | -1.472025324 | -1.347662139 |
| H | -0.934253762 | -0.252687626 | -0.331120804 |
| H | 0.272306769 | 1.004929359 | -0.295753875 |
| H | 1.770105536 | -2.855300534 | 2.380651526 |
| H | 4.030223487 | -2.000493016 | 0.520904098 |
| H | 3.148395201 | -3.278494580 | -0.349724748 |
| H | 2.027265221 | -0.200628268 | -2.388610225 |
| H | 2.674368248 | 0.488620469 | -0.869531257 |
| H | 0.617562869 | -1.754553033 | -1.143499341 |
| C | 0.309263556 | -1.218259459 | 2.335140408 |
| C | -0.328027671 | -1.643391382 | 3.630644235 |
| C | -0.768342629 | 1.089373981 | 2.224818954 |
| P | 2.499977050 | 1.872872000 | 3.378097242 |
| H | -0.694482713 | -0.794715353 | 4.201674615 |
| H | -1.176588291 | -2.302483935 | 3.403162973 |
| H | 0.381341468 | -2.214639083 | 4.236598967 |
| C | 2.595864252 | 1.856284661 | 5.179764478 |
| C | 3.799800964 | 2.115817430 | 5.855214828 |
| H | 4.709045143 | 2.324324315 | 5.298002102 |
| C | 3.823947797 | 2.096987971 | 7.249175290 |
| H | 4.753258169 | 2.294614277 | 7.775330223 |
| C | 2.655492381 | 1.824687072 | 7.965455747 |
| H | 2.679169213 | 1.810751868 | 9.051327978 |
| C | 1.458500416 | 1.567800928 | 7.292016241 |
| H | 0.553072211 | 1.355285444 | 7.852830193 |

| | | | |
|---|---|---|---|
| C | 1.421399797 | 1.577375244 | 5.898516282 |
| H | 0.494916823 | 1.374667807 | 5.367575518 |
| C | 4.056518890 | 1.247579003 | 2.654818561 |
| C | 4.555377656 | 1.735933662 | 1.421883152 |
| H | 4.172718759 | 2.669426082 | 1.020646414 |
| C | 5.534884841 | 1.025164763 | 0.737379528 |
| H | 5.921545527 | 1.410642216 | -0.201265390 |
| C | 6.028393420 | -0.183322492 | 1.249861125 |
| H | 6.796902826 | -0.725480091 | 0.707300872 |
| C | 5.541351921 | -0.684194366 | 2.454507396 |
| H | 5.934262923 | -1.611329303 | 2.861654848 |
| C | 4.545078309 | 0.009439821 | 3.157323601 |
| H | 4.219061657 | -0.349760807 | 4.129926733 |
| C | 2.150494235 | 3.546594964 | 2.785441529 |
| C | 2.366146760 | 4.672416505 | 3.594783909 |
| H | 2.739226993 | 4.554216435 | 4.608023475 |
| C | 2.091718507 | 5.944352563 | 3.095089281 |
| H | 2.259376099 | 6.816199975 | 3.720464766 |
| C | 1.601431055 | 6.098008007 | 1.795602457 |
| H | 1.387666468 | 7.091655619 | 1.412383374 |
| C | 1.376910234 | 4.978072758 | 0.990711239 |
| H | 0.985944217 | 5.097719946 | -0.015544711 |
| C | 1.643033966 | 3.702170764 | 1.484612429 |
| H | 1.445849028 | 2.827631365 | 0.871832077 |
| O | -1.256139023 | 1.932322756 | 1.300073399 |
| O | -0.931002449 | 1.260362386 | 3.420734127 |
| C | -1.970702353 | 3.079328816 | 1.813344870 |
| H | -2.803421654 | 2.757598473 | 2.442309892 |
| H | -2.330570908 | 3.612036086 | 0.933643481 |
| H | -1.293406088 | 3.708352670 | 2.396349747 |

==============================

24

==============================

| | | | |
|---|---|---|---|
| C | -0.012244356 | -0.034467050 | 0.040565421 |
| C | -0.013847711 | -0.045694020 | 1.569398642 |
| Rh | 2.093842615 | 0.009579353 | 2.061093682 |
| C | 1.424982002 | -2.057459379 | 1.661947450 |
| C | 1.878599976 | -1.698535631 | 0.407696049 |
| C | 1.028440136 | -0.965639354 | -0.601057910 |
| C | 2.130076876 | -0.354940398 | -1.479111832 |
| C | 3.127498996 | -2.126197354 | -0.318808115 |
| O | 3.150753305 | -1.355679084 | -1.524745364 |
| H | -1.024222664 | -0.296885055 | -0.296757016 |
| H | 0.144735598 | 1.001259281 | -0.284399463 |
| H | 1.908822671 | -2.823911918 | 2.258808092 |
| H | 4.026443363 | -1.932345143 | 0.283209443 |
| H | 3.114212789 | -3.193867377 | -0.578541383 |
| H | 1.816911701 | -0.147178348 | -2.505452585 |
| H | 2.505911211 | 0.575562844 | -1.021650005 |
| H | 0.526816064 | -1.742143582 | -1.196844159 |
| C | 0.403055111 | -1.221005318 | 2.287425940 |
| C | -0.038641110 | -1.557585176 | 3.685811009 |
| C | -0.968644145 | 0.962239078 | 2.163686549 |
| H | -0.894204118 | 1.019298333 | 3.245500658 |
| H | -0.781829699 | 1.950288390 | 1.734105093 |
| H | -1.998539231 | 0.683555315 | 1.902229519 |
| P | 2.541915854 | 1.906314827 | 3.258273966 |
| C | 2.902693298 | 1.835692915 | 5.026252071 |
| C | 4.091619032 | 2.368078422 | 5.553660031 |
| H | 4.827946129 | 2.823651625 | 4.897363777 |
| C | 4.325775245 | 2.303704070 | 6.925719466 |
| H | 5.243759398 | 2.712835632 | 7.336673076 |
| C | 3.380960061 | 1.712503519 | 7.769476405 |
| H | 3.567355469 | 1.665577153 | 8.838353764 |
| C | 2.203311864 | 1.177646572 | 7.244074891 |
| H | 1.474245680 | 0.713846226 | 7.901584095 |
| C | 1.958867285 | 1.230944754 | 5.871630833 |
| H | 1.051690864 | 0.796255098 | 5.460901300 |
| C | 4.021255570 | 1.373768554 | 2.315002926 |
| C | 4.288136159 | 1.845222557 | 1.005036559 |
| H | 3.782574690 | 2.733765646 | 0.640867799 |
| C | 5.208873584 | 1.184543327 | 0.196812521 |
| H | 5.411169977 | 1.559499941 | -0.801547109 |
| C | 5.873950536 | 0.042051087 | 0.660563073 |
| H | 6.591707927 | -0.462229949 | 0.021565304 |
| C | 5.617498787 | -0.441792166 | 1.942705768 |
| H | 6.141172661 | -1.318434248 | 2.312084039 |
| C | 4.684684931 | 0.199659368 | 2.768071690 |
| H | 4.527240592 | -0.153155477 | 3.783432869 |
| C | 2.037472987 | 3.578142088 | 2.781230435 |
| C | 2.195747070 | 4.676286907 | 3.639671092 |
| H | 2.631784208 | 4.544488939 | 4.624675785 |
| C | 1.783847642 | 5.942158092 | 3.225536108 |
| H | 1.906841103 | 6.790997492 | 3.891165397 |
| C | 1.214043859 | 6.119387286 | 1.963208770 |
| H | 0.892970428 | 7.107576640 | 1.648017393 |
| C | 1.046079257 | 5.026917160 | 1.108724187 |
| H | 0.594262706 | 5.163055341 | 0.130803511 |
| C | 1.446869211 | 3.757391121 | 1.518084284 |
| H | 1.304170186 | 2.902692818 | 0.862279090 |
| O | -0.433995146 | -0.767109154 | 4.520520634 |
| O | 0.074915025 | -2.875312596 | 3.905028066 |
| C | -0.289498059 | -3.323530827 | 5.231885527 |
| H | -0.139732234 | -4.402092383 | 5.224066246 |
| H | 0.349042025 | -2.845657600 | 5.978978037 |
| H | -1.332788244 | -3.075954060 | 5.439240640 |

==============================

25

==============================

| | | | |
|---|---|---|---|
| Rh | 0.545618397 | 1.040419355 | -0.407019467 |
| P | -1.383807151 | 2.335991741 | 0.106776766 |
| P | -0.319145517 | 0.341646547 | -2.526900450 |
| C | 2.688374684 | 0.334574590 | -0.982015199 |
| C | 2.054854737 | -0.760631543 | -0.444328826 |
| C | 1.582072996 | 2.145355585 | 1.237582585 |
| C | 1.233886905 | 1.073084543 | 1.745617101 |
| C | 1.086389240 | -0.155286708 | 2.557525932 |
| O | 1.215587080 | -1.340593371 | 1.793723208 |
| C | 2.354395183 | -1.325164874 | 0.934352593 |
| H | 1.842946525 | -0.122153148 | 3.359044208 |
| H | 0.099803874 | -0.193853945 | 3.029094762 |
| H | 2.656244141 | -2.371244542 | 0.816764365 |
| H | 3.190202210 | -0.788674169 | 1.402865849 |
| H | 1.513047894 | -1.445984102 | -1.092345344 |
| H | 2.000748679 | 3.122489652 | 1.099041821 |
| H | 3.394896856 | 0.902920911 | -0.384260377 |
| H | 2.722946300 | 0.520068439 | -2.048429718 |
| C | 0.902694541 | -0.300959518 | -3.757932603 |
| C | -1.496640022 | -1.073119620 | -2.375214502 |
| C | -1.223624496 | 1.554701211 | -3.588938835 |
| C | -1.550298528 | 2.819970435 | 1.879655273 |
| C | -1.458695658 | 3.971546402 | -0.748326203 |
| C | -3.052101745 | 1.600261067 | -0.198100432 |
| H | -1.487760424 | 3.841270293 | -1.831531733 |
| H | -2.345288346 | 4.530590429 | -0.433302747 |

| | | | |
|---|---|---|---|
| H | -0.566786282 | 4.552169732 | -0.499363358 |
| H | -1.606074324 | 1.928695730 | 2.509308896 |
| H | -0.694103994 | 3.414478000 | 2.200555141 |
| H | -2.464279241 | 3.405712534 | 2.015697544 |
| H | -3.216220092 | 1.394865224 | -1.256927733 |
| H | -3.141876403 | 0.661589119 | 0.355544461 |
| H | -3.832228847 | 2.286228068 | 0.145631406 |
| H | -2.115190801 | 1.938563814 | -3.091272854 |
| H | -0.566694952 | 2.395938147 | -3.825816150 |
| H | -1.529922890 | 1.075510642 | -4.523923897 |
| H | 1.560966893 | 0.505974315 | -4.090830908 |
| H | 1.514113053 | -1.102087656 | -3.337592295 |
| H | 0.370095108 | -0.693717710 | -4.629116390 |
| H | -2.350671146 | -0.795065788 | -1.755269400 |
| H | -1.856222951 | -1.386064514 | -3.360293652 |
| H | -0.994130149 | -1.917621788 | -1.896421265 |

==============================

26

==============================

| | | | |
|---|---|---|---|
| Rh | 0.887941042 | 1.248168281 | -0.584106147 |
| P | -0.617498700 | 2.558562998 | 0.676876495 |
| P | -0.826063814 | 0.222087892 | -1.899105131 |
| C | 2.583067752 | 0.514214413 | -2.090743698 |
| C | 2.276978372 | -0.388417473 | -1.311222065 |
| C | 2.568706502 | 2.456477511 | 0.213266644 |
| C | 2.486360723 | 1.524580116 | 1.021088818 |
| C | 2.672973802 | 0.569323230 | 2.123031914 |
| H | 3.034172718 | -0.393122913 | 1.724071682 |
| H | 3.462395396 | 0.962908246 | 2.785700046 |
| H | 2.896532294 | 3.370883458 | -0.240634400 |
| C | -0.294208303 | -0.387493417 | -3.559560068 |
| C | -1.465804964 | -1.313113898 | -1.094309483 |
| C | -2.376464425 | 1.118769915 | -2.361442892 |
| C | 0.097771606 | 3.426815731 | 2.135551722 |
| C | -1.427332672 | 3.944524536 | -0.238583029 |
| C | -2.015726540 | 1.647221643 | 1.467299715 |
| C | 3.101227306 | 1.433765890 | -3.103037619 |
| H | 2.248139068 | -1.342149856 | -0.824437744 |
| O | 1.454627278 | 0.402524162 | 2.827458477 |
| C | 1.624626215 | -0.373678612 | 4.026246594 |
| C | 0.279747268 | -0.617352859 | 4.637880047 |
| H | 2.109540068 | -1.329959458 | 3.768295133 |
| H | 2.283775599 | 0.158327937 | 4.728172888 |
| H | -0.424692441 | -1.179115231 | 4.025309959 |
| H | -0.017916862 | 0.458230283 | -4.195405861 |
| H | 0.570716611 | -1.044711437 | -3.463964015 |
| H | -1.112577766 | -0.932909273 | -4.039456340 |
| H | -1.909910979 | -1.076528391 | -0.124821585 |
| H | -2.218999958 | -1.796075903 | -1.724906202 |
| H | -0.641510599 | -2.011562858 | -0.929563904 |
| H | -2.936131424 | 1.435835866 | -1.479497427 |
| H | -2.128765637 | 2.003471106 | -2.954588492 |
| H | -3.019103563 | 0.467440593 | -2.962020644 |
| H | -2.629589620 | 1.127694515 | 0.728915772 |
| H | -1.599146463 | 0.910988352 | 2.159872965 |
| H | -2.653264136 | 2.339017572 | 2.027082488 |
| H | -2.005392381 | 3.574933139 | -1.086641415 |
| H | -2.091651624 | 4.505777250 | 0.426231985 |
| H | -0.656865384 | 4.620821177 | -0.619064427 |
| H | 0.531004037 | 2.686956919 | 2.812225713 |
| H | 0.879785566 | 4.123058975 | 1.826334597 |
| H | -0.691490400 | 3.981289301 | 2.652492471 |
| H | 2.298277187 | 1.830762943 | -3.731526913 |
| H | 3.618061172 | 2.278946737 | -2.637515938 |
| H | 3.816737128 | 0.909950764 | -3.747209107 |
| C | -0.063355787 | -0.218876742 | 5.862884182 |
| H | 0.623734538 | 0.334841537 | 6.498402934 |
| H | -1.038568560 | -0.444550243 | 6.283656466 |

==============================

26A

==============================

| | | | |
|---|---|---|---|
| Rh | 0.738474246 | 1.134145459 | -0.606878672 |
| P | -0.666078920 | 2.593605669 | 0.580975074 |
| P | -0.989881435 | 0.343029921 | -2.038996891 |
| C | 2.297467521 | -0.049632857 | -1.844776233 |
| C | 1.987828626 | -0.844321042 | -0.959426690 |
| C | 2.458301415 | 2.452211093 | -0.134667263 |
| C | 2.493018752 | 1.645628226 | 0.801421717 |
| C | 2.950058739 | 0.926308074 | 2.003123526 |
| H | 3.664154487 | 0.140385348 | 1.704178762 |
| H | 3.502702307 | 1.638044207 | 2.638129699 |
| H | 2.677415682 | 3.257630539 | -0.807139803 |
| C | -0.376522577 | -0.416887815 | -3.605816661 |
| C | -2.056905596 | -0.997489842 | -1.344670385 |
| C | -2.218101774 | 1.550500952 | -2.710424613 |
| C | 0.041768458 | 3.188262172 | 2.173680974 |
| C | -1.130441950 | 4.167676941 | -0.265155020 |
| C | -2.284182760 | 1.904354582 | 1.149216745 |
| H | 2.744615795 | 0.425307566 | -2.692368722 |
| C | 1.772607918 | -1.932899909 | -0.000855933 |
| O | 1.854172493 | 0.368987149 | 2.708194328 |
| C | 2.254026486 | -0.171212088 | 3.980362531 |
| C | 1.055152355 | -0.765852458 | 4.651625315 |
| H | 3.030123672 | -0.937696404 | 3.816328046 |
| H | 2.695034713 | 0.621493625 | 4.602693109 |
| H | 0.541979674 | -1.551330562 | 4.098166583 |
| H | 0.865394442 | -2.497417170 | -0.241281174 |
| H | 2.620040198 | -2.626892716 | -0.035652509 |
| H | 1.672974384 | -1.542413392 | 1.016340420 |
| H | 0.161984943 | 0.331557639 | -4.193572085 |
| H | 0.306899960 | -1.238864212 | -3.388068554 |
| H | -1.217788062 | -0.791959043 | -4.196645724 |
| H | -2.570695196 | -0.653521649 | -0.444670365 |
| H | -2.802025987 | -1.318294909 | -2.079610065 |
| H | -1.437143524 | -1.856695233 | -1.075935339 |
| H | -2.818856155 | 1.998444781 | -1.916805952 |
| H | -1.691293072 | 2.348641487 | -3.240944696 |
| H | -2.891015935 | 1.049298924 | -3.412944169 |
| H | -2.924627844 | 1.630317791 | 0.308469629 |
| H | -2.100490230 | 1.012192048 | 1.754317570 |
| H | -2.814312815 | 2.640931548 | 1.761519965 |
| H | -1.670869448 | 3.967297159 | -1.192076326 |
| H | -1.757213281 | 4.785122950 | 0.385648888 |
| H | -0.223462557 | 4.725630541 | -0.512492480 |
| H | 0.344267362 | 2.327190396 | 2.775010254 |
| H | 0.919509484 | 3.809612612 | 1.989060313 |
| H | -0.706568996 | 3.774555428 | 2.715860912 |
| C | 0.632165177 | -0.410860491 | 5.864859760 |
| H | 1.130942301 | 0.364398681 | 6.441700690 |
| H | -0.219098067 | -0.893545631 | 6.334972292 |

==============================

26-TS

==============================

| | | | |
|---|---|---|---|
| C | -0.433131020 | -0.344030271 | 0.225679396 |
| C | -0.069105749 | 0.383057919 | 2.189530055 |
| Rh | 1.703719671 | -0.261754054 | 1.070135404 |
| P | 2.769723117 | -1.241190708 | 3.008475733 |
| P | 3.636862792 | 0.399703870 | -0.159165978 |
| C | 0.470618459 | 0.097503500 | -0.569449169 |
| C | 0.906010527 | 1.222677750 | 2.216625234 |
| C | 1.356231506 | 2.588829907 | 2.552259847 |
| H | 1.159431938 | 3.249931119 | 1.688929673 |
| H | 0.729146370 | 2.953200550 | 3.384549441 |
| H | -0.960730154 | 0.098984401 | 2.725790556 |
| C | 3.859198122 | -0.478447130 | -1.768618887 |
| C | 3.528337297 | 2.175711740 | -0.638116720 |
| C | 5.329913374 | 0.307988991 | 0.569713155 |
| C | 1.567773151 | -1.669418750 | 4.340893471 |

| | | | |
|---|---|---|---|
| C | 3.518661154 | -2.879348707 | 2.594122335 |
| C | 4.099883943 | -0.371169491 | 3.942452047 |
| C | -1.753018726 | -0.996518143 | 0.416318935 |
| H | 0.574566259 | 0.320718771 | -1.620442030 |
| O | 2.727954563 | 2.610052902 | 2.899260313 |
| C | 3.125403628 | 3.881330592 | 3.444828268 |
| C | 4.594327449 | 3.849585584 | 3.733637912 |
| H | 2.890981379 | 4.674691068 | 2.715683490 |
| H | 2.553913651 | 4.090146460 | 4.361208648 |
| H | 5.245875115 | 3.683008594 | 2.876513738 |
| H | 4.070776367 | -1.536392558 | -1.589282617 |
| H | 2.946865621 | -0.409404021 | -2.365695261 |
| H | 4.688439914 | -0.044788397 | -2.336241230 |
| H | 3.483770100 | 2.776750443 | 0.273386623 |
| H | 4.399751760 | 2.474678366 | -1.229471409 |
| H | 2.619659280 | 2.350221112 | -1.218713846 |
| H | 5.374326420 | 0.931771077 | 1.465215913 |
| H | 5.580089228 | -0.721255539 | 0.840166931 |
| H | 6.072360790 | 0.667859569 | -0.149597568 |
| H | 5.017195324 | -0.342031815 | 3.351066705 |
| H | 3.788077161 | 0.656819800 | 4.141204132 |
| H | 4.308202530 | -0.888197966 | 4.884226393 |
| H | 4.329654196 | -2.753074289 | 1.871746558 |
| H | 3.920247664 | -3.364360026 | 3.489485679 |
| H | 2.762116045 | -3.532678119 | 2.150103568 |
| H | 1.111670824 | -0.757063355 | 4.732463770 |
| H | 0.776644270 | -2.306341575 | 3.936446175 |
| H | 2.067439235 | -2.196268537 | 5.159590186 |
| H | -1.670782011 | -1.911937267 | 1.009233703 |
| H | -2.478325723 | -0.332739291 | 0.895426428 |
| H | -2.136785603 | -1.265902227 | -0.573869259 |
| C | 5.116771593 | 4.043984207 | 4.944758481 |
| H | 6.190054640 | 4.047884584 | 5.108957863 |
| H | 4.491411622 | 4.223382999 | 5.816191852 |

==============================

26A-TS

==============================

| | | | |
|---|---|---|---|
| C | -0.382136388 | -0.380417982 | 0.365818148 |
| C | -0.019259970 | 0.429886598 | 2.245951587 |
| Rh | 1.670723566 | -0.245371362 | 1.052258713 |
| P | 2.718745105 | -1.301687648 | 2.980061204 |
| P | 3.666330549 | 0.419414402 | -0.099639512 |
| C | 0.361446125 | 0.081276376 | -0.576541992 |
| C | 0.962711027 | 1.260328265 | 2.243979780 |
| C | 1.431873103 | 2.626857708 | 2.549540434 |
| H | 1.227035491 | 3.280852953 | 1.683069716 |
| H | 0.824221135 | 3.006660959 | 3.389189937 |
| H | -0.912821930 | 0.142159427 | 2.776685293 |
| C | 4.037593233 | -0.517446799 | -1.648039300 |
| C | 3.571144721 | 2.174688237 | -0.653407963 |
| C | 5.296765382 | 0.402244706 | 0.764675668 |
| C | 1.471267474 | -1.869845115 | 4.215161581 |
| C | 3.570801059 | -2.876182295 | 2.522375583 |
| C | 3.945736127 | -0.414824188 | 4.032781210 |
| H | -1.306011350 | -0.856217643 | 0.652003510 |
| C | 0.335016225 | 0.400338616 | -2.027633699 |
| O | 2.810206309 | 2.635904074 | 2.870966024 |
| C | 3.230560348 | 3.908912665 | 3.396733548 |
| C | 4.710512169 | 3.879053334 | 3.621356548 |
| H | 2.964233776 | 4.700337489 | 2.676626564 |
| H | 2.697971975 | 4.118545207 | 4.335939123 |
| H | 5.324614013 | 3.722136121 | 2.735227792 |
| H | 4.205539463 | -1.572518261 | -1.413728456 |
| H | 3.194745407 | -0.450081646 | -2.339128663 |
| H | 4.931023395 | -0.118747594 | -2.138624742 |
| H | 3.492475504 | 2.807639464 | 0.233669874 |
| H | 4.465341741 | 2.453682871 | -1.219616820 |
| H | 2.688679398 | 2.331360465 | -1.276804842 |
| H | 5.240309176 | 1.037959501 | 1.651074789 |
| H | 5.563781889 | -0.613008489 | 1.069315890 |
| H | 6.080509458 | 0.781855156 | 0.101478740 |
| H | 4.903586010 | -0.343445233 | 3.513827549 |
| H | 3.590597634 | 0.599940485 | 4.226840136 |
| H | 4.098100596 | -0.946498908 | 4.976887429 |
| H | 4.408988059 | -2.676022387 | 1.849736660 |
| H | 3.950865303 | -3.387762531 | 3.412421434 |
| H | 2.868891825 | -3.539837478 | 2.009075148 |
| H | 0.960700180 | -1.006018791 | 4.647462339 |
| H | 0.727708252 | -2.505806432 | 3.727440167 |
| H | 1.952730106 | -2.435265427 | 5.018944482 |
| C | 5.284874741 | 4.065691024 | 4.809862069 |
| H | 6.364293716 | 4.071070326 | 4.926869085 |
| H | 4.697705407 | 4.236340918 | 5.709141507 |
| H | 0.828055503 | 1.353004221 | -2.245400104 |
| H | 0.856593754 | -0.374280631 | -2.600181914 |
| H | -0.693136147 | 0.455605977 | -2.398556616 |

==============================

27

==============================

| | | | |
|---|---|---|---|
| C | 0.971477542 | 2.331013137 | -0.557011739 |
| C | 2.321570324 | 1.777319862 | -0.420386288 |
| C | -0.049249253 | 1.464063056 | -0.412435802 |
| Rh | 0.542709189 | -0.396969607 | 0.019844612 |
| C | 2.346360131 | 0.455116401 | -0.144381221 |
| C | -1.515931098 | 1.696569980 | -0.556002840 |
| H | 0.805140012 | 3.387380208 | -0.769623352 |
| C | 3.552309978 | 2.635524071 | -0.586022429 |
| H | 3.238569107 | -0.160534933 | -0.024168492 |
| P | 0.530770558 | 0.054721618 | 2.370782777 |
| P | 0.596589334 | -1.035797796 | -2.286961093 |
| H | -1.727802768 | 2.780022403 | -0.539754653 |
| H | -1.878595306 | 1.313994842 | -1.527207393 |
| O | -2.198324711 | 1.024223872 | 0.499076178 |
| C | -3.627451762 | 1.107252080 | 0.373244916 |
| C | -4.264379582 | 0.519074239 | 1.594564748 |
| H | -3.915160411 | 2.165488906 | 0.265405640 |
| H | -3.957533621 | 0.576862573 | -0.533419426 |
| C | -5.075184958 | -0.539818795 | 1.575510925 |
| H | -4.053549336 | 1.030460920 | 2.533007037 |
| H | -5.316493685 | -1.059635709 | 0.650808071 |
| H | -5.543937783 | -0.915825301 | 2.479512667 |
| C | 2.062880935 | -0.495231206 | 3.234399355 |
| C | 0.295530324 | 1.791381458 | 2.912174726 |
| C | -0.814815843 | -0.878458350 | 3.216799243 |
| C | 1.805071725 | -2.396784306 | -2.584418652 |
| C | -0.997687277 | -1.723019570 | -2.911277520 |
| C | 1.051093219 | 0.245590735 | -3.522153997 |
| H | -1.778360003 | -0.596097446 | 2.786468024 |
| H | -0.668737102 | -1.953585011 | 3.077463399 |
| H | -0.816713903 | -0.662660925 | 4.290064946 |
| H | -0.678313002 | 2.137638173 | 2.560884664 |
| H | 0.341948662 | 1.857879940 | 4.003112791 |
| H | 1.072155452 | 2.421872946 | 2.474573106 |
| H | 2.243229134 | -1.556867753 | 3.041782232 |
| H | 2.916014208 | 0.076424192 | 2.862090795 |
| H | 1.972663874 | -0.345544114 | 4.314867098 |
| H | -1.779583605 | -0.961049311 | -2.855720006 |
| H | -0.897943569 | -2.050714611 | -3.950665970 |
| H | -1.303337235 | -2.576795626 | -2.300208639 |
| H | 0.313599607 | 1.051192746 | -3.509775580 |
| H | 2.023450819 | 0.668181404 | -3.258546313 |
| H | 1.099684833 | -0.186937216 | -4.525859074 |
| H | 2.811768079 | -2.054910940 | -2.330302008 |
| H | 1.562090500 | -3.260691444 | -1.959056345 |
| H | 1.789670334 | -2.708409574 | -3.633380828 |
| H | 4.467754395 | 2.051555348 | -0.458428275 |
| H | 3.580948773 | 3.099062669 | -1.579532594 |
| H | 3.567063389 | 3.451223413 | 0.146815335 |

==============================

28

==============================

| | | | |
|---|---|---|---|
| C | 1.183674567 | 2.325964592 | -0.613179516 |
| C | 2.489333263 | 1.688356501 | -0.504040186 |
| C | 0.117988815 | 1.525136496 | -0.423045702 |
| Rh | 0.634785120 | -0.358860950 | 0.022368788 |
| C | 2.500948030 | 0.366989097 | -0.221710053 |
| C | -1.345140316 | 1.814832506 | -0.520701547 |
| H | 1.081160230 | 3.388142730 | -0.834890017 |
| H | 3.411917263 | 2.247992535 | -0.655549294 |
| C | 3.682810925 | -0.537356376 | -0.073172140 |
| P | 0.629270695 | 0.131818021 | 2.375153481 |
| P | 0.489636840 | -1.008131436 | -2.278377593 |
| H | -1.543056198 | 2.881037437 | -0.315810315 |
| H | -1.723008860 | 1.608748772 | -1.538442711 |
| O | -2.033750527 | 0.980862147 | 0.411871218 |
| C | -3.463201040 | 1.074686684 | 0.280845266 |
| C | -4.116096180 | 0.282376702 | 1.370543097 |
| H | -3.755907004 | 2.134960436 | 0.350208378 |
| H | -3.774319116 | 0.706875859 | -0.708794132 |
| C | -4.938308258 | -0.745084810 | 1.152735060 |
| H | -3.913565723 | 0.614375766 | 2.388328068 |
| H | -5.173559742 | -1.086973660 | 0.147200676 |
| H | -5.422729813 | -1.271526584 | 1.969570469 |
| C | 2.171572433 | -0.268933672 | 3.301156989 |
| C | 0.247992465 | 1.855634907 | 2.876546060 |
| C | -0.661434299 | -0.884494046 | 3.214549266 |
| C | 1.396301361 | -2.566373350 | -2.671897217 |
| C | -1.243439740 | -1.401700020 | -2.776565947 |
| C | 1.072137796 | 0.177370930 | -3.555741907 |
| H | -1.634330862 | -0.676999661 | 2.762306536 |
| H | -0.438441149 | -1.948445844 | 3.091520224 |
| H | -0.702379979 | -0.656523303 | 4.284477328 |
| H | -0.738903122 | 2.123196998 | 2.493388104 |
| H | 0.256962481 | 1.945759431 | 3.967160701 |
| H | 0.988517535 | 2.534869055 | 2.448249974 |
| H | 2.432504093 | -1.322635508 | 3.167660357 |
| H | 2.995021506 | 0.346254847 | 2.930520636 |
| H | 2.036363926 | -0.075980274 | 4.370103012 |
| H | -1.880122413 | -0.522884386 | -2.649362867 |
| H | -1.282786411 | -1.720639635 | -3.822904629 |
| H | -1.636583866 | -2.205309692 | -2.147203249 |
| H | 0.492086541 | 1.100594420 | -3.494469374 |
| H | 2.120214729 | 0.424021984 | -3.370953338 |
| H | 0.967320859 | -0.254786301 | -4.555443248 |
| H | 2.470985035 | -2.420959489 | -2.535999687 |
| H | 1.067617186 | -3.372959797 | -2.010056649 |
| H | 1.210679199 | -2.865298041 | -3.708536218 |
| H | 3.665097632 | -1.368849757 | -0.789142664 |
| H | 4.611001557 | 0.024075888 | -0.242750204 |
| H | 3.743729606 | -0.983201324 | 0.927053540 |

==============================

8A-TS1’

==============================

| | | | |
|---|---|---|---|
| C | 0.049888282 | 0.062092706 | -0.275007081 |
| C | -0.399948249 | 0.396798025 | 1.968571040 |
| C | 2.652193027 | -0.148646561 | 0.550698292 |
| C | 0.601741987 | -0.030430103 | 2.615179911 |
| Rh | 0.973559304 | -1.343806196 | 0.971236424 |
| P | 1.750665899 | -3.117474417 | 2.586998533 |
| P | 1.175388329 | -2.690757476 | -0.990152352 |
| C | 0.849938006 | 1.079854371 | -0.611603691 |
| C | 2.181460245 | 1.214498643 | 0.073040465 |
| C | 2.992855782 | 1.905826979 | -1.048805164 |
| C | 0.791240272 | 2.098396576 | -1.728954991 |
| O | 2.052984480 | 2.772999237 | -1.679415853 |
| H | 3.191565986 | -0.100374229 | 1.493726307 |
| H | 3.220462493 | -0.687167506 | -0.208564952 |
| H | -0.899573390 | -0.137227184 | -0.768441974 |
| H | 0.638626053 | 1.604627150 | -2.699371409 |
| H | 0.000456213 | 2.848226356 | -1.595746464 |
| H | 3.825902048 | 2.515194688 | -0.690094950 |
| H | 3.377863628 | 1.151327883 | -1.755566577 |
| H | 2.086283720 | 1.904830971 | 0.922743717 |
| C | 1.167306449 | 0.161677116 | 3.958397296 |
| O | 0.519037520 | 0.520130390 | 4.917106144 |
| O | 2.485321336 | -0.117658657 | 3.987116619 |
| C | 3.116229825 | 0.022509897 | 5.278000887 |
| H | 3.115210050 | 1.070416636 | 5.587755978 |
| H | 2.582507430 | -0.573809514 | 6.021157319 |
| H | 4.132831706 | -0.344880538 | 5.142376530 |
| C | 0.882122044 | -1.810842081 | -2.580526495 |
| C | 1.956884784 | -1.145482535 | -3.193618454 |
| C | -0.395582731 | -1.702976512 | -3.146541729 |
| C | 1.757603590 | -0.395022583 | -4.351353805 |
| C | -0.591718405 | -0.946670451 | -4.304693038 |
| C | 0.480736762 | -0.289833130 | -4.910144480 |
| H | 2.956981341 | -1.227119574 | -2.777919047 |
| H | -1.239800621 | -2.215812061 | -2.696702231 |
| H | 2.599707284 | 0.110083992 | -4.815329763 |
| H | -1.586042114 | -0.879329738 | -4.737014829 |
| H | 0.325494952 | 0.294383750 | -5.812088950 |
| C | 2.761787638 | -3.538354123 | -1.331291311 |
| C | 2.946827763 | -4.284047164 | -2.509145525 |
| C | 3.839390464 | -3.391688824 | -0.449037886 |
| C | 4.166710057 | -4.908919011 | -2.762303816 |
| C | 5.065125458 | -4.006822295 | -0.709436244 |
| C | 5.228620674 | -4.776042647 | -1.861770768 |
| H | 2.142879028 | -4.363555433 | -3.235010134 |
| H | 3.723247095 | -2.806716610 | 0.453265845 |
| H | 4.292683514 | -5.491922025 | -3.669959407 |
| H | 5.884206895 | -3.883270291 | -0.006476169 |
| H | 6.179138214 | -5.259487381 | -2.066604133 |
| C | -0.170904302 | -3.930418869 | -0.896757031 |
| C | -1.394280512 | -3.485491214 | -0.360407488 |
| C | -0.061910766 | -5.255804334 | -1.332809931 |
| C | -2.491343813 | -4.342001777 | -0.286939578 |
| C | -1.162560168 | -6.109801771 | -1.256593916 |
| C | -2.377643618 | -5.656580152 | -0.742141223 |
| H | -1.497175748 | -2.459079479 | -0.012400269 |
| H | 0.880727899 | -5.635100813 | -1.709532232 |
| H | -3.431136067 | -3.982572242 | 0.122772008 |
| H | -1.064824147 | -7.137825803 | -1.592676552 |
| H | -3.230250486 | -6.327147216 | -0.687510318 |
| C | 1.372865545 | -4.867039865 | 2.093276308 |
| C | 0.122757872 | -5.450803273 | 2.347724687 |
| C | 2.355575441 | -5.638312890 | 1.450431539 |
| C | -0.131405013 | -6.771358837 | 1.980055459 |
| C | 2.097407460 | -6.957778356 | 1.083635448 |
| C | 0.854025164 | -7.530992573 | 1.350647780 |
| H | -0.655309415 | -4.894352695 | 2.855424094 |
| H | 3.337213441 | -5.225626551 | 1.255604647 |
| H | -1.103618605 | -7.205737035 | 2.193364503 |
| H | 2.876612703 | -7.538450305 | 0.598092255 |
| H | 0.655430903 | -8.562320997 | 1.073451873 |
| C | 3.471121830 | -3.274135729 | 3.212535933 |
| C | 3.863821374 | -4.402167102 | 3.955392609 |

| | | | |
|---|---|---|---|
| C | 4.415148621 | -2.270595820 | 2.959703401 |
| C | 5.167391934 | -4.514222532 | 4.434934490 |
| C | 5.723239333 | -2.388105587 | 3.432802809 |
| C | 6.101218411 | -3.508847202 | 4.172804099 |
| H | 3.151275407 | -5.196625007 | 4.153357309 |
| H | 4.129235280 | -1.391735486 | 2.400311341 |
| H | 5.454904882 | -5.389571204 | 5.010022033 |
| H | 6.443891508 | -1.603046804 | 3.221900970 |
| H | 7.118449094 | -3.601217210 | 4.542137337 |
| C | 0.707476450 | -2.914770521 | 4.095137129 |
| C | -0.665812644 | -2.668765923 | 3.917237661 |
| C | 1.215565533 | -2.989123756 | 5.398379041 |
| C | -1.512337196 | -2.533024665 | 5.015413587 |
| C | 0.368698663 | -2.827942454 | 6.496640578 |
| C | -0.994655924 | -2.605521649 | 6.309801494 |
| H | -1.081613472 | -2.581325677 | 2.916036572 |
| H | 2.272431506 | -3.165614869 | 5.564420518 |
| H | -2.573714747 | -2.360668485 | 4.860248487 |
| H | 0.778800164 | -2.879392326 | 7.501125717 |
| H | -1.650230532 | -2.484063470 | 7.166802347 |
| C | -1.755915267 | 0.857054825 | 1.918369706 |
| C | -2.286925563 | 1.694324047 | 0.920618638 |
| C | -2.556848864 | 0.485424913 | 3.021454735 |
| C | -3.602432822 | 2.134972366 | 1.017483437 |
| C | -3.869397809 | 0.936455424 | 3.107375413 |
| C | -4.396678127 | 1.753112149 | 2.103351504 |
| H | -1.660769263 | 2.002715821 | 0.092846038 |
| H | -2.120605838 | -0.122378529 | 3.806615854 |
| H | -4.009453558 | 2.785666916 | 0.249383736 |
| H | -4.480276813 | 0.656157264 | 3.960604870 |
| H | -5.423112917 | 2.101537240 | 2.171775139 |

==============================

8B-TS1’

==============================

| | | | |
|---|---|---|---|
| C | -0.067223298 | -0.110650562 | 0.000371616 |
| C | 0.077328759 | -0.147507909 | 2.255334516 |
| C | 2.695681439 | 0.037316876 | 0.019178245 |
| C | 1.274530223 | 0.056037526 | 2.678260523 |
| Rh | 1.400842134 | -1.283029078 | 1.008805521 |
| P | 3.218615808 | -2.564832278 | 2.007819855 |
| P | 0.742479471 | -2.804720496 | -0.829816787 |
| C | 0.485490345 | 0.997748532 | -0.492859658 |
| C | 1.935121746 | 1.319712038 | -0.226957704 |
| C | 2.278589558 | 2.104712752 | -1.519635220 |
| C | -0.020206221 | 1.977382443 | -1.533747599 |
| O | 1.091002176 | 2.831872707 | -1.810742628 |
| H | 3.581755968 | 0.140056256 | 0.645187729 |
| H | 2.934440943 | -0.480362785 | -0.909300373 |
| H | -1.081554025 | -0.437571217 | -0.186984836 |
| H | -0.350280796 | 1.443082172 | -2.437707466 |
| H | -0.848853699 | 2.596621909 | -1.173785909 |
| H | 3.095539375 | 2.820355423 | -1.401299721 |
| H | 2.526844695 | 1.400386622 | -2.331166782 |
| H | 2.016164297 | 1.992433042 | 0.638442710 |
| C | -1.309856956 | 0.015680856 | 2.700301343 |
| O | -1.546756583 | 0.331866159 | 3.852257533 |
| O | -2.253604543 | -0.263905434 | 1.790525411 |
| C | -3.615339736 | -0.155495177 | 2.259211172 |
| H | -3.772269298 | -0.799401494 | 3.128556112 |
| H | -3.839479847 | 0.877504580 | 2.536477232 |
| H | -4.233092693 | -0.477265196 | 1.421298280 |
| C | 0.652252622 | -1.923189572 | -2.436292056 |
| C | 1.847681233 | -1.574274165 | -3.088660309 |
| C | -0.564772026 | -1.500373933 | -2.989550933 |
| C | 1.825650347 | -0.833363965 | -4.269439834 |
| C | -0.583784769 | -0.757425015 | -4.171406226 |
| C | 0.608178707 | -0.421528877 | -4.813966009 |
| H | 2.805970810 | -1.891040263 | -2.688324920 |
| H | -1.501283113 | -1.745410870 | -2.500638757 |
| H | 2.759099211 | -0.578778131 | -4.763006819 |
| H | -1.535175148 | -0.439679489 | -4.588152915 |
| H | 0.589677720 | 0.160116205 | -5.730500952 |
| C | 1.527339129 | -4.424001415 | -1.219988617 |
| C | 1.570708900 | -5.357648318 | -0.173760858 |
| C | 2.029495263 | -4.781186799 | -2.477203348 |
| C | 2.142748549 | -6.610668483 | -0.368473540 |
| C | 2.598971749 | -6.041523024 | -2.669552388 |
| C | 2.666763001 | -6.953628360 | -1.617226424 |
| H | 1.149337837 | -5.108282357 | 0.794109748 |
| H | 1.978027300 | -4.090129889 | -3.310719310 |
| H | 2.173112088 | -7.319685614 | 0.453980512 |
| H | 2.985856531 | -6.309179311 | -3.648739895 |
| H | 3.113798810 | -7.931222273 | -1.771793847 |
| C | -0.959098939 | -3.384203900 | -0.448677128 |
| C | -1.674336852 | -4.148428312 | -1.387053777 |
| C | -1.517634298 | -3.161334091 | 0.815088864 |
| C | -2.934514207 | -4.648697630 | -1.071464656 |
| C | -2.775813397 | -3.674185165 | 1.134424865 |
| C | -3.486512285 | -4.415171004 | 0.191954951 |
| H | -1.240743529 | -4.355378812 | -2.361348856 |
| H | -0.975540263 | -2.586243681 | 1.556712690 |
| H | -3.482130731 | -5.231771678 | -1.806071251 |
| H | -3.194355163 | -3.496226612 | 2.120862444 |
| H | -4.465996525 | -4.814834834 | 0.438251233 |
| C | 4.426144519 | -3.411998822 | 0.925996113 |
| C | 5.439351344 | -4.215849657 | 1.479815701 |
| C | 4.392449764 | -3.221940869 | -0.457841337 |
| C | 6.360335637 | -4.852342654 | 0.652566983 |
| C | 5.319872062 | -3.853143003 | -1.286779198 |
| C | 6.298458192 | -4.676530395 | -0.733179304 |
| H | 5.514320682 | -4.330242925 | 2.557183760 |
| H | 3.636562993 | -2.577589150 | -0.885061299 |
| H | 7.133322108 | -5.478358295 | 1.088635425 |
| H | 5.271858637 | -3.705331375 | -2.361503110 |
| H | 7.019655652 | -5.172886486 | -1.376006341 |
| C | 2.327753976 | -3.855752167 | 2.969759985 |
| C | 1.233070781 | -3.448281975 | 3.754592079 |
| C | 2.657467517 | -5.218956363 | 2.924081161 |
| C | 0.494226024 | -4.381277556 | 4.481449390 |
| C | 1.910816804 | -6.148473942 | 3.647418603 |
| C | 0.830412964 | -5.734282673 | 4.428242977 |
| H | 0.957633172 | -2.399764131 | 3.805885478 |
| H | 3.475067935 | -5.564829827 | 2.302878646 |
| H | -0.343043702 | -4.048020271 | 5.087736124 |
| H | 2.176203149 | -7.200813887 | 3.599864321 |
| H | 0.252820823 | -6.462473921 | 4.989436178 |
| C | 4.358278855 | -1.783101990 | 3.222461641 |
| C | 4.128870744 | -1.823993070 | 4.604653042 |
| C | 5.507403224 | -1.132676261 | 2.741217670 |
| C | 5.032371638 | -1.229439287 | 5.485937187 |
| C | 6.408544357 | -0.543418657 | 3.626357433 |
| C | 6.174875441 | -0.590204958 | 5.002795833 |

| | | | |
|---|---|---|---|
| H | 3.252759483 | -2.324010109 | 5.002966932 |
| H | 5.715408000 | -1.108845695 | 1.674943104 |
| H | 4.839965414 | -1.269915578 | 6.554026907 |
| H | 7.297997763 | -0.053959415 | 3.239705775 |
| H | 6.879087765 | -0.134945509 | 5.692603886 |
| C | 2.030820129 | 0.699169493 | 3.720954661 |
| C | 1.450157408 | 0.806398934 | 5.002406073 |
| C | 3.287971257 | 1.275330687 | 3.475874100 |
| C | 2.135675976 | 1.467177051 | 6.016793138 |
| C | 3.952926419 | 1.953760046 | 4.490807499 |
| C | 3.383782815 | 2.042888763 | 5.763705678 |
| H | 0.461862436 | 0.393274684 | 5.170698765 |
| H | 3.733451971 | 1.191776013 | 2.492782034 |
| H | 1.689654758 | 1.546175248 | 7.003881071 |
| H | 4.922983753 | 2.399267182 | 4.294731586 |
| H | 3.911141934 | 2.564407613 | 6.557360179 |

==============================

8A-TS1’’

==============================

| | | | |
|---|---|---|---|
| C | 0.000694065 | 0.039765556 | -0.244787668 |
| C | -0.478794515 | 0.260260741 | 1.923084332 |
| C | 2.601417926 | -0.117708518 | 0.642687964 |
| C | 0.525935706 | -0.008424597 | 2.646542951 |
| Rh | 0.938730627 | -1.343756547 | 1.040774138 |
| P | 1.805332651 | -3.072952164 | 2.657428664 |
| P | 1.194505947 | -2.715009715 | -0.916567500 |
| C | 0.803853149 | 1.052193932 | -0.585448904 |
| C | 2.118086340 | 1.223328396 | 0.125424415 |
| C | 2.945810355 | 1.900216012 | -0.993936885 |
| C | 0.760895536 | 2.037785650 | -1.733247998 |
| O | 2.008495348 | 2.736607366 | -1.666382762 |
| H | 3.117961509 | -0.043164528 | 1.595921800 |
| H | 3.189428871 | -0.667961977 | -0.091900219 |
| H | -0.929804941 | -0.179507308 | -0.764740100 |
| H | 0.647628628 | 1.511420654 | -2.692002173 |
| H | -0.047942679 | 2.773477702 | -1.644238492 |
| H | 3.760847654 | 2.529993560 | -0.629260456 |
| H | 3.359130481 | 1.134673344 | -1.672103547 |
| H | 1.991916054 | 1.931639123 | 0.956027697 |
| C | -1.862540613 | 0.797346290 | 1.808302017 |
| H | -2.380588430 | 0.308604971 | 0.975520317 |
| C | 1.087598483 | 0.272585447 | 3.970397170 |
| O | 0.477868463 | 0.827162499 | 4.860141989 |
| O | 2.366964367 | -0.141690229 | 4.064747251 |
| C | 2.987382226 | 0.046839477 | 5.354699282 |
| H | 3.038630558 | 1.110083976 | 5.600236355 |
| H | 2.414330944 | -0.477886431 | 6.122257963 |
| H | 3.984596864 | -0.381569242 | 5.256899838 |
| C | 0.952786942 | -1.843208348 | -2.518573582 |
| C | 2.052835376 | -1.193425451 | -3.102478628 |
| C | -0.300912163 | -1.751636008 | -3.136886853 |
| C | 1.902956989 | -0.477081775 | -4.287918287 |
| C | -0.447544473 | -1.030691716 | -4.324011835 |
| C | 0.651195536 | -0.394772404 | -4.902778734 |
| H | 3.035232429 | -1.265819562 | -2.645350533 |
| H | -1.161977244 | -2.254475081 | -2.708210531 |
| H | 2.763199039 | 0.015405546 | -4.731687364 |
| H | -1.421875868 | -0.976232321 | -4.801029262 |
| H | 0.534613676 | 0.160220896 | -5.828843457 |
| C | 2.792875392 | -3.549213961 | -1.230876446 |
| C | 2.994096755 | -4.316891947 | -2.391747797 |
| C | 3.868680087 | -3.348390373 | -0.358282605 |
| C | 4.228054593 | -4.917145579 | -2.631918014 |
| C | 5.108809929 | -3.938585956 | -0.605167220 |
| C | 5.286515742 | -4.733779856 | -1.736896158 |
| H | 2.194137752 | -4.428657223 | -3.117310514 |
| H | 3.741438974 | -2.742452215 | 0.527581392 |
| H | 4.368762572 | -5.518293953 | -3.525465870 |
| H | 5.926221587 | -3.776437811 | 0.091767298 |
| H | 6.248459642 | -5.198816479 | -1.931979546 |
| C | -0.154962953 | -3.954636022 | -0.870625189 |
| C | -1.389145751 | -3.512418776 | -0.359084121 |
| C | -0.047356627 | -5.269214529 | -1.339141394 |
| C | -2.497442693 | -4.356923313 | -0.343867884 |
| C | -1.158730816 | -6.112231751 | -1.321194855 |
| C | -2.384634172 | -5.659282093 | -0.833225492 |
| H | -1.488992143 | -2.495811669 | 0.016983291 |
| H | 0.901763298 | -5.649701405 | -1.696657548 |
| H | -3.445474412 | -3.998852196 | 0.047503251 |
| H | -1.061532843 | -7.131264286 | -1.684069605 |
| H | -3.246082127 | -6.320563203 | -0.825538621 |
| C | 1.369774115 | -4.804355163 | 2.148581331 |
| C | 0.102611149 | -5.338437471 | 2.429826978 |
| C | 2.297643576 | -5.605536972 | 1.461811731 |
| C | -0.220257202 | -6.639919577 | 2.048960611 |
| C | 1.972640357 | -6.908211463 | 1.087272734 |
| C | 0.714299686 | -7.431879312 | 1.382534981 |
| H | -0.634511287 | -4.756642847 | 2.970388066 |
| H | 3.287851141 | -5.231056190 | 1.236601366 |
| H | -1.204676817 | -7.035024935 | 2.281625395 |
| H | 2.711546416 | -7.513905183 | 0.570159098 |
| H | 0.463252777 | -8.449013954 | 1.095703598 |
| C | 3.564928268 | -3.238740121 | 3.169574591 |
| C | 4.035203032 | -4.416886562 | 3.776952324 |
| C | 4.459967830 | -2.176853952 | 2.979542400 |
| C | 5.368310840 | -4.528243550 | 4.168866587 |
| C | 5.795930983 | -2.292005607 | 3.366411420 |
| C | 6.253764277 | -3.468961683 | 3.959865862 |
| H | 3.362160522 | -5.252083873 | 3.939471619 |
| H | 4.113866586 | -1.255509548 | 2.534669467 |
| H | 5.715117583 | -5.445489067 | 4.635723491 |
| H | 6.476385246 | -1.461125976 | 3.203136013 |
| H | 7.293641770 | -3.561071247 | 4.259504766 |
| C | 0.879851417 | -2.901174449 | 4.243272689 |
| C | -0.486480923 | -2.576237824 | 4.193632063 |
| C | 1.478174977 | -3.101927241 | 5.495440524 |
| C | -1.236459428 | -2.463165670 | 5.362505589 |
| C | 0.725977102 | -2.982885086 | 6.664978312 |
| C | -0.630724441 | -2.664148330 | 6.603000000 |
| H | -0.970221792 | -2.397993199 | 3.237778484 |
| H | 2.532222223 | -3.345516778 | 5.566959353 |
| H | -2.289858088 | -2.205764883 | 5.304058364 |
| H | 1.205429259 | -3.140333265 | 7.626984117 |
| H | -1.211091981 | -2.567875871 | 7.515540756 |
| C | -2.597236515 | 0.471946865 | 3.127442097 |
| H | -3.592575894 | 0.926504814 | 3.116174587 |
| H | -2.712912787 | -0.608195880 | 3.258992379 |
| H | -2.036983723 | 0.860034276 | 3.982678667 |
| C | -1.814996581 | 2.314248301 | 1.539291989 |
| H | -1.314743752 | 2.830316409 | 2.364467975 |
| H | -1.275866839 | 2.532921047 | 0.614513742 |
| H | -2.832928356 | 2.705968243 | 1.453504488 |

==============================

8B-TS1’

==============================

| | | | |
|---|---|---|---|
| C | -0.146876346 | -0.096087845 | 0.013215739 |
| C | 0.130056310 | -0.077484589 | 2.338155783 |
| C | 2.614476307 | 0.119034363 | 0.088284022 |
| C | 1.332141258 | 0.015445055 | 2.744186030 |
| Rh | 1.329195312 | -1.272820481 | 0.999891789 |
| P | 3.178591723 | -2.621475130 | 1.884345044 |
| P | 0.705881227 | -2.707503052 | -0.916473573 |
| C | 0.383655884 | 1.013437644 | -0.495706567 |
| C | 1.821479332 | 1.369432190 | -0.216564367 |
| C | 2.165767007 | 2.142182499 | -1.514060617 |
| C | -0.135244344 | 1.991651410 | -1.532479218 |
| O | 0.970163084 | 2.855783061 | -1.811790539 |
| H | 3.441400229 | 0.272979229 | 0.781131102 |
| H | 2.946687588 | -0.394730444 | -0.813345162 |
| H | -1.165736682 | -0.429637277 | -0.147816241 |
| H | -0.459671666 | 1.455144843 | -2.437313512 |
| H | -0.968229556 | 2.606056789 | -1.173454252 |
| H | 2.974575705 | 2.868000235 | -1.400276516 |
| H | 2.422578607 | 1.433532780 | -2.318801630 |
| H | 1.861634148 | 2.068820537 | 0.629196413 |
| C | 2.172885609 | 0.607056392 | 3.821590420 |
| H | 3.217723335 | 0.324597977 | 3.694957174 |
| C | -1.236250136 | 0.317907336 | 2.694758800 |
| O | -1.441982485 | 1.146973947 | 3.558462175 |
| O | -2.194789841 | -0.364138312 | 2.048612555 |
| C | -3.552004912 | -0.028556399 | 2.411633469 |
| H | -3.716041349 | -0.204029146 | 3.477700877 |
| H | -3.757057412 | 1.020879235 | 2.186316336 |
| H | -4.178344912 | -0.684724796 | 1.807928370 |
| C | 0.645402657 | -1.795328257 | -2.507968975 |
| C | 1.845428885 | -1.402784307 | -3.125564280 |
| C | -0.569079937 | -1.406003885 | -3.090900018 |
| C | 1.830699841 | -0.655838954 | -4.302924822 |
| C | -0.580850032 | -0.658056407 | -4.269891161 |
| C | 0.615948070 | -0.281666735 | -4.879474116 |
| H | 2.803081461 | -1.687693410 | -2.701943802 |
| H | -1.509920560 | -1.685059325 | -2.629612935 |
| H | 2.768280331 | -0.367457647 | -4.769181312 |
| H | -1.530674888 | -0.369475418 | -4.710646239 |
| H | 0.603365500 | 0.302467226 | -5.794576755 |
| C | 1.526803115 | -4.305897141 | -1.313830935 |
| C | 1.542612668 | -5.270728456 | -0.295335422 |
| C | 2.087383712 | -4.618737723 | -2.558912870 |
| C | 2.143791088 | -6.508385330 | -0.503595672 |
| C | 2.686774825 | -5.862600906 | -2.764696777 |
| C | 2.725978877 | -6.805231364 | -1.737930564 |
| H | 1.074465952 | -5.059920626 | 0.660082640 |
| H | 2.060107140 | -3.903800903 | -3.373177259 |
| H | 2.152282171 | -7.239795660 | 0.299085369 |
| H | 3.120697610 | -6.093279885 | -3.733565436 |
| H | 3.197084575 | -7.769975460 | -1.901727739 |
| C | -0.997206587 | -3.323225893 | -0.606889663 |
| C | -1.639761662 | -4.118664097 | -1.571785271 |
| C | -1.623320156 | -3.105442952 | 0.625853490 |
| C | -2.896048160 | -4.658693044 | -1.312723057 |
| C | -2.879816058 | -3.655537108 | 0.886437750 |
| C | -3.518194568 | -4.429181011 | -0.081569531 |
| H | -1.152578338 | -4.318849043 | -2.521832015 |
| H | -1.148447682 | -2.491841943 | 1.382700663 |
| H | -3.386808037 | -5.266624762 | -2.067089017 |
| H | -3.353968490 | -3.478147430 | 1.847526764 |
| H | -4.495566397 | -4.857837548 | 0.120041006 |
| C | 4.444047405 | -3.320056260 | 0.758981484 |
| C | 5.533229009 | -4.045665090 | 1.275293031 |
| C | 4.400013661 | -3.052470475 | -0.610967332 |
| C | 6.524144104 | -4.526035794 | 0.423883531 |
| C | 5.396687043 | -3.525384792 | -1.464407410 |
| C | 6.456585007 | -4.268934412 | -0.948840967 |
| H | 5.617372429 | -4.214441611 | 2.344730783 |
| H | 3.581991235 | -2.469840710 | -1.008877080 |
| H | 7.358050640 | -5.089297480 | 0.832370107 |
| H | 5.342382263 | -3.312776057 | -2.528264776 |
| H | 7.235818592 | -4.637641870 | -1.609570382 |
| C | 2.364561433 | -4.010475197 | 2.772374041 |
| C | 1.198448175 | -3.717780401 | 3.503022266 |
| C | 2.825710625 | -5.334818924 | 2.734367958 |
| C | 0.512848082 | -4.727297033 | 4.180653732 |
| C | 2.134925797 | -6.340939444 | 3.410559968 |
| C | 0.976820732 | -6.043340308 | 4.133192833 |
| H | 0.826109580 | -2.697903245 | 3.550975320 |
| H | 3.704656400 | -5.592576739 | 2.155163835 |
| H | -0.384868592 | -4.483948267 | 4.741797268 |
| H | 2.502664398 | -7.362397060 | 3.368877689 |
| H | 0.440846767 | -6.831088581 | 4.654058912 |
| C | 4.288083291 | -1.874713059 | 3.142037102 |
| C | 4.204139091 | -2.184315869 | 4.504611801 |
| C | 5.254361650 | -0.951012791 | 2.705854013 |
| C | 5.056163836 | -1.561606632 | 5.419878059 |
| C | 6.098517475 | -0.329318709 | 3.622336102 |
| C | 5.997042851 | -0.629511090 | 4.984157310 |
| H | 3.479615445 | -2.910204327 | 4.858339558 |
| H | 5.360070052 | -0.730647449 | 1.647129302 |
| H | 4.982648164 | -1.809961057 | 6.474728097 |
| H | 6.839772280 | 0.384487170 | 3.274949013 |
| H | 6.655903719 | -0.145196733 | 5.698760880 |
| C | 2.050820722 | 2.144335668 | 3.748054001 |
| H | 2.452659219 | 2.535534191 | 2.808424665 |
| H | 1.002964016 | 2.445304126 | 3.834643387 |
| H | 2.615219990 | 2.594216217 | 4.571313724 |
| C | 1.666499444 | 0.081635891 | 5.182567170 |
| H | 0.624487048 | 0.378759373 | 5.336324259 |
| H | 1.734642037 | -1.008268809 | 5.239953185 |
| H | 2.275847813 | 0.501320738 | 5.989024307 |

==============================

15A-TS1’

==============================

| | | | |
|---|---|---|---|
| C | 0.853089406 | 0.548777413 | -0.878371709 |
| C | 0.417681128 | -0.449417720 | 1.768628061 |
| C | 2.511678575 | 0.235205491 | 4.532945385 |
| C | 1.222634871 | 0.240002598 | 5.067657919 |
| H | 0.418168855 | 0.733911030 | 4.535087500 |
| C | 0.957439407 | -0.383585562 | 6.295533661 |
| C | -0.445274566 | -0.411464040 | 6.857452065 |
| C | 2.013813875 | -0.999022113 | 6.971866438 |
| H | 1.819409197 | -1.482530087 | 7.927508036 |
| C | 3.321398109 | -1.006519795 | 6.462485369 |
| C | 4.437353370 | -1.675024226 | 7.231031748 |
| C | 3.557745858 | -0.380639100 | 5.238826952 |
| H | 4.566032321 | -0.368812686 | 4.832173691 |
| C | 3.831093984 | 2.549434084 | 3.702248718 |
| C | 5.201713441 | 2.545947634 | 3.973572713 |

| | | | |
|---|---|---|---|
| H | 5.820567399 | 1.709769475 | 3.666917562 |
| C | 5.795845979 | 3.630659035 | 4.627779040 |
| C | 7.285152177 | 3.631948799 | 4.878653940 |
| C | 4.979153848 | 4.686249900 | 5.057223816 |
| H | 5.432373670 | 5.522128698 | 5.584837230 |
| C | 3.594111662 | 4.684210507 | 4.844756984 |
| C | 2.706544485 | 5.780325738 | 5.384491870 |
| C | 3.034761999 | 3.618334791 | 4.133422408 |
| H | 1.967613193 | 3.622305371 | 3.920502910 |
| C | 3.918231834 | -0.037111040 | 2.001182773 |
| C | 3.528178023 | -1.411154250 | 2.121046347 |
| H | 2.918599444 | -1.708548964 | 2.965612125 |
| C | 3.895494821 | -2.343895146 | 1.191301160 |
| H | 3.578959248 | -3.376793765 | 1.299035592 |
| C | 4.684635050 | -1.976484308 | 0.071806671 |
| C | 5.059604935 | -2.928356205 | -0.910223145 |
| H | 4.709630068 | -3.951512402 | -0.800920005 |
| C | 5.855339342 | -2.568058006 | -1.973835843 |
| H | 6.143335801 | -3.305705006 | -2.717064036 |
| C | 6.301783421 | -1.231612221 | -2.097252885 |
| H | 6.929548670 | -0.948672382 | -2.937247826 |
| C | 5.946869025 | -0.283631750 | -1.163125810 |
| H | 6.284816383 | 0.738239383 | -1.282390790 |
| C | 5.130906883 | -0.622663242 | -0.047923074 |
| C | 4.746731776 | 0.353649196 | 0.941911472 |
| C | 5.397350513 | 1.704377997 | 0.856932299 |
| C | 6.802031467 | 1.755347159 | 1.177692884 |
| C | 7.499066630 | 0.640814582 | 1.722667443 |
| H | 6.967187056 | -0.287695208 | 1.896747866 |
| C | 8.836103893 | 0.726096556 | 2.042113308 |
| H | 9.344783824 | -0.136179561 | 2.463419154 |
| C | 9.554110217 | 1.924777158 | 1.824658164 |
| H | 10.609875299 | 1.976080280 | 2.073781645 |
| C | 8.910985342 | 3.023185755 | 1.300962165 |
| H | 9.450092304 | 3.951579639 | 1.131859924 |
| C | 7.531124314 | 2.970450073 | 0.974840472 |
| C | 6.856789579 | 4.092892030 | 0.431921739 |
| H | 7.418619129 | 5.001022832 | 0.229942471 |
| C | 5.516606332 | 4.035481367 | 0.161491036 |
| H | 5.022364449 | 4.899854741 | -0.266076983 |
| C | 4.758684800 | 2.854688898 | 0.415278613 |
| C | 2.380344058 | 4.579848900 | 0.394828300 |
| C | 1.196871712 | 5.013967367 | -0.224079783 |
| H | 0.697770933 | 4.373355410 | -0.947298470 |
| C | 0.654001225 | 6.266613807 | 0.079241785 |
| C | -0.603092629 | 6.746784156 | -0.606324846 |
| C | 1.321344756 | 7.080245585 | 1.002477941 |
| H | 0.914866656 | 8.064082310 | 1.230558137 |
| C | 2.499394008 | 6.668552131 | 1.634463300 |
| C | 3.245053477 | 7.600731954 | 2.560886406 |
| C | 3.008903395 | 5.396455899 | 1.345574152 |
| H | 3.902988673 | 5.062466855 | 1.858534026 |
| C | 3.141592108 | 2.852107437 | -1.855109157 |
| C | 3.126919100 | 4.006587555 | -2.647939003 |
| H | 2.942140614 | 4.976613871 | -2.198406547 |
| C | 3.331074093 | 3.925734453 | -4.028911298 |
| C | 3.282082761 | 5.164237706 | -4.892264825 |
| C | 3.554412457 | 2.667715769 | -4.606836701 |
| H | 3.707367090 | 2.598179155 | -5.682052042 |
| C | 3.588036480 | 1.502357222 | -3.834932523 |
| C | 3.770568395 | 0.141049296 | -4.460140109 |
| C | 3.391221208 | 1.611043227 | -2.452048236 |
| H | 3.414696788 | 0.710219258 | -1.846508373 |
| P | 2.907283937 | 1.147078992 | 2.996557891 |
| P | 2.964058823 | 2.896301831 | -0.022388452 |
| Rh | 1.660762202 | 1.145375916 | 0.947803062 |
| C | -0.079554093 | 2.299691198 | 1.158152690 |
| C | -1.003554081 | 1.567281638 | 2.120058242 |
| C | -0.694008441 | 0.104136265 | 2.271149902 |
| H | 0.703589824 | -1.473577215 | 1.982946324 |
| C | -1.873963340 | -0.480466874 | 3.009576546 |
| H | -0.854148902 | 1.994181567 | 3.127725713 |
| C | -2.521217501 | 1.489249695 | 1.903341344 |
| H | 0.263783239 | 3.256054549 | 1.550304235 |
| H | -0.498647978 | 2.440425202 | 0.162034534 |
| H | -2.155822941 | -1.466257169 | 2.611862409 |
| H | -1.688852779 | -0.598045366 | 4.087275004 |
| H | -3.068876526 | 2.402253736 | 2.151649297 |
| H | -2.746687483 | 1.206734872 | 0.862007349 |
| O | -2.936872923 | 0.467884390 | 2.826677956 |
| C | 0.631066427 | -0.563564904 | -0.293647172 |
| C | 0.480733518 | 1.206137431 | -2.137524001 |
| O | 0.099652373 | 2.358520801 | -2.219716973 |
| O | 0.566283708 | 0.360701440 | -3.180189104 |
| C | 0.186341457 | 0.924815131 | -4.456203353 |
| H | 0.287843838 | 0.110606050 | -5.173182135 |
| H | -0.844155461 | 1.285752189 | -4.422154313 |
| H | 0.855266573 | 1.751080302 | -4.708917292 |
| H | 5.400959479 | -1.560026357 | 6.726664919 |
| H | 4.531160615 | -1.250734374 | 8.237031090 |
| H | 4.246134287 | -2.747773846 | 7.350415425 |
| H | -0.443300550 | -0.312160439 | 7.947409377 |
| H | -1.059888341 | 0.394919600 | 6.445620627 |
| H | -0.943986525 | -1.359845022 | 6.620453918 |
| H | 7.631306398 | 4.601205179 | 5.247542445 |
| H | 7.557360370 | 2.875435036 | 5.624382075 |
| H | 7.836606190 | 3.395426380 | 3.962927542 |
| H | 1.935696138 | 6.058499228 | 4.658679517 |
| H | 2.194571878 | 5.446024019 | 6.294999273 |
| H | 3.276979032 | 6.678107503 | 5.636877051 |
| H | 2.841178777 | -0.436149892 | -4.379783155 |
| H | 4.039491601 | 0.210529013 | -5.517868545 |
| H | 4.547161633 | -0.431112967 | -3.942720921 |
| H | 4.101403394 | 5.176161705 | -5.618680082 |
| H | 2.344885147 | 5.206833743 | -5.460354976 |
| H | 3.346775595 | 6.076269903 | -4.292423329 |
| H | -1.245475738 | 7.306373191 | 0.081138129 |
| H | -0.359823601 | 7.417176191 | -1.439388682 |
| H | -1.178643794 | 5.911686102 | -1.014236516 |
| H | 3.974228129 | 7.059438867 | 3.169290823 |
| H | 3.790018491 | 8.357920010 | 1.983873725 |
| H | 2.565212716 | 8.134699586 | 3.232154038 |
| C | 0.525249537 | -1.990567704 | -0.518896959 |
| C | -0.524167423 | -2.773624234 | -0.010728443 |
| C | 1.493606140 | -2.581011151 | -1.353698557 |
| C | -0.604999391 | -4.124637461 | -0.337149381 |
| C | 1.410142281 | -3.933795557 | -1.668079991 |
| C | 0.363613169 | -4.707863492 | -1.159149182 |
| H | -1.279769525 | -2.309727636 | 0.611951267 |
| H | 2.301528373 | -1.971581218 | -1.741879780 |
| H | -1.426052343 | -4.723513100 | 0.046121217 |
| H | 2.161217078 | -4.382743531 | -2.312011494 |
| H | 0.298934765 | -5.763689296 | -1.406494324 |

================================

15B-TS1’

================================

| | | | |
|---|---|---|---|
| C | 0.592654468 | -0.350342914 | -0.022956496 |
| C | 0.227933481 | -0.149407375 | 1.928437671 |
| C | 2.186556048 | 0.245391072 | 4.487011277 |
| C | 1.017430932 | 0.669059194 | 5.136663271 |
| H | 0.486477301 | 1.545315211 | 4.778294732 |
| C | 0.528837870 | -0.000587353 | 6.259224491 |
| C | -0.719817414 | 0.477154258 | 6.964566244 |
| C | 1.234950393 | -1.117852661 | 6.726473408 |
| H | 0.862240417 | -1.654990452 | 7.596403689 |
| C | 2.413087087 | -1.551884103 | 6.111026225 |
| C | 3.149722131 | -2.764623350 | 6.630964472 |
| C | 2.888864653 | -0.854427303 | 4.989879563 |

| | | | |
|---|---|---|---|
| H | 3.813774238 | -1.179385902 | 4.523576422 |
| C | 3.363104224 | 2.739724942 | 4.038090504 |
| C | 4.388863434 | 2.573415991 | 4.975435279 |
| H | 4.857675418 | 1.601162301 | 5.102228233 |
| C | 4.819336307 | 3.651097030 | 5.751192533 |
| C | 5.963436708 | 3.490151475 | 6.721787330 |
| C | 4.190870383 | 4.891339317 | 5.576019942 |
| H | 4.521484143 | 5.737321570 | 6.175308316 |
| C | 3.146767776 | 5.074620233 | 4.663639836 |
| C | 2.516289685 | 6.432638333 | 4.469039829 |
| C | 2.729545964 | 3.976704532 | 3.902997842 |
| H | 1.907168221 | 4.082364206 | 3.201291316 |
| C | 4.085869515 | 0.471641458 | 2.173177019 |
| C | 3.886368043 | -0.917550362 | 1.896642341 |
| H | 3.047985213 | -1.442146412 | 2.337818819 |
| C | 4.718181339 | -1.591801934 | 1.040087635 |
| H | 4.544668820 | -2.645593042 | 0.840586130 |
| C | 5.780268525 | -0.923086063 | 0.383056918 |
| C | 6.639943873 | -1.610096966 | -0.515443952 |
| H | 6.499599346 | -2.679396522 | -0.652223161 |
| C | 7.620339278 | -0.937411537 | -1.208475177 |
| H | 8.269043206 | -1.470729069 | -1.897105851 |
| C | 7.777134206 | 0.458175428 | -1.026162758 |
| H | 8.539003494 | 0.990387052 | -1.588271514 |
| C | 6.974969774 | 1.148869575 | -0.147309333 |
| H | 7.105295162 | 2.217840941 | -0.029059888 |
| C | 5.957432928 | 0.484212130 | 0.594205888 |
| C | 5.091219324 | 1.179365408 | 1.503314313 |
| C | 5.291375142 | 2.656925067 | 1.667958929 |
| C | 6.420233173 | 3.113527234 | 2.425188854 |
| C | 7.281321215 | 2.217234487 | 3.116360188 |
| H | 7.087885176 | 1.151393981 | 3.064448700 |
| C | 8.352673784 | 2.684028297 | 3.845113388 |
| H | 8.998446218 | 1.980522124 | 4.362894028 |
| C | 8.621248283 | 4.072504315 | 3.927738914 |
| H | 9.472211604 | 4.425977401 | 4.502344960 |
| C | 7.805096633 | 4.967086179 | 3.273077380 |
| H | 8.000964877 | 6.034880958 | 3.326058955 |
| C | 6.695617740 | 4.516332085 | 2.510191658 |
| C | 5.859090410 | 5.417654794 | 1.808792104 |
| H | 6.085791637 | 6.480515519 | 1.838992424 |
| C | 4.778837282 | 4.965987722 | 1.093272703 |
| H | 4.167253462 | 5.681265464 | 0.558428344 |
| C | 4.460265212 | 3.581105575 | 1.037238075 |
| C | 2.085205045 | 4.507444190 | -0.438847196 |
| C | 1.607869102 | 4.661776660 | -1.744034837 |
| H | 1.890196218 | 3.956365791 | -2.516145188 |
| C | 0.753786166 | 5.723374650 | -2.078752653 |
| C | 0.221989874 | 5.841558590 | -3.487653446 |
| C | 0.407578230 | 6.642658601 | -1.086568423 |
| H | -0.242937250 | 7.477413991 | -1.339229627 |
| C | 0.879739605 | 6.522280151 | 0.227833757 |
| C | 0.478000299 | 7.523797302 | 1.285687204 |
| C | 1.706138784 | 5.441913287 | 0.542728089 |
| H | 2.075517472 | 5.344985844 | 1.558299029 |
| C | 3.879076597 | 2.436980958 | -1.470155999 |
| C | 4.627401038 | 3.369580845 | -2.206755240 |
| H | 4.653418629 | 4.410335311 | -1.895051625 |
| C | 5.327461383 | 2.979230116 | -3.348560905 |
| C | 6.113911626 | 3.977738059 | -4.165444231 |
| C | 5.270009503 | 1.631967679 | -3.739016595 |
| H | 5.801674028 | 1.321494443 | -4.636721910 |
| C | 4.553943279 | 0.679721221 | -3.010840221 |
| C | 4.510883384 | -0.768753006 | -3.435154033 |
| C | 3.868214915 | 1.096315139 | -1.859908834 |
| H | 3.310760544 | 0.364210943 | -1.283995630 |
| P | 2.738497759 | 1.309566011 | 3.101779831 |
| P | 3.006425324 | 2.998617199 | 0.042212824 |
| Rh | 1.584143870 | 1.293582228 | 1.001835924 |
| C | -0.084460700 | 2.566158763 | 1.177713079 |
| C | -0.930563465 | 2.024783881 | 2.309785289 |
| C | -0.784632352 | 0.539111154 | 2.468099689 |
| H | 0.442012889 | -1.177476537 | 2.195213531 |
| C | -1.966828094 | 0.083548793 | 3.292714486 |
| H | -0.571549069 | 2.468698539 | 3.253816156 |
| C | -2.463140058 | 2.149834420 | 2.325716176 |
| H | 0.304333171 | 3.564238435 | 1.367444323 |
| H | -0.589277886 | 2.554058338 | 0.210940970 |
| H | -2.462486822 | -0.779943290 | 2.823772508 |
| H | -1.697252497 | -0.191928678 | 4.319505856 |
| H | -2.842633675 | 3.133018935 | 2.615819311 |
| H | -2.883579197 | 1.877659477 | 1.342753673 |
| O | -2.848300888 | 1.217163392 | 3.343339528 |
| C | 0.652759062 | 0.637577440 | -0.846585019 |
| C | 0.745331512 | -1.818261466 | -0.134716422 |
| O | 1.401821972 | -2.520096292 | 0.610679695 |
| O | 0.091072902 | -2.258220903 | -1.219595708 |
| C | 0.211824300 | -3.674098684 | -1.481438143 |
| H | -0.389742243 | -3.854551281 | -2.371520010 |
| H | 1.256991180 | -3.940486707 | -1.658717678 |
| H | -0.165472963 | -4.250998392 | -0.633265102 |
| H | 4.178183948 | -2.801639680 | 6.260147229 |
| H | 3.182722804 | -2.773105772 | 7.725155281 |
| H | 2.651134352 | -3.688973394 | 6.314440611 |
| H | -0.467938555 | 0.982198016 | 7.904928923 |
| H | -1.288968784 | 1.180555920 | 6.349436552 |
| H | -1.378636455 | -0.360692666 | 7.216972744 |
| H | 5.891117065 | 4.199619318 | 7.550999637 |
| H | 5.996189016 | 2.478869434 | 7.138335603 |
| H | 6.916000838 | 3.667072529 | 6.210807327 |
| H | 1.472458291 | 6.349688934 | 4.151422623 |
| H | 2.545644196 | 7.029337245 | 5.385892549 |
| H | 3.053846888 | 6.996737702 | 3.694454621 |
| H | 3.538642669 | -1.218597669 | -3.206991010 |
| H | 4.693069019 | -0.879129845 | -4.508231345 |
| H | 5.274539238 | -1.346373411 | -2.902718769 |
| H | 7.135000952 | 3.623654620 | -4.345805471 |
| H | 5.650148567 | 4.135360971 | -5.146568001 |
| H | 6.176076544 | 4.948566921 | -3.665949645 |
| H | -0.341296008 | 6.767857184 | -3.631226120 |
| H | 1.036354408 | 5.822752432 | -4.221050240 |
| H | -0.439116362 | 4.998837529 | -3.721140558 |
| H | 1.249069191 | 7.628196939 | 2.053911241 |
| H | 0.294413950 | 8.511530277 | 0.852355700 |
| H | -0.446472649 | 7.213014557 | 1.788050880 |
| C | 0.100239606 | 1.164533014 | -2.070426540 |
| C | 0.853344039 | 1.627144549 | -3.163044425 |
| C | -1.309220208 | 1.200722233 | -2.151238549 |
| C | 0.212170838 | 2.109264713 | -4.301034196 |
| C | -1.939884610 | 1.703218522 | -3.285189320 |
| C | -1.182561696 | 2.158624455 | -4.366686227 |
| H | 1.932836700 | 1.585324182 | -3.129198819 |

| | | | |
|---|---|---|---|
| H | -1.894170503 | 0.827031995 | -1.317043500 |
| H | 0.808669032 | 2.447292979 | -5.143788971 |
| H | -3.025183648 | 1.727970084 | -3.327701388 |
| H | -1.675543600 | 2.540446014 | -5.255936904 |

==============================

15A-TS1’

==============================

| | | | |
|---|---|---|---|
| C | 0.755146255 | 0.668091600 | -0.851003932 |
| C | 0.306568932 | -0.468775447 | 1.678987895 |
| C | 2.492243888 | -0.006408454 | 4.469056466 |
| C | 1.200108522 | 0.019598318 | 4.998083483 |
| H | 0.414281268 | 0.556735296 | 4.479474560 |
| C | 0.907982989 | -0.630677424 | 6.204948721 |
| C | -0.497119825 | -0.623707077 | 6.761735916 |
| C | 1.940584744 | -1.301334800 | 6.865867786 |
| H | 1.726747138 | -1.804954213 | 7.806858862 |
| C | 3.248748692 | -1.337100012 | 6.360038035 |
| C | 4.338699229 | -2.062351754 | 7.114497955 |
| C | 3.513485540 | -0.679508650 | 5.157910968 |
| H | 4.524486962 | -0.686220482 | 4.757944795 |
| C | 3.717588175 | 2.410255473 | 3.810442404 |
| C | 5.071396841 | 2.453334127 | 4.151881353 |
| H | 5.741192811 | 1.658300936 | 3.843022760 |
| C | 5.582276577 | 3.534690261 | 4.876753766 |
| C | 7.057412023 | 3.600311899 | 5.193674246 |
| C | 4.694781027 | 4.529877873 | 5.308626761 |
| H | 5.082285615 | 5.362942103 | 5.890646405 |
| C | 3.323642312 | 4.477986685 | 5.027095270 |
| C | 2.363293909 | 5.511137745 | 5.567156715 |
| C | 2.850363519 | 3.421589582 | 4.243235737 |
| H | 1.796252332 | 3.386643166 | 3.975031875 |
| C | 4.012032309 | -0.058034074 | 1.975069375 |
| C | 3.663084826 | -1.447660339 | 1.973619393 |
| H | 2.995319030 | -1.820502839 | 2.741490852 |
| C | 4.150601780 | -2.305656377 | 1.026740788 |
| H | 3.882651483 | -3.357999860 | 1.053784541 |
| C | 4.997596821 | -1.836154607 | -0.009131048 |
| C | 5.473761744 | -2.705486337 | -1.023503498 |
| H | 5.177059681 | -3.750618909 | -0.994571171 |
| C | 6.294684881 | -2.236059214 | -2.023553069 |
| H | 6.655743074 | -2.908830514 | -2.795762727 |
| C | 6.664787458 | -0.870883774 | -2.048414838 |
| H | 7.302189272 | -0.500938335 | -2.846169393 |
| C | 6.219454533 | -0.003747750 | -1.075835857 |
| H | 6.499835204 | 1.041005719 | -1.121467087 |
| C | 5.376791776 | -0.454766594 | -0.021613554 |
| C | 4.891063323 | 0.434666251 | 1.003973679 |
| C | 5.455902884 | 1.824678347 | 1.025159431 |
| C | 6.839453295 | 1.949691506 | 1.412995244 |
| C | 7.585465246 | 0.856596400 | 1.937168106 |
| H | 7.109752607 | -0.112217733 | 2.040225006 |
| C | 8.897344544 | 1.012602239 | 2.328303778 |
| H | 9.441290952 | 0.164243594 | 2.733503935 |
| C | 9.544799133 | 2.264806773 | 2.205463165 |
| H | 10.581446328 | 2.371759388 | 2.509994184 |
| C | 8.853706203 | 3.342383000 | 1.698349809 |
| H | 9.335741569 | 4.311184334 | 1.597341912 |
| C | 7.496484757 | 3.216114927 | 1.301197705 |
| C | 6.772876599 | 4.314801200 | 0.774784806 |
| H | 7.280906617 | 5.264742049 | 0.634886216 |
| C | 5.450671444 | 4.187077908 | 0.444509782 |
| H | 4.923089779 | 5.041405828 | 0.038475028 |
| C | 4.757355893 | 2.952685829 | 0.613644299 |
| C | 2.325687844 | 4.582039061 | 0.472573150 |
| C | 1.229307013 | 5.048218514 | -0.268599080 |
| H | 0.812463564 | 4.436697300 | -1.062109820 |
| C | 0.662597497 | 6.298120057 | 0.007644322 |
| C | -0.492301596 | 6.815796708 | -0.818224434 |
| C | 1.211676847 | 7.073039871 | 1.034627146 |
| H | 0.787507013 | 8.053951154 | 1.243078996 |
| C | 2.298087903 | 6.625790097 | 1.795096306 |
| C | 2.920314615 | 7.516401424 | 2.845273571 |
| C | 2.832086792 | 5.361212422 | 1.523948024 |
| H | 3.658914004 | 5.004612083 | 2.127127411 |
| C | 3.217452336 | 2.821573540 | -1.718533860 |
| C | 3.298759933 | 3.967300401 | -2.520155575 |
| H | 3.162641653 | 4.951600242 | -2.083946540 |
| C | 3.531761303 | 3.858644078 | -3.893775535 |
| C | 3.577778391 | 5.086758405 | -4.772111726 |
| C | 3.691089955 | 2.581677242 | -4.451226829 |
| H | 3.867015540 | 2.490754732 | -5.521560722 |
| C | 3.630337502 | 1.424230428 | -3.670087611 |
| C | 3.752424775 | 0.047805930 | -4.276927413 |
| C | 3.404721676 | 1.560160380 | -2.294617667 |
| H | 3.359451722 | 0.666234824 | -1.681311836 |
| P | 2.904898614 | 1.000667914 | 2.998560794 |
| P | 2.976413943 | 2.907704070 | 0.101776956 |
| Rh | 1.643423465 | 1.118084559 | 0.973331044 |
| C | -0.016937065 | 2.363681926 | 1.292000594 |
| C | -0.985993063 | 1.611659705 | 2.186462363 |
| C | -0.770080816 | 0.125054302 | 2.213316400 |
| H | 0.519361725 | -1.514814308 | 1.876054616 |
| C | -1.988130219 | -0.443621864 | 2.902480661 |
| H | -0.804559439 | 1.933755186 | 3.227462433 |
| C | -2.507082588 | 1.654587442 | 1.986030203 |
| H | 0.393370675 | 3.254896045 | 1.765348912 |
| H | -0.419333460 | 2.621630599 | 0.312618014 |
| H | -2.341073752 | -1.352940803 | 2.394576990 |
| H | -1.811830849 | -0.691969503 | 3.959006333 |
| H | -2.987510555 | 2.580219492 | 2.313631266 |
| H | -2.763551653 | 1.470298329 | 0.930000399 |
| O | -2.976586430 | 0.596128041 | 2.836633457 |
| C | 0.644325542 | -0.490730976 | -0.307787139 |
| C | 0.592605392 | -1.957626263 | -0.624820211 |
| C | 0.402000499 | 1.392288056 | -2.071241797 |
| H | 0.964295794 | -2.523477416 | 0.235697639 |
| O | 0.150238129 | 2.582389218 | -2.121654522 |
| O | 0.344188315 | 0.575844850 | -3.142794641 |
| C | 0.025879983 | 1.227421725 | -4.393479047 |
| H | -0.010028519 | 0.428863333 | -5.134008136 |
| H | -0.937453798 | 1.738193514 | -4.325695996 |
| H | 0.806905251 | 1.951381235 | -4.639732113 |
| H | 5.303679037 | -1.984245447 | 6.605822185 |
| H | 4.456527151 | -1.652099518 | 8.123894398 |
| H | 4.101124333 | -3.126638267 | 7.225156117 |
| H | -0.495660099 | -0.533464521 | 7.852583258 |
| H | -1.086689480 | 0.203972256 | 6.354951412 |
| H | -1.022388390 | -1.555286436 | 6.515539132 |
| H | 7.335402504 | 4.569544868 | 5.616557227 |
| H | 7.337236896 | 2.827571468 | 5.919562641 |
| H | 7.659755819 | 3.433597968 | 4.294469808 |
| H | 1.605396894 | 5.775023604 | 4.822696581 |

| | | | |
|---|---|---|---|
| H | 1.837344857 | 5.123645666 | 6.448134944 |
| H | 2.878445811 | 6.427518377 | 5.867561023 |
| H | 2.776208143 | -0.452821301 | -4.275159256 |
| H | 4.110934405 | 0.088397909 | -5.309392356 |
| H | 4.437765559 | -0.580138847 | -3.697988675 |
| H | 4.398376479 | 5.029531567 | -5.495022525 |
| H | 2.647975909 | 5.191258230 | -5.344772412 |
| H | 3.706648291 | 5.999214979 | -4.182996173 |
| H | -1.218293491 | 7.352904720 | -0.198982093 |
| H | -0.139486583 | 7.517721905 | -1.584900257 |
| H | -1.011857064 | 6.001824129 | -1.331766866 |
| H | 3.630389866 | 6.965527332 | 3.467070655 |
| H | 3.462183203 | 8.344753214 | 2.373159093 |
| H | 2.162836541 | 7.959631776 | 3.499861317 |
| C | 1.513765656 | -2.251919017 | -1.823685624 |
| H | 2.550719136 | -1.985892202 | -1.597551283 |
| H | 1.476164619 | -3.320854402 | -2.060784252 |
| H | 1.193949268 | -1.687013786 | -2.701232042 |
| C | -0.867785650 | -2.367589668 | -0.894061838 |
| H | -1.263611836 | -1.814773386 | -1.751583783 |
| H | -0.924028895 | -3.437558229 | -1.116938488 |
| H | -1.500838941 | -2.158494954 | -0.027595172 |

==============================

15B-TS1’

==============================

| | | | |
|---|---|---|---|
| C | 0.259467629 | -0.127785250 | -0.062790638 |
| C | 0.209150838 | -0.215440134 | 1.922117612 |
| C | 2.642792469 | -0.098415374 | 4.292328488 |
| C | 1.590484360 | 0.229723646 | 5.158784949 |
| H | 0.997401881 | 1.120320274 | 4.980830635 |
| C | 1.302189045 | -0.553285166 | 6.277569595 |
| C | 0.181447619 | -0.169065680 | 7.216062694 |
| C | 2.093786756 | -1.683620001 | 6.516746057 |
| H | 1.877829352 | -2.307602687 | 7.381956723 |
| C | 3.162140221 | -2.025867735 | 5.681679662 |
| C | 3.992785235 | -3.256263421 | 5.963716742 |
| C | 3.435228811 | -1.218043498 | 4.568817611 |
| H | 4.275479786 | -1.470959653 | 3.929804551 |
| C | 3.598840481 | 2.497257949 | 3.899835117 |
| C | 4.786956988 | 2.422171857 | 4.630082114 |
| H | 5.426485563 | 1.547864266 | 4.544233120 |
| C | 5.171835081 | 3.475618692 | 5.466382694 |
| C | 6.491039042 | 3.425456629 | 6.198361336 |
| C | 4.322771491 | 4.582618329 | 5.581017216 |
| H | 4.608770397 | 5.399166280 | 6.241317842 |
| C | 3.112132995 | 4.667302029 | 4.882283986 |
| C | 2.215021177 | 5.871735201 | 5.042278564 |
| C | 2.762032562 | 3.611681594 | 4.036586370 |
| H | 1.824637601 | 3.645926653 | 3.489697549 |
| C | 4.175563034 | 0.344660624 | 1.777758385 |
| C | 3.881629963 | -0.998484890 | 1.378090412 |
| H | 3.069119271 | -1.536723411 | 1.851949835 |
| C | 4.594188282 | -1.608596453 | 0.377987426 |
| H | 4.353950634 | -2.628328834 | 0.090636256 |
| C | 5.623712823 | -0.916793849 | -0.307295734 |
| C | 6.376584058 | -1.537253966 | -1.338173564 |
| H | 6.184711214 | -2.582977539 | -1.564409268 |
| C | 7.323765784 | -0.829755141 | -2.042805079 |
| H | 7.893396828 | -1.315676196 | -2.829828328 |
| C | 7.549600935 | 0.537082900 | -1.751096889 |
| H | 8.281354938 | 1.096738391 | -2.326082052 |
| C | 6.850607444 | 1.160792078 | -0.743115569 |
| H | 7.030135740 | 2.208382113 | -0.534778257 |
| C | 5.881124288 | 0.454055493 | 0.023751208 |
| C | 5.139669588 | 1.078453507 | 1.082324410 |
| C | 5.395312416 | 2.525276917 | 1.367946944 |
| C | 6.622474467 | 2.897248763 | 2.009996061 |
| C | 7.562924292 | 1.932976357 | 2.468845471 |
| H | 7.351017599 | 0.878594829 | 2.328791159 |
| C | 8.732664185 | 2.320668873 | 3.083568972 |
| H | 9.436341059 | 1.566985355 | 3.424989913 |
| C | 9.030114525 | 3.692649972 | 3.273678090 |
| H | 9.960178420 | 3.983541827 | 3.752907163 |
| C | 8.137353667 | 4.650928918 | 2.849477661 |
| H | 8.351079925 | 5.707145805 | 2.991424697 |
| C | 6.920101866 | 4.282502057 | 2.216627432 |
| C | 5.989550958 | 5.250311830 | 1.768967521 |
| H | 6.219540308 | 6.303629343 | 1.903409805 |
| C | 4.813936685 | 4.878272514 | 1.166264996 |
| H | 4.134397167 | 5.648147847 | 0.824843431 |
| C | 4.489677271 | 3.507958001 | 0.970020155 |
| C | 2.032408116 | 4.606442235 | -0.206858427 |
| C | 1.587143681 | 4.960988704 | -1.484771040 |
| H | 1.852618954 | 4.354366149 | -2.344389074 |
| C | 0.807344510 | 6.108469091 | -1.681210370 |
| C | 0.302595955 | 6.450236573 | -3.062366729 |
| C | 0.496582601 | 6.905877252 | -0.576704380 |
| H | -0.096208616 | 7.806256395 | -0.722645241 |
| C | 0.935807496 | 6.582157401 | 0.713388492 |
| C | 0.580486969 | 7.452662473 | 1.895708091 |
| C | 1.695378728 | 5.422432142 | 0.886852176 |
| H | 2.054361607 | 5.172606998 | 1.881096245 |
| C | 3.594411379 | 2.505112510 | -1.527636575 |
| C | 4.380713590 | 3.401593408 | -2.267318071 |
| H | 4.551456664 | 4.408136010 | -1.893925900 |
| C | 4.949008065 | 3.016864857 | -3.484062093 |
| C | 5.781421474 | 3.980379899 | -4.296896457 |
| C | 4.726543875 | 1.711217498 | -3.942532554 |
| H | 5.167888997 | 1.402206706 | -4.888519208 |
| C | 3.971233041 | 0.789660709 | -3.212943995 |
| C | 3.791352330 | -0.627144575 | -3.702535352 |
| C | 3.413283007 | 1.200561158 | -1.997377479 |
| H | 2.843465288 | 0.484808982 | -1.414884618 |
| P | 2.956120422 | 1.092424371 | 2.936360722 |
| P | 2.916430538 | 3.032057849 | 0.096818884 |
| Rh | 1.517672487 | 1.325679917 | 1.043654332 |
| C | -0.078357792 | 2.572217041 | 1.616816369 |
| C | -0.677979412 | 1.928614440 | 2.845333387 |
| C | -0.617495183 | 0.429434207 | 2.755361183 |
| H | 0.387356295 | -1.280531618 | 2.004918016 |
| C | -1.624984085 | -0.097501066 | 3.752833350 |
| H | -0.075195977 | 2.213024220 | 3.723141761 |
| C | -2.148546932 | 2.118592656 | 3.256984572 |
| H | 0.316258443 | 3.573577527 | 1.776287793 |
| H | -0.776062549 | 2.585257580 | 0.781482027 |
| H | -2.303617682 | -0.820207821 | 3.273813571 |
| H | -1.164554413 | -0.580452378 | 4.622451750 |
| H | -2.367160813 | 3.061709121 | 3.764332851 |
| H | -2.816782328 | 2.011869502 | 2.386342024 |
| O | -2.344270914 | 1.060265836 | 4.205257204 |
| C | 0.413951133 | 0.964343522 | -0.730635364 |
| C | 0.133217994 | 1.632177971 | -2.035504309 |

| | | | |
|---|---|---|---|
| C | 0.277834051 | -1.575318365 | -0.366926068 |
| H | 1.006514582 | 2.236183903 | -2.293948641 |
| O | 1.089101964 | -2.364219300 | 0.078216359 |
| O | -0.689839318 | -1.890392304 | -1.240711955 |
| C | -0.692664526 | -3.265512806 | -1.688516134 |
| H | -1.545745960 | -3.348137380 | -2.360751400 |
| H | 0.238326137 | -3.495985262 | -2.212788410 |
| H | -0.800890261 | -3.941018177 | -0.836657443 |
| H | 4.955096889 | -3.221050344 | 5.444669785 |
| H | 4.188584687 | -3.368683321 | 7.034927258 |
| H | 3.471779490 | -4.163114628 | 5.632694911 |
| H | 0.568468265 | 0.396823668 | 8.072239524 |
| H | -0.566130283 | 0.455514221 | 6.717049540 |
| H | -0.325116887 | -1.052809392 | 7.616137899 |
| H | 6.478899473 | 4.052751316 | 7.094297683 |
| H | 6.742411677 | 2.403699594 | 6.499470123 |
| H | 7.298175539 | 3.782211344 | 5.548103442 |
| H | 1.271132545 | 5.746379522 | 4.503660941 |
| H | 1.978270714 | 6.054199252 | 6.096251529 |
| H | 2.700983056 | 6.778473622 | 4.661829543 |
| H | 2.999189302 | -1.145264208 | -3.153332373 |
| H | 3.543111840 | -0.654552389 | -4.768469262 |
| H | 4.715652269 | -1.197169717 | -3.561030858 |
| H | 6.719368991 | 3.515667363 | -4.619483233 |
| H | 5.248986256 | 4.293406173 | -5.202893399 |
| H | 6.028202684 | 4.880772684 | -3.727436132 |
| H | 0.004878687 | 7.499597194 | -3.136778556 |
| H | 1.063230883 | 6.256283131 | -3.825277238 |
| H | -0.572648322 | 5.838892237 | -3.313301304 |
| H | 1.392552679 | 7.484319807 | 2.627816824 |
| H | 0.359452041 | 8.478145904 | 1.586885277 |
| H | -0.307965054 | 7.067890202 | 2.411370310 |
| C | -1.069452485 | 2.591159384 | -1.905863900 |
| H | -1.285450299 | 3.037018000 | -2.882908520 |
| H | -1.964079294 | 2.055860987 | -1.571244549 |
| H | -0.858574117 | 3.404744366 | -1.206908994 |
| C | -0.084725535 | 0.601031746 | -3.157360511 |
| H | -0.241128618 | 1.116826318 | -4.110810906 |
| H | 0.786652455 | -0.052389154 | -3.263656556 |
| H | -0.955336736 | -0.025499110 | -2.949033390 |

**Table S5.** Vibrational Frequencies of Optimized Structures

===============================

1

===============================

| | | | | | |
|---|---|---|---|---|---|
| 44.49 | 98.32 | 103.35 | 149.28 | 218.30 | 285.89 |
| 333.21 | 379.96 | 469.81 | 573.78 | 756.63 | 823.64 |
| 976.59 | 1048.83 | 1055.91 | 1105.29 | 1177.40 | 1215.15 |
| 1301.13 | 1421.87 | 1479.89 | 1481.68 | 1484.39 | 1494.21 |
| 1507.13 | 1796.81 | 2363.79 | 3036.81 | 3058.37 | 3103.98 |
| 3107.82 | 3134.87 | 3171.55 | | | |

===============================

2

===============================

| | | | | | |
|---|---|---|---|---|---|
| 45.79 | 80.17 | 143.65 | 173.51 | 304.49 | 347.74 |
| 393.76 | 436.89 | 529.38 | 604.89 | 665.50 | 680.57 |
| 925.84 | 942.14 | 959.64 | 992.85 | 1015.44 | 1033.77 |
| 1039.41 | 1118.74 | 1180.36 | 1258.23 | 1295.82 | 1317.89 |
| 1386.29 | 1423.66 | 1467.00 | 1498.47 | 1515.07 | 1724.62 |
| 2248.28 | 2961.97 | 2982.81 | 3006.43 | 3076.75 | 3147.76 |
| 3162.20 | 3238.14 | 3495.98 | | | |

===============================

$PPh_3$

==============================
26.40 27.07 42.52 53.78 54.10 67.42
188.35 205.94 207.27 249.40 261.49 262.67
407.59 407.80 412.25 417.00 431.20 432.11
507.83 508.77 520.63 631.35 631.44 631.71
693.54 706.04 706.28 712.26 712.34 713.61
762.85 763.13 763.34 865.85 866.18 868.54
934.08 934.75 935.86 982.32 982.55 983.60
1004.42 1004.63 1005.07 1013.36 1013.41 1013.65
1051.89 1052.06 1052.41 1109.73 1109.94 1110.50
1111.59 1111.67 1118.47 1196.38 1196.80 1196.83
1221.24 1221.30 1222.61 1322.69 1327.05 1327.45
1362.24 1362.51 1362.65 1474.66 1475.71 1475.97
1524.27 1525.20 1525.28 1627.35 1628.14 1628.29
1645.05 1645.09 1646.89 3168.48 3168.54 3168.89
3176.20 3176.25 3176.29 3187.22 3187.31 3187.50
3198.86 3199.20 3199.49 3205.33 3205.68 3206.64
==============================
3
==============================
20.68 64.05 95.47 114.53 128.40 150.58
163.03 198.02 211.49 291.91 306.83 314.09
353.70 363.43 400.61 441.16 515.07 552.76
568.75 646.41 691.47 729.30 785.24 792.77
821.31 882.77 904.77 940.98 943.18 995.74
1015.36 1029.09 1046.28 1077.81 1095.54 1103.78
1117.31 1153.34 1179.59 1180.67 1204.54 1211.37
1219.87 1245.59 1247.78 1275.87 1296.85 1332.89
1355.61 1369.45 1384.64 1416.03 1422.37 1477.23
1478.87 1479.76 1493.91 1508.13 1509.06 1511.99
1530.06 1644.08 1743.33 1788.45 2966.69 2977.34
2981.17 3001.87 3022.35 3059.95 3064.22 3064.41
3080.39 3111.70 3135.74 3168.97 3210.45 3222.78
==============================
4
==============================
39.86 59.98 86.02 92.29 137.48 157.02
174.67 188.01 206.82 291.46 308.29 313.81
339.40 361.47 387.82 445.57 547.96 557.09
590.47 649.19 671.59 719.25 773.06 797.52
821.99 873.27 923.91 940.86 967.77 1002.65
1014.10 1040.61 1065.11 1075.07 1098.28 1103.07
1114.57 1116.03 1180.44 1183.22 1207.35 1213.67
1222.51 1242.46 1262.44 1272.55 1296.20 1336.88
1353.91 1372.16 1382.51 1418.30 1424.99 1479.71
1483.29 1492.60 1493.89 1507.32 1509.32 1513.79
1531.00 1629.31 1740.52 1774.63 2972.57 2980.38
2987.83 3003.12 3029.07 3055.51 3057.30 3074.18
3112.89 3130.24 3133.13 3164.06 3165.43 3213.94
==============================
$Rh(PPh_3)_2$
==============================
6.10 12.94 20.00 22.39 27.58 35.01
40.81 42.41 46.40 48.04 53.18 55.70
57.30 63.35 69.24 85.97 89.62 118.53
127.36 166.05 179.21 192.44 197.32 199.68
210.20 219.33 229.03 243.22 246.32 259.12
262.27 272.85 276.86 405.10 406.53 407.48
408.29 409.73 413.71 420.13 427.93 448.37
449.62 460.95 466.28 501.57 503.06 517.89
522.02 523.08 538.69 626.37 627.11 627.63
628.14 629.03 629.25 691.33 693.22 706.76
707.05 707.72 709.28 709.46 711.04 722.57
724.70 726.36 729.20 759.60 763.58 765.65
766.01 766.57 769.79 858.39 864.98 866.29
868.49 871.33 873.64 926.80 943.99 944.81
946.07 946.98 950.01 985.67 986.61 987.19
988.27 989.87 991.56 1006.56 1008.52 1010.53
1011.24 1012.03 1013.16 1016.20 1017.68 1018.56
1018.82 1019.15 1019.87 1047.08 1048.77 1049.17
1049.81 1051.34 1052.07 1101.22 1109.85 1111.99
1112.66 1113.79 1114.53 1115.78 1117.21 1120.55
1121.10 1124.99 1126.89 1199.84 1201.96 1202.28
1202.32 1203.43 1203.66 1218.34 1220.20 1221.96
1222.49 1223.46 1225.25 1322.02 1324.27 1330.29
1331.16 1332.96 1333.90 1363.18 1363.47 1363.77
1364.57 1366.80 1367.81 1468.13 1471.78 1476.42
1477.40 1477.97 1478.42 1514.14 1521.57 1523.52
1523.56 1524.11 1525.06 1606.92 1617.68 1625.72
1626.09 1626.41 1627.66 1631.28 1638.11 1641.91
1642.37 1643.32 1643.56 3136.30 3160.54 3170.98
3171.87 3178.41 3185.36 3187.20 3187.80 3188.73
3189.79 3189.87 3191.71 3194.21 3196.73 3197.64
3197.77 3200.53 3202.41 3203.00 3205.54 3205.83
3205.97 3207.55 3209.75 3211.82 3212.80 3214.24
3215.20 3215.31 3219.03
==============================
5
==============================
8.14 23.85 32.58 40.49 42.77 48.60
50.49 54.26 58.05 60.78 63.34 66.06
69.33 75.21 79.86 86.66 95.36 101.65
110.19 113.49 124.05 125.94 152.66 166.06
179.64 193.41 202.64 206.87 211.39 214.88
223.70 233.86 240.26 249.92 255.76 259.31
261.96 266.96 271.85 275.45 280.73 294.47
348.06 355.15 387.09 407.92 409.08 411.83
418.01 422.26 424.72 428.03 430.64 434.63
440.76 446.67 463.19 472.53 486.79 492.17
507.98 518.04 526.14 535.03 542.43 605.01
627.67 629.53 630.33 630.60 632.21 633.64
646.50 693.88 697.05 707.81 708.15 709.46
713.20 714.55 715.98 716.88 717.17 721.03
721.95 737.80 742.24 760.58 763.51 765.86
768.34 770.03 772.94 861.55 867.38 869.25
876.19 877.44 881.03 898.05 914.85 938.73
940.30 945.03 949.26 954.34 956.38 961.23
962.97 983.67 985.60 988.66 990.37 997.34
998.44 1000.38 1010.02 1012.38 1012.89 1013.35
1013.66 1013.86 1014.81 1016.29 1016.72 1018.63
1019.59 1020.23 1022.14 1038.78 1050.18 1051.12
1051.68 1052.42 1053.18 1054.21 1102.23 1109.04
1109.57 1110.92 1112.45 1115.63 1116.11 1117.05
1117.64 1118.43 1119.13 1121.77 1125.02 1192.40
1199.68 1201.25 1201.63 1202.15 1202.97 1203.05
1220.75 1222.89 1226.99 1229.16 1231.02 1235.99
1256.26 1298.96 1317.23 1321.47 1321.71 1326.79
1327.47 1334.83 1335.03 1361.41 1362.04 1366.71
1367.35 1367.96 1371.55 1372.18 1393.76 1453.80
1472.62 1473.00 1475.18 1476.15 1478.79 1479.76
1486.73 1505.91 1523.20 1525.12 1526.56 1526.93
1527.33 1530.33 1592.55 1623.84 1624.12 1625.77

1627.37 1629.39 1629.85 1641.23 1642.71 1642.75
1644.72 1645.25 1647.16 2063.62 2994.86 3034.12
3097.25 3107.56 3161.66 3165.41 3178.86 3180.64
3188.58 3189.14 3190.23 3190.33 3190.40 3190.54
3195.54 3196.95 3198.13 3199.14 3199.41 3200.97
3204.90 3205.12 3205.49 3207.13 3212.12 3212.81
3212.82 3214.53 3215.22 3215.81 3222.24 3222.83
3231.52 3235.98 3243.90 3257.59 3258.57 3412.61

==============================
5-TS
==============================

-316.25 14.68 23.70 32.15 32.54 44.75
49.77 51.89 53.51 56.69 61.24 64.13
69.28 73.81 75.67 76.96 84.65 95.82
97.27 102.97 106.10 114.53 123.04 154.71
163.62 196.38 201.91 209.20 215.19 217.78
232.40 242.76 244.45 255.46 257.18 262.62
268.78 275.50 277.41 287.30 308.84 323.21
357.29 406.54 411.02 413.26 414.11 418.75
426.00 433.21 436.60 445.35 452.52 461.81
466.03 470.78 489.54 506.33 509.85 518.75
523.29 531.59 534.43 549.12 584.84 628.68
630.01 630.28 631.18 631.58 632.40 658.91
693.47 697.87 708.05 710.11 710.87 712.99
713.45 714.63 718.35 719.61 720.12 726.84
758.14 763.91 766.19 768.18 768.60 771.80
772.02 775.02 862.47 873.58 873.91 874.58
878.03 879.14 904.09 935.56 942.81 952.06
954.17 954.80 956.21 959.16 964.43 973.97
986.65 990.70 994.27 995.66 995.76 997.21
999.30 1001.01 1012.92 1013.14 1013.35 1014.03
1014.81 1015.90 1016.34 1017.20 1018.49 1018.73
1019.55 1019.74 1020.75 1050.89 1052.11 1052.88
1052.99 1053.59 1053.88 1091.54 1111.78 1112.92
1113.46 1115.35 1116.32 1116.68 1118.14 1119.78
1120.64 1121.74 1123.59 1124.12 1126.80 1150.73
1197.96 1201.54 1201.82 1202.34 1202.59 1202.79
1203.07 1223.14 1224.91 1226.71 1227.08 1230.26
1231.44 1241.31 1305.03 1323.88 1325.49 1328.24
1332.88 1333.17 1334.82 1335.42 1361.79 1365.74
1366.15 1366.62 1367.13 1369.35 1371.53 1409.40
1474.06 1474.35 1476.34 1477.60 1479.67 1480.44
1486.91 1489.13 1509.41 1524.94 1525.17 1525.59
1525.77 1527.08 1527.78 1624.76 1625.56 1627.01
1627.11 1628.36 1628.67 1642.53 1643.06 1644.12
1644.37 1644.73 1644.98 1755.05 2961.49 2986.03
3104.65 3110.48 3112.25 3150.49 3170.99 3187.27
3187.76 3187.86 3187.99 3188.08 3189.82 3195.18
3195.84 3195.91 3196.63 3197.88 3197.99 3204.30
3206.81 3208.29 3208.82 3209.36 3209.95 3212.63
3214.01 3214.83 3215.43 3217.23 3217.43 3217.96
3218.64 3221.17 3223.86 3225.94 3238.19 3269.58

==============================
6
==============================

2.11 5.54 10.19 19.92 26.49 28.56
34.43 37.11 41.65 46.40 48.46 51.79
55.59 56.41 62.41 64.16 71.80 78.08
83.83 96.31 103.25 125.31 142.04 150.47
175.53 190.53 195.13 198.92 203.88 220.19
226.67 231.13 244.14 245.36 253.77 256.61
263.70 267.25 275.36 277.28 311.90 324.95
404.29 405.42 406.88 407.24 413.50 414.75
420.09 430.07 445.77 450.33 461.47 465.44
502.94 507.72 517.34 520.26 526.16 534.34
540.33 579.01 617.78 627.21 628.51 629.38
629.92 630.63 630.71 645.20 676.55 695.25
695.76 706.38 707.08 709.10 710.05 710.12
712.27 720.99 722.39 725.34 728.21 762.84
763.16 763.79 766.32 768.61 769.39 771.22
781.49 817.44 863.18 865.36 866.81 869.47
871.47 875.88 935.67 940.16 944.08 944.46
946.51 946.77 954.94 984.12 984.52 986.31
987.69 990.74 991.31 994.45 999.95 1011.65
1011.98 1012.78 1013.13 1013.32 1013.89 1014.98
1016.98 1018.19 1019.01 1019.12 1019.56 1020.47
1050.86 1051.19 1051.53 1051.87 1052.36 1052.59
1060.55 1071.39 1111.43 1112.55 1113.08 1114.39
1115.24 1116.92 1117.31 1117.55 1118.24 1120.31
1121.36 1123.64 1125.21 1144.32 1157.88 1200.49
1202.25 1202.52 1202.70 1202.76 1202.80 1203.90
1222.02 1222.40 1223.59 1224.90 1225.54 1226.88
1230.93 1248.22 1305.95 1323.20 1324.16 1328.12
1328.99 1330.20 1334.97 1335.05 1351.64 1364.11
1364.24 1365.98 1366.64 1367.85 1369.74 1396.18
1473.64 1473.94 1476.87 1477.77 1479.49 1479.94
1486.04 1512.72 1523.12 1523.18 1524.23 1524.32
1526.44 1527.04 1529.75 1621.58 1622.18 1627.13
1627.41 1628.21 1628.57 1640.72 1640.75 1643.24
1643.64 1643.85 1644.91 1691.08 2960.29 2990.65
3055.83 3065.40 3075.80 3116.01 3139.03 3146.84
3156.63 3159.63 3181.79 3181.86 3185.02 3188.03
3188.41 3189.01 3189.52 3189.80 3191.39 3194.72
3196.41 3197.12 3197.41 3197.56 3200.60 3203.53
3204.85 3205.05 3205.70 3206.45 3208.76 3212.38
3213.00 3213.74 3213.78 3214.31 3215.29 3222.12

==============================
7
==============================

9.47 22.44 23.48 34.44 36.15 43.57
49.98 52.97 54.85 62.30 67.95 72.60
73.33 85.76 97.34 108.88 120.37 136.19
148.34 155.53 158.43 175.12 199.80 208.42
216.45 221.07 228.71 246.00 248.46 266.90
269.45 271.63 282.64 315.71 334.46 346.87
371.39 404.67 407.48 413.48 424.06 428.62
456.12 460.78 509.62 511.66 528.92 534.99
542.81 563.92 604.49 627.83 629.12 629.87
636.36 671.48 681.11 695.95 707.44 709.16
711.78 719.79 725.52 747.06 761.88 764.52
769.92 771.23 782.33 824.81 841.43 862.07
864.03 879.48 940.23 941.06 947.30 961.24
966.02 974.19 986.31 987.99 1002.00 1006.45
1009.07 1010.98 1013.90 1014.24 1017.17 1020.16
1026.84 1032.65 1043.02 1048.98 1051.85 1052.75
1054.53 1075.93 1081.42 1107.49 1114.55 1116.23
1117.06 1119.34 1120.33 1122.76 1145.60 1162.16
1177.40 1201.01 1202.91 1203.81 1204.65 1212.87
1214.30 1224.80 1225.86 1230.19 1251.79 1287.55
1291.24 1321.15 1327.11 1333.80 1335.42 1342.71
1366.56 1367.46 1373.56 1389.34 1411.30 1468.38
1469.25 1474.56 1477.98 1478.75 1480.13 1480.63

1496.59 1499.72 1499.95 1524.28 1526.04 1526.32
1528.54 1624.43 1628.05 1629.52 1641.45 1642.33
1644.91 1645.41 1791.44 1952.71 2978.09 3003.52
3040.78 3048.76 3059.50 3072.47 3089.27 3127.99
3129.12 3137.17 3160.22 3168.51 3173.07 3175.16
3176.11 3190.98 3193.25 3193.53 3195.46 3199.55
3203.08 3203.22 3209.61 3212.49 3213.06 3217.13
3219.55 3219.88 3220.52

==============================

7-TS

==============================

-287.15 20.79 26.69 33.89 46.38 46.70
51.73 60.09 63.59 70.80 72.74 81.00
85.08 96.33 108.73 116.27 119.39 134.65
144.03 155.67 162.96 167.20 180.49 204.19
212.63 219.50 221.90 238.14 254.56 261.80
269.42 281.53 294.63 300.26 312.41 322.60
355.65 365.18 400.79 409.30 414.11 423.03
438.52 446.42 468.80 477.13 481.08 501.71
510.03 522.67 538.96 547.20 600.17 628.46
629.24 630.49 645.04 662.00 697.06 710.87
711.96 713.59 721.08 724.05 747.96 756.36
767.22 768.72 771.64 787.56 839.11 871.04
874.12 877.87 889.74 936.54 950.30 951.73
958.80 960.71 970.40 992.20 993.50 993.76
996.85 999.17 1005.06 1012.35 1013.68 1014.44
1019.96 1022.41 1023.08 1024.24 1028.83 1051.63
1052.69 1053.33 1056.30 1083.34 1097.11 1114.22
1116.52 1116.76 1118.19 1119.45 1124.90 1134.77
1144.06 1178.85 1199.50 1203.63 1204.27 1204.82
1215.42 1226.81 1228.03 1234.51 1240.02 1289.99
1302.69 1327.07 1329.10 1334.71 1337.53 1362.32
1368.57 1368.93 1375.10 1404.03 1413.03 1471.62
1474.39 1475.00 1475.37 1479.72 1480.60 1490.79
1493.43 1495.70 1504.15 1514.03 1525.35 1526.36
1528.92 1624.52 1625.87 1627.88 1640.76 1642.40
1642.60 1738.63 1791.38 2062.48 2996.29 3000.29
3045.18 3072.65 3107.32 3120.12 3121.57 3122.44
3123.42 3146.94 3158.80 3175.33 3188.16 3190.79
3191.85 3194.51 3195.50 3198.25 3202.86 3205.94
3206.51 3213.16 3214.41 3217.03 3219.61 3219.90
3221.06 3247.51 3292.48

==============================

8A

==============================

12.93 23.77 28.58 35.70 39.97 44.08
48.65 51.88 55.78 59.61 62.63 63.43
65.15 68.78 73.92 75.88 81.29 83.43
86.52 97.79 101.98 104.04 107.49 110.63
114.41 119.60 126.51 133.66 138.24 151.90
159.41 166.06 175.19 186.95 203.28 209.37
212.66 223.93 227.83 237.78 241.72 250.34
255.98 258.39 261.84 266.42 270.26 274.61
279.68 280.68 287.31 289.45 305.71 344.13
354.84 400.66 410.39 411.14 413.77 418.37
421.62 424.10 429.50 431.53 440.12 447.38
459.50 466.89 469.58 498.92 505.73 516.97
520.36 532.98 535.73 537.57 555.47 606.24
618.68 627.82 628.45 628.99 629.53 629.83
632.09 632.65 673.59 692.56 697.25 706.67
710.34 712.36 712.65 715.26 716.48 716.80
717.99 720.76 723.43 744.52 761.17 762.13
762.83 767.35 770.00 770.96 779.40 821.32
838.90 845.89 866.42 869.47 872.26 874.51
875.80 877.06 937.50 945.02 945.68 949.29
950.99 952.30 952.45 956.59 981.05 988.52
988.99 990.53 994.25 994.76 997.28 1011.54
1012.35 1012.38 1013.92 1014.33 1015.21 1015.38
1015.60 1016.28 1016.98 1018.56 1020.10 1020.64
1020.99 1024.08 1049.55 1051.22 1051.97 1053.44
1053.69 1054.53 1055.88 1058.60 1075.27 1087.68
1109.84 1110.83 1112.91 1114.05 1114.96 1115.94
1116.99 1118.01 1119.57 1119.82 1122.03 1122.85
1123.87 1142.40 1167.66 1180.64 1200.89 1201.37
1201.46 1202.10 1202.77 1203.01 1208.10 1222.31
1223.58 1225.97 1227.93 1228.65 1230.78 1233.29
1233.84 1254.83 1278.27 1298.87 1322.88 1324.02
1327.13 1331.17 1334.21 1335.44 1335.89 1351.85
1363.84 1365.89 1366.50 1367.76 1370.99 1372.40
1397.87 1405.83 1466.21 1472.25 1473.13 1475.99
1476.70 1478.14 1478.22 1479.61 1479.93 1486.44
1496.72 1503.12 1508.70 1525.15 1527.03 1527.50
1527.84 1528.81 1529.13 1529.38 1624.18 1626.13
1627.19 1627.89 1628.37 1629.36 1642.97 1643.67
1644.51 1645.10 1645.36 1646.63 1698.66 1809.67
2150.65 2985.18 3008.42 3030.44 3048.83 3069.93
3074.20 3117.31 3119.08 3123.70 3137.98 3140.18
3161.65 3187.21 3187.83 3188.02 3188.28 3188.83
3190.90 3191.30 3193.85 3194.41 3196.83 3197.00
3197.16 3202.38 3202.69 3204.26 3206.86 3209.01
3209.41 3209.92 3210.83 3213.39 3214.39 3215.22
3217.71 3218.30 3219.52 3220.24 3223.80 3224.90
3245.35 3251.17 3256.51

==============================

8B

==============================

16.76 27.51 30.83 32.54 42.61 46.23
49.07 51.59 56.53 57.50 60.25 63.31
66.90 68.06 68.96 76.54 81.77 84.27
88.84 90.62 97.64 104.11 110.00 113.58
115.34 123.25 127.38 133.20 135.36 142.13
159.48 165.96 170.24 180.14 198.57 206.88
210.03 224.53 229.51 236.78 240.69 243.77
253.95 257.88 259.71 265.25 266.35 272.04
276.65 282.13 288.36 297.76 309.49 334.27
354.90 375.25 408.40 410.17 414.44 417.29
419.12 424.29 426.75 434.16 445.24 452.56
463.10 465.89 473.78 501.13 509.99 514.78
523.32 533.35 538.84 540.80 563.25 569.35
619.18 630.09 630.26 630.61 630.90 631.88
632.80 634.88 674.63 693.53 697.64 708.28
710.48 712.25 713.44 715.05 715.68 718.48
719.89 721.74 723.17 740.47 764.65 764.86
766.10 766.95 769.44 771.45 773.44 819.32
820.10 839.14 869.13 870.93 873.72 875.03
876.29 883.86 934.48 944.39 946.32 950.12
951.99 952.60 954.63 960.58 983.30 990.55
990.97 992.86 994.50 996.54 1005.88 1012.05
1013.36 1014.48 1014.90 1015.29 1016.06 1016.32
1017.47 1017.73 1019.02 1019.44 1019.97 1022.85
1027.81 1036.81 1051.33 1052.00 1052.41 1052.98
1053.79 1054.43 1059.13 1062.34 1071.80 1092.19

1110.00 1110.73 1111.90 1114.69 1115.27 1116.62
1117.58 1118.51 1119.07 1120.48 1121.20 1121.78
1122.65 1144.60 1177.26 1178.84 1201.44 1201.66
1201.92 1202.13 1203.23 1203.24 1209.90 1217.70
1224.33 1226.88 1228.23 1229.62 1230.50 1232.95
1233.89 1253.80 1259.32 1300.96 1322.41 1324.70
1329.62 1329.85 1333.65 1334.18 1335.95 1353.26
1364.30 1366.35 1368.08 1369.77 1369.87 1372.93
1399.30 1415.32 1470.14 1472.78 1473.63 1476.03
1476.13 1477.66 1478.45 1480.26 1480.63 1490.26
1496.60 1503.93 1508.45 1526.48 1526.85 1527.69
1528.45 1528.87 1529.33 1530.11 1624.14 1625.64
1625.96 1627.12 1627.91 1629.00 1641.96 1642.83
1643.67 1644.84 1645.38 1646.91 1706.36 1832.94
2263.13 2975.32 2997.02 3015.87 3042.64 3068.95
3070.88 3095.22 3107.98 3114.91 3125.09 3137.06
3152.35 3163.00 3183.01 3184.11 3185.64 3186.74
3186.98 3187.64 3191.16 3191.71 3192.56 3192.80
3193.65 3194.19 3197.17 3202.35 3203.36 3203.80
3206.54 3206.73 3208.68 3208.75 3208.97 3212.43
3214.27 3214.82 3215.57 3216.21 3219.90 3222.86
3225.94 3255.03 3256.93

==============================

8C

==============================

7.29 11.07 22.01 28.30 42.26 42.79
46.08 47.85 50.64 53.42 59.38 61.33
63.05 68.10 68.83 75.90 80.12 88.18
91.92 93.61 99.96 108.53 114.43 120.82
124.96 135.61 136.96 143.87 145.36 157.48
164.20 165.98 178.81 189.74 199.97 206.29
210.09 219.07 225.83 230.77 238.12 247.99
256.12 259.31 260.04 262.32 264.33 275.91
279.58 281.91 285.07 301.59 326.65 335.68
349.14 380.22 409.63 411.97 416.94 417.33
421.06 425.31 428.39 433.33 442.20 448.56
463.21 468.36 476.30 498.61 505.57 517.72
521.62 531.12 537.57 544.80 566.50 574.22
627.65 629.40 630.23 631.74 632.57 633.03
633.31 651.14 681.15 695.46 696.38 706.68
710.70 712.03 714.28 714.77 715.25 715.96
720.97 723.39 727.33 740.04 759.95 763.13
767.65 768.70 770.22 771.41 784.10 798.71
818.03 834.78 858.67 868.36 872.10 875.51
877.46 884.98 929.05 939.77 941.17 945.78
951.56 953.43 955.17 964.90 983.65 989.05
993.35 997.47 997.85 998.16 1003.86 1007.81
1012.84 1013.58 1014.24 1015.01 1015.09 1015.72
1015.87 1016.62 1018.03 1019.50 1019.58 1020.23
1022.25 1046.88 1052.92 1053.32 1053.32 1053.92
1054.25 1054.34 1054.85 1069.15 1072.08 1085.35
1108.40 1112.31 1112.96 1114.22 1115.36 1117.31
1119.05 1119.64 1120.83 1121.04 1121.64 1123.44
1123.92 1157.54 1180.35 1188.41 1202.77 1202.81
1202.98 1203.47 1204.10 1204.15 1211.42 1217.56
1226.00 1227.70 1228.27 1230.13 1231.30 1233.14
1235.00 1253.85 1258.74 1311.09 1319.12 1324.13
1328.43 1330.31 1333.85 1335.49 1338.04 1356.44
1366.73 1366.92 1367.63 1370.24 1371.20 1372.67
1401.62 1417.26 1464.18 1471.55 1473.36 1473.86
1476.35 1477.75 1477.88 1480.80 1481.61 1491.34
1492.00 1505.55 1506.58 1527.45 1527.51 1527.89
1528.76 1529.29 1529.72 1532.79 1623.98 1625.44
1627.75 1628.86 1630.22 1630.39 1644.16 1644.35
1645.36 1645.84 1646.28 1646.76 1702.27 1830.98
2315.51 2986.39 3004.64 3046.50 3048.28 3051.73
3066.62 3085.34 3106.31 3110.23 3127.24 3129.29
3130.22 3183.25 3185.49 3185.78 3186.39 3186.99
3187.61 3188.73 3192.29 3193.04 3193.53 3193.85
3194.71 3195.63 3196.31 3202.43 3203.99 3204.11
3205.73 3209.81 3210.36 3211.72 3213.14 3213.44
3213.93 3217.56 3218.41 3223.29 3227.40 3229.42
3230.10 3231.97 3232.07

==============================

8C-BPR

==============================

-47.44 17.50 24.24 29.37 33.93 36.74
41.78 46.33 50.58 51.89 56.85 58.92
61.20 65.95 67.80 71.42 74.47 79.05
83.11 87.02 89.70 95.67 98.88 104.07
113.08 114.10 119.02 122.52 131.77 145.35
150.41 159.48 177.40 195.30 200.97 202.54
207.70 211.02 217.96 221.30 226.21 232.51
245.23 253.74 255.12 257.69 258.20 260.89
264.05 277.59 278.37 298.39 307.01 331.87
335.75 360.51 411.83 413.74 415.38 415.75
418.33 423.70 429.67 436.80 438.29 453.64
460.52 469.20 484.51 494.79 504.74 516.38
524.06 526.28 534.37 539.62 562.21 575.41
618.39 627.53 628.03 628.80 630.39 630.84
632.00 642.15 677.01 694.39 695.50 709.50
710.11 711.77 712.71 715.06 715.84 718.12
718.38 719.16 725.97 740.80 764.19 764.96
767.25 767.40 771.71 772.86 778.86 797.04
826.91 835.09 870.05 872.11 873.25 874.59
878.10 886.12 930.97 948.07 951.75 952.40
954.36 957.19 962.67 966.33 991.00 991.73
993.55 995.04 995.91 996.30 1003.28 1005.40
1008.45 1013.30 1013.41 1014.16 1014.68 1014.97
1016.18 1016.54 1017.34 1017.66 1019.23 1021.45
1023.55 1038.90 1048.13 1049.59 1050.40 1051.86
1052.28 1053.26 1063.57 1073.63 1076.15 1101.33
1110.05 1111.67 1113.27 1114.54 1115.13 1116.40
1117.43 1118.07 1118.85 1119.83 1120.43 1122.71
1127.41 1157.41 1176.09 1176.15 1199.80 1201.63
1201.96 1202.52 1203.12 1203.58 1206.24 1218.04
1222.68 1224.54 1229.04 1229.19 1231.64 1232.41
1234.75 1254.40 1297.66 1311.83 1321.28 1322.87
1327.40 1329.50 1330.55 1333.75 1334.51 1350.26
1361.59 1366.99 1368.29 1369.08 1371.62 1372.50
1396.56 1418.29 1470.81 1472.40 1474.25 1475.09
1475.99 1476.83 1478.13 1479.78 1481.43 1489.89
1491.31 1500.44 1506.54 1523.88 1526.52 1526.99
1527.58 1528.30 1528.36 1532.67 1622.72 1622.79
1625.89 1627.68 1628.42 1628.91 1641.67 1641.98
1642.41 1643.37 1643.99 1645.19 1692.76 1790.64
2302.27 2993.76 3004.97 3018.02 3041.30 3068.54
3069.86 3111.16 3115.64 3122.84 3127.95 3151.95
3179.82 3183.79 3184.57 3185.09 3185.21 3188.71
3190.10 3190.45 3191.02 3192.33 3193.57 3193.90
3195.25 3196.24 3196.73 3200.53 3200.65 3201.15
3203.70 3204.27 3206.81 3207.61 3208.98 3209.24

3210.05 3211.25 3213.20 3214.84 3215.02 3215.56
3216.88 3218.39 3264.31
==============================
8A-TS1
==============================
-193.65 10.30 25.26 29.00 33.57 39.81
44.75 47.53 52.91 53.89 57.36 60.77
63.75 67.12 69.76 75.01 75.29 78.87
79.86 88.83 90.25 96.84 98.77 102.30
113.32 114.50 129.37 138.96 148.20 155.16
168.05 174.33 180.33 185.62 206.12 209.65
215.42 218.26 221.84 226.53 233.31 241.82
255.38 256.14 259.39 260.66 267.92 273.06
274.35 282.14 286.40 288.94 306.28 336.86
374.65 406.83 408.52 410.63 414.46 418.55
420.68 423.79 426.44 433.71 435.39 439.68
467.56 470.47 481.95 499.24 503.59 517.84
520.26 530.57 533.70 534.92 557.40 612.63
627.51 628.01 630.01 630.75 631.52 632.01
632.79 674.05 683.42 692.22 693.97 704.84
706.68 709.31 710.64 711.50 713.63 714.27
716.25 720.39 721.70 752.25 757.98 761.11
764.95 765.49 767.23 769.58 771.95 817.19
848.10 860.01 864.00 865.75 870.06 870.54
881.45 883.64 935.27 942.18 942.32 943.99
945.23 947.81 953.52 961.53 981.01 981.92
985.05 987.98 989.42 990.71 1002.34 1008.32
1011.71 1011.94 1012.39 1013.48 1013.99 1014.71
1015.37 1015.66 1016.83 1018.22 1020.66 1021.86
1023.22 1024.15 1048.36 1051.76 1052.52 1053.63
1054.19 1054.77 1055.17 1062.69 1082.21 1083.94
1107.05 1108.74 1111.41 1114.50 1115.67 1115.88
1117.40 1118.90 1119.31 1119.82 1121.18 1121.86
1124.74 1139.84 1166.51 1181.23 1200.89 1202.03
1202.25 1202.33 1203.42 1203.71 1207.85 1219.75
1221.82 1226.66 1226.76 1229.39 1230.10 1236.55
1237.16 1258.62 1263.78 1290.71 1322.58 1324.08
1327.67 1328.87 1331.57 1335.28 1336.56 1349.54
1363.84 1365.61 1366.56 1368.20 1368.61 1374.64
1396.24 1405.92 1468.11 1473.77 1474.29 1475.75
1476.02 1479.34 1479.85 1480.09 1480.63 1486.55
1497.06 1504.10 1505.87 1524.69 1526.18 1526.52
1527.60 1527.92 1528.27 1531.49 1625.30 1626.84
1627.08 1628.86 1629.47 1630.54 1642.90 1644.47
1644.96 1645.17 1646.10 1646.85 1662.32 1803.53
1994.66 2979.15 3025.18 3029.95 3040.19 3068.15
3068.74 3108.77 3114.87 3119.57 3132.64 3152.79
3154.80 3156.45 3172.50 3181.32 3183.53 3184.19
3186.60 3186.78 3187.79 3188.66 3188.87 3193.19
3194.13 3194.83 3196.56 3198.12 3198.84 3202.27
3202.69 3204.50 3205.87 3206.35 3209.62 3211.68
3212.12 3213.58 3213.96 3218.54 3223.98 3229.02
3237.26 3256.81 3273.75
==============================
8A-TS2
==============================
-228.41 15.47 18.01 31.24 36.05 38.38
43.21 44.78 46.74 52.02 54.64 56.77
59.98 62.50 65.77 71.66 73.41 76.45
80.57 83.48 91.93 95.90 99.00 103.23
106.14 115.19 128.53 130.16 135.58 152.31
161.37 164.36 170.65 194.79 201.94 206.93
210.47 216.33 219.71 222.69 229.92 232.43
241.81 254.40 256.17 259.12 261.76 262.32
278.10 280.88 283.48 296.92 337.61 348.19
396.93 404.93 406.65 410.81 415.72 418.59
421.61 425.51 434.96 438.41 440.06 454.97
461.33 467.32 479.28 498.45 505.11 516.09
519.78 532.32 539.52 561.22 570.95 628.47
629.80 630.34 631.07 631.62 633.16 633.82
650.24 663.60 679.39 694.40 698.09 705.50
707.79 709.12 710.44 712.86 714.23 717.92
719.94 722.54 725.15 747.64 760.32 762.82
765.55 767.81 768.98 770.37 775.32 821.12
831.06 849.18 863.58 864.94 868.13 873.73
877.51 878.46 936.13 940.35 941.40 944.42
950.29 956.59 958.43 980.30 982.89 984.36
987.02 989.84 994.55 995.82 997.30 1004.46
1008.35 1011.93 1013.60 1013.70 1013.95 1014.21
1015.29 1015.70 1016.40 1016.88 1017.81 1018.97
1020.90 1042.86 1051.07 1051.43 1051.84 1053.58
1053.97 1054.04 1054.49 1059.03 1077.53 1082.17
1107.94 1109.35 1111.85 1113.38 1115.04 1117.21
1117.77 1118.18 1119.54 1119.88 1122.10 1123.36
1123.73 1138.64 1177.03 1177.97 1199.43 1200.09
1201.88 1202.59 1202.88 1203.34 1206.28 1213.24
1219.97 1223.07 1225.16 1227.58 1229.24 1230.26
1234.87 1249.11 1261.82 1290.40 1325.32 1326.17
1327.24 1329.75 1335.96 1337.12 1337.61 1353.76
1362.01 1363.57 1368.61 1369.77 1370.80 1374.45
1395.90 1402.39 1470.41 1473.77 1474.61 1475.50
1476.48 1476.97 1478.87 1480.52 1480.83 1481.74
1495.52 1504.29 1511.98 1524.66 1526.62 1527.60
1528.09 1528.37 1529.76 1530.74 1625.79 1626.41
1627.58 1627.84 1629.83 1630.60 1643.38 1644.30
1644.79 1645.29 1645.65 1647.39 1665.16 1779.99
1882.69 2972.83 3010.63 3039.82 3041.36 3052.36
3061.45 3065.91 3113.67 3121.83 3127.20 3150.79
3154.98 3169.18 3178.71 3182.36 3182.92 3183.37
3185.23 3186.95 3187.65 3188.46 3191.07 3191.84
3192.20 3192.76 3196.63 3198.97 3199.73 3202.33
3202.79 3205.08 3209.32 3209.61 3210.62 3212.20
3213.74 3215.81 3225.14 3225.90 3227.59 3230.73
3230.74 3234.28 3245.15
==============================
8B-TS1
==============================
-128.87 2.31 8.95 29.29 35.74 35.86
40.24 46.63 48.29 50.60 52.50 54.94
55.58 59.43 61.39 68.36 70.96 73.99
79.40 81.28 91.41 96.22 100.84 110.46
116.68 119.72 132.48 136.17 143.14 146.28
155.86 167.14 177.61 192.34 196.77 202.32
210.17 224.63 228.39 233.58 236.69 239.78
250.30 252.45 254.69 258.86 261.98 272.31
277.27 281.37 282.65 296.98 307.80 353.43
380.91 407.35 409.63 410.48 412.26 420.69
422.18 426.37 427.75 434.60 438.98 441.74
463.58 466.74 502.21 508.72 513.84 516.47
518.99 529.94 535.56 537.57 566.93 609.48
626.54 627.26 628.77 630.28 630.94 631.46
632.60 661.58 677.14 695.31 697.44 706.90

708.22 711.60 712.30 712.58 714.02 715.63
717.08 721.45 721.82 744.17 761.59 763.42
764.84 765.68 767.69 767.85 770.43 816.07
848.58 863.80 865.81 867.44 870.07 873.98
875.20 876.80 940.62 943.52 943.99 945.84
946.09 949.90 953.01 956.52 982.08 985.15
986.60 988.04 988.87 991.90 994.42 1008.38
1009.61 1012.45 1012.54 1013.54 1014.50 1014.94
1016.18 1016.66 1017.18 1017.52 1017.97 1019.70
1021.74 1033.38 1048.01 1050.99 1051.35 1051.82
1052.48 1053.02 1053.36 1058.12 1080.58 1083.47
1111.27 1112.77 1114.10 1114.33 1114.95 1116.60
1117.34 1118.49 1118.67 1119.52 1120.15 1121.54
1122.68 1141.35 1159.53 1177.72 1200.44 1200.84
1201.82 1203.05 1203.18 1203.25 1208.47 1215.16
1221.70 1223.12 1225.94 1226.60 1228.92 1230.12
1231.48 1255.11 1286.42 1293.96 1324.81 1325.75
1327.18 1329.19 1331.71 1336.89 1338.71 1349.11
1364.39 1365.07 1366.61 1367.63 1368.69 1371.11
1396.41 1409.01 1471.92 1473.48 1475.08 1475.35
1476.97 1477.82 1479.99 1480.92 1481.11 1485.20
1495.05 1505.97 1506.85 1525.40 1525.68 1526.61
1526.78 1527.12 1527.61 1528.61 1625.22 1626.23
1627.22 1628.70 1630.97 1631.13 1642.91 1643.71
1644.54 1645.70 1647.14 1647.60 1668.77 1799.64
1954.68 2989.26 3011.51 3032.20 3053.26 3067.08
3079.46 3105.16 3121.20 3131.94 3136.12 3149.97
3170.56 3176.47 3180.72 3181.58 3183.50 3184.25
3185.55 3186.22 3187.40 3189.23 3189.85 3190.89
3192.64 3192.96 3196.09 3198.29 3199.18 3199.56
3201.77 3206.58 3208.34 3209.56 3209.69 3210.21
3212.16 3214.54 3215.52 3217.39 3219.51 3220.78
3221.53 3224.77 3248.66

==============================

8B-TS2

==============================

-212.69 7.23 12.85 16.95 24.85 34.83
38.33 43.35 47.94 50.42 51.95 55.19
60.37 64.06 66.58 67.82 70.15 78.58
79.72 81.63 86.21 93.37 97.46 103.06
104.93 115.40 121.91 125.35 130.24 139.29
148.08 169.10 189.60 196.96 202.42 205.47
214.70 217.32 223.35 229.01 230.61 243.52
253.48 254.39 256.60 259.09 260.66 262.20
277.10 280.23 285.99 297.85 330.86 346.03
364.47 404.97 406.68 410.13 412.80 415.22
419.77 421.53 422.41 434.38 436.85 443.85
458.07 466.80 488.56 499.19 510.73 514.40
518.05 525.78 532.33 539.72 568.87 626.17
628.60 629.01 630.00 631.20 632.46 633.15
658.33 678.85 693.94 697.25 705.38 707.93
708.53 709.65 712.13 713.10 715.26 717.10
724.88 726.46 736.39 757.65 761.48 763.98
765.96 768.71 769.35 769.59 772.83 820.31
837.01 849.24 865.42 866.53 870.41 872.59
874.84 883.53 933.19 942.34 942.66 950.68
951.86 955.03 956.84 978.23 983.91 985.29
985.85 990.66 991.59 994.77 996.13 1001.32
1009.49 1011.04 1011.66 1012.91 1013.50 1014.08
1015.51 1016.23 1016.47 1016.83 1016.98 1019.92
1020.21 1038.92 1049.88 1051.17 1052.03 1052.45
1052.71 1052.91 1053.95 1058.96 1076.61 1081.56
1107.26 1110.19 1111.36 1113.88 1114.76 1115.31
1115.71 1116.44 1119.00 1119.96 1120.83 1122.44
1126.62 1132.26 1169.27 1174.99 1200.20 1200.56
1201.30 1202.05 1202.36 1202.69 1205.43 1210.82
1220.41 1223.11 1224.76 1227.51 1228.41 1232.54
1233.80 1252.69 1272.49 1291.93 1323.41 1325.72
1326.67 1328.62 1334.81 1336.22 1336.58 1355.50
1362.77 1363.63 1368.38 1369.35 1373.12 1374.18
1396.21 1410.06 1468.05 1471.80 1473.26 1474.03
1475.11 1476.18 1479.16 1480.05 1480.44 1491.00
1494.05 1504.31 1512.60 1525.53 1525.94 1526.20
1528.09 1528.50 1529.78 1530.26 1623.89 1625.42
1626.32 1626.81 1629.46 1630.06 1641.78 1643.93
1644.04 1644.67 1646.14 1646.86 1665.08 1789.80
1917.87 2969.33 3013.98 3033.43 3042.20 3054.57
3065.24 3068.86 3115.98 3116.96 3126.40 3151.37
3151.84 3169.55 3182.82 3183.19 3184.82 3185.37
3186.58 3187.08 3188.18 3188.43 3190.62 3190.85
3192.83 3194.44 3194.89 3199.09 3200.38 3201.12
3203.09 3204.51 3205.77 3208.35 3210.93 3211.62
3212.28 3212.66 3216.07 3226.54 3227.78 3228.07
3235.01 3244.96 3254.20

==============================

9A

==============================

21.87 27.80 30.11 38.04 41.89 48.31
49.11 51.95 58.12 60.42 64.43 67.49
70.27 73.34 73.60 79.09 82.75 86.24
91.01 93.39 94.62 98.83 103.83 106.13
118.08 123.50 130.64 150.04 154.47 172.35
179.11 191.61 196.25 197.85 202.23 204.76
209.26 219.27 227.16 230.02 238.96 250.58
253.86 257.71 261.31 263.56 278.18 278.68
282.95 312.23 317.65 343.16 368.52 404.32
408.93 411.12 412.08 415.65 416.65 423.61
428.08 432.72 437.18 443.02 451.57 459.61
472.56 493.28 500.08 506.72 514.74 522.28
528.88 537.73 541.71 576.76 616.04 630.03
630.70 630.98 631.93 632.50 633.36 647.98
695.73 698.84 705.80 707.96 708.49 709.34
710.01 711.27 712.32 714.96 717.16 719.47
722.95 744.56 756.33 759.64 762.07 763.83
765.82 767.23 768.47 792.25 798.33 858.19
862.88 865.01 868.42 868.93 871.62 901.46
915.13 934.51 938.99 942.16 946.19 948.10
948.38 952.80 957.09 962.63 982.51 983.89
987.31 988.70 989.37 990.34 1007.27 1007.72
1011.49 1012.20 1013.58 1014.23 1014.73 1015.34
1016.30 1016.70 1017.59 1017.90 1018.20 1018.77
1027.31 1049.87 1051.66 1052.42 1052.50 1053.02
1053.37 1053.51 1068.84 1080.07 1104.89 1110.93
1111.82 1113.29 1114.26 1115.35 1116.34 1117.48
1117.70 1118.57 1120.05 1121.04 1122.24 1123.13
1155.10 1179.80 1181.01 1199.79 1200.71 1201.06
1201.35 1201.78 1202.79 1216.82 1219.81 1222.89
1224.44 1225.31 1227.63 1230.20 1231.32 1236.81
1270.86 1283.13 1289.14 1315.76 1324.35 1324.80
1327.69 1328.50 1334.85 1335.12 1338.83 1355.28
1361.26 1363.45 1363.91 1365.99 1368.46 1370.37
1400.37 1417.53 1473.11 1474.69 1474.90 1475.22

1478.21 1479.86 1480.72 1483.22 1487.76 1491.87
1498.66 1501.48 1502.97 1524.67 1525.16 1525.52
1525.94 1526.95 1527.79 1529.46 1620.48 1624.66
1625.74 1626.09 1626.57 1627.98 1628.83 1642.06
1643.01 1643.82 1644.15 1644.51 1645.95 1680.52
1763.17 2944.02 2982.89 3039.70 3046.50 3065.00
3078.53 3098.02 3118.88 3129.16 3131.35 3144.32
3144.50 3172.08 3172.60 3182.31 3185.69 3186.66
3187.73 3189.17 3189.63 3190.63 3191.20 3195.62
3196.15 3197.09 3198.19 3199.19 3202.08 3203.02
3204.66 3208.30 3209.49 3209.78 3211.20 3212.93
3213.79 3215.41 3216.95 3217.76 3219.81 3224.85
3232.42 3233.01 3262.07

==============================

9B

==============================

11.87 17.85 21.17 28.65 34.55 35.78
41.36 43.98 48.68 49.89 53.29 55.47
58.09 66.98 68.26 73.98 83.85 84.02
87.22 91.57 97.89 105.47 114.12 115.02
122.76 129.30 132.21 137.32 153.50 154.82
161.07 180.64 184.45 198.66 203.03 214.19
215.29 218.29 232.88 239.82 242.18 257.02
259.22 260.81 263.91 264.95 274.43 286.11
298.51 314.11 323.16 350.20 371.77 399.85
405.25 408.88 416.38 417.35 418.56 423.29
424.74 431.70 435.97 447.57 458.10 471.53
475.75 482.76 502.22 509.47 515.09 522.12
530.03 535.12 547.72 607.80 615.99 626.70
628.58 630.34 630.73 631.29 632.66 634.68
681.29 692.01 695.11 702.32 705.16 709.87
712.33 715.42 715.86 719.14 721.07 723.09
723.88 726.76 755.60 761.10 765.73 768.26
769.84 770.30 771.46 783.26 795.59 849.66
856.98 867.72 870.18 871.05 878.07 878.87
932.91 946.27 947.24 948.06 950.83 957.35
958.82 962.31 971.62 978.95 982.29 990.66
992.27 994.65 998.93 1000.03 1008.69 1011.80
1012.93 1013.18 1013.34 1014.30 1015.03 1016.59
1017.01 1019.70 1020.76 1022.22 1023.10 1037.84
1042.01 1051.31 1051.89 1051.99 1053.02 1053.76
1054.06 1067.43 1081.62 1102.81 1106.06 1109.79
1110.14 1114.67 1114.97 1116.10 1117.18 1118.60
1120.58 1121.46 1125.16 1126.17 1128.69 1133.19
1163.97 1174.26 1178.05 1185.73 1201.04 1201.13
1202.19 1203.23 1203.64 1204.46 1219.00 1222.27
1226.08 1226.87 1228.71 1229.73 1235.47 1236.41
1262.58 1276.02 1292.79 1318.25 1319.78 1322.18
1322.98 1326.19 1332.71 1335.68 1348.98 1362.63
1365.82 1366.45 1367.33 1370.33 1370.74 1373.57
1410.66 1415.74 1467.33 1472.03 1472.50 1474.62
1475.55 1479.40 1479.94 1480.00 1490.58 1494.16
1503.87 1506.25 1507.03 1524.28 1525.14 1525.77
1526.37 1527.04 1529.97 1530.54 1591.40 1623.20
1623.71 1624.66 1625.96 1627.95 1628.30 1641.92
1642.12 1642.41 1643.13 1644.21 1644.65 1645.54
1790.29 2986.14 3024.62 3048.63 3050.97 3057.01
3063.95 3108.71 3109.69 3126.15 3143.76 3151.14
3168.23 3178.44 3181.47 3182.87 3186.69 3187.82
3189.69 3190.34 3191.17 3191.37 3192.74 3193.63
3196.24 3198.38 3198.60 3199.43 3200.00 3203.33
3206.22 3207.93 3211.02 3212.02 3212.75 3213.51
3214.34 3215.51 3223.08 3224.81 3230.50 3231.76
3238.60 3241.28 3242.69

==============================

11A

==============================

5.95 10.10 19.17 20.66 24.41 27.05
31.48 37.02 42.10 42.81 46.57 52.35
54.32 60.12 61.31 66.13 69.06 70.38
76.23 81.26 83.97 87.38 94.13 101.49
106.50 125.11 142.49 152.42 155.72 172.75
190.24 195.37 197.68 203.81 205.54 216.83
223.94 227.41 236.52 242.17 247.53 249.97
254.46 257.50 261.66 268.35 270.17 272.83
276.48 289.12 315.14 325.53 353.60 376.41
406.68 407.43 408.72 410.38 415.13 416.06
418.27 422.55 431.13 444.57 457.87 464.30
467.76 501.28 508.61 511.67 512.99 517.81
532.33 537.94 570.63 620.26 628.85 629.51
629.77 630.12 630.52 631.71 632.55 653.00
685.56 690.56 694.60 696.27 709.23 709.77
709.98 710.83 711.50 712.29 719.50 721.19
724.35 726.70 762.40 763.16 764.98 766.37
768.09 770.37 774.40 792.50 813.29 836.95
864.63 867.01 868.76 871.80 874.03 875.21
909.11 940.10 945.92 946.96 948.00 953.05
955.04 960.66 969.19 986.88 987.38 988.02
989.46 991.74 993.92 994.46 997.05 1012.20
1012.82 1012.94 1013.42 1014.26 1015.01 1017.38
1017.97 1018.30 1018.77 1019.45 1020.91 1026.46
1042.19 1050.65 1051.00 1051.81 1052.39 1052.92
1053.58 1060.82 1087.65 1094.17 1111.86 1113.21
1113.30 1114.59 1114.97 1115.74 1116.97 1118.56
1119.53 1119.64 1120.00 1120.35 1123.35 1128.36
1164.21 1178.83 1201.09 1202.04 1202.26 1202.36
1203.34 1203.42 1208.08 1214.84 1223.51 1223.82
1224.45 1226.99 1228.61 1228.86 1232.00 1242.76
1261.74 1283.93 1291.05 1322.98 1325.10 1328.89
1329.89 1332.63 1336.34 1347.79 1355.89 1363.51
1363.88 1367.47 1368.06 1370.00 1370.13 1391.59
1405.47 1408.37 1452.70 1469.12 1472.65 1475.06
1476.26 1476.73 1478.62 1479.83 1481.01 1494.45
1497.05 1507.76 1511.94 1523.58 1524.62 1525.71
1525.79 1526.83 1527.56 1530.29 1622.74 1624.46
1625.04 1626.51 1627.09 1628.05 1637.79 1641.38
1642.37 1642.86 1643.26 1643.45 1644.24 1708.75
1771.07 2962.01 2968.01 3020.22 3021.82 3062.44
3079.27 3080.14 3095.81 3102.05 3119.32 3129.16
3134.71 3138.11 3141.36 3156.91 3174.88 3184.11
3186.80 3188.50 3189.09 3189.69 3189.88 3191.63
3192.62 3196.23 3196.58 3198.61 3198.98 3199.21
3201.82 3204.37 3204.69 3206.09 3206.89 3209.60
3210.14 3212.56 3213.51 3214.09 3214.36 3215.03
3216.81 3217.66 3247.07

==============================

11B

==============================

4.67 13.17 20.32 24.87 26.22 34.73
38.23 44.35 47.67 50.01 53.96 57.31
61.10 63.23 67.78 72.53 75.74 80.17
83.44 88.01 91.36 96.82 99.47 106.18

122.71 135.54 142.03 151.45 159.07 171.58
175.24 191.25 198.57 201.10 204.17 213.95
220.98 224.80 227.98 236.46 242.90 246.17
247.60 261.20 266.03 272.15 274.18 282.81
290.99 304.06 327.58 338.94 348.95 394.95
400.66 407.76 410.83 412.59 414.15 417.65
419.98 420.79 428.27 451.37 458.26 463.10
467.86 501.45 508.78 510.04 512.92 520.74
532.71 540.55 566.16 588.57 626.32 626.61
627.35 628.81 630.19 630.57 630.99 658.27
693.62 694.69 695.96 708.59 709.36 710.14
711.69 713.56 714.64 717.44 720.52 721.48
722.19 762.40 763.33 765.06 765.75 766.34
769.85 770.98 781.01 784.82 813.53 863.48
864.74 866.48 868.47 869.39 876.54 879.50
903.16 942.87 944.49 945.41 947.29 947.72
954.98 960.10 967.70 976.60 984.79 987.28
988.06 988.78 995.12 1002.06 1009.68 1012.47
1012.70 1013.18 1013.61 1013.97 1015.05 1015.88
1016.14 1016.79 1018.13 1019.31 1022.07 1028.65
1037.10 1045.98 1051.17 1052.03 1052.19 1052.87
1053.20 1060.99 1090.85 1094.85 1101.52 1111.13
1113.95 1114.71 1115.15 1115.46 1117.24 1118.06
1118.28 1118.93 1119.87 1120.35 1121.45 1132.75
1176.69 1200.94 1201.40 1201.80 1202.11 1202.27
1202.36 1204.80 1207.32 1213.54 1222.47 1223.05
1225.78 1226.75 1227.85 1229.24 1231.74 1255.17
1266.40 1289.31 1324.35 1324.69 1329.18 1331.66
1333.60 1334.19 1334.95 1349.18 1357.28 1364.28
1367.35 1367.80 1368.67 1370.03 1372.85 1395.72
1409.87 1412.64 1472.54 1472.94 1475.71 1477.07
1478.45 1478.83 1481.30 1483.54 1494.29 1496.28
1501.28 1505.39 1509.20 1524.28 1526.32 1526.63
1527.35 1527.47 1528.23 1528.46 1622.69 1624.15
1625.37 1626.52 1627.88 1628.41 1630.67 1641.77
1642.61 1644.35 1644.55 1644.69 1645.39 1680.19
1715.72 2946.50 2953.68 3013.07 3027.94 3034.53
3040.54 3071.86 3075.86 3079.15 3088.27 3110.06
3160.29 3168.50 3184.03 3185.24 3185.57 3186.29
3187.26 3188.09 3190.10 3192.45 3192.55 3194.47
3194.57 3194.93 3196.42 3200.44 3201.19 3205.32
3206.06 3207.61 3208.08 3208.33 3209.95 3211.02
3212.78 3213.51 3214.17 3214.91 3215.86 3216.25
3217.53 3218.43 3227.38

==============================

9A-TS

==============================

-307.08 11.88 17.97 28.69 36.22 39.63
48.58 50.23 53.13 56.58 61.70 62.73
63.47 70.84 74.12 77.75 81.92 83.69
88.81 95.17 96.90 105.53 106.50 112.74
121.22 126.99 131.41 139.67 146.66 159.64
164.39 169.27 193.00 201.02 209.80 216.88
217.69 222.93 227.13 234.76 237.03 240.23
257.63 259.12 260.99 262.94 270.87 276.48
280.66 293.76 303.42 337.18 358.92 401.31
408.13 409.89 412.71 419.88 423.17 427.04
430.30 432.92 440.31 444.89 456.78 467.08
469.90 475.98 490.22 503.64 508.52 518.02
527.35 532.71 538.67 547.08 560.63 627.59
627.82 629.15 629.84 630.85 632.28 633.20
693.60 698.02 701.59 705.31 707.24 707.82
709.60 712.44 714.15 716.50 718.12 720.92
723.72 725.15 748.50 761.33 763.67 766.79
769.03 770.40 772.96 803.17 813.82 864.80
867.92 871.43 873.96 880.67 886.92 890.08
920.28 942.80 943.95 947.37 950.15 953.74
956.24 962.40 970.20 985.44 985.85 991.23
994.00 996.05 1001.42 1005.66 1007.23 1010.17
1012.26 1012.78 1013.56 1014.05 1014.66 1015.47
1016.41 1016.78 1018.81 1019.18 1020.80 1026.03
1037.28 1050.29 1050.85 1053.11 1053.25 1053.71
1054.63 1055.02 1075.96 1084.60 1107.62 1108.99
1109.78 1110.94 1111.55 1116.54 1117.21 1117.64
1119.00 1119.34 1122.07 1123.09 1124.95 1133.94
1155.25 1176.58 1179.98 1199.84 1200.65 1201.18
1202.95 1203.35 1203.79 1217.30 1219.42 1220.95
1222.33 1228.17 1231.42 1233.11 1233.91 1246.52
1259.09 1293.16 1300.44 1320.58 1321.58 1323.78
1328.01 1328.79 1335.28 1336.74 1341.05 1360.37
1363.74 1366.47 1367.57 1370.22 1370.79 1375.05
1409.00 1423.60 1473.29 1473.55 1474.56 1475.37
1475.57 1480.10 1480.59 1481.01 1489.88 1492.55
1495.27 1501.30 1502.98 1522.72 1523.84 1524.81
1527.07 1528.11 1529.48 1531.19 1569.03 1624.64
1625.84 1626.94 1627.09 1629.61 1630.29 1639.35
1642.88 1643.62 1644.26 1644.87 1646.87 1647.30
1769.83 2969.72 3032.36 3035.16 3045.70 3076.49
3091.67 3092.27 3101.51 3126.38 3150.90 3166.18
3169.10 3184.93 3185.36 3185.54 3185.78 3185.98
3187.63 3188.02 3192.76 3192.84 3194.13 3194.44
3194.51 3194.83 3200.01 3201.12 3201.49 3202.25
3202.86 3203.25 3204.53 3207.55 3210.14 3211.31
3211.74 3212.92 3213.78 3223.02 3226.67 3227.79
3228.01 3229.64 3233.76

==============================

9B-TS

==============================

-301.01 2.49 22.90 28.27 34.18 45.69
47.70 49.42 53.31 57.79 60.24 63.50
67.41 71.00 72.39 76.26 79.04 79.60
85.10 93.65 96.16 105.30 112.54 115.91
119.10 129.15 130.58 135.76 141.44 162.51
165.77 181.61 195.97 199.78 213.77 216.44
221.23 224.49 229.54 237.78 239.32 257.35
258.56 261.84 262.93 270.69 274.25 280.48
287.87 311.27 316.70 327.51 368.21 384.14
407.75 411.01 412.07 419.42 423.49 427.71
429.96 431.71 432.59 440.75 447.14 461.16
471.99 472.40 486.36 499.13 505.24 519.40
528.17 534.84 544.26 562.32 590.77 616.08
627.26 627.34 628.95 630.12 631.50 632.66
676.09 693.75 697.63 705.98 707.90 710.17
710.70 712.36 714.99 715.79 716.89 720.35
722.92 723.72 736.91 759.60 762.10 766.82
768.96 771.50 773.76 781.08 804.44 845.58
862.25 865.12 870.44 870.81 880.24 885.32
930.29 940.64 944.40 949.14 951.06 955.42
960.33 966.47 974.82 985.32 990.22 990.71
991.24 993.80 998.91 1000.77 1004.52 1011.03
1012.02 1012.65 1013.90 1014.20 1015.05 1015.50
1017.72 1018.34 1019.30 1021.20 1026.30 1026.72

1030.69 1048.58 1049.73 1050.39 1050.72 1053.63
1054.15 1069.61 1085.54 1095.60 1107.15 1107.83
1108.78 1113.80 1114.66 1116.32 1116.87 1117.79
1118.60 1119.29 1120.56 1121.71 1123.89 1138.14
1161.34 1178.16 1180.19 1185.51 1200.09 1200.31
1201.77 1201.96 1203.77 1203.90 1218.75 1220.10
1221.66 1226.90 1227.24 1228.86 1230.88 1234.69
1257.33 1284.18 1292.67 1318.65 1320.58 1322.94
1326.57 1329.67 1334.91 1335.69 1341.08 1359.49
1362.61 1366.82 1367.34 1368.29 1370.07 1371.29
1410.62 1418.60 1460.33 1467.89 1473.02 1473.99
1474.17 1476.41 1479.52 1479.73 1480.80 1483.34
1495.88 1501.38 1503.91 1522.35 1524.10 1524.67
1526.42 1526.91 1527.32 1529.86 1550.93 1617.19
1623.81 1624.58 1626.64 1626.99 1629.20 1630.09
1641.79 1642.25 1644.24 1644.94 1646.27 1646.98
1771.68 2965.98 3041.29 3042.51 3056.71 3063.90
3098.55 3107.95 3116.97 3124.71 3145.55 3161.05
3177.68 3178.35 3181.36 3184.89 3185.52 3186.08
3187.34 3187.65 3187.73 3191.79 3194.25 3194.31
3194.64 3195.04 3196.86 3200.72 3202.62 3203.07
3207.03 3207.56 3207.80 3211.09 3211.15 3213.61
3214.12 3214.40 3217.43 3218.61 3227.36 3228.17
3230.21 3239.84 3251.86

==============================

11A-TS

==============================

-139.15 11.07 17.48 27.26 32.95 37.53
42.12 48.08 51.22 56.39 58.09 61.90
63.23 66.97 69.91 77.75 79.05 81.62
90.37 99.02 102.24 104.40 109.48 111.74
118.53 122.55 127.95 134.88 148.27 159.03
165.51 177.39 179.12 188.93 196.08 206.90
217.21 220.89 224.79 230.66 241.55 242.64
248.66 254.46 258.57 263.35 265.16 279.64
280.81 283.63 302.09 325.65 356.51 383.07
404.83 405.75 413.42 417.43 421.82 424.13
429.39 431.41 434.50 439.03 445.62 462.88
470.41 488.19 503.22 504.23 520.20 522.85
531.40 537.72 561.55 617.67 624.49 628.78
630.69 630.74 631.59 632.01 632.76 638.09
682.51 696.99 698.12 699.95 705.70 708.72
712.42 712.84 714.45 715.21 715.90 720.99
722.67 728.82 760.19 762.35 767.14 767.93
770.54 771.25 772.20 783.30 820.91 836.87
862.32 864.20 868.84 872.34 877.29 887.67
908.92 937.14 938.26 942.42 946.93 951.05
960.48 963.01 969.63 971.13 983.10 986.15
990.18 992.28 995.67 999.31 1008.48 1012.22
1013.67 1014.05 1014.08 1014.73 1015.26 1015.99
1016.18 1016.95 1017.59 1020.11 1022.55 1025.81
1050.51 1050.82 1052.61 1053.03 1053.53 1054.25
1055.68 1081.03 1096.52 1102.76 1111.52 1112.06
1113.18 1113.78 1115.30 1116.95 1118.43 1119.13
1121.51 1121.67 1122.33 1123.51 1125.35 1128.24
1140.05 1181.73 1200.81 1201.35 1202.14 1202.23
1202.95 1203.08 1204.08 1217.06 1223.08 1226.36
1226.82 1227.41 1229.44 1233.71 1234.75 1244.48
1265.70 1290.76 1300.63 1320.16 1324.23 1330.79
1331.03 1335.35 1335.59 1336.35 1345.27 1357.59
1362.07 1364.63 1366.45 1366.88 1367.73 1368.60
1378.19 1405.85 1445.88 1459.47 1472.69 1475.00
1476.58 1477.54 1478.05 1480.64 1481.68 1482.69
1495.34 1501.95 1504.67 1505.08 1524.76 1526.19
1527.13 1527.32 1527.74 1528.89 1531.47 1624.58
1626.89 1627.35 1628.21 1629.95 1630.10 1642.68
1644.03 1644.69 1644.83 1645.77 1647.60 1677.03
1713.65 2964.19 2970.16 3016.19 3023.85 3030.59
3070.37 3090.67 3103.20 3120.98 3123.85 3125.03
3157.77 3177.80 3182.39 3184.49 3185.09 3185.68
3185.97 3186.57 3187.69 3188.17 3191.74 3193.90
3194.31 3195.05 3195.50 3197.42 3200.41 3202.94
3203.23 3204.64 3205.90 3207.22 3209.87 3211.02
3211.45 3211.82 3213.09 3217.33 3218.52 3218.72
3220.28 3225.08 3228.60

==============================

11B-TS

==============================

-235.68 13.53 17.54 20.66 26.07 30.10
42.16 45.28 47.98 51.36 54.05 55.70
58.25 62.32 62.89 68.39 69.95 71.91
82.82 86.66 89.96 92.51 100.35 105.62
110.15 113.99 122.86 124.03 125.21 146.99
150.41 164.88 175.24 201.29 205.26 207.27
217.09 226.18 234.32 235.97 240.92 248.43
250.99 262.09 263.35 264.87 270.17 275.18
277.11 280.53 299.14 318.64 327.29 403.32
408.61 409.94 413.99 415.24 418.68 421.31
423.32 426.57 434.23 451.13 454.07 462.08
468.69 493.62 500.54 508.06 514.55 523.64
534.69 545.13 553.78 580.28 620.60 627.75
628.09 628.83 630.39 631.08 633.10 662.26
687.96 694.38 699.36 708.03 709.56 711.42
712.37 714.01 714.99 715.46 720.60 721.98
724.96 729.00 761.52 764.29 767.47 768.27
772.12 772.90 773.83 788.87 855.91 862.29
868.10 874.24 875.72 877.08 879.29 882.61
911.08 928.15 939.23 944.55 946.56 953.79
955.42 959.00 961.72 968.45 985.08 989.97
995.83 996.84 1000.90 1001.90 1011.33 1011.56
1011.97 1012.92 1014.81 1015.31 1015.89 1016.32
1017.47 1018.30 1018.67 1018.86 1021.56 1024.45
1045.68 1050.25 1051.07 1051.94 1052.51 1053.84
1055.80 1057.24 1074.13 1080.91 1103.61 1111.01
1112.40 1112.57 1114.02 1116.32 1117.50 1117.80
1118.96 1119.45 1120.48 1122.54 1123.69 1124.55
1175.05 1186.49 1199.25 1200.29 1200.63 1201.65
1202.22 1202.93 1205.05 1210.82 1220.40 1222.54
1223.97 1226.72 1228.48 1229.72 1235.78 1235.85
1259.57 1271.28 1285.67 1323.13 1326.22 1327.85
1328.37 1329.40 1335.28 1337.26 1338.47 1362.86
1363.20 1365.99 1366.97 1371.45 1378.37 1381.32
1396.30 1425.28 1471.62 1474.04 1474.37 1475.37
1475.87 1478.26 1480.07 1481.06 1486.82 1495.31
1500.86 1503.88 1507.71 1522.02 1522.56 1523.92
1524.36 1525.56 1527.46 1531.52 1623.64 1624.42
1625.07 1626.55 1628.89 1630.39 1634.74 1640.71
1641.52 1642.99 1644.89 1645.64 1647.10 1669.66
1771.27 2931.23 2973.20 2990.61 3007.87 3033.36
3049.83 3072.07 3085.73 3116.10 3124.91 3157.49
3158.19 3174.11 3183.20 3185.17 3185.70 3185.83
3186.93 3187.50 3188.05 3193.07 3193.61 3194.13

3195.05 3196.36 3196.97 3202.45 3204.94 3205.68
3206.03 3206.25 3206.41 3211.30 3211.95 3212.61
3212.88 3213.21 3213.29 3214.21 3223.73 3227.53
3228.19 3230.00 3232.33
==============================
10
==============================
9.44 26.47 27.45 37.11 40.95 43.63
49.56 53.44 55.97 56.95 60.66 63.90
68.36 70.81 76.84 77.46 83.78 89.25
97.51 102.94 106.49 108.97 111.64 120.78
126.95 143.16 146.71 154.36 163.61 170.50
178.53 189.20 197.25 203.43 207.16 210.23
220.19 228.11 239.48 242.35 247.13 253.38
257.31 262.34 266.15 271.53 280.51 288.51
314.43 327.56 354.31 367.87 408.16 411.17
413.52 415.08 416.93 419.24 422.47 425.07
433.23 441.14 445.25 451.64 459.17 465.44
477.91 495.24 505.18 513.69 517.44 521.16
534.96 540.22 552.44 606.44 626.77 629.04
629.53 630.82 631.59 633.07 633.37 662.16
693.58 698.37 708.91 709.12 711.09 713.09
715.68 716.55 717.19 717.91 718.54 721.77
722.47 762.27 762.98 766.94 769.00 770.46
773.98 790.24 802.19 812.26 863.96 867.41
874.19 877.18 878.95 879.98 887.59 921.33
933.35 943.16 946.52 954.00 954.13 955.03
957.55 958.04 979.55 986.74 990.30 995.78
997.08 999.74 999.84 1003.38 1012.57 1013.39
1014.09 1014.37 1014.96 1016.26 1017.51 1017.56
1018.04 1019.09 1019.81 1020.92 1032.26 1050.94
1051.02 1051.95 1052.95 1053.56 1054.25 1055.55
1073.65 1079.14 1108.61 1111.68 1112.65 1114.30
1114.91 1116.02 1116.07 1117.24 1117.48 1118.50
1119.56 1120.33 1120.79 1123.31 1129.63 1176.16
1176.51 1196.27 1198.59 1200.43 1202.48 1202.81
1202.88 1203.59 1216.27 1223.22 1224.50 1226.34
1227.10 1228.36 1230.60 1232.62 1236.92 1256.48
1294.55 1305.38 1320.35 1323.56 1325.30 1327.63
1328.83 1329.87 1332.68 1335.57 1346.81 1361.05
1363.86 1365.98 1367.49 1371.45 1372.86 1397.86
1414.07 1421.10 1423.09 1472.66 1473.79 1475.60
1476.18 1479.30 1479.82 1481.63 1484.40 1493.83
1495.03 1504.08 1505.95 1510.63 1519.20 1524.64
1525.72 1526.48 1526.74 1528.97 1530.11 1540.01
1624.34 1625.33 1626.90 1627.76 1628.73 1629.72
1641.30 1642.74 1643.92 1645.09 1645.84 1646.71
1756.66 2987.34 3005.37 3041.67 3056.08 3062.93
3069.60 3107.42 3116.15 3120.40 3127.40 3149.94
3156.42 3178.29 3183.52 3185.46 3186.09 3186.43
3186.54 3186.57 3188.29 3192.31 3193.97 3194.15
3196.24 3196.42 3198.41 3200.63 3202.68 3202.96
3205.63 3207.69 3209.73 3210.04 3212.22 3213.02
3213.33 3213.71 3218.62 3222.21 3223.11 3236.72
3244.23 3246.09 3263.31
==============================
12
==============================
4.74 21.73 25.58 36.33 40.20 48.35
50.05 52.85 54.94 58.08 60.14 63.20
66.25 73.97 77.21 83.46 84.92 88.73
93.87 100.67 106.16 109.84 115.15 118.25
125.29 138.54 139.68 148.28 152.75 163.26
167.33 176.67 196.08 200.33 206.44 212.28
221.36 229.99 232.36 238.12 242.91 253.12
259.31 262.05 266.49 280.45 282.00 288.82
292.54 307.81 324.58 353.49 381.00 408.28
412.38 416.73 421.31 425.36 427.37 428.35
432.69 435.22 436.85 443.41 464.67 469.05
474.23 487.63 502.70 508.65 517.59 522.64
531.93 540.79 571.68 588.04 628.25 629.09
629.64 630.75 632.32 632.70 633.29 687.54
694.17 696.70 705.99 708.62 710.81 712.81
715.78 717.75 718.74 720.83 723.93 729.49
732.85 760.85 765.27 767.41 771.01 772.06
772.42 779.11 789.50 824.96 865.55 870.37
874.07 880.43 882.99 886.25 896.21 911.46
926.05 942.14 946.36 950.14 953.53 958.58
964.06 966.03 985.23 988.15 992.96 993.90
999.06 1003.64 1005.01 1006.55 1012.27 1012.90
1013.12 1013.35 1014.42 1014.51 1016.12 1016.53
1018.42 1020.42 1021.58 1028.12 1029.56 1044.04
1047.53 1050.91 1052.67 1053.05 1053.25 1054.19
1077.13 1090.84 1107.48 1109.36 1110.57 1112.67
1114.32 1115.48 1115.86 1116.98 1118.10 1118.56
1120.91 1121.55 1122.88 1124.93 1164.50 1177.45
1183.84 1199.39 1199.79 1200.79 1201.12 1201.34
1202.96 1209.35 1215.69 1222.62 1224.28 1225.65
1227.63 1231.70 1234.55 1239.17 1241.68 1248.48
1281.82 1308.75 1315.75 1323.81 1324.25 1327.81
1329.86 1332.60 1337.54 1345.11 1361.76 1365.12
1365.59 1367.94 1370.56 1371.30 1372.68 1380.26
1412.79 1428.19 1469.92 1472.98 1473.87 1476.39
1477.25 1479.43 1479.79 1482.36 1492.87 1494.21
1497.26 1502.71 1509.49 1523.34 1525.62 1526.26
1526.61 1527.05 1528.43 1531.34 1539.50 1572.66
1622.26 1624.07 1625.53 1626.24 1626.85 1629.30
1642.08 1642.50 1643.25 1643.51 1644.13 1644.46
1791.54 2992.26 3031.41 3038.79 3044.71 3052.01
3066.52 3088.79 3108.76 3112.83 3126.86 3150.14
3168.88 3178.22 3184.30 3185.24 3185.83 3186.09
3187.86 3188.53 3189.83 3191.99 3194.17 3196.33
3196.43 3197.07 3198.52 3200.60 3206.74 3207.51
3208.14 3209.97 3209.99 3211.20 3213.26 3214.09
3215.41 3216.70 3218.49 3220.72 3229.04 3231.21
3235.40 3255.65 3261.75
==============================
13
==============================
12.25 21.33 23.69 27.35 32.71 35.72
38.25 40.88 44.58 47.47 49.57 50.20
52.58 53.41 55.03 58.82 59.54 66.54
70.10 72.70 76.33 80.23 82.86 87.92
90.96 96.05 104.55 106.99 116.56 138.46
142.01 144.91 146.63 161.84 163.17 165.90
172.19 175.36 179.68 181.71 189.02 192.63
194.47 199.37 205.38 208.81 214.40 218.47
224.08 228.77 230.96 237.83 244.19 245.75
256.60 263.40 265.55 280.67 283.02 286.93
296.56 297.63 299.70 316.78 325.24 330.85
343.68 353.90 381.28 397.80 403.62 415.31
418.16 419.86 425.88 430.03 448.12 453.31

461.00 464.26 469.38 476.05 487.75 497.33
502.44 514.24 517.99 519.77 520.90 524.23
525.58 528.53 531.03 531.96 533.43 536.47
542.87 544.98 547.96 552.98 555.63 559.82
562.45 567.38 573.12 576.42 596.95 607.89
616.17 635.83 654.67 678.12 682.02 689.87
702.52 711.25 713.45 714.14 716.79 718.28
729.08 765.58 768.70 790.26 806.25 814.38
835.07 838.91 850.20 853.16 861.43 867.38
869.96 870.91 873.96 876.84 883.54 884.41
888.11 889.84 894.27 899.40 903.72 916.20
920.63 925.77 931.83 934.90 937.14 940.37
943.28 945.46 957.12 958.88 962.83 964.16
966.22 966.92 967.55 970.21 973.26 987.36
990.59 1003.79 1007.23 1010.14 1011.29 1011.93
1014.11 1014.52 1022.15 1022.60 1024.48 1025.35
1035.02 1046.02 1046.54 1047.61 1048.94 1057.88
1059.58 1063.25 1065.90 1066.30 1067.09 1067.28
1067.34 1068.42 1069.12 1075.64 1099.22 1140.47
1155.11 1160.60 1161.01 1163.93 1166.96 1174.61
1189.40 1190.14 1201.58 1203.10 1203.60 1204.31
1207.28 1208.71 1216.90 1242.40 1249.57 1259.45
1262.57 1283.07 1287.35 1289.13 1290.44 1294.94
1300.64 1306.40 1312.03 1318.82 1319.10 1321.00
1329.46 1339.18 1344.59 1357.31 1359.81 1360.39
1366.25 1367.74 1390.26 1391.19 1410.01 1411.19
1425.20 1426.35 1427.72 1429.02 1429.54 1431.13
1431.42 1431.66 1432.93 1453.55 1454.90 1457.85
1459.37 1462.36 1463.74 1464.32 1464.77 1466.25
1466.74 1467.47 1491.97 1497.01 1497.66 1497.95
1498.48 1498.87 1500.54 1501.17 1501.24 1501.99
1502.08 1502.23 1509.83 1510.39 1511.88 1512.83
1518.57 1520.64 1521.11 1522.21 1551.84 1552.54
1601.96 1606.89 1607.49 1634.02 1634.91 1636.42
1636.73 1641.44 1643.01 1648.06 1649.06 1650.39
1651.98 1673.92 1674.37 2058.08 2993.45 3035.64
3036.16 3037.17 3037.42 3038.50 3041.26 3041.31
3043.70 3046.85 3088.70 3089.12 3097.00 3097.73
3099.39 3099.56 3100.80 3100.95 3101.99 3111.45
3121.20 3125.48 3127.06 3128.57 3129.50 3129.50
3131.56 3144.09 3151.19 3152.36 3156.60 3166.33
3169.91 3170.16 3173.24 3176.27 3177.73 3178.28
3181.58 3182.42 3184.13 3186.29 3188.09 3192.61
3193.43 3204.30 3205.17 3206.58 3217.87 3217.89
3224.38 3228.59 3243.57 3243.82 3243.91 3398.26

==============================

13-TS

==============================

-299.10 9.31 16.92 22.63 26.30 29.60
32.95 36.22 41.48 43.38 46.42 47.88
49.13 52.34 56.14 59.03 61.05 61.96
64.38 64.73 68.79 69.66 75.04 75.91
81.43 90.52 100.13 102.07 106.97 111.69
114.91 142.38 146.92 151.92 156.68 166.78
170.06 174.28 176.17 185.17 188.90 189.92
196.32 197.76 201.65 209.49 220.12 222.40
223.01 232.62 234.31 237.66 243.31 248.31
253.07 253.78 280.81 283.54 287.68 294.79
297.01 307.70 321.84 325.55 326.48 342.71
357.10 402.79 405.64 414.96 421.98 429.24
434.99 443.51 452.40 460.52 464.05 468.90

483.85 487.56 494.26 496.03 499.67 502.05
515.22 517.18 517.95 521.74 522.91 523.98
526.83 528.35 531.27 532.08 534.87 538.52
542.74 543.73 548.14 552.29 554.02 558.13
563.70 570.07 573.16 593.64 599.59 617.84
629.97 654.74 660.88 681.25 686.54 703.76
703.95 709.72 710.40 711.30 754.35 766.71
767.07 784.81 785.79 803.35 806.12 838.95
839.93 845.77 849.82 857.48 864.08 867.99
870.85 872.60 876.59 877.52 880.54 882.21
889.92 892.38 899.82 907.33 910.00 918.50
918.80 931.49 936.34 938.62 942.03 942.50
951.88 955.83 962.01 962.84 965.15 970.21
971.51 975.05 980.72 986.68 987.25 987.80
994.25 998.04 1005.33 1005.48 1006.00 1007.17
1009.59 1019.02 1022.74 1022.92 1023.54 1024.02
1044.67 1045.44 1046.22 1048.78 1055.90 1058.04
1064.44 1064.67 1065.11 1065.58 1066.00 1066.36
1066.83 1067.39 1074.95 1083.14 1128.63 1140.34
1149.86 1158.84 1159.86 1162.59 1165.37 1167.59
1175.65 1188.59 1192.78 1200.22 1203.79 1203.97
1206.65 1207.30 1208.20 1213.23 1240.15 1243.02
1257.24 1257.44 1277.62 1285.21 1288.90 1291.00
1294.64 1301.52 1307.17 1319.07 1320.16 1323.12
1325.05 1333.52 1347.06 1352.42 1360.44 1361.21
1362.23 1362.62 1364.53 1386.37 1407.70 1409.54
1412.07 1422.84 1424.36 1425.48 1426.85 1428.69
1428.72 1430.75 1430.97 1431.12 1454.82 1454.98
1460.32 1461.63 1463.41 1463.67 1464.68 1465.05
1465.75 1465.99 1483.56 1492.28 1496.24 1497.17
1498.31 1499.40 1499.75 1500.32 1501.37 1501.64
1501.99 1502.48 1507.62 1508.99 1510.22 1510.57
1511.28 1516.08 1517.54 1518.96 1520.44 1552.59
1553.32 1602.84 1603.09 1633.08 1633.68 1635.26
1635.95 1638.12 1640.01 1647.69 1649.12 1649.62
1651.81 1674.16 1675.30 1746.54 2976.80 2981.69
3038.06 3040.72 3041.06 3041.10 3041.35 3042.12
3043.69 3043.77 3100.62 3102.51 3102.70 3102.94
3103.41 3104.73 3104.94 3106.42 3107.22 3111.55
3115.22 3123.00 3126.23 3128.80 3128.98 3129.28
3130.28 3130.32 3131.70 3150.31 3166.04 3167.18
3168.60 3169.55 3170.63 3171.79 3175.04 3175.34
3178.63 3181.40 3182.18 3185.35 3189.19 3190.44
3193.89 3199.81 3201.41 3205.67 3205.69 3206.84
3215.80 3225.18 3225.44 3225.88 3230.66 3272.48

==============================

14

==============================

12.94 16.98 18.72 22.94 23.62 28.47
31.82 34.04 34.76 36.35 39.42 43.20
43.25 44.21 47.72 50.65 60.34 64.77
66.66 67.70 69.33 73.20 78.81 80.82
86.94 88.33 94.93 96.65 108.91 123.34
139.99 144.69 149.28 158.35 162.67 167.25
175.12 176.12 188.51 189.80 191.97 193.67
195.81 198.81 212.77 217.14 221.87 222.93
227.65 229.08 233.86 236.89 243.00 251.07
252.65 258.60 280.32 284.57 286.92 294.12
295.37 312.64 318.22 323.60 342.42 347.10
397.39 400.98 413.33 416.62 428.78 448.02
450.03 454.91 469.82 474.23 475.41 484.39

492.90 498.03 515.55 518.78 519.67 520.48
521.25 524.00 524.98 527.26 529.90 532.59
534.74 540.91 543.58 546.21 547.57 553.02
554.54 557.72 562.81 567.98 570.13 574.25
584.10 598.18 615.60 620.89 635.10 636.59
661.49 673.60 681.72 689.64 701.00 706.91
708.79 710.25 713.67 753.78 767.99 769.03
790.72 801.54 804.53 814.93 834.71 837.55
842.58 854.01 856.16 864.34 865.28 866.85
870.18 871.28 875.23 877.09 878.05 878.75
891.70 894.80 903.02 910.00 912.37 914.46
930.68 932.41 937.40 939.06 945.95 957.54
963.69 964.41 967.15 969.15 973.60 977.27
980.20 989.70 991.60 1004.67 1006.94 1009.46
1009.87 1011.82 1012.56 1014.18 1015.38 1020.88
1022.36 1024.04 1026.15 1044.86 1046.49 1046.57
1047.00 1057.32 1059.27 1060.87 1064.37 1065.25
1065.75 1066.81 1066.99 1068.17 1068.80 1068.89
1072.20 1076.53 1110.17 1130.71 1149.62 1154.75
1157.18 1159.95 1160.74 1162.83 1166.09 1175.65
1189.31 1201.59 1202.41 1204.79 1206.65 1207.07
1208.99 1209.33 1215.62 1222.91 1241.87 1251.52
1257.69 1259.51 1277.52 1285.44 1286.35 1287.62
1291.51 1295.37 1300.87 1321.88 1323.81 1324.31
1327.35 1327.92 1337.19 1342.68 1343.91 1360.94
1363.50 1366.22 1367.43 1392.12 1392.58 1409.36
1411.17 1427.22 1427.99 1428.75 1429.10 1430.34
1430.37 1431.01 1431.41 1432.09 1456.75 1457.82
1458.91 1460.93 1462.45 1463.72 1464.65 1465.07
1465.28 1466.85 1469.30 1494.79 1495.10 1495.67
1496.54 1496.78 1498.03 1499.05 1499.75 1500.88
1504.53 1505.11 1509.81 1511.52 1511.54 1512.29
1520.28 1520.33 1520.62 1522.88 1526.88 1548.80
1551.89 1595.19 1606.56 1634.41 1634.81 1635.35
1636.99 1641.66 1643.21 1643.47 1649.76 1650.34
1651.32 1652.48 1672.68 1673.83 2967.10 2992.83
3001.92 3011.85 3039.48 3040.22 3040.58 3040.93
3041.86 3042.06 3043.44 3044.94 3059.68 3097.39
3098.67 3100.13 3101.15 3102.13 3102.58 3106.34
3106.39 3107.37 3117.87 3124.22 3127.06 3129.37
3130.30 3130.81 3131.72 3133.42 3135.60 3147.83
3149.93 3171.40 3172.03 3172.61 3173.03 3179.92
3184.04 3184.64 3186.85 3188.16 3188.88 3190.01
3193.18 3193.85 3197.75 3198.84 3208.64 3209.74
3213.94 3216.24 3218.79 3224.26 3230.11 3231.70

==============================

15A

==============================

10.44 17.54 23.17 25.13 29.63 33.29
34.57 37.66 38.77 41.53 45.80 46.23
49.86 51.73 53.84 56.90 59.33 61.66
63.92 69.42 73.28 75.21 82.37 85.53
86.29 92.90 96.53 102.39 103.94 107.88
112.12 117.80 122.30 131.80 133.96 143.39
150.25 153.61 155.51 159.01 162.89 166.39
172.30 179.51 182.93 184.84 188.61 193.21
196.23 199.82 202.42 207.54 211.21 221.90
223.95 229.59 233.57 235.26 242.06 247.08
249.45 251.11 259.96 264.91 270.45 274.91
276.82 282.25 288.29 290.83 292.66 302.84
309.54 314.30 327.08 335.06 341.79 345.25
389.35 391.90 400.68 412.75 418.87 427.62
444.21 446.85 453.11 458.16 465.15 486.15
488.48 491.99 497.69 511.62 516.13 518.00
519.56 520.71 523.42 524.91 526.92 528.19
530.50 533.71 534.98 536.87 540.00 542.37
545.91 550.89 554.42 556.94 559.63 561.34
564.40 566.62 571.73 574.65 580.67 598.65
619.27 620.07 631.11 633.99 658.07 677.34
685.23 690.56 703.11 705.85 711.65 713.79
719.48 753.59 761.99 768.76 771.47 788.00
803.70 813.58 828.60 830.12 839.23 840.52
842.49 854.57 860.29 861.70 864.66 865.05
871.92 873.90 875.27 877.50 881.07 888.33
891.29 894.92 900.43 907.32 909.30 930.20
931.15 932.44 934.42 939.27 948.62 958.28
963.21 965.79 966.50 969.01 970.09 971.48
974.24 984.52 988.03 991.55 1003.58 1003.79
1005.94 1012.09 1013.22 1016.32 1021.20 1022.50
1024.23 1024.27 1028.65 1030.51 1032.72 1044.37
1047.07 1052.49 1057.28 1058.25 1061.70 1064.64
1065.56 1066.46 1066.62 1069.52 1069.70 1070.87
1072.34 1073.67 1077.41 1088.82 1109.20 1118.73
1136.52 1138.35 1153.32 1156.81 1159.17 1164.43
1166.61 1167.56 1174.41 1174.72 1175.47 1188.97
1200.30 1203.57 1205.37 1205.56 1206.95 1208.81
1210.13 1211.63 1216.05 1229.09 1245.59 1250.88
1258.03 1260.77 1274.17 1284.18 1289.34 1290.42
1290.79 1295.19 1301.60 1302.41 1318.05 1321.40
1322.22 1326.35 1330.90 1348.68 1350.58 1351.03
1358.68 1361.38 1362.81 1363.31 1389.60 1395.04
1409.05 1409.86 1412.62 1427.49 1428.95 1429.88
1430.42 1430.57 1431.33 1433.59 1435.63 1451.63
1458.85 1461.24 1461.75 1464.44 1464.89 1466.15
1467.30 1467.76 1472.37 1473.96 1477.51 1478.78
1479.21 1485.66 1494.78 1496.75 1496.93 1497.43
1498.57 1499.28 1499.65 1501.76 1502.01 1502.73
1503.40 1507.91 1510.89 1513.26 1515.73 1517.04
1520.56 1520.72 1523.05 1528.07 1531.95 1536.89
1549.80 1551.36 1597.53 1603.52 1631.29 1634.16
1635.76 1636.67 1638.16 1639.86 1647.95 1650.23
1651.53 1651.88 1672.54 1674.97 1708.90 1786.28
2165.29 2725.55 2800.78 2848.39 2970.48 2994.63
3000.49 3001.42 3037.89 3038.73 3039.64 3041.05
3042.32 3046.69 3047.33 3054.40 3056.57 3066.38
3086.87 3089.29 3096.57 3099.63 3100.20 3100.84
3105.43 3108.32 3112.39 3121.30 3124.47 3125.45
3127.87 3128.73 3130.88 3130.91 3132.71 3148.03
3165.63 3169.58 3169.86 3169.88 3175.84 3178.75
3180.70 3180.87 3183.74 3186.79 3187.35 3188.90
3191.54 3192.06 3193.17 3194.51 3198.20 3199.41
3202.23 3206.81 3207.84 3217.60 3220.36 3225.58
3230.63 3234.05 3234.53

==============================

15B

==============================

12.87 15.30 25.09 28.07 32.27 33.90
37.49 39.12 40.49 41.89 47.29 48.26
49.46 53.35 53.86 55.78 56.31 66.34
69.44 72.05 73.00 75.01 80.23 91.27
94.93 100.95 104.62 107.85 109.27 111.96
113.97 118.24 120.02 122.65 132.61 138.55

140.45 144.90 149.13 151.46 155.26 162.24
164.26 168.43 169.66 177.40 181.57 185.29
190.48 191.12 196.31 197.92 199.68 209.80
217.12 218.73 222.79 227.42 233.46 234.81
239.86 240.54 249.37 252.57 264.37 269.96
277.58 282.08 286.81 289.12 290.56 294.80
300.68 303.87 316.88 322.16 336.19 344.22
346.79 364.49 391.94 401.98 410.73 416.50
433.56 441.47 448.22 452.36 459.49 466.72
484.78 486.39 490.64 493.81 515.08 515.34
516.00 518.27 521.16 522.87 523.93 525.71
530.51 531.08 533.61 536.45 537.40 538.81
543.26 545.42 547.60 553.10 556.24 559.87
565.11 566.61 568.66 573.99 581.43 598.65
617.30 618.75 627.86 638.95 659.13 677.82
680.95 685.91 701.15 708.37 711.89 712.65
716.04 750.58 767.60 768.72 775.03 787.18
803.86 810.74 826.52 830.00 836.47 841.35
849.42 851.12 853.28 859.94 861.28 863.68
870.31 873.27 875.36 877.51 878.83 890.57
891.31 892.54 894.77 912.57 916.33 930.71
931.69 931.91 937.93 944.65 951.95 957.10
961.91 966.82 968.39 969.88 971.85 972.38
974.85 989.71 990.60 994.65 1007.57 1008.75
1010.75 1012.47 1014.32 1015.86 1019.60 1023.95
1024.39 1024.68 1026.09 1027.74 1028.73 1041.21
1044.66 1047.44 1048.52 1055.79 1060.59 1061.24
1064.52 1065.42 1066.17 1066.57 1067.17 1068.26
1068.97 1069.09 1070.66 1075.36 1079.18 1089.27
1123.34 1128.78 1148.33 1157.67 1161.63 1162.75
1165.26 1169.66 1173.61 1174.05 1178.88 1187.73
1201.15 1202.46 1203.86 1205.07 1207.68 1209.12
1210.73 1214.77 1215.57 1229.86 1239.52 1249.61
1254.43 1259.55 1282.27 1283.84 1290.67 1292.47
1293.02 1296.14 1301.11 1302.43 1315.92 1318.31
1324.25 1325.63 1331.46 1335.02 1337.99 1353.03
1355.35 1361.14 1361.58 1371.58 1395.77 1397.74
1403.06 1408.42 1411.43 1425.22 1427.75 1428.81
1429.18 1430.28 1430.65 1431.22 1433.15 1434.95
1454.17 1456.50 1459.10 1460.93 1462.78 1463.99
1464.88 1465.58 1466.46 1470.65 1472.05 1481.12
1482.94 1492.30 1493.48 1495.01 1495.07 1496.04
1499.85 1500.15 1501.53 1501.70 1501.81 1503.74
1504.07 1508.46 1508.84 1510.98 1511.94 1513.87
1514.98 1519.50 1521.14 1521.71 1525.97 1529.14
1546.38 1552.09 1593.87 1610.40 1631.65 1635.25
1635.78 1637.05 1637.93 1646.37 1646.94 1649.35
1649.96 1651.60 1667.95 1672.88 1708.20 1777.77
2184.36 2970.81 2984.43 2999.58 3029.21 3036.83
3036.89 3038.17 3041.73 3041.80 3042.42 3043.32
3043.35 3050.93 3065.58 3088.28 3098.86 3100.51
3100.72 3102.10 3102.13 3104.72 3104.79 3112.67
3114.70 3118.52 3125.19 3127.13 3128.20 3128.79
3130.01 3131.13 3133.50 3133.65 3138.67 3140.76
3152.30 3167.61 3168.67 3170.48 3172.26 3174.05
3176.41 3181.26 3182.16 3183.06 3185.76 3186.17
3190.13 3193.00 3193.68 3194.92 3197.93 3208.37
3211.15 3213.63 3214.31 3215.92 3220.11 3220.60
3220.92 3231.37 3263.50

==============================

15A-TS1

==============================

-271.77 11.92 15.47 25.52 26.33 28.74
32.32 35.89 36.59 41.34 43.68 46.53
48.40 50.16 53.83 54.34 55.29 58.22
59.11 65.53 70.15 70.73 74.13 75.45
83.93 89.55 93.10 104.91 107.23 108.27
112.77 117.08 125.80 128.94 132.33 141.50
144.53 152.97 157.92 161.95 164.58 166.86
171.19 175.94 179.13 181.91 188.60 191.54
194.09 195.72 200.56 208.46 214.52 218.15
221.89 223.66 230.33 232.61 234.39 238.68
242.20 249.04 252.69 256.05 264.72 270.34
283.00 284.47 286.79 287.36 292.59 298.03
301.82 306.25 310.62 323.71 334.54 345.37
383.47 395.31 398.31 410.59 411.92 417.06
436.27 442.78 446.37 447.86 459.11 466.49
482.88 485.41 489.01 499.02 506.85 515.42
517.42 519.32 520.33 522.70 525.23 527.22
528.85 531.93 532.73 532.83 540.23 543.16
545.59 553.18 556.36 557.70 560.10 563.30
565.72 568.85 574.39 582.60 598.98 612.94
619.73 628.10 638.42 654.55 680.00 681.81
683.45 687.84 702.74 706.60 712.76 713.13
718.90 763.44 768.11 769.51 780.53 788.68
806.39 811.04 830.56 840.51 840.54 844.97
852.97 856.60 861.80 862.94 865.36 870.78
872.17 874.69 879.02 880.43 892.62 893.78
895.36 897.31 905.45 909.58 917.04 917.12
931.28 933.05 935.90 944.81 957.29 960.95
963.25 964.87 967.71 968.85 971.57 972.47
975.30 993.67 995.16 1004.52 1008.04 1008.93
1010.75 1012.06 1012.50 1012.67 1013.79 1023.92
1024.89 1025.56 1027.64 1032.00 1032.78 1042.13
1046.49 1048.13 1051.24 1053.54 1056.15 1058.71
1063.05 1064.07 1065.50 1066.19 1066.67 1067.47
1067.90 1068.66 1069.47 1069.55 1075.67 1093.78
1130.93 1144.86 1148.08 1159.18 1163.23 1163.42
1167.34 1173.67 1174.47 1177.20 1179.89 1188.47
1195.59 1201.80 1203.05 1204.61 1206.81 1207.78
1208.27 1214.20 1215.25 1218.15 1239.98 1248.74
1255.22 1257.72 1270.31 1288.41 1291.08 1291.21
1294.42 1296.28 1301.51 1304.37 1316.44 1319.32
1320.59 1325.82 1326.06 1336.21 1340.49 1350.04
1355.18 1359.29 1362.62 1370.82 1396.73 1397.66
1402.94 1404.58 1410.42 1426.07 1427.45 1428.81
1429.03 1429.97 1430.19 1430.91 1431.14 1434.43
1455.92 1458.11 1461.49 1461.98 1462.72 1464.10
1464.94 1465.58 1467.73 1470.22 1473.86 1480.02
1484.01 1493.88 1494.85 1496.02 1496.47 1497.63
1498.75 1499.64 1499.86 1502.58 1502.68 1504.46
1504.82 1508.02 1509.17 1510.58 1512.33 1513.45
1513.87 1519.62 1521.33 1524.09 1525.47 1533.02
1548.16 1552.23 1597.22 1611.00 1634.01 1635.34
1637.29 1637.40 1641.72 1647.11 1647.61 1649.46
1650.20 1651.08 1655.45 1670.79 1673.43 1775.98
1934.40 2957.59 2989.38 3006.35 3037.39 3038.66
3038.73 3040.52 3040.61 3040.73 3040.92 3041.39
3042.10 3043.27 3068.02 3098.74 3100.13 3101.15
3101.29 3101.72 3101.91 3103.45 3104.46 3110.78
3111.76 3114.39 3124.21 3126.13 3128.54 3130.32
3130.85 3131.05 3132.31 3133.23 3135.35 3154.17

3162.74 3164.07 3164.65 3165.65 3167.32 3175.38
3175.60 3178.64 3181.07 3181.62 3182.10 3183.46
3184.59 3187.56 3191.93 3192.15 3197.94 3201.03
3206.89 3207.83 3209.63 3218.17 3220.17 3220.31
3224.16 3226.40 3232.67
==============================
15A-TS2
==============================
-175.94 14.56 21.32 24.37 29.92 31.45
34.09 35.50 36.08 39.55 43.58 45.94
50.07 52.92 54.45 57.29 62.04 63.20
64.62 67.98 73.76 75.62 77.22 78.62
82.08 90.67 93.76 95.43 99.68 100.82
103.98 111.76 122.84 126.82 130.05 132.68
135.34 139.01 142.46 144.79 155.05 160.62
165.18 168.87 171.42 176.55 178.26 185.03
189.09 191.44 196.50 202.14 204.26 209.75
212.92 224.16 227.10 227.61 238.32 243.56
246.24 248.68 252.16 254.45 258.70 265.06
271.84 285.51 286.95 291.15 292.35 300.34
315.94 322.98 327.65 340.53 343.52 357.40
391.42 396.42 405.67 412.75 418.17 428.14
433.58 436.40 439.99 444.90 450.05 458.95
462.33 482.29 486.37 492.09 496.25 517.68
518.92 520.88 521.62 522.26 523.38 527.35
528.50 529.54 534.15 535.79 539.87 546.53
548.75 553.54 554.73 556.80 559.10 564.20
566.62 571.57 575.21 584.89 598.68 615.58
621.04 637.06 658.29 661.59 671.82 686.30
690.83 692.12 703.16 706.16 712.54 713.86
717.06 736.26 755.39 765.59 768.50 790.78
804.77 815.04 825.75 841.53 842.34 843.62
851.37 856.25 861.16 863.38 866.40 870.97
871.56 872.45 876.23 880.65 885.04 888.40
892.12 894.87 901.25 908.01 914.52 920.93
927.82 932.63 934.04 942.77 944.03 958.70
960.59 963.23 965.68 966.03 969.90 969.95
971.19 988.47 993.36 995.39 1004.40 1004.75
1005.46 1011.75 1012.78 1013.43 1014.74 1023.06
1023.71 1025.31 1025.84 1034.67 1040.48 1043.35
1047.26 1048.06 1048.25 1049.48 1057.60 1060.71
1063.03 1064.55 1064.97 1066.19 1066.45 1067.39
1068.51 1069.38 1071.50 1073.71 1076.69 1079.19
1110.19 1138.18 1156.43 1158.48 1162.57 1165.36
1167.64 1170.22 1172.27 1174.24 1177.38 1188.11
1197.53 1200.10 1204.92 1206.18 1206.36 1207.49
1211.73 1214.11 1215.91 1219.51 1231.59 1241.91
1256.98 1257.73 1260.72 1286.99 1289.03 1290.29
1291.15 1291.71 1295.65 1301.80 1306.80 1318.07
1323.32 1323.61 1328.70 1343.07 1347.65 1353.34
1358.85 1363.59 1364.40 1368.61 1394.29 1400.02
1403.75 1408.26 1410.31 1424.92 1426.40 1427.88
1428.85 1430.03 1430.37 1431.09 1431.13 1432.33
1453.66 1459.07 1461.95 1463.44 1464.42 1464.65
1464.93 1465.82 1467.80 1468.92 1469.87 1476.33
1478.16 1478.90 1492.08 1493.61 1496.47 1498.73
1498.99 1499.64 1501.71 1502.13 1503.06 1503.23
1505.34 1508.26 1510.31 1510.94 1513.13 1515.70
1517.26 1519.73 1520.62 1522.27 1523.79 1532.29
1550.80 1552.21 1604.56 1604.87 1634.87 1635.68
1636.46 1638.70 1640.49 1642.98 1649.74 1650.60
1651.47 1652.97 1673.08 1673.74 1676.09 1775.26
1896.86 2955.00 3000.72 3019.84 3027.93 3038.93
3039.34 3040.79 3041.58 3041.62 3041.76 3043.28
3045.93 3046.81 3054.28 3066.17 3099.95 3100.77
3100.94 3101.66 3103.55 3105.02 3105.69 3106.21
3111.70 3124.08 3124.56 3125.96 3128.23 3128.43
3128.66 3129.03 3129.30 3133.24 3133.77 3150.96
3150.97 3158.17 3166.42 3169.17 3170.83 3171.73
3178.39 3179.77 3181.23 3182.08 3182.84 3183.50
3183.55 3185.43 3189.89 3190.26 3191.27 3192.56
3196.92 3205.89 3206.91 3207.48 3216.11 3225.81
3229.13 3231.85 3237.73
==============================
15B-TS1
==============================
-319.48 10.62 16.13 18.92 23.34 28.11
28.31 30.28 35.72 38.26 40.18 40.95
42.53 47.17 47.28 48.81 51.12 54.90
55.54 64.67 68.83 72.35 74.90 79.44
81.40 83.35 90.17 94.06 96.46 100.21
107.98 110.21 121.73 129.07 134.71 136.89
139.30 145.77 148.44 153.26 155.67 158.31
164.02 167.73 170.57 173.14 175.80 182.68
187.24 188.92 196.87 201.27 202.51 205.68
208.75 214.47 220.69 227.53 235.46 237.65
241.45 246.67 251.08 254.17 258.35 267.14
269.67 278.07 282.75 287.29 290.48 292.24
301.34 309.20 319.88 326.55 329.88 344.29
396.04 397.10 399.59 413.33 419.17 431.67
444.08 446.84 448.32 449.86 456.57 464.20
483.12 485.56 490.33 496.57 501.29 517.04
519.14 520.13 520.53 524.79 526.37 526.94
527.65 532.15 532.99 535.29 537.59 545.99
549.38 553.43 556.28 559.69 561.00 563.76
565.65 569.71 573.88 576.99 597.95 599.80
620.26 623.23 634.21 649.91 657.55 676.91
686.35 690.79 703.51 706.33 711.61 713.78
716.70 757.66 762.38 768.00 771.05 789.83
805.19 814.22 827.04 833.16 837.16 841.03
853.79 858.43 863.38 864.74 866.74 872.79
876.02 877.35 877.72 882.03 885.30 888.49
894.38 895.97 902.42 906.27 911.83 924.17
926.37 930.34 934.63 938.91 950.34 958.40
961.05 964.13 965.26 968.08 968.25 971.81
976.18 982.94 993.69 1002.82 1004.59 1006.22
1009.49 1010.87 1013.20 1013.76 1014.15 1023.85
1024.46 1024.85 1025.22 1026.47 1045.47 1046.91
1048.17 1048.82 1049.49 1055.52 1057.85 1058.47
1064.28 1064.50 1065.32 1066.16 1066.70 1067.07
1069.26 1069.81 1070.49 1072.61 1078.71 1093.89
1136.37 1136.94 1155.01 1157.43 1161.33 1165.27
1168.50 1169.19 1172.40 1174.10 1176.09 1188.56
1199.47 1203.16 1204.00 1204.41 1204.97 1206.56
1209.95 1211.59 1214.05 1224.10 1244.27 1251.86
1255.28 1258.19 1269.01 1287.74 1290.08 1292.45
1292.75 1294.66 1301.43 1309.74 1317.06 1320.06
1320.81 1326.96 1331.11 1349.92 1353.17 1354.50
1357.56 1359.83 1364.23 1364.63 1390.35 1398.33
1408.29 1409.56 1411.06 1425.72 1426.41 1429.21
1429.73 1430.57 1430.90 1431.34 1432.20 1434.24
1454.75 1460.23 1462.73 1463.22 1464.51 1464.76

1465.72 1465.86 1467.10 1467.20 1478.14 1480.20
1484.13 1488.43 1491.81 1494.83 1495.67 1496.95
1497.96 1498.66 1499.14 1501.94 1502.13 1503.74
1505.46 1508.66 1509.41 1510.62 1511.50 1512.16
1514.65 1520.67 1521.00 1521.21 1522.13 1531.07
1548.30 1551.11 1598.47 1602.60 1635.37 1636.13
1637.07 1637.38 1637.72 1638.74 1649.81 1650.14
1650.88 1652.71 1653.03 1671.34 1672.00 1783.38
1865.29 2961.56 2986.46 3007.70 3030.83 3035.96
3038.55 3040.82 3041.10 3041.40 3041.84 3042.83
3046.14 3060.98 3065.22 3095.32 3097.94 3098.81
3100.34 3100.58 3102.06 3102.87 3104.22 3106.58
3110.68 3111.42 3121.70 3124.61 3125.33 3125.70
3126.79 3127.85 3128.28 3130.88 3139.20 3146.25
3166.82 3167.52 3168.46 3172.77 3179.54 3179.93
3180.45 3180.93 3183.45 3183.96 3184.34 3185.83
3187.67 3191.63 3191.76 3192.68 3193.40 3195.78
3200.76 3206.59 3206.92 3208.07 3217.74 3221.83
3225.07 3227.40 3230.27

===============================
15B-TS2
===============================

-198.89 16.77 21.78 23.49 25.95 31.73
32.64 34.85 35.35 41.62 44.78 47.01
53.19 56.11 58.64 59.65 60.22 65.04
68.82 70.64 72.03 77.40 81.43 82.16
89.35 93.40 93.91 100.00 101.27 106.56
110.09 111.60 119.07 125.28 127.85 134.88
135.06 140.22 150.67 151.99 162.24 163.80
166.05 174.02 178.25 185.72 187.99 194.87
195.75 200.65 205.75 208.45 212.62 219.49
222.59 226.61 230.11 239.76 241.07 244.42
247.36 250.20 254.17 259.74 261.12 269.46
282.29 286.37 289.31 293.29 299.06 307.75
315.98 328.13 334.66 342.54 345.96 375.27
389.53 393.94 408.18 413.08 420.87 429.40
440.44 446.05 451.74 460.12 460.97 466.12
476.80 483.66 485.25 497.07 499.28 515.65
516.22 517.54 519.14 520.75 526.23 527.64
529.76 529.94 534.39 534.61 540.99 541.85
546.47 548.57 551.32 556.21 558.69 563.31
564.92 568.94 571.01 579.87 600.75 602.89
621.94 636.08 639.46 658.49 668.31 687.51
691.51 703.39 707.87 712.23 715.05 719.12
726.77 758.21 763.95 768.27 769.29 791.26
805.39 814.98 820.67 841.56 842.67 846.43
851.74 853.88 856.81 859.81 863.68 868.84
871.88 874.15 878.71 878.76 884.47 887.70
893.24 894.81 898.21 915.20 916.68 926.35
931.25 937.62 939.29 941.66 942.24 955.05
958.81 961.80 964.33 966.15 967.11 970.49
971.74 978.52 989.23 1002.16 1003.84 1005.53
1005.74 1011.63 1012.12 1013.42 1014.89 1017.13
1024.20 1024.70 1028.07 1033.51 1033.63 1043.90
1045.15 1047.60 1052.26 1056.08 1057.35 1060.76
1064.51 1064.84 1065.32 1066.00 1066.85 1067.24
1069.50 1070.05 1076.24 1076.98 1091.34 1099.64
1119.40 1136.34 1139.88 1158.21 1161.15 1164.70
1168.42 1169.84 1173.92 1174.18 1182.77 1187.90
1199.81 1202.70 1204.37 1205.86 1206.74 1209.31
1210.03 1211.73 1216.65 1224.65 1242.75 1256.74
1257.89 1264.57 1270.28 1288.45 1289.76 1291.27
1292.98 1295.66 1301.62 1308.19 1316.55 1319.36
1321.65 1326.00 1329.94 1341.36 1347.57 1349.41
1357.49 1362.05 1363.06 1363.51 1387.24 1391.40
1408.47 1410.47 1410.93 1426.09 1428.03 1428.43
1429.60 1430.12 1430.28 1430.37 1431.56 1453.14
1459.65 1461.29 1462.71 1463.13 1464.44 1465.20
1466.58 1466.97 1466.98 1468.05 1471.00 1473.29
1478.02 1484.86 1493.24 1496.61 1498.24 1499.55
1500.27 1501.68 1501.85 1502.66 1503.09 1503.36
1505.72 1505.99 1507.40 1509.32 1511.91 1513.75
1517.12 1520.65 1521.21 1522.37 1524.30 1531.59
1550.98 1554.14 1603.05 1603.92 1631.35 1635.11
1636.46 1638.19 1639.06 1645.40 1645.62 1649.75
1650.60 1651.05 1672.69 1674.70 1689.37 1784.93
1909.86 2806.96 2870.95 2910.44 2970.37 2987.44
3031.68 3036.01 3038.87 3039.25 3039.85 3040.64
3040.73 3043.91 3047.38 3066.29 3093.13 3094.14
3097.38 3097.41 3101.67 3102.59 3105.64 3113.11
3114.50 3117.93 3118.65 3121.78 3122.59 3124.82
3125.24 3125.61 3125.80 3128.59 3129.02 3143.73
3147.97 3165.29 3169.70 3170.80 3173.41 3176.50
3177.24 3178.02 3179.62 3179.70 3181.17 3182.74
3184.20 3189.66 3191.24 3195.04 3196.23 3196.50
3205.42 3206.96 3211.03 3211.86 3216.28 3223.94
3231.17 3234.63 3235.73

===============================
16A
===============================

6.45 15.75 21.35 26.50 30.79 34.78
39.27 40.24 40.41 43.34 44.46 47.24
48.51 51.17 51.81 53.37 54.30 55.99
60.85 62.50 66.54 70.86 73.74 75.99
80.99 84.85 91.94 95.25 97.85 100.66
105.09 108.54 115.66 118.97 127.19 131.24
139.75 142.83 153.53 158.27 161.60 167.58
168.46 170.20 178.03 180.88 188.41 191.42
192.03 194.93 198.15 198.88 199.81 206.51
214.99 220.61 225.82 229.83 230.79 239.42
240.00 246.49 253.61 256.35 260.05 269.18
282.67 286.59 292.68 293.11 296.49 319.58
321.86 324.04 330.62 339.79 367.72 389.42
393.76 409.05 410.76 414.74 425.95 430.18
446.26 450.10 455.56 460.48 463.14 477.09
486.51 491.89 496.14 509.78 510.86 516.08
519.57 520.02 521.02 522.48 524.12 526.97
530.69 532.75 533.47 534.48 536.01 544.31
546.60 547.49 549.42 556.95 559.14 565.14
569.39 571.81 594.63 604.44 616.23 628.09
633.44 644.22 644.64 675.47 682.70 698.32
703.98 705.61 706.83 708.10 709.09 717.23
756.03 765.16 769.36 778.23 785.70 803.35
807.94 810.47 837.94 839.15 842.50 849.36
852.80 855.89 863.21 868.59 871.58 877.47
879.58 880.10 885.52 890.47 895.17 900.71
902.49 911.62 914.50 927.81 929.79 934.61
937.91 940.87 943.42 944.17 949.86 956.68
957.34 960.67 962.23 962.96 964.74 969.15
972.42 987.18 989.87 999.27 1005.03 1006.52
1007.46 1010.28 1011.00 1012.52 1015.88 1021.28
1022.77 1023.85 1025.32 1038.53 1044.78 1045.70

1046.20 1047.02 1050.07 1050.58 1057.31 1063.81
1064.28 1065.76 1065.90 1067.82 1068.08 1068.16
1069.15 1072.59 1073.87 1087.35 1104.84 1116.87
1136.37 1142.68 1159.07 1159.72 1160.90 1167.89
1170.86 1174.60 1175.37 1179.22 1188.04 1200.44
1203.65 1203.74 1206.02 1207.56 1208.80 1214.29
1214.42 1222.06 1239.11 1242.52 1253.42 1257.60
1267.82 1279.03 1284.67 1287.42 1290.97 1292.14
1292.68 1293.30 1298.50 1316.50 1321.32 1322.89
1324.04 1325.81 1334.94 1346.58 1351.10 1359.46
1362.61 1363.36 1364.33 1367.73 1386.46 1407.67
1407.87 1410.41 1421.70 1425.87 1425.91 1427.10
1427.82 1428.85 1429.22 1430.39 1430.75 1431.48
1452.69 1455.46 1457.01 1460.03 1461.12 1461.93
1463.62 1465.07 1466.61 1467.27 1479.10 1485.80
1493.16 1493.56 1494.21 1496.01 1496.41 1496.50
1496.94 1497.55 1498.08 1499.24 1502.80 1503.49
1504.82 1506.32 1506.72 1507.21 1508.73 1510.73
1511.85 1518.06 1518.48 1520.77 1521.41 1526.34
1547.81 1550.33 1595.93 1600.69 1607.72 1634.05
1634.79 1635.69 1636.94 1638.51 1639.46 1645.78
1648.59 1650.71 1651.28 1655.29 1674.38 1674.61
1775.55 3002.69 3024.65 3030.46 3038.07 3040.36
3040.68 3040.98 3041.64 3042.17 3042.36 3050.22
3062.73 3070.32 3086.82 3100.00 3100.77 3101.55
3102.60 3103.07 3103.24 3105.41 3110.69 3117.49
3119.03 3123.05 3123.41 3124.65 3126.07 3128.14
3128.40 3128.52 3129.37 3131.58 3131.90 3135.71
3156.97 3157.65 3167.12 3168.29 3169.71 3170.51
3175.54 3180.78 3181.24 3181.83 3183.44 3185.12
3188.47 3191.55 3191.63 3194.70 3199.91 3205.80
3206.16 3208.86 3214.04 3218.36 3224.99 3230.96
3233.82 3238.53 3254.16

==============================
16B
==============================

4.18 13.05 15.88 23.04 28.76 31.71
37.01 38.56 40.07 41.34 42.66 47.41
50.89 52.54 53.16 55.01 56.89 58.51
60.36 61.60 67.14 69.35 71.27 74.87
79.72 86.53 94.95 98.17 99.92 102.03
103.47 112.80 116.88 127.05 132.75 134.67
140.59 144.63 158.80 159.86 162.37 167.72
171.57 174.70 178.46 187.29 191.72 193.49
195.12 196.87 199.61 201.42 210.11 218.47
221.21 228.19 233.91 237.03 243.41 246.34
252.45 264.39 269.69 275.29 282.02 285.91
286.82 291.86 293.15 298.06 304.11 308.29
324.76 328.84 344.60 346.35 372.00 379.46
392.30 397.99 404.80 414.81 423.40 430.29
437.85 449.01 450.02 453.61 459.92 470.36
471.63 489.43 498.29 504.31 506.87 516.61
517.75 521.03 521.79 524.64 526.33 528.31
530.84 532.37 533.80 535.40 537.96 547.51
549.48 553.71 555.79 558.96 562.88 565.72
569.71 576.47 592.11 604.21 610.03 611.83
632.82 647.17 664.05 668.81 684.32 702.48
706.84 708.62 711.31 716.29 722.24 723.65
764.83 768.85 769.54 773.27 784.15 804.17
806.74 832.82 834.31 837.99 840.86 843.94
852.04 856.97 857.92 869.50 871.56 872.89
875.02 883.02 889.38 889.97 891.12 896.05
903.79 904.29 919.25 930.64 932.30 935.52
942.31 944.20 946.40 950.45 955.18 958.19
963.79 964.33 965.46 969.01 970.24 975.96
976.52 990.86 992.90 1003.81 1007.53 1010.36
1011.22 1013.72 1014.47 1014.63 1021.46 1022.95
1024.79 1025.77 1027.66 1035.60 1044.68 1046.19
1046.39 1047.72 1047.99 1058.21 1061.73 1065.36
1065.50 1066.35 1067.09 1067.35 1068.26 1068.95
1070.84 1073.75 1076.93 1100.35 1129.79 1131.69
1134.55 1146.22 1161.69 1162.44 1164.96 1167.02
1168.55 1172.99 1176.34 1178.61 1183.43 1189.06
1202.36 1203.93 1206.14 1206.27 1208.28 1210.12
1217.21 1217.78 1224.50 1240.86 1250.85 1257.42
1262.31 1275.10 1281.54 1282.62 1284.79 1290.24
1290.33 1292.48 1298.87 1319.63 1320.27 1327.20
1327.59 1330.47 1332.77 1343.49 1344.76 1355.15
1359.50 1360.03 1363.17 1366.87 1389.42 1401.57
1408.93 1413.64 1414.41 1427.51 1428.20 1429.02
1429.42 1430.42 1431.16 1431.38 1431.51 1432.60
1453.14 1457.44 1458.17 1459.27 1462.34 1464.41
1465.22 1465.52 1467.22 1467.74 1476.22 1479.51
1483.48 1490.45 1493.33 1493.86 1494.04 1496.71
1497.38 1498.22 1499.72 1500.34 1500.53 1500.59
1501.15 1502.94 1507.27 1510.23 1510.48 1512.02
1512.54 1518.72 1520.14 1522.70 1523.06 1525.22
1547.82 1550.93 1580.06 1602.50 1607.22 1626.78
1633.61 1634.39 1636.80 1639.09 1639.85 1642.75
1648.53 1649.65 1650.18 1651.47 1674.00 1674.58
1787.83 2961.65 2995.32 3037.29 3037.62 3037.77
3039.62 3040.55 3041.25 3042.05 3043.98 3046.56
3061.90 3079.55 3094.49 3095.96 3098.62 3099.76
3099.91 3101.51 3102.03 3105.26 3110.37 3114.97
3122.33 3123.61 3126.45 3127.78 3128.34 3129.04
3129.31 3129.64 3131.34 3140.94 3144.07 3165.10
3167.93 3170.49 3170.79 3171.67 3171.96 3172.58
3173.43 3175.53 3176.89 3178.27 3180.02 3180.81
3182.04 3183.77 3184.78 3190.78 3191.84 3196.59
3203.78 3205.02 3205.83 3206.66 3210.69 3222.40
3231.68 3239.46 3254.97

==============================
18A
==============================

12.81 19.04 23.29 24.77 31.78 32.38
32.85 36.08 37.47 39.29 42.23 49.02
51.97 52.63 54.72 55.89 59.93 64.56
66.25 69.76 71.50 72.63 74.72 78.59
80.16 84.95 88.23 92.37 93.16 97.10
103.87 109.69 119.29 122.93 125.02 144.82
150.80 155.66 160.11 167.11 171.31 173.88
176.93 178.39 184.65 188.65 191.87 194.97
198.41 203.98 204.37 213.46 218.43 221.77
227.98 231.58 233.98 235.68 239.42 243.77
246.69 252.78 256.42 259.24 267.59 281.41
283.46 285.74 289.40 295.40 296.83 306.38
311.10 318.07 337.85 341.56 361.25 391.19
396.29 400.77 411.40 416.14 424.07 438.38
444.92 449.56 453.61 462.79 465.45 485.25
489.00 491.55 493.94 499.43 505.49 518.15
518.44 520.01 522.30 523.46 524.72 525.89
526.84 528.44 534.41 536.89 542.50 542.82

| | | | | | |
|---|---|---|---|---|---|
| 546.22 | 555.88 | 559.02 | 561.39 | 563.09 | 564.43 |
| 568.53 | 574.41 | 601.15 | 609.28 | 619.03 | 621.83 |
| 631.59 | 657.43 | 660.52 | 686.63 | 689.02 | 691.49 |
| 699.81 | 704.38 | 705.52 | 705.98 | 709.11 | 713.28 |
| 765.69 | 769.57 | 774.90 | 784.80 | 788.85 | 804.22 |
| 806.19 | 812.12 | 829.57 | 840.84 | 848.65 | 851.51 |
| 853.28 | 860.32 | 860.94 | 863.78 | 865.09 | 868.23 |
| 871.14 | 876.24 | 876.87 | 879.28 | 880.70 | 892.97 |
| 895.65 | 899.25 | 905.85 | 912.43 | 917.18 | 929.61 |
| 937.50 | 940.42 | 941.96 | 947.23 | 951.50 | 957.66 |
| 960.28 | 965.48 | 966.54 | 971.02 | 972.07 | 975.09 |
| 985.42 | 997.53 | 999.59 | 1001.71 | 1004.70 | 1006.68 |
| 1008.22 | 1009.99 | 1013.03 | 1013.38 | 1015.63 | 1023.47 |
| 1023.83 | 1025.36 | 1026.88 | 1031.50 | 1041.60 | 1043.33 |
| 1046.55 | 1047.22 | 1047.62 | 1056.61 | 1060.71 | 1063.52 |
| 1064.66 | 1066.60 | 1066.89 | 1067.58 | 1068.04 | 1068.66 |
| 1070.59 | 1072.12 | 1075.87 | 1079.26 | 1102.85 | 1133.92 |
| 1134.59 | 1157.14 | 1158.66 | 1160.07 | 1161.63 | 1165.89 |
| 1167.18 | 1175.35 | 1178.54 | 1188.74 | 1199.47 | 1205.84 |
| 1207.90 | 1208.10 | 1209.24 | 1210.15 | 1210.74 | 1213.40 |
| 1214.77 | 1220.98 | 1240.60 | 1244.97 | 1257.05 | 1258.17 |
| 1262.43 | 1285.23 | 1290.07 | 1291.02 | 1291.63 | 1295.48 |
| 1302.42 | 1303.85 | 1311.98 | 1317.87 | 1320.61 | 1321.07 |
| 1325.52 | 1330.77 | 1346.63 | 1357.37 | 1357.82 | 1359.10 |
| 1360.00 | 1362.05 | 1366.78 | 1379.36 | 1386.88 | 1405.49 |
| 1408.35 | 1411.30 | 1415.54 | 1425.84 | 1425.89 | 1427.89 |
| 1428.78 | 1429.16 | 1429.35 | 1430.56 | 1431.59 | 1432.74 |
| 1435.48 | 1454.82 | 1456.60 | 1460.13 | 1460.68 | 1462.39 |
| 1463.50 | 1464.60 | 1467.19 | 1468.44 | 1468.86 | 1475.96 |
| 1481.34 | 1490.53 | 1493.44 | 1497.17 | 1497.44 | 1498.20 |
| 1499.90 | 1500.22 | 1500.44 | 1501.27 | 1501.77 | 1502.28 |
| 1502.95 | 1506.88 | 1507.55 | 1510.24 | 1510.81 | 1513.10 |
| 1514.63 | 1516.98 | 1517.63 | 1521.60 | 1524.70 | 1530.63 |
| 1550.94 | 1553.39 | 1596.71 | 1602.36 | 1632.80 | 1633.93 |
| 1635.81 | 1636.11 | 1637.93 | 1639.28 | 1648.56 | 1649.48 |
| 1650.42 | 1651.41 | 1673.09 | 1673.76 | 1674.65 | 1688.66 |
| 1781.69 | 2886.45 | 2996.87 | 3004.30 | 3017.12 | 3025.78 |
| 3032.56 | 3035.53 | 3038.71 | 3039.04 | 3039.37 | 3041.73 |
| 3042.83 | 3043.98 | 3047.36 | 3063.80 | 3086.09 | 3086.83 |
| 3097.48 | 3097.55 | 3100.77 | 3101.42 | 3101.78 | 3103.71 |
| 3106.56 | 3112.81 | 3113.62 | 3125.25 | 3126.72 | 3128.02 |
| 3129.73 | 3131.22 | 3132.62 | 3132.97 | 3133.59 | 3138.50 |
| 3144.79 | 3154.06 | 3154.18 | 3170.28 | 3172.05 | 3174.12 |
| 3174.50 | 3175.95 | 3176.62 | 3180.90 | 3182.09 | 3183.59 |
| 3185.49 | 3186.06 | 3191.11 | 3193.62 | 3199.25 | 3202.25 |
| 3203.88 | 3206.39 | 3208.47 | 3209.23 | 3212.86 | 3219.71 |
| 3222.49 | 3226.64 | 3233.87 | | | |

==============================

18B

==============================

| | | | | | |
|---|---|---|---|---|---|
| 12.27 | 14.34 | 18.00 | 21.86 | 25.37 | 28.38 |
| 34.89 | 36.32 | 39.34 | 41.69 | 43.98 | 47.06 |
| 47.83 | 51.64 | 55.63 | 61.56 | 64.71 | 64.90 |
| 65.35 | 70.63 | 71.99 | 72.59 | 75.85 | 79.32 |
| 80.52 | 84.41 | 88.65 | 93.34 | 96.00 | 105.39 |
| 109.36 | 115.15 | 119.86 | 125.22 | 138.03 | 144.10 |
| 152.73 | 156.08 | 158.97 | 161.30 | 166.43 | 170.44 |
| 171.40 | 175.91 | 177.78 | 181.12 | 186.16 | 191.91 |
| 193.14 | 198.28 | 203.50 | 204.37 | 210.82 | 218.20 |
| 223.81 | 225.91 | 230.90 | 231.66 | 237.59 | 237.92 |
| 240.65 | 251.31 | 255.08 | 255.86 | 258.34 | 280.27 |
| 283.82 | 288.64 | 295.99 | 296.77 | 302.46 | 306.41 |
| 310.64 | 328.03 | 336.39 | 341.94 | 357.06 | 390.97 |
| 396.51 | 401.95 | 413.45 | 418.13 | 420.80 | 424.69 |
| 447.05 | 449.57 | 454.12 | 463.15 | 466.53 | 467.75 |
| 485.18 | 489.19 | 490.45 | 500.97 | 508.57 | 517.59 |
| 518.27 | 518.55 | 520.46 | 523.64 | 523.85 | 525.33 |
| 526.27 | 529.80 | 535.86 | 537.02 | 543.09 | 544.22 |
| 548.88 | 555.83 | 558.99 | 559.45 | 564.69 | 565.01 |
| 568.17 | 576.57 | 602.30 | 608.24 | 622.82 | 623.89 |
| 631.88 | 659.16 | 686.99 | 688.28 | 690.01 | 701.88 |
| 705.39 | 706.01 | 706.21 | 708.56 | 713.63 | 746.34 |
| 765.13 | 768.79 | 769.94 | 788.50 | 789.02 | 804.20 |
| 810.30 | 812.19 | 841.95 | 847.81 | 848.95 | 852.37 |
| 854.37 | 861.12 | 863.07 | 865.73 | 867.64 | 871.24 |
| 872.64 | 876.78 | 878.11 | 878.97 | 881.10 | 892.74 |
| 895.21 | 902.84 | 907.25 | 912.31 | 915.42 | 930.44 |
| 934.53 | 937.91 | 939.22 | 942.82 | 952.52 | 958.44 |
| 960.95 | 965.44 | 966.81 | 970.46 | 971.06 | 973.63 |
| 985.79 | 997.87 | 1000.64 | 1002.67 | 1006.17 | 1006.77 |
| 1009.48 | 1013.22 | 1014.39 | 1015.50 | 1023.05 | 1023.75 |
| 1024.11 | 1024.29 | 1025.26 | 1033.06 | 1041.77 | 1043.66 |
| 1046.56 | 1046.65 | 1054.50 | 1055.76 | 1059.14 | 1063.07 |
| 1065.15 | 1065.46 | 1065.88 | 1066.50 | 1067.59 | 1068.37 |
| 1068.70 | 1070.77 | 1075.50 | 1089.30 | 1114.18 | 1134.48 |
| 1136.52 | 1160.82 | 1162.60 | 1164.26 | 1169.23 | 1170.33 |
| 1175.27 | 1177.24 | 1187.95 | 1198.84 | 1204.85 | 1206.85 |
| 1208.90 | 1209.53 | 1209.57 | 1209.98 | 1210.53 | 1214.34 |
| 1215.61 | 1231.69 | 1244.18 | 1251.88 | 1256.16 | 1257.37 |
| 1283.87 | 1288.23 | 1291.47 | 1292.13 | 1292.64 | 1294.91 |
| 1300.16 | 1301.78 | 1318.10 | 1318.84 | 1320.42 | 1322.91 |
| 1325.87 | 1345.63 | 1356.84 | 1358.90 | 1360.04 | 1362.34 |
| 1362.88 | 1365.55 | 1371.72 | 1383.12 | 1385.05 | 1407.43 |
| 1410.54 | 1415.71 | 1419.50 | 1425.37 | 1426.04 | 1426.81 |
| 1428.49 | 1428.89 | 1429.07 | 1430.60 | 1431.03 | 1431.36 |
| 1456.35 | 1456.97 | 1461.12 | 1461.64 | 1462.32 | 1465.29 |
| 1465.60 | 1466.34 | 1467.44 | 1468.81 | 1482.77 | 1488.56 |
| 1494.34 | 1496.82 | 1497.05 | 1497.76 | 1499.20 | 1499.93 |
| 1500.70 | 1501.74 | 1502.13 | 1502.39 | 1502.62 | 1503.03 |
| 1503.40 | 1503.85 | 1507.45 | 1509.65 | 1510.35 | 1511.88 |
| 1514.87 | 1518.30 | 1518.83 | 1520.64 | 1521.92 | 1533.42 |
| 1549.09 | 1553.10 | 1595.18 | 1601.48 | 1609.89 | 1634.83 |
| 1635.07 | 1636.28 | 1637.74 | 1638.34 | 1639.01 | 1648.96 |
| 1649.95 | 1651.41 | 1652.28 | 1671.84 | 1674.43 | 1685.65 |
| 1691.74 | 2969.39 | 3004.54 | 3023.50 | 3029.65 | 3032.65 |
| 3034.95 | 3035.48 | 3039.36 | 3039.93 | 3040.29 | 3041.19 |
| 3043.16 | 3047.47 | 3048.82 | 3051.31 | 3074.29 | 3082.24 |
| 3094.51 | 3095.10 | 3096.34 | 3098.19 | 3101.07 | 3102.01 |
| 3102.34 | 3103.79 | 3113.53 | 3124.37 | 3126.60 | 3128.30 |
| 3128.80 | 3129.21 | 3130.14 | 3130.33 | 3131.42 | 3131.59 |
| 3164.87 | 3166.95 | 3170.19 | 3170.45 | 3172.40 | 3177.79 |
| 3179.26 | 3180.88 | 3181.83 | 3183.58 | 3185.19 | 3185.28 |
| 3190.13 | 3192.24 | 3192.42 | 3196.02 | 3196.33 | 3201.18 |
| 3202.73 | 3203.30 | 3207.89 | 3208.73 | 3211.95 | 3215.23 |
| 3221.29 | 3225.57 | 3233.69 | | | |

==============================

16A-TS

==============================

| | | | | | |
|---|---|---|---|---|---|
| -322.35 | 4.71 | 14.71 | 27.09 | 30.44 | 34.10 |
| 38.56 | 40.46 | 44.32 | 46.82 | 50.88 | 53.18 |
| 54.57 | 56.41 | 59.31 | 66.29 | 67.72 | 69.15 |
| 73.94 | 75.49 | 76.65 | 78.22 | 80.24 | 88.69 |

91.11 93.34 96.24 97.28 101.92 107.98
109.62 113.47 118.48 124.48 125.80 140.51
146.61 149.14 153.91 160.60 162.71 166.58
172.39 175.52 178.08 185.74 193.53 193.69
195.38 198.54 204.60 205.82 206.30 209.91
222.98 226.65 229.51 234.58 238.10 241.53
246.19 255.86 265.18 270.23 277.45 283.53
286.29 297.46 299.01 304.83 306.15 319.51
322.77 326.49 336.52 341.55 362.79 383.59
397.95 401.57 414.27 417.35 424.78 429.17
445.77 451.02 452.40 461.25 465.64 481.99
486.09 487.38 494.94 496.84 504.43 514.59
519.17 519.85 521.61 524.51 526.74 527.79
531.05 533.01 537.34 542.21 544.57 546.25
548.66 549.97 551.31 552.76 556.75 561.05
563.26 564.20 567.44 573.85 597.11 617.94
633.22 640.19 652.22 679.92 689.73 705.26
705.82 708.28 712.45 715.76 719.53 725.32
766.12 767.87 769.13 777.94 788.75 803.96
804.95 813.92 831.71 837.78 845.67 856.01
860.32 864.85 868.80 870.05 872.06 875.10
877.26 881.19 883.42 887.93 890.27 892.97
904.46 907.99 919.28 927.50 929.23 933.72
939.01 945.30 949.16 955.55 957.93 959.48
962.39 963.06 968.25 969.65 971.86 972.74
984.29 985.44 986.82 991.45 1005.25 1005.80
1009.90 1012.43 1013.69 1013.94 1015.48 1022.65
1024.03 1024.51 1025.11 1025.30 1039.10 1044.94
1046.58 1047.09 1049.25 1055.72 1059.37 1063.61
1065.00 1065.36 1065.38 1066.02 1067.32 1068.49
1069.96 1072.75 1075.94 1080.95 1103.40 1108.27
1141.26 1152.84 1156.60 1158.09 1162.35 1164.51
1166.40 1169.39 1174.71 1181.39 1189.74 1201.44
1203.81 1204.16 1204.61 1207.08 1208.18 1216.40
1216.66 1217.35 1238.77 1244.01 1251.19 1259.27
1262.73 1272.58 1284.69 1289.35 1291.31 1291.70
1292.15 1295.22 1301.33 1316.75 1318.03 1318.89
1321.74 1323.21 1339.55 1346.49 1350.07 1359.17
1360.02 1360.54 1361.99 1368.32 1391.06 1412.24
1412.32 1412.51 1416.79 1423.66 1423.98 1426.52
1426.79 1427.76 1427.90 1430.81 1430.90 1434.22
1451.96 1455.42 1460.31 1463.05 1463.76 1464.60
1465.58 1465.90 1466.12 1468.84 1472.78 1481.08
1487.48 1488.56 1493.92 1494.90 1496.71 1498.17
1498.61 1499.85 1500.62 1500.91 1501.80 1502.27
1502.49 1503.88 1506.00 1508.30 1509.82 1510.78
1511.13 1518.00 1518.45 1520.93 1523.94 1528.91
1542.55 1552.39 1553.45 1601.58 1607.12 1618.06
1630.37 1633.55 1636.96 1638.41 1641.73 1642.95
1645.99 1648.07 1651.16 1651.75 1674.31 1674.90
1750.44 3026.91 3032.67 3035.77 3035.86 3038.90
3040.19 3040.92 3041.08 3042.34 3044.38 3045.03
3052.81 3059.07 3070.87 3092.52 3097.68 3098.61
3101.63 3103.68 3104.13 3105.50 3106.66 3107.74
3110.25 3121.91 3125.59 3125.60 3127.37 3128.24
3128.31 3129.04 3129.54 3130.62 3157.17 3159.47
3162.46 3165.51 3168.20 3170.03 3170.85 3173.95
3175.45 3175.82 3178.24 3179.43 3180.98 3182.25
3182.34 3184.24 3184.89 3188.25 3190.56 3192.79
3204.15 3205.10 3206.00 3219.09 3220.22 3230.49
3238.03 3239.29 3266.01

===============================

16B-TS

===============================

-312.17 11.29 20.26 28.51 33.06 36.23
38.89 41.60 44.84 46.52 46.89 50.69
51.26 52.60 55.43 58.18 60.84 63.13
65.39 69.45 71.57 76.01 77.98 81.64
88.33 93.20 97.58 102.33 107.00 109.74
114.74 125.91 129.19 136.67 143.72 146.96
161.19 164.17 168.46 171.49 172.71 176.02
180.72 181.64 184.51 189.05 193.04 194.67
195.72 199.52 200.28 205.14 216.13 217.66
221.82 227.63 229.23 234.97 236.30 248.43
252.74 254.96 270.51 278.39 281.93 286.69
289.49 290.56 296.32 300.26 315.68 318.08
323.07 324.37 334.69 340.35 358.02 361.76
394.60 402.66 408.93 414.48 422.56 426.91
438.91 445.37 450.98 453.92 462.40 467.94
483.52 486.24 492.22 492.92 500.05 501.06
514.69 515.62 516.00 519.68 522.84 525.28
526.74 529.14 531.87 532.92 534.77 537.54
543.35 546.79 550.88 556.42 561.11 561.34
565.48 568.98 571.51 588.03 595.78 605.82
628.42 630.42 640.20 661.42 682.96 683.92
701.58 703.99 707.37 716.16 717.05 726.30
739.81 762.71 767.54 767.94 785.88 792.39
801.11 804.20 828.88 838.31 840.13 842.37
844.89 856.26 858.98 865.29 867.09 869.98
874.15 875.75 883.22 885.19 891.05 893.53
901.03 904.18 922.66 924.88 930.72 932.29
934.26 943.18 945.49 945.84 949.98 962.67
964.74 967.55 968.47 971.59 972.61 973.77
983.43 987.98 989.16 991.43 1005.64 1006.04
1006.75 1013.20 1013.57 1014.10 1023.10 1023.69
1024.32 1024.67 1025.59 1026.77 1041.01 1046.52
1047.04 1048.07 1050.00 1056.68 1064.34 1064.66
1065.34 1065.70 1065.86 1066.33 1066.99 1067.18
1069.49 1074.34 1075.74 1093.30 1106.81 1114.12
1130.09 1149.81 1156.57 1160.56 1162.23 1167.18
1168.81 1172.28 1175.02 1178.23 1188.85 1190.68
1200.85 1204.01 1205.96 1206.14 1207.34 1208.01
1215.69 1218.96 1224.38 1239.21 1249.08 1255.59
1262.55 1276.58 1280.19 1288.83 1290.20 1291.27
1291.77 1292.30 1299.22 1319.23 1319.34 1322.66
1323.79 1327.52 1333.57 1344.88 1345.49 1361.01
1361.80 1362.20 1364.74 1370.66 1389.49 1408.82
1412.48 1414.33 1417.28 1422.72 1424.06 1424.27
1426.52 1426.57 1426.74 1429.95 1430.06 1431.88
1453.50 1455.39 1457.39 1462.36 1462.72 1464.40
1465.27 1465.50 1467.53 1468.19 1475.68 1479.72
1482.28 1492.81 1492.94 1494.06 1498.84 1499.73
1500.52 1500.88 1501.44 1501.72 1501.99 1502.82
1502.99 1507.86 1508.46 1509.22 1509.78 1511.74
1512.70 1518.20 1518.55 1521.40 1522.10 1532.57
1547.17 1549.13 1551.89 1601.64 1604.78 1617.38
1633.39 1634.72 1637.09 1637.78 1638.83 1640.24
1647.90 1649.08 1651.60 1652.14 1673.71 1674.60
1780.38 3035.71 3037.62 3037.84 3041.59 3041.91
3042.24 3042.55 3045.10 3046.38 3047.74 3048.41
3052.78 3067.77 3070.30 3099.05 3099.16 3101.77
3103.58 3107.69 3109.55 3110.85 3114.10 3114.66

3114.84 3118.36 3121.45 3123.09 3125.75 3127.56
3129.35 3129.90 3130.53 3132.71 3149.44 3155.97
3163.12 3168.35 3169.58 3170.77 3172.16 3177.07
3177.14 3177.17 3177.50 3178.82 3179.33 3180.06
3180.17 3180.35 3188.46 3188.99 3191.33 3193.16
3202.11 3204.52 3205.02 3209.88 3218.43 3224.49
3226.35 3245.17 3250.81

==============================
18A-TS
==============================

-258.09 8.83 17.87 20.80 29.37 30.26
37.08 38.85 40.38 42.87 44.91 46.23
48.28 51.85 52.92 55.73 57.12 64.33
65.16 67.98 71.92 73.10 84.09 87.23
94.51 100.57 103.53 107.94 113.04 116.13
124.50 127.59 133.94 143.18 145.33 153.80
154.62 158.82 164.68 167.36 173.43 178.32
179.85 183.73 188.11 188.93 194.80 199.54
207.51 208.79 212.64 215.46 220.38 226.37
233.61 235.62 239.92 246.66 252.69 256.45
262.17 267.16 270.91 285.34 286.01 289.11
296.53 299.23 303.11 311.32 328.67 332.37
341.34 341.85 369.40 390.25 397.37 399.49
414.36 418.73 426.40 431.86 437.07 441.55
445.22 453.76 463.84 470.69 474.30 476.85
487.26 497.04 500.19 517.30 518.93 519.96
522.40 523.78 527.27 528.41 530.05 530.63
532.10 537.37 543.26 547.62 548.50 552.21
553.63 555.34 558.80 561.74 567.73 571.41
579.16 595.17 610.76 614.93 620.87 636.23
654.61 661.69 676.31 681.50 690.80 704.19
706.83 710.41 713.90 715.58 718.27 739.43
767.68 768.91 769.75 792.07 804.49 814.64
816.14 835.61 837.85 839.41 840.08 854.86
860.03 864.38 866.34 871.76 875.29 876.99
879.12 879.80 884.86 887.39 892.55 894.38
905.01 914.55 915.74 921.04 931.16 934.53
942.62 944.34 944.79 947.56 957.83 961.01
963.25 966.04 969.91 972.40 974.53 975.98
989.66 991.48 991.70 1005.51 1007.92 1008.64
1009.36 1011.76 1013.92 1015.31 1020.31 1024.57
1025.55 1026.08 1038.13 1047.54 1048.07 1049.12
1049.87 1051.33 1056.91 1061.78 1065.30 1065.36
1065.73 1065.91 1067.54 1068.41 1072.66 1074.20
1075.70 1082.00 1087.47 1110.83 1128.50 1137.51
1157.56 1158.39 1161.21 1161.92 1165.52 1171.94
1175.61 1178.87 1183.57 1188.51 1201.30 1203.94
1204.86 1205.20 1206.67 1209.73 1210.07 1213.16
1214.66 1223.80 1228.42 1240.77 1253.41 1255.60
1257.92 1266.25 1273.12 1282.43 1284.55 1286.19
1293.55 1296.01 1300.82 1301.35 1307.07 1319.18
1321.78 1322.59 1329.43 1338.13 1343.61 1352.04
1358.98 1360.92 1363.15 1365.42 1366.21 1387.78
1396.34 1407.25 1411.95 1428.39 1428.55 1429.32
1429.68 1430.31 1430.96 1431.89 1432.90 1434.54
1453.57 1454.98 1458.51 1462.19 1463.03 1463.42
1464.20 1464.56 1467.73 1469.33 1473.97 1480.57
1490.12 1492.46 1495.36 1496.95 1497.27 1499.10
1500.64 1501.13 1501.94 1502.72 1502.92 1503.73
1505.38 1505.85 1508.91 1509.77 1512.28 1512.44
1513.74 1518.59 1519.35 1523.86 1524.66 1525.26
1537.40 1547.15 1553.11 1588.38 1612.36 1621.69
1629.50 1632.17 1636.14 1636.91 1638.44 1646.41
1647.12 1648.34 1650.69 1652.45 1670.95 1673.80
1773.03 2393.88 2414.47 2531.56 3005.48 3021.73
3030.59 3033.33 3037.70 3039.84 3040.07 3040.13
3041.35 3042.98 3043.67 3046.31 3061.98 3090.35
3095.51 3098.20 3099.09 3100.43 3101.05 3101.79
3102.19 3102.66 3104.79 3124.01 3124.30 3125.28
3126.59 3127.84 3128.37 3130.22 3130.35 3135.50
3140.70 3143.17 3164.59 3165.67 3165.96 3166.58
3167.45 3178.31 3178.95 3180.14 3181.38 3185.06
3185.58 3187.79 3191.69 3192.00 3198.67 3205.31
3205.56 3207.06 3215.45 3219.01 3221.40 3222.26
3222.56 3223.09 3261.91

==============================
18B-TS
==============================

-142.63 10.25 16.71 21.10 28.27 30.75
33.00 36.35 41.32 44.54 47.89 48.52
51.12 56.65 59.67 59.87 61.27 66.07
69.87 74.74 77.17 79.19 87.10 87.64
92.89 94.59 98.63 102.75 107.51 110.08
111.63 113.02 122.01 130.51 132.41 141.20
141.86 153.49 162.74 164.78 169.48 172.60
177.11 179.96 184.46 187.88 189.52 196.95
197.15 200.18 202.03 203.72 214.54 219.82
221.87 228.58 231.77 236.73 242.12 245.73
250.81 257.22 266.32 270.08 278.18 282.67
284.94 287.45 296.30 300.35 304.28 315.68
321.61 341.57 343.31 362.86 378.37 391.82
400.45 408.14 414.79 419.16 423.80 430.04
446.08 451.45 458.34 464.14 466.34 481.62
485.13 487.00 496.84 501.99 513.01 518.61
519.69 520.64 523.26 525.27 527.95 529.57
532.95 533.14 535.09 538.11 542.48 546.12
546.49 551.63 555.20 559.33 561.80 565.97
571.50 572.80 575.72 595.71 616.59 623.84
636.43 655.93 681.48 690.24 696.92 699.03
708.34 710.56 712.93 715.33 721.47 748.73
764.57 768.65 784.61 790.69 796.51 805.09
813.53 830.04 837.65 839.58 846.58 850.54
854.02 862.30 868.96 872.50 874.51 876.33
879.83 881.18 881.83 887.62 890.35 890.79
894.53 907.11 915.79 921.60 927.32 929.47
935.70 941.64 944.56 946.20 952.20 956.90
962.76 964.06 966.91 967.39 969.67 969.86
973.49 987.53 991.98 992.17 1003.42 1005.70
1009.25 1011.60 1011.78 1012.79 1015.32 1023.16
1023.98 1024.75 1025.51 1026.20 1038.14 1046.57
1047.38 1047.97 1049.43 1052.14 1057.42 1059.59
1063.49 1064.50 1065.21 1065.90 1067.10 1067.75
1069.89 1069.97 1072.73 1080.66 1085.65 1123.67
1142.14 1156.11 1163.68 1165.71 1166.85 1169.33
1171.52 1174.53 1180.49 1188.29 1194.01 1201.13
1202.22 1205.05 1205.13 1206.58 1207.47 1214.85
1216.56 1219.79 1235.65 1239.40 1248.98 1258.71
1259.74 1262.31 1273.19 1285.63 1289.99 1291.89
1293.77 1295.31 1300.35 1307.26 1309.30 1317.59
1321.01 1321.36 1330.83 1332.79 1342.59 1354.67
1359.31 1360.35 1360.46 1365.88 1368.06 1387.77
1396.56 1408.36 1410.15 1417.83 1423.98 1425.56

1426.18 1426.67 1427.48 1429.34 1430.31 1431.26
1431.61 1454.39 1455.32 1461.31 1462.65 1464.19
1464.80 1465.16 1465.38 1466.34 1466.71 1469.28
1480.55 1484.73 1491.14 1496.43 1497.02 1497.69
1498.24 1499.28 1499.84 1500.22 1500.26 1502.52
1502.86 1503.94 1504.49 1504.58 1508.71 1509.50
1509.70 1514.94 1519.18 1519.86 1520.02 1524.71
1527.71 1551.45 1552.34 1598.75 1604.08 1630.29
1637.95 1638.49 1638.92 1642.29 1643.27 1645.62
1649.84 1650.59 1651.39 1652.71 1672.18 1674.43
1763.32 2990.12 3028.98 3033.50 3035.92 3036.06
3039.08 3041.45 3042.53 3042.62 3043.87 3044.22
3046.85 3047.56 3049.27 3064.57 3089.88 3096.59
3096.84 3100.20 3100.73 3101.59 3103.15 3105.46
3105.60 3113.66 3121.31 3122.41 3125.57 3129.05
3131.08 3131.19 3131.54 3131.69 3136.17 3137.91
3145.35 3147.38 3168.21 3169.30 3169.47 3169.55
3173.23 3173.61 3177.86 3178.07 3178.24 3179.39
3180.79 3182.38 3183.92 3185.17 3188.78 3191.23
3202.80 3206.44 3206.92 3207.89 3219.69 3224.79
3226.18 3235.63 3237.16

===============================

17

===============================

13.79 17.62 22.12 25.18 26.12 28.77
32.55 34.06 37.08 42.95 45.73 48.67
50.64 51.67 56.87 60.29 61.41 63.12
68.45 73.63 77.01 82.98 92.00 94.58
97.63 99.70 104.40 107.94 110.70 113.52
114.04 131.25 135.35 138.86 144.05 146.63
152.11 161.43 166.65 169.12 172.14 177.86
184.45 188.15 189.09 191.74 196.23 198.22
198.62 202.21 204.43 207.03 209.27 215.41
221.97 226.30 231.11 237.06 245.21 250.04
254.74 262.91 267.62 272.05 281.80 282.87
288.52 293.89 298.91 300.07 309.94 313.18
328.48 331.12 345.36 356.04 376.82 388.18
400.78 402.71 414.99 418.76 430.30 433.15
444.67 450.06 453.37 461.01 469.83 476.75
490.18 499.25 500.88 518.53 519.54 520.37
523.99 525.35 526.39 528.85 529.90 531.45
531.72 532.34 537.11 545.00 547.70 550.20
554.09 557.05 558.94 559.74 561.99 566.14
570.78 573.22 581.56 597.44 617.31 635.66
639.74 654.33 656.91 682.27 690.73 706.44
709.08 711.38 713.13 715.90 723.60 764.17
767.61 769.51 790.21 800.56 806.82 814.33
820.78 835.46 837.97 850.16 853.07 861.05
868.74 870.86 871.64 874.26 876.21 879.34
880.11 882.54 885.41 888.77 893.38 908.47
915.32 917.16 917.86 926.59 932.18 932.54
939.15 941.50 946.04 957.49 958.38 964.26
965.03 966.09 968.88 970.08 971.94 988.43
989.33 996.24 1000.03 1002.87 1006.11 1012.45
1012.75 1013.52 1015.32 1021.80 1023.38 1023.91
1024.53 1033.86 1044.53 1045.98 1047.26 1048.51
1049.36 1058.48 1060.06 1064.86 1065.32 1066.03
1066.84 1067.76 1068.97 1069.26 1076.01 1076.44
1077.44 1093.07 1097.79 1104.48 1116.18 1142.23
1155.26 1158.58 1161.38 1163.26 1167.90 1174.97
1182.02 1183.34 1189.38 1201.53 1204.18 1204.98
1205.17 1207.62 1210.54 1213.14 1216.48 1216.89
1224.79 1237.47 1244.00 1261.18 1262.52 1264.04
1271.81 1283.79 1286.35 1286.70 1289.13 1295.17
1296.10 1301.14 1320.78 1322.43 1324.42 1328.28
1331.88 1342.03 1347.59 1351.02 1359.41 1361.09
1363.66 1368.44 1369.05 1372.66 1391.89 1412.30
1413.27 1421.64 1424.23 1424.43 1425.61 1427.47
1429.60 1429.92 1430.28 1431.49 1433.57 1438.19
1455.20 1456.67 1458.70 1461.82 1463.33 1463.86
1464.76 1466.00 1467.09 1467.85 1469.36 1480.73
1482.71 1494.47 1494.63 1495.67 1496.65 1496.79
1497.91 1498.92 1499.95 1500.54 1501.18 1501.72
1502.71 1503.03 1505.70 1511.27 1512.33 1514.14
1514.79 1516.71 1520.44 1520.54 1523.55 1527.74
1533.88 1552.13 1552.93 1602.05 1609.52 1630.58
1636.95 1638.49 1638.92 1641.49 1643.16 1649.16
1651.78 1652.12 1654.45 1660.83 1675.39 1676.03
1782.07 2988.17 2994.87 3012.12 3032.44 3036.27
3038.20 3039.83 3040.24 3040.96 3042.22 3042.50
3044.18 3048.17 3067.54 3069.34 3098.14 3100.32
3100.50 3101.08 3101.52 3102.00 3109.14 3112.78
3113.17 3116.14 3120.17 3122.26 3125.61 3127.62
3129.03 3130.72 3131.71 3136.17 3146.29 3154.43
3164.74 3167.32 3170.11 3171.51 3172.92 3176.04
3179.34 3179.65 3182.07 3183.39 3184.03 3186.05
3188.00 3189.13 3189.99 3191.38 3191.92 3194.22
3206.82 3208.84 3218.69 3230.54 3230.83 3234.55
3237.55 3242.61 3245.43

===============================

19

===============================

11.27 17.99 25.43 29.55 32.85 38.13
39.89 41.00 43.79 46.76 47.72 51.31
55.85 56.34 57.44 58.46 62.93 65.01
66.73 73.06 76.38 81.34 88.61 94.23
94.79 99.40 102.58 111.08 112.50 120.71
133.28 136.76 138.46 141.21 146.90 157.25
162.35 162.62 164.04 165.10 171.09 177.62
186.42 187.19 188.86 191.03 193.96 197.87
202.21 207.49 209.41 216.93 217.60 223.56
224.68 226.83 231.09 235.37 247.37 252.33
260.07 264.32 268.01 280.62 285.95 288.25
297.17 298.52 301.29 303.36 320.02 323.91
335.10 346.35 367.46 383.58 394.25 395.58
399.36 413.75 415.89 429.65 433.19 439.19
443.57 447.91 452.40 463.07 471.63 474.75
485.82 496.56 498.97 513.53 516.88 518.73
520.36 521.95 524.13 527.90 529.25 532.84
534.55 535.52 536.85 544.20 546.19 547.65
549.90 556.27 559.32 563.03 570.20 571.55
580.02 586.04 593.61 609.17 618.83 637.83
639.08 656.17 680.07 684.90 690.78 708.66
708.81 711.54 717.09 719.41 721.29 767.53
768.79 788.28 789.69 792.05 804.17 816.79
823.03 838.64 840.75 852.25 854.42 862.76
866.88 872.08 876.41 877.32 880.82 883.65
887.15 893.26 893.46 894.89 898.41 917.74
919.29 921.93 922.77 926.16 936.31 939.54
940.50 943.05 945.70 957.72 964.13 964.64
965.44 968.06 968.49 972.77 974.48 980.52
988.98 991.38 992.02 1007.06 1007.93 1011.44

1013.35 1014.36 1014.82 1022.58 1023.90 1026.68
1027.58 1029.47 1043.78 1047.44 1048.28 1049.94
1050.24 1058.48 1061.09 1065.08 1065.88 1066.11
1067.36 1067.53 1068.23 1069.00 1072.62 1075.39
1076.17 1081.21 1104.37 1117.72 1135.25 1154.33
1161.03 1162.76 1167.74 1169.40 1171.55 1174.58
1179.69 1184.71 1188.30 1198.00 1201.32 1204.72
1205.95 1206.82 1207.09 1208.97 1211.85 1215.29
1227.31 1236.49 1240.77 1256.97 1258.35 1259.63
1271.22 1286.03 1287.92 1288.64 1289.04 1296.57
1297.88 1301.03 1320.67 1323.13 1323.25 1325.59
1328.46 1331.84 1335.58 1339.87 1349.40 1360.32
1361.63 1363.12 1365.34 1391.08 1395.63 1408.26
1408.57 1412.20 1420.44 1428.23 1428.99 1429.04
1429.23 1430.47 1431.25 1431.75 1432.36 1433.61
1434.45 1451.89 1458.16 1459.68 1462.43 1462.73
1463.59 1465.02 1466.26 1466.89 1467.55 1479.34
1487.18 1491.07 1493.20 1496.06 1497.07 1497.19
1497.66 1498.55 1499.58 1499.89 1501.88 1502.12
1502.48 1504.12 1505.34 1509.83 1510.41 1510.93
1511.17 1514.10 1514.59 1519.95 1522.69 1525.39
1528.93 1537.54 1548.85 1552.10 1592.29 1610.62
1633.29 1635.25 1635.41 1635.64 1642.00 1643.88
1648.25 1648.49 1650.13 1650.58 1671.70 1673.92
1796.43 2981.75 3000.01 3022.70 3034.03 3037.33
3038.69 3040.56 3040.91 3041.18 3041.66 3042.65
3043.50 3049.11 3073.96 3097.39 3098.09 3101.04
3101.18 3101.61 3101.81 3102.75 3104.53 3105.29
3112.11 3115.61 3127.66 3127.76 3128.45 3128.68
3131.30 3134.14 3134.60 3137.40 3151.14 3154.69
3163.90 3166.72 3166.77 3169.83 3171.97 3174.92
3180.47 3183.18 3183.33 3185.77 3186.02 3186.89
3187.51 3190.40 3193.60 3193.88 3206.76 3207.39
3208.06 3209.82 3212.82 3218.91 3222.77 3225.16
3227.47 3246.16 3254.06

==============================
20A
==============================

16.78 25.10 26.19 32.11 36.64 43.60
45.99 52.97 62.46 72.13 80.17 83.95
94.76 98.15 104.80 106.08 125.63 131.00
139.34 164.29 169.96 177.75 197.37 208.27
217.17 226.74 233.95 237.06 250.24 263.08
269.79 276.26 281.44 302.20 318.39 332.28
340.13 386.43 405.89 409.29 417.28 433.12
456.45 461.06 468.54 510.76 518.38 539.08
542.17 557.71 582.01 617.41 628.13 628.71
629.61 657.42 677.39 697.76 708.66 710.21
712.49 721.80 726.01 739.24 764.36 765.85
770.41 776.09 810.12 812.48 831.70 865.54
868.49 872.31 926.35 936.90 946.23 949.31
952.97 986.53 990.40 991.85 994.66 1007.54
1012.79 1013.33 1014.17 1016.58 1020.58 1023.26
1023.65 1044.36 1051.31 1052.38 1052.46 1052.53
1062.46 1075.70 1083.18 1114.57 1116.27 1116.35
1117.85 1119.02 1121.67 1123.93 1151.41 1173.80
1176.45 1204.11 1204.94 1205.39 1208.68 1212.52
1224.60 1228.48 1230.82 1232.34 1234.49 1255.00
1303.19 1324.61 1326.52 1329.77 1336.79 1349.80
1366.10 1367.60 1371.24 1396.84 1410.51 1461.92
1473.75 1474.21 1475.77 1476.52 1478.65 1480.76
1493.06 1500.62 1506.79 1524.86 1525.79 1527.91
1529.88 1623.81 1626.14 1628.51 1640.08 1643.06
1643.38 1687.57 1856.41 2264.45 2981.61 2998.84
3023.82 3034.82 3068.48 3070.96 3083.24 3110.62
3120.10 3121.24 3135.77 3148.00 3153.42 3171.52
3192.42 3192.56 3194.41 3199.44 3199.71 3203.10
3209.56 3209.98 3212.00 3214.00 3215.74 3216.39
3218.48 3220.02 3220.51

==============================
20B
==============================

3.79 16.65 19.56 34.54 35.72 40.28
47.84 53.26 55.74 58.57 71.13 76.53
90.43 94.88 99.15 101.46 107.78 113.27
117.27 158.65 161.39 178.66 191.37 202.37
218.06 226.06 232.91 243.48 251.88 255.96
265.16 269.37 276.42 295.69 312.80 332.82
339.16 380.01 407.41 410.73 417.08 437.51
453.41 457.69 468.45 507.78 518.79 535.77
540.03 560.52 580.35 616.30 627.15 627.87
628.77 655.52 676.95 695.91 708.22 708.64
711.66 719.50 723.23 739.50 765.81 767.21
769.03 780.19 788.80 815.79 827.43 865.78
867.09 872.45 930.34 934.42 947.16 948.29
953.21 985.22 990.28 992.42 995.23 1003.27
1008.83 1011.31 1014.47 1017.64 1021.98 1023.25
1023.49 1033.93 1047.94 1049.98 1050.63 1052.54
1059.05 1070.96 1082.86 1107.68 1114.65 1117.14
1117.44 1117.82 1120.19 1122.37 1150.29 1174.36
1179.46 1204.56 1205.15 1205.41 1209.27 1213.68
1225.73 1227.17 1227.56 1230.18 1247.49 1250.83
1297.59 1325.37 1327.34 1330.12 1336.42 1350.76
1367.53 1368.52 1370.68 1398.06 1414.95 1464.09
1468.75 1474.00 1476.84 1478.05 1479.96 1490.25
1494.76 1503.33 1509.08 1523.95 1526.15 1526.66
1529.76 1624.43 1625.14 1627.65 1641.67 1642.06
1643.23 1697.12 1848.11 2268.25 2985.05 2992.06
3032.39 3045.30 3060.28 3073.27 3079.16 3118.26
3118.53 3123.43 3133.42 3157.71 3164.08 3181.95
3189.60 3190.82 3194.21 3196.70 3196.71 3201.86
3205.67 3209.27 3211.32 3212.49 3215.55 3217.35
3217.62 3220.29 3227.91

==============================
20A-TS1
==============================

-299.82 13.07 22.47 25.76 35.35 37.56
38.15 42.85 47.90 49.70 60.21 68.05
73.82 79.88 92.23 113.50 123.72 135.40
141.62 161.22 171.99 180.12 190.77 197.84
214.31 228.99 235.05 241.89 250.40 255.15
268.34 280.17 284.40 292.62 318.51 342.42
363.73 407.68 407.88 417.48 431.10 444.44
447.59 460.62 486.39 505.60 518.73 534.92
543.58 576.17 606.10 629.55 629.95 631.16
637.58 663.36 679.28 697.44 708.72 711.02
713.70 722.15 723.61 748.49 763.22 765.01
770.14 771.61 799.38 834.66 865.81 867.59
874.02 905.50 942.45 944.80 944.93 948.35
949.97 975.91 986.86 989.35 992.23 1003.94
1012.18 1012.25 1012.92 1013.92 1018.35 1020.01
1021.10 1022.57 1051.71 1052.51 1052.55 1053.45

1058.54 1076.39 1082.10 1113.13 1114.26 1116.48
1117.49 1120.44 1120.67 1123.44 1142.27 1172.55
1175.25 1202.93 1203.46 1204.05 1209.57 1212.37
1223.64 1225.54 1226.80 1237.64 1250.28 1266.28
1286.00 1321.83 1324.67 1329.97 1335.53 1339.03
1365.01 1365.74 1367.73 1392.88 1406.95 1463.21
1473.02 1474.65 1476.91 1477.79 1479.36 1479.98
1489.71 1493.51 1503.70 1524.98 1526.05 1526.32
1528.23 1589.30 1624.23 1626.50 1627.73 1641.65
1642.31 1643.23 1817.78 1969.10 2988.15 2991.18
3030.29 3044.50 3066.55 3071.67 3078.77 3122.96
3132.52 3138.92 3151.70 3154.30 3156.73 3161.47
3183.79 3184.04 3189.15 3191.02 3191.38 3191.72
3198.60 3199.57 3199.85 3205.14 3206.67 3208.06
3215.86 3216.14 3217.10

==============================

20A-TS2

==============================

-218.71 17.41 24.48 31.09 37.77 40.59
43.96 47.10 51.05 52.83 55.52 64.50
69.57 87.87 96.09 108.72 124.31 140.50
150.39 158.79 165.81 194.62 200.43 205.94
211.90 223.12 231.20 246.74 251.27 259.83
267.02 275.95 278.55 295.47 335.20 356.94
406.48 410.20 411.41 415.79 440.78 449.15
459.30 470.65 488.48 508.04 521.17 535.02
557.54 600.25 615.77 626.82 629.64 629.80
668.29 684.13 688.31 696.98 708.86 710.20
711.26 720.00 725.33 761.27 765.30 766.09
769.09 773.74 808.92 818.84 856.62 867.11
868.83 873.56 942.03 944.84 949.95 954.26
973.26 988.88 990.11 992.56 993.75 997.26
1007.84 1008.67 1012.90 1013.79 1020.97 1021.69
1022.30 1037.68 1046.24 1048.85 1051.61 1052.46
1061.42 1076.10 1090.97 1107.77 1114.85 1116.21
1117.32 1118.62 1121.91 1122.57 1136.36 1172.61
1174.14 1203.74 1203.89 1204.49 1206.83 1210.06
1222.99 1224.01 1224.99 1226.64 1250.10 1263.38
1287.52 1328.43 1331.41 1332.07 1335.70 1345.60
1366.62 1366.83 1369.63 1391.35 1403.32 1465.35
1469.24 1475.66 1477.04 1478.69 1479.56 1480.11
1493.80 1502.58 1504.56 1524.53 1525.24 1525.39
1527.32 1625.88 1627.64 1628.42 1642.07 1642.32
1643.44 1643.64 1791.33 1826.89 2984.57 3003.51
3016.29 3047.18 3063.38 3073.81 3085.98 3123.23
3135.16 3140.32 3159.36 3166.20 3178.59 3187.18
3190.36 3192.71 3192.74 3192.85 3195.67 3200.82
3201.17 3203.79 3208.86 3209.24 3210.35 3216.46
3216.69 3217.22 3247.07

==============================

20B-TS1

==============================

-354.96 15.79 25.15 31.69 38.95 43.91
45.81 48.82 52.73 60.49 64.52 66.61
73.20 78.90 90.24 97.73 112.43 129.07
133.84 149.48 158.81 170.21 185.50 198.24
206.07 220.54 227.07 229.45 249.56 255.00
257.79 275.67 278.73 301.23 327.53 356.40
378.17 407.58 408.99 414.84 429.70 432.66
443.20 464.08 501.87 517.83 533.28 535.84
569.27 574.40 601.15 618.83 626.70 629.61
630.89 645.11 679.25 694.22 707.85 711.13
712.04 719.68 723.51 758.33 762.08 764.37
769.66 773.13 800.38 828.14 865.13 868.16
871.56 915.53 941.60 944.19 947.41 948.45
967.41 971.24 987.11 990.07 991.49 999.50
1011.07 1012.19 1012.95 1017.61 1019.36 1020.24
1021.44 1025.78 1048.28 1051.14 1052.73 1053.17
1057.87 1067.50 1085.92 1114.33 1114.88 1117.37
1118.32 1121.62 1121.95 1122.74 1141.12 1166.05
1174.20 1202.60 1202.96 1204.11 1209.12 1210.16
1222.48 1225.68 1228.36 1234.09 1267.53 1280.42
1285.35 1318.56 1323.85 1329.55 1334.69 1339.43
1363.53 1366.76 1368.83 1393.52 1406.90 1470.98
1473.43 1475.49 1476.05 1476.87 1479.37 1479.88
1490.72 1494.49 1501.82 1523.27 1525.62 1527.10
1528.40 1586.91 1624.26 1626.03 1627.77 1641.28
1641.78 1643.02 1790.23 1881.90 2991.63 2996.26
3012.30 3038.04 3061.06 3072.95 3084.58 3106.08
3124.58 3136.80 3154.20 3158.58 3158.97 3180.99
3184.92 3187.93 3191.51 3193.24 3194.71 3198.38
3199.42 3201.38 3203.81 3205.42 3207.40 3211.73
3216.06 3217.23 3218.49

==============================

20B-TS2

==============================

-217.25 10.19 24.42 26.49 32.65 36.21
45.67 50.71 54.20 59.35 65.17 68.30
76.43 84.20 88.08 113.34 120.02 127.85
152.67 163.59 196.09 199.70 206.32 208.30
215.31 223.65 228.53 242.66 249.20 265.75
271.25 274.58 292.75 311.04 323.66 347.50
369.77 405.22 408.58 413.02 430.96 436.68
453.73 462.73 481.41 509.48 517.85 531.90
539.60 556.94 614.24 627.82 629.82 630.14
668.02 687.09 697.67 707.99 708.50 709.94
721.16 726.01 729.65 765.17 765.47 769.38
769.78 790.72 806.90 816.28 860.53 865.35
869.16 874.77 941.30 942.84 948.77 957.99
974.21 976.55 987.69 992.12 996.27 997.31
1006.85 1011.24 1013.78 1014.82 1017.89 1020.93
1022.37 1041.88 1046.57 1048.89 1050.93 1052.17
1054.12 1071.96 1089.36 1108.55 1116.08 1116.41
1118.17 1120.11 1121.11 1122.91 1126.61 1155.36
1177.57 1203.00 1204.21 1204.57 1206.53 1212.51
1222.83 1224.47 1226.19 1228.65 1249.34 1279.98
1287.37 1328.09 1333.61 1333.99 1335.74 1349.70
1366.49 1369.34 1371.86 1392.84 1411.03 1470.64
1475.78 1479.36 1479.44 1479.86 1480.49 1493.18
1495.42 1502.31 1506.59 1525.24 1525.96 1527.19
1528.02 1626.08 1628.02 1628.55 1642.99 1643.74
1644.11 1649.97 1793.65 1867.23 2985.50 3009.92
3021.17 3037.66 3041.11 3069.68 3086.64 3113.85
3127.23 3131.93 3155.12 3164.17 3169.88 3190.21
3190.81 3192.27 3192.37 3192.94 3199.03 3200.83
3201.01 3207.56 3210.31 3210.82 3211.76 3215.62
3217.32 3218.71 3228.75

==============================

21A

==============================

22.88 24.13 36.40 41.38 42.26 48.65
56.63 59.17 63.95 69.08 81.55 84.09

91.47 103.13 107.40 120.70 129.81 142.69
155.72 171.04 184.04 194.58 200.73 211.32
216.74 223.50 249.87 257.13 261.83 276.14
278.62 301.24 314.65 337.89 371.76 405.87
406.32 412.31 414.33 431.19 445.35 458.47
467.91 490.41 504.82 521.71 527.17 540.87
578.77 619.63 627.95 628.49 630.41 640.62
696.26 708.03 708.69 710.03 712.84 725.20
728.37 757.88 758.98 764.25 767.58 769.21
790.26 804.21 866.34 869.22 872.68 905.57
918.44 945.68 949.51 951.46 952.41 959.78
965.36 989.95 993.10 994.27 1006.03 1009.12
1011.14 1013.36 1014.70 1020.83 1023.95 1024.49
1026.21 1051.01 1051.81 1052.48 1053.17 1068.74
1080.97 1112.51 1113.99 1115.74 1118.42 1120.10
1120.85 1121.48 1124.74 1148.08 1170.80 1178.78
1204.90 1205.08 1205.56 1216.96 1219.08 1225.78
1226.35 1228.48 1241.77 1263.45 1279.50 1287.56
1317.35 1325.55 1332.09 1335.89 1339.57 1362.64
1367.29 1368.47 1369.61 1404.65 1416.36 1473.76
1477.56 1479.47 1480.59 1483.47 1493.48 1495.43
1496.94 1503.36 1503.92 1523.27 1524.42 1525.53
1527.16 1598.01 1622.51 1627.29 1627.78 1639.33
1642.20 1643.62 1652.22 1781.56 2953.06 2983.68
3039.33 3056.81 3070.37 3071.42 3093.83 3116.25
3133.45 3135.67 3152.64 3168.56 3171.59 3180.77
3191.73 3192.53 3192.86 3194.43 3198.73 3201.19
3202.95 3206.52 3211.17 3212.53 3214.50 3218.53
3218.64 3220.94 3234.17

==============================

21B

==============================

16.41 19.00 23.44 37.11 40.88 45.11
46.03 49.83 56.98 62.31 73.62 82.72
93.23 102.08 111.48 124.71 130.81 144.61
155.29 159.20 186.28 195.37 201.21 216.76
219.67 231.32 246.99 260.76 273.44 276.91
291.29 298.52 317.14 340.10 369.28 395.99
404.65 408.22 414.19 433.52 448.83 459.00
467.79 495.34 508.38 519.85 530.76 541.56
608.25 624.73 627.54 628.45 629.81 636.52
681.07 698.28 708.88 710.48 711.81 718.75
724.26 728.13 757.75 763.28 766.00 767.06
784.59 789.80 849.97 865.42 868.84 872.38
925.97 944.34 950.60 950.83 952.40 961.49
971.12 989.33 991.86 993.18 1003.23 1011.93
1012.67 1013.69 1022.41 1023.22 1023.93 1024.63
1042.63 1051.24 1052.32 1052.43 1062.05 1070.50
1104.57 1115.11 1116.13 1117.90 1120.44 1120.52
1122.70 1123.57 1128.97 1152.35 1169.97 1177.41
1184.84 1205.12 1205.19 1205.80 1217.02 1224.17
1224.39 1225.85 1226.60 1258.48 1281.65 1288.52
1318.70 1327.06 1331.66 1336.91 1341.03 1363.82
1366.48 1366.78 1368.01 1405.26 1411.12 1463.29
1474.99 1478.02 1480.58 1480.69 1484.88 1489.26
1494.43 1503.09 1504.33 1524.15 1524.32 1524.94
1526.09 1603.65 1625.28 1626.62 1628.17 1640.98
1641.45 1641.83 1642.57 1800.27 2948.15 2981.46
3045.72 3058.39 3069.49 3072.92 3108.19 3115.69
3133.52 3142.99 3147.22 3152.17 3157.49 3167.24
3180.07 3187.04 3192.16 3193.05 3194.54 3198.03
3200.50 3203.08 3206.12 3208.73 3211.53 3213.23
3217.35 3219.29 3219.76

==============================

23A

==============================

14.15 22.03 24.76 32.39 39.83 46.87
51.26 54.21 59.21 63.97 79.52 86.04
88.63 107.51 122.57 139.89 147.86 164.88
186.90 194.25 206.14 211.53 219.10 228.82
234.94 246.39 250.67 256.23 257.38 258.14
279.90 295.92 305.41 323.07 350.63 364.64
404.43 404.55 420.32 430.92 440.81 447.29
469.21 497.18 514.43 520.45 541.64 567.24
620.93 626.24 628.22 629.58 630.63 658.81
685.27 687.82 699.85 704.52 707.10 714.29
721.34 727.63 753.49 762.10 765.80 768.25
774.04 795.16 837.15 858.99 865.47 879.14
904.06 942.36 948.80 956.69 962.61 970.92
979.03 988.40 991.07 1000.17 1003.86 1009.89
1012.32 1014.46 1016.68 1023.62 1023.79 1026.73
1035.57 1047.95 1052.58 1053.43 1055.45 1084.24
1088.32 1110.97 1113.66 1116.43 1118.24 1119.50
1121.34 1124.51 1130.74 1161.97 1177.87 1205.81
1206.17 1206.17 1210.38 1214.01 1225.07 1227.06
1230.72 1232.58 1248.63 1263.61 1284.69 1290.04
1328.89 1330.49 1339.85 1346.74 1352.98 1366.72
1368.92 1372.20 1394.44 1405.63 1412.10 1438.17
1468.82 1475.85 1476.07 1480.10 1481.90 1493.92
1494.71 1504.48 1505.23 1524.50 1525.17 1527.57
1529.99 1625.35 1626.86 1629.00 1641.20 1641.53
1643.41 1650.95 1670.04 1781.78 2939.87 2966.82
2972.81 3032.05 3068.38 3075.50 3093.17 3095.16
3104.13 3127.68 3134.78 3147.15 3150.68 3172.48
3184.50 3191.23 3191.31 3197.03 3197.26 3197.98
3204.89 3205.73 3206.30 3212.26 3213.89 3217.14
3219.89 3220.15 3227.33

==============================

23B

==============================

6.17 22.36 23.22 36.78 44.56 47.98
56.60 62.98 75.43 79.39 86.24 97.52
106.47 114.29 131.70 145.82 155.90 161.83
185.31 193.54 201.70 207.07 215.15 220.10
235.34 239.90 250.66 262.01 268.89 273.80
285.95 298.77 303.76 330.15 345.09 396.95
410.19 414.81 418.70 424.50 432.68 462.44
464.31 486.16 508.75 517.22 543.18 584.16
589.34 627.39 628.75 630.23 630.97 657.21
691.76 697.98 707.71 710.26 712.58 722.30
726.51 763.99 767.23 768.29 769.49 777.70
791.58 806.59 863.89 868.87 872.04 879.33
904.60 947.05 954.29 957.21 964.33 965.80
989.18 993.19 994.81 1003.14 1008.48 1013.32
1013.83 1015.46 1021.36 1022.13 1022.60 1026.67
1030.89 1051.74 1052.59 1054.46 1055.55 1090.02
1094.68 1111.14 1112.02 1112.93 1118.08 1119.34
1123.59 1123.68 1131.54 1175.00 1205.32 1205.35
1206.04 1206.10 1209.99 1212.50 1228.55 1230.68
1232.75 1234.27 1251.05 1258.67 1289.09 1325.70
1334.03 1336.67 1343.31 1347.36 1368.84 1370.22
1371.07 1372.46 1384.92 1407.63 1414.06 1473.59

1478.25 1480.33 1481.23 1485.24 1494.92 1497.66
1501.85 1505.09 1507.82 1524.19 1525.76 1528.00
1531.10 1623.79 1625.72 1628.04 1638.98 1641.05
1642.03 1644.21 1669.46 1679.37 2967.16 2986.06
3017.38 3033.19 3055.37 3063.78 3076.45 3082.32
3084.39 3113.31 3118.09 3165.82 3193.32 3193.79
3195.74 3197.64 3199.53 3201.44 3202.99 3203.29
3210.10 3212.19 3213.40 3217.53 3219.29 3223.96
3226.07 3227.94 3228.22

==============================

21A-TS

==============================

-367.25 22.50 27.39 34.27 38.93 47.42
48.31 51.59 62.23 65.55 76.66 78.81
88.22 93.28 103.24 114.83 122.68 139.09
150.63 159.91 186.89 195.82 204.91 211.49
220.35 226.91 248.72 252.72 257.91 278.18
285.97 291.00 312.04 342.30 357.97 389.34
407.32 410.15 415.28 425.33 439.51 454.40
459.51 468.27 493.91 501.37 519.03 532.38
544.41 572.77 624.00 625.09 627.08 630.10
686.43 706.44 709.22 710.79 712.26 719.87
730.28 732.32 757.05 765.59 767.38 779.56
792.94 817.37 855.06 867.13 875.44 885.25
922.63 925.53 948.13 950.37 955.37 962.11
986.18 987.28 992.27 997.80 1003.63 1004.71
1010.03 1010.64 1013.41 1022.03 1023.64 1025.34
1032.24 1046.91 1048.31 1052.74 1054.14 1078.00
1085.61 1094.19 1112.72 1114.23 1114.78 1118.59
1121.06 1125.49 1133.96 1147.31 1179.39 1184.46
1204.23 1204.38 1205.31 1216.54 1218.39 1219.53
1219.68 1227.14 1250.10 1254.22 1264.86 1290.56
1317.97 1321.38 1327.54 1335.06 1341.73 1363.22
1365.51 1368.64 1369.56 1411.67 1414.67 1465.55
1471.26 1475.76 1479.15 1479.50 1481.69 1495.61
1502.91 1503.38 1506.39 1509.86 1522.48 1523.28
1526.09 1537.09 1601.21 1609.91 1625.83 1627.53
1633.66 1641.08 1643.11 1784.51 2967.41 3000.32
3038.03 3038.39 3065.69 3075.17 3091.34 3129.28
3131.63 3148.82 3150.98 3152.31 3183.51 3184.89
3187.17 3193.42 3194.81 3195.23 3195.66 3196.12
3202.39 3204.25 3204.94 3212.59 3213.42 3214.09
3219.03 3219.49 3221.55

==============================

21B-TS

==============================

-353.80 19.62 23.30 25.00 36.03 42.34
52.22 52.67 58.06 64.98 69.65 81.73
97.25 104.62 119.60 125.83 135.95 155.64
161.25 186.69 195.63 201.98 213.11 216.15
221.72 232.88 251.35 257.28 263.79 274.02
278.10 299.18 318.04 328.51 360.92 384.57
406.30 407.97 414.65 422.78 430.58 448.91
453.87 467.87 472.30 499.14 516.37 530.55
569.38 606.67 614.25 625.06 627.57 629.21
679.39 687.16 706.03 708.77 711.07 728.70
730.84 732.26 738.10 758.99 765.01 765.99
785.30 803.86 846.34 859.34 864.58 873.86
929.80 937.92 945.33 953.45 954.70 971.76
987.77 988.04 988.35 996.09 998.73 1004.92
1012.13 1012.93 1022.19 1023.21 1026.06 1028.37
1036.66 1047.50 1049.38 1052.90 1070.42 1084.30
1095.30 1098.39 1111.78 1114.52 1118.40 1120.38
1120.63 1127.04 1134.56 1160.80 1177.78 1186.71
1194.61 1203.87 1205.19 1205.90 1219.54 1219.61
1220.16 1225.30 1230.40 1251.32 1290.05 1296.09
1320.16 1323.39 1330.16 1335.27 1341.11 1363.25
1367.33 1369.13 1372.69 1411.24 1414.65 1466.45
1472.31 1475.34 1476.96 1479.20 1481.41 1487.21
1494.35 1504.63 1508.18 1511.10 1522.45 1523.19
1524.84 1529.36 1596.75 1602.44 1625.76 1627.02
1634.89 1641.74 1642.40 1790.63 2966.17 3001.06
3040.49 3050.75 3068.74 3070.65 3117.10 3121.06
3128.01 3142.28 3150.89 3161.76 3173.97 3184.84
3185.92 3186.97 3188.25 3194.59 3196.11 3196.97
3202.89 3204.19 3204.46 3211.70 3211.94 3212.59
3218.33 3219.18 3219.52

==============================

23A-TS

==============================

-338.11 17.31 22.09 25.34 38.05 41.28
47.48 49.40 50.16 57.72 64.80 75.10
80.80 88.97 115.72 127.82 133.43 142.89
154.21 176.90 188.61 194.05 209.02 215.04
222.41 234.34 239.56 251.13 267.56 270.36
295.21 298.57 318.67 334.01 347.68 368.29
404.58 408.10 414.31 421.16 426.49 457.26
464.65 470.88 501.77 520.48 540.61 545.96
591.00 623.95 624.70 627.80 628.33 647.03
689.26 691.19 705.07 706.79 707.77 717.34
731.22 732.27 753.96 762.94 764.91 768.35
779.62 826.43 839.75 864.97 866.17 871.50
916.12 945.65 945.97 951.54 956.29 967.43
987.07 988.56 993.15 996.42 1008.01 1011.94
1012.52 1019.81 1021.95 1023.38 1024.93 1028.49
1039.04 1050.13 1051.23 1051.72 1063.61 1068.21
1087.35 1105.43 1108.89 1112.32 1115.12 1116.39
1117.87 1119.79 1128.25 1156.30 1178.13 1186.71
1204.67 1205.35 1206.78 1208.24 1218.13 1219.60
1223.29 1223.84 1225.82 1262.69 1272.58 1282.17
1317.96 1324.17 1333.46 1334.27 1338.98 1365.61
1366.70 1370.91 1373.00 1396.44 1422.33 1468.03
1469.95 1473.22 1477.53 1478.02 1480.43 1492.68
1494.11 1504.17 1505.66 1515.99 1521.55 1522.15
1524.24 1584.74 1612.52 1623.93 1626.14 1634.35
1639.14 1642.77 1652.14 1778.82 2750.25 2968.33
2993.88 3041.00 3067.15 3069.49 3082.61 3123.47
3128.40 3141.68 3147.83 3149.33 3179.57 3181.93
3188.84 3189.32 3193.74 3195.56 3198.33 3202.31
3203.91 3206.18 3209.50 3211.48 3213.63 3213.96
3218.31 3219.25 3220.62

==============================

23B-TS

==============================

-355.22 22.08 31.10 36.17 44.42 48.79
50.10 54.27 61.06 72.26 76.79 82.49
90.70 98.64 101.99 111.88 126.14 147.09
156.43 166.07 178.05 196.39 204.42 209.41
217.12 232.34 233.28 248.58 256.74 269.38
271.00 305.94 310.36 329.38 339.34 399.37
407.42 408.03 414.98 424.62 443.00 459.47
467.41 475.81 501.55 520.00 541.52 545.97

564.85 611.76 624.40 627.75 628.67 663.35
690.05 691.67 704.89 707.36 710.05 724.85
731.98 732.89 762.33 768.35 769.83 773.53
794.01 843.93 863.34 867.99 870.45 877.97
895.93 935.93 945.40 946.47 952.33 961.79
966.96 991.21 994.81 1003.60 1007.52 1008.99
1011.62 1013.32 1013.55 1022.15 1027.69 1028.47
1038.29 1048.97 1050.20 1051.99 1053.01 1065.50
1069.24 1105.15 1109.14 1112.79 1116.33 1117.65
1118.42 1121.41 1126.26 1178.87 1188.32 1193.07
1204.72 1205.84 1206.94 1211.47 1213.99 1219.34
1223.46 1225.18 1229.17 1265.84 1273.31 1294.96
1321.13 1323.77 1333.95 1334.65 1340.12 1366.50
1370.49 1372.78 1379.97 1397.83 1421.45 1462.94
1469.50 1478.08 1478.92 1479.27 1485.02 1493.43
1502.51 1505.92 1507.15 1515.44 1522.64 1522.81
1525.84 1604.60 1611.17 1624.94 1625.46 1635.06
1641.09 1642.73 1650.75 1760.57 2743.36 2965.65
2987.43 3015.36 3033.46 3059.11 3074.10 3082.97
3122.22 3126.82 3161.18 3186.06 3187.38 3189.02
3191.54 3195.53 3197.09 3198.34 3198.86 3204.13
3206.25 3207.16 3211.90 3214.93 3215.80 3218.58
3220.48 3225.19 3248.42

==============================
22
==============================

18.82 23.71 29.14 38.79 44.22 47.96
50.50 59.63 62.96 69.58 75.93 83.53
103.15 118.80 126.42 132.00 151.86 157.44
173.22 184.85 192.37 198.39 204.74 212.29
215.43 229.72 247.36 262.63 273.70 294.59
303.04 325.30 339.60 370.89 403.43 407.24
409.70 414.25 423.75 433.22 448.50 455.79
469.22 499.05 510.91 522.87 528.85 578.20
595.33 624.13 627.04 628.18 629.65 656.64
682.21 705.15 708.45 709.45 722.30 731.36
735.93 762.12 765.57 770.29 778.51 811.63
817.54 863.73 868.29 876.32 900.80 911.92
936.01 942.17 944.80 954.77 957.36 987.94
992.41 993.88 998.66 1002.14 1003.17 1012.17
1013.66 1022.58 1024.72 1026.06 1026.14 1048.46
1051.63 1052.67 1053.00 1070.97 1083.91 1094.80
1096.80 1107.07 1111.02 1115.15 1116.66 1118.18
1122.42 1130.65 1180.04 1183.62 1204.30 1205.19
1206.66 1212.07 1215.45 1220.74 1221.37 1222.85
1224.81 1260.39 1266.62 1285.09 1293.72 1317.40
1322.58 1333.25 1334.72 1347.54 1355.91 1366.11
1369.66 1370.31 1375.41 1417.83 1420.53 1435.49
1466.53 1479.02 1479.59 1480.97 1487.84 1495.66
1498.57 1501.69 1505.12 1510.69 1512.84 1523.56
1525.63 1529.83 1593.74 1602.87 1626.14 1626.82
1629.92 1641.92 1642.37 1775.08 2969.49 3009.47
3018.08 3037.96 3044.75 3055.16 3068.79 3108.02
3108.69 3116.08 3155.08 3181.52 3183.19 3184.70
3185.63 3186.53 3190.71 3192.28 3194.80 3197.61
3198.64 3201.00 3204.76 3206.08 3206.20 3208.67
3213.87 3214.36 3217.39

==============================
24
==============================

17.88 23.41 34.37 40.29 44.99 48.61
54.65 57.97 65.44 79.01 79.22 82.86
101.43 121.23 131.38 146.77 159.39 164.82
182.36 190.32 197.64 199.24 207.54 217.60
219.90 231.36 249.17 260.77 271.44 296.08
310.51 315.06 339.35 374.72 402.01 407.81
410.23 410.89 416.67 423.12 448.18 457.30
472.42 487.52 499.11 513.31 529.96 593.60
595.50 623.87 627.52 629.22 640.74 679.66
688.99 707.22 709.48 710.92 727.70 728.77
735.23 761.70 766.07 769.36 782.04 788.77
831.19 866.74 873.45 876.60 888.36 908.14
923.79 936.41 946.11 955.14 957.64 981.75
991.09 996.71 998.37 1003.20 1003.60 1012.08
1013.58 1022.08 1023.70 1025.54 1025.97 1044.70
1048.65 1051.17 1053.23 1078.91 1089.04 1091.95
1103.99 1110.48 1112.60 1114.17 1117.98 1120.97
1130.17 1167.90 1177.59 1182.90 1204.40 1204.60
1204.90 1209.22 1210.34 1221.65 1222.57 1223.06
1225.31 1244.06 1264.85 1289.47 1299.05 1318.80
1324.97 1332.81 1335.65 1347.73 1362.82 1367.66
1368.06 1370.34 1377.12 1419.27 1429.33 1442.53
1464.48 1477.47 1479.77 1481.64 1484.23 1494.53
1502.91 1507.10 1508.28 1510.95 1511.92 1524.24
1525.28 1531.63 1596.24 1602.62 1626.69 1627.19
1628.21 1642.07 1642.94 1795.03 2982.41 3016.65
3017.54 3030.08 3038.16 3048.07 3065.86 3069.87
3111.58 3115.01 3153.45 3180.21 3185.97 3189.97
3190.23 3192.43 3195.04 3199.30 3199.43 3202.60
3205.00 3207.63 3210.37 3210.79 3213.01 3213.76
3215.85 3218.41 3221.89

==============================
25
==============================

26.69 42.70 71.98 77.02 100.94 114.35
128.52 141.93 155.16 160.96 166.01 171.99
181.25 185.58 190.65 200.22 201.47 215.98
221.78 225.92 232.13 241.80 257.70 262.21
273.59 278.18 283.90 295.94 331.55 344.07
346.93 349.31 380.28 417.37 473.95 597.31
654.13 658.06 665.63 709.99 716.70 719.83
721.67 723.17 727.33 807.85 812.42 865.06
867.41 869.38 876.44 894.54 907.38 941.32
960.78 971.75 971.98 974.38 975.14 975.50
978.31 995.56 999.32 1030.43 1118.53 1187.39
1261.22 1292.28 1319.91 1338.78 1342.50 1344.82
1346.38 1364.16 1370.83 1374.22 1396.86 1452.96
1461.72 1463.06 1468.93 1469.58 1473.03 1475.62
1479.27 1480.64 1483.74 1486.64 1487.07 1493.71
1495.80 1502.99 1599.78 2057.94 2993.84 3035.56
3061.47 3062.27 3062.40 3062.68 3063.70 3066.32
3099.39 3100.49 3143.45 3144.34 3145.56 3146.64
3146.86 3150.10 3160.13 3163.74 3165.53 3167.12
3171.48 3172.89 3174.36 3174.88 3268.10 3399.51

==============================
26
==============================

19.47 24.26 26.87 46.38 59.75 69.86
71.24 83.99 86.36 114.13 115.38 119.28
135.49 140.74 146.18 151.29 163.60 170.90
173.40 176.96 184.82 191.97 194.58 207.77
212.34 218.02 229.42 235.57 242.24 259.67

263.87 267.67 277.01 280.12 283.16 305.72
332.81 346.48 349.34 355.74 366.10 386.62
434.25 528.74 651.62 654.63 658.11 671.81
674.29 703.38 717.41 720.69 721.91 724.62
727.54 810.26 816.71 866.12 868.87 871.68
882.26 923.25 928.23 950.56 972.74 976.65
977.87 979.20 982.80 986.44 990.25 998.75
1016.71 1027.66 1032.94 1035.58 1061.87 1119.52
1182.35 1247.72 1271.98 1320.79 1337.57 1340.90
1342.08 1344.38 1362.09 1368.28 1369.98 1417.60
1426.93 1461.72 1462.98 1469.37 1471.78 1473.73
1474.33 1474.66 1476.75 1478.79 1480.95 1481.79
1484.46 1487.16 1496.07 1500.51 1503.72 1524.57
1730.21 2071.53 2104.01 2973.21 2982.48 3002.94
3033.56 3046.25 3058.33 3058.89 3060.14 3060.64
3061.66 3062.22 3116.14 3122.75 3140.41 3142.04
3142.89 3143.90 3144.82 3150.61 3154.68 3157.59
3159.43 3162.78 3164.01 3168.20 3172.28 3173.18
3245.72 3393.57 3408.68

==============================

26A

==============================

20.74 24.90 33.87 46.02 57.67 68.80
79.01 86.45 89.71 112.19 115.08 124.19
139.31 144.40 149.59 155.72 159.90 165.93
177.18 178.57 186.86 191.33 193.07 204.28
208.95 215.51 224.86 227.60 232.68 247.61
260.04 261.77 272.46 275.32 283.39 322.11
333.12 342.64 348.71 354.20 369.80 387.20
429.77 515.37 642.13 655.13 658.44 662.36
666.20 684.40 718.83 720.64 722.09 724.56
734.45 809.02 815.35 865.28 867.83 872.03
881.90 919.21 929.00 955.29 971.54 975.14
976.56 978.30 980.32 986.40 993.08 999.11
1013.49 1025.81 1031.44 1043.69 1074.76 1122.20
1184.30 1252.08 1272.41 1321.82 1338.30 1341.18
1342.43 1345.43 1362.78 1368.66 1369.49 1419.33
1426.14 1461.75 1462.24 1468.93 1471.84 1474.04
1474.46 1475.59 1477.41 1479.96 1480.69 1483.89
1486.71 1492.79 1496.75 1500.04 1505.38 1525.01
1730.61 2064.31 2111.62 2974.67 2980.73 3006.60
3032.61 3043.25 3058.08 3059.65 3060.25 3060.33
3061.38 3061.95 3112.41 3124.63 3141.23 3141.84
3144.22 3144.50 3145.03 3149.33 3155.95 3157.66
3160.92 3162.95 3164.73 3165.89 3168.16 3169.30
3246.62 3398.16 3432.86

==============================

26-TS

==============================

-398.41 23.55 33.64 36.90 49.65 54.55
70.64 74.74 79.38 92.82 108.47 120.08
138.91 146.44 147.85 153.04 157.93 166.89
179.28 180.57 188.24 196.40 202.11 204.44
215.80 228.21 239.36 253.18 254.15 258.03
262.79 268.26 273.48 305.07 326.18 335.58
345.74 360.45 390.65 420.51 450.85 504.69
515.62 546.33 655.53 660.15 667.85 692.03
704.34 722.67 725.39 727.13 727.60 812.14
814.35 858.96 863.60 868.80 873.68 877.57
901.02 922.05 938.17 957.51 973.39 976.99
979.57 981.85 983.22 985.75 992.35 998.30
1004.91 1026.34 1029.43 1037.49 1057.74 1127.79
1183.23 1247.70 1273.84 1321.50 1336.83 1339.80
1341.55 1342.05 1361.26 1363.04 1364.53 1411.56
1420.93 1461.14 1463.21 1470.38 1471.15 1471.65
1474.74 1475.70 1476.21 1478.81 1479.60 1482.93
1486.43 1489.33 1490.01 1496.76 1499.49 1523.61
1729.54 1773.84 1842.88 2957.03 2981.88 2989.78
3034.23 3050.75 3054.06 3056.26 3056.55 3057.46
3057.91 3061.99 3124.88 3131.53 3139.70 3140.03
3141.31 3144.08 3145.91 3145.98 3151.06 3153.79
3154.85 3155.28 3159.34 3161.08 3162.79 3167.58
3246.13 3286.93 3292.50

==============================

26A-TS

==============================

-366.10 17.05 30.84 35.47 48.71 54.61
63.58 76.12 78.05 93.03 104.83 117.44
127.35 141.03 144.84 150.55 156.37 167.44
176.75 182.71 189.75 194.61 198.70 205.00
209.43 223.89 225.62 240.53 256.48 261.15
263.80 267.46 273.15 322.98 329.66 337.22
350.49 369.29 390.72 425.77 448.64 498.75
504.53 555.21 654.53 658.13 660.44 675.00
686.57 721.93 724.85 726.93 727.50 812.08
814.38 863.75 866.36 872.91 876.05 893.98
935.17 946.83 965.83 973.58 977.55 978.97
981.47 983.08 984.02 987.15 996.30 999.46
1018.18 1026.71 1035.40 1038.29 1065.50 1126.72
1182.89 1247.96 1274.20 1322.03 1335.94 1339.36
1340.96 1343.40 1360.60 1364.11 1364.81 1412.76
1421.84 1461.79 1462.97 1470.46 1471.73 1472.08
1473.82 1474.52 1479.13 1479.76 1481.87 1483.70
1486.50 1490.09 1492.53 1495.87 1501.91 1523.77
1730.04 1764.73 1831.51 2959.08 2982.80 2991.66
3035.73 3046.08 3054.00 3056.98 3057.59 3057.84
3059.97 3063.00 3110.45 3121.82 3139.22 3139.98
3141.19 3143.65 3147.05 3148.01 3152.55 3154.86
3155.22 3159.65 3160.56 3162.68 3165.46 3166.80
3246.52 3293.24 3298.63

==============================

27

==============================

14.47 25.73 38.19 47.45 58.47 64.04
70.18 75.83 86.55 105.49 129.80 134.18
145.26 161.73 162.89 169.93 179.86 183.62
187.79 193.40 196.38 201.32 205.01 215.77
222.76 227.78 232.19 246.35 248.77 252.96
258.40 259.76 322.30 325.77 331.54 365.87
377.69 435.43 470.68 545.55 571.24 596.52
654.33 656.76 670.25 687.20 726.45 729.29
733.62 733.88 738.05 812.82 815.90 848.27
860.26 865.53 872.33 876.99 919.81 939.20
977.35 979.80 981.42 982.11 983.70 984.06
986.38 993.32 997.31 1011.65 1023.01 1031.42
1054.25 1086.75 1106.00 1139.80 1181.59 1205.72
1238.22 1271.54 1322.01 1337.83 1340.14 1341.29
1342.27 1343.73 1361.06 1364.32 1370.47 1421.99
1425.42 1460.48 1460.91 1468.54 1468.90 1471.71
1472.36 1473.47 1476.50 1477.40 1480.25 1481.47
1488.69 1490.92 1494.15 1496.36 1501.04 1523.44
1600.79 1668.20 1727.58 2946.07 2985.30 2986.47

3026.36 3036.90 3053.56 3055.40 3056.06 3056.33
3063.41 3065.36 3092.04 3129.91 3137.04 3139.77
3140.81 3143.12 3146.65 3149.25 3150.68 3151.70
3152.91 3154.47 3155.84 3157.64 3158.77 3162.60
3164.12 3172.11 3246.05
===============================
28
===============================
15.80 25.33 39.59 45.49 62.75 67.44
79.34 89.66 100.10 108.83 127.07 137.00
142.90 153.15 160.03 164.69 169.04 176.85
182.40 192.86 196.69 203.66 206.56 216.02
224.63 227.18 239.54 248.92 252.27 258.92
261.97 275.83 321.00 325.92 331.91 392.47
397.65 439.24 485.68 504.16 530.53 549.09
654.57 656.79 668.35 706.33 724.74 727.98
733.32 735.69 810.24 813.05 815.88 861.15
865.47 871.19 872.71 877.31 932.19 945.29
976.98 978.58 980.90 982.15 982.97 983.79
986.79 995.03 997.50 1017.29 1031.59 1047.97
1049.18 1095.65 1108.90 1134.41 1181.85 1225.10
1258.91 1271.80 1317.44 1321.73 1339.07 1340.55
1342.59 1343.68 1361.59 1364.21 1366.58 1413.70
1420.18 1459.41 1461.40 1465.27 1468.15 1469.92
1472.72 1473.41 1473.98 1475.73 1478.03 1480.81
1482.40 1487.76 1492.42 1496.77 1506.09 1523.86
1626.64 1669.59 1727.40 2947.61 2986.25 2989.85
3021.43 3028.17 3053.20 3054.24 3055.91 3056.15
3061.87 3063.22 3079.40 3091.20 3137.18 3138.67
3139.90 3141.40 3150.85 3151.34 3151.66 3152.56
3153.53 3153.59 3155.31 3155.61 3162.06 3163.88
3165.47 3168.75 3244.48
===============================
8A-TS1’
===============================
-134.01 11.08 21.84 24.56 29.58 34.02
37.47 44.25 47.10 50.27 53.85 57.29
60.55 64.08 65.46 68.73 70.76 72.98
73.65 79.02 83.83 86.98 88.91 94.05
96.65 104.27 115.19 121.19 127.64 134.13
145.23 149.37 157.66 168.02 177.72 188.25
206.98 208.69 211.94 219.27 221.52 228.58
230.99 236.51 253.60 254.75 258.26 259.85
267.30 267.86 275.07 277.05 281.60 303.31
327.51 336.87 365.72 392.18 406.74 408.56
410.44 414.72 418.64 421.63 423.53 426.15
433.52 435.12 438.59 467.59 470.22 498.70
501.71 503.33 516.73 520.83 530.34 533.98
535.06 549.62 560.61 584.09 618.39 625.78
627.41 629.25 629.64 630.52 631.04 632.38
632.71 674.19 683.05 690.86 694.30 702.55
705.23 706.71 708.35 710.47 710.89 713.73
714.54 717.74 719.68 720.94 749.81 758.29
761.41 765.38 766.47 767.68 768.98 771.47
777.74 798.23 820.55 859.69 861.24 863.18
867.56 869.87 872.67 880.89 881.20 904.26
937.37 940.18 940.71 943.47 944.96 948.36
956.90 961.08 981.26 983.17 983.76 988.22
988.76 989.78 992.33 1002.31 1007.15 1010.80
1011.12 1011.92 1012.26 1012.86 1013.15 1014.66
1015.02 1015.08 1016.48 1017.42 1019.28 1020.16
1021.67 1022.52 1026.48 1047.07 1051.94 1052.50
1053.07 1054.05 1055.03 1057.27 1062.93 1081.06
1107.43 1109.69 1110.56 1113.97 1115.70 1116.11
1117.85 1118.49 1119.02 1120.30 1120.41 1120.76
1122.08 1124.91 1141.03 1167.06 1180.74 1196.18
1199.62 1201.82 1201.94 1202.18 1203.07 1203.18
1203.22 1210.84 1219.12 1221.63 1225.34 1226.57
1228.44 1229.88 1230.31 1235.86 1236.93 1260.28
1283.02 1291.61 1323.75 1323.79 1327.79 1328.82
1331.35 1335.25 1336.28 1337.11 1351.59 1362.93
1365.36 1366.71 1367.74 1368.63 1368.67 1374.29
1396.60 1473.25 1474.39 1475.45 1475.63 1479.10
1479.82 1480.47 1481.50 1486.28 1495.58 1505.76
1506.06 1523.98 1526.10 1526.60 1527.41 1527.54
1527.91 1528.21 1531.42 1620.25 1624.50 1626.38
1627.42 1628.56 1628.68 1630.86 1642.85 1643.76
1644.57 1645.20 1645.66 1647.18 1647.45 1659.37
1797.11 1975.27 2979.67 3020.27 3028.99 3056.20
3068.57 3102.40 3118.58 3152.51 3153.88 3158.31
3162.01 3183.51 3183.57 3183.70 3186.28 3186.48
3187.34 3187.51 3188.60 3189.74 3191.99 3193.54
3194.20 3194.30 3194.57 3196.32 3198.82 3199.38
3201.78 3204.31 3205.45 3206.30 3208.93 3209.58
3211.07 3212.55 3212.83 3213.73 3217.18 3224.89
3226.32 3228.03 3235.15 3240.96 3251.46 3268.52
===============================
8B-TS1’
===============================
-128.62 5.02 11.65 18.07 23.38 31.38
36.49 41.14 43.62 46.83 51.09 53.01
55.96 57.02 61.09 63.69 65.42 70.23
74.77 83.25 85.95 91.16 95.03 98.86
104.33 108.53 111.53 120.50 128.36 137.19
140.27 143.18 152.42 162.58 172.51 194.54
199.75 206.80 217.79 221.65 226.21 229.28
232.95 240.99 247.65 253.33 256.04 258.30
263.67 266.58 279.10 281.23 284.18 290.30
306.07 358.46 366.26 405.15 406.42 409.44
412.23 414.68 421.67 422.61 424.33 428.50
435.10 435.91 439.79 464.90 468.01 501.90
506.54 516.13 518.76 528.66 533.04 535.53
539.29 567.79 574.24 601.89 613.77 628.28
629.69 629.94 631.22 631.72 632.22 633.80
641.18 675.30 683.99 695.29 696.93 700.84
705.64 707.11 708.48 711.65 712.04 713.47
716.18 718.21 723.05 723.82 739.51 758.29
759.31 761.22 763.70 764.68 766.99 768.37
779.93 799.12 815.74 856.21 859.28 862.83
864.31 867.58 875.06 876.21 888.79 910.85
936.65 939.68 941.31 943.53 944.60 946.56
950.74 954.01 979.32 980.93 981.34 982.90
984.87 987.57 992.07 995.59 1007.31 1008.55
1009.92 1012.05 1012.63 1012.89 1013.24 1013.78
1015.19 1015.77 1016.92 1017.00 1017.45 1018.05
1019.51 1020.77 1029.06 1051.51 1052.27 1053.08
1053.50 1054.02 1054.27 1054.89 1058.10 1083.32
1110.36 1111.53 1113.21 1113.92 1114.27 1114.83
1116.99 1118.24 1118.62 1119.03 1120.71 1121.76
1122.26 1122.47 1133.56 1162.02 1177.35 1200.00
1200.63 1201.34 1201.51 1202.10 1203.18 1203.29
1203.53 1208.14 1217.61 1221.87 1221.99 1226.52

1228.79 1230.09 1230.54 1231.05 1233.44 1254.38
1287.54 1313.44 1324.43 1325.45 1327.09 1330.47
1332.24 1337.23 1337.84 1338.85 1347.10 1364.13
1364.43 1367.56 1369.69 1369.76 1371.24 1372.62
1395.76 1473.24 1474.77 1476.21 1477.54 1478.11
1481.32 1481.49 1481.81 1484.31 1492.73 1505.06
1505.67 1525.64 1526.30 1527.14 1528.12 1528.34
1528.81 1529.22 1531.99 1623.91 1625.99 1626.41
1626.54 1629.75 1631.52 1631.75 1643.17 1644.85
1645.49 1646.57 1647.64 1648.20 1650.84 1662.22
1763.51 1865.37 2985.24 3009.74 3031.69 3064.26
3080.80 3105.47 3121.64 3145.94 3178.20 3179.92
3181.50 3183.74 3183.85 3184.39 3185.78 3186.79
3187.41 3189.42 3189.45 3191.36 3192.39 3192.53
3193.31 3197.01 3199.33 3199.85 3199.88 3201.74
3204.73 3206.22 3208.81 3209.37 3209.77 3210.98
3211.54 3213.14 3213.23 3215.00 3216.32 3216.78
3219.19 3224.86 3226.90 3236.28 3240.62 3245.19

==============================

8A-TS1’’

==============================

-163.91 11.82 25.10 30.30 32.42 37.72
44.09 45.55 47.96 51.93 55.08 56.81
60.77 62.26 66.83 68.93 73.12 75.86
78.06 79.20 87.10 89.61 95.09 98.44
101.44 108.52 111.93 125.13 135.90 142.14
148.86 162.78 168.47 178.05 195.58 200.85
207.87 209.64 214.82 220.94 223.96 230.44
239.44 242.14 251.51 256.78 258.43 260.64
267.51 272.44 273.93 274.94 282.91 283.41
302.25 330.98 335.72 346.02 381.76 406.66
408.36 410.08 416.55 420.86 424.11 425.47
433.06 435.14 440.34 458.97 468.98 471.47
499.35 503.85 517.78 518.66 526.90 531.54
534.67 545.90 552.50 568.07 616.94 626.86
628.29 629.76 630.90 631.65 633.03 633.51
675.79 692.43 694.31 696.23 705.64 706.83
709.49 711.44 712.47 713.45 714.75 717.00
720.95 724.62 751.48 758.23 761.43 765.27
766.16 768.05 770.31 772.15 803.15 819.00
860.55 864.80 866.27 870.30 871.25 875.42
881.41 885.03 932.63 935.77 941.86 943.57
944.68 945.75 949.02 954.80 960.92 973.30
981.13 982.35 986.14 988.60 989.78 991.52
1001.02 1008.46 1011.80 1012.38 1012.80 1014.05
1014.08 1015.02 1015.99 1016.59 1017.87 1019.05
1020.80 1022.31 1022.85 1043.33 1049.05 1052.04
1052.98 1053.68 1054.57 1055.65 1062.78 1082.27
1107.52 1109.47 1111.42 1115.25 1115.31 1116.68
1116.85 1117.68 1119.36 1120.20 1120.67 1121.58
1123.23 1124.95 1135.57 1140.58 1165.58 1181.21
1191.55 1200.76 1202.18 1202.31 1203.09 1203.43
1204.08 1208.46 1220.01 1221.86 1226.80 1226.99
1229.96 1230.57 1235.53 1237.24 1258.95 1264.54
1290.25 1322.42 1322.76 1324.85 1327.53 1329.67
1331.82 1335.47 1337.31 1347.59 1350.63 1363.63
1365.92 1366.56 1368.87 1369.90 1374.92 1396.79
1414.19 1436.15 1473.95 1474.57 1476.18 1476.53
1479.95 1480.18 1480.60 1487.62 1497.13 1498.24
1503.65 1503.93 1506.36 1512.52 1525.21 1525.22
1526.45 1526.80 1527.81 1528.33 1528.87 1531.62
1626.51 1627.13 1627.96 1628.44 1629.91 1630.55
1644.49 1644.92 1645.44 1645.56 1646.51 1647.17
1664.47 1791.56 1972.17 2981.52 3022.22 3029.35
3050.76 3055.91 3058.90 3065.15 3068.75 3109.22
3119.32 3127.14 3132.66 3142.78 3146.32 3154.26
3155.03 3159.19 3179.16 3180.87 3183.65 3184.27
3186.69 3187.12 3187.63 3189.04 3189.66 3193.15
3194.08 3195.44 3196.11 3198.51 3198.76 3202.12
3202.43 3205.71 3206.16 3206.94 3209.54 3211.60
3212.12 3213.58 3214.46 3218.20 3222.79 3228.95
3236.64 3259.40 3271.74

==============================

8B-TS1’’

==============================

-51.77 15.44 19.09 30.35 34.67 36.73
44.22 45.09 48.59 50.46 53.08 55.93
56.92 58.51 61.95 65.11 68.21 75.89
79.39 82.77 90.69 94.37 96.93 99.87
100.99 111.40 113.94 119.40 133.52 139.04
140.23 145.63 150.90 163.42 177.60 198.91
206.15 209.34 215.74 225.85 229.18 230.79
235.77 243.85 253.20 255.79 257.17 262.52
263.77 270.00 278.40 281.73 282.94 289.53
304.48 309.68 331.52 361.30 399.79 408.24
410.75 413.18 413.99 420.79 423.21 427.60
433.87 439.41 440.98 463.88 466.96 469.67
503.63 508.29 515.25 519.02 526.88 533.40
535.01 558.95 565.26 595.26 613.75 626.18
627.89 629.68 630.28 631.22 631.57 633.60
673.95 680.83 696.23 698.45 707.09 707.57
711.48 713.00 714.21 714.34 716.20 717.82
720.90 721.74 746.69 761.58 763.11 763.73
764.71 766.20 767.79 770.36 809.40 820.33
862.47 864.76 867.46 871.16 874.63 875.71
877.23 879.44 937.25 939.31 942.79 944.29
944.43 946.23 950.37 953.57 958.01 974.73
981.86 984.42 986.03 988.17 991.45 992.17
995.92 1010.39 1011.85 1012.71 1013.10 1013.56
1013.87 1014.95 1016.18 1016.68 1017.15 1017.38
1018.36 1020.23 1021.54 1041.88 1050.49 1051.57
1052.32 1052.47 1053.24 1053.91 1060.49 1083.48
1106.22 1110.56 1113.46 1114.35 1115.18 1116.19
1116.90 1117.68 1117.96 1118.65 1119.98 1120.02
1121.68 1122.60 1142.20 1144.43 1162.15 1177.95
1192.19 1200.55 1200.93 1201.30 1202.04 1203.00
1203.56 1207.99 1216.02 1222.42 1222.84 1226.67
1227.11 1230.11 1230.76 1231.05 1253.98 1287.71
1294.75 1324.38 1325.06 1327.79 1329.52 1333.77
1337.54 1338.72 1342.80 1350.04 1355.21 1364.61
1365.10 1367.62 1367.87 1369.46 1371.30 1396.03
1411.58 1432.90 1472.68 1473.86 1477.04 1477.25
1479.26 1480.09 1481.00 1481.28 1495.28 1498.20
1505.30 1506.41 1507.60 1514.04 1525.45 1526.10
1527.00 1527.38 1527.79 1528.15 1528.54 1528.87
1623.86 1626.58 1628.04 1629.21 1631.28 1631.64
1642.02 1644.73 1644.88 1645.94 1647.17 1648.14
1679.31 1782.16 1934.68 2990.03 3009.38 3039.98
3047.40 3050.10 3065.94 3078.23 3106.02 3119.90
3121.70 3126.07 3130.60 3134.61 3149.09 3152.87
3179.52 3179.85 3180.52 3184.03 3184.37 3185.16
3185.37 3186.86 3188.86 3189.86 3189.90 3191.06

3193.26 3194.43 3197.42 3198.99 3199.92 3200.85
3202.70 3206.20 3208.48 3210.28 3211.30 3211.59
3211.83 3214.10 3214.72 3216.24 3216.97 3218.35
3219.23 3223.67 3255.31
==============================
15A-TS1’
==============================
-248.14 5.57 13.10 15.54 23.41 26.68
30.25 32.32 34.38 36.87 41.05 42.34
44.69 45.41 47.88 50.24 51.07 53.40
58.64 63.34 64.26 69.20 72.20 76.67
78.60 87.25 90.05 94.38 96.25 101.20
108.20 109.68 110.71 122.77 123.70 128.71
131.43 141.10 143.84 151.44 158.83 162.51
162.84 166.13 172.08 174.68 178.45 181.93
185.76 190.48 192.68 196.52 197.25 201.24
208.48 210.38 218.72 221.93 226.60 230.39
236.01 238.46 242.47 244.21 253.99 259.71
264.14 269.83 270.99 280.18 286.50 286.61
292.17 295.59 300.12 309.54 324.27 331.34
337.90 344.34 375.58 396.39 401.48 412.66
417.15 418.93 423.49 437.08 443.00 444.59
448.57 459.30 467.98 484.25 485.65 489.91
496.76 499.23 516.87 519.05 519.56 522.73
524.42 525.11 525.33 528.02 531.66 532.67
533.86 538.11 540.17 540.51 544.78 545.82
552.29 557.58 560.44 563.08 565.16 569.07
574.55 583.15 598.62 611.02 618.55 621.10
631.43 632.89 639.43 659.22 677.71 683.08
685.00 688.49 703.23 705.84 711.11 713.67
714.71 718.25 756.91 768.36 769.37 779.11
780.67 792.05 802.51 808.04 814.90 830.49
840.66 841.92 853.92 856.96 860.23 862.48
862.85 867.49 871.23 873.59 877.56 878.57
880.29 891.55 893.15 895.30 895.77 897.95
909.49 916.06 919.02 921.74 931.35 934.18
936.93 939.50 947.93 958.18 960.40 962.52
964.08 967.20 968.46 972.64 974.80 983.00
993.35 997.34 1004.95 1007.60 1008.27 1009.82
1011.08 1011.79 1012.12 1013.36 1013.92 1016.77
1023.54 1024.21 1025.56 1027.10 1027.80 1033.28
1041.69 1046.13 1047.73 1050.98 1054.12 1058.72
1061.07 1063.40 1064.48 1065.41 1066.37 1066.97
1067.59 1067.78 1069.14 1069.48 1076.60 1088.20
1115.19 1131.39 1140.38 1150.15 1159.51 1161.10
1163.68 1166.40 1173.26 1174.50 1176.41 1180.05
1188.66 1190.69 1196.03 1200.23 1201.88 1203.15
1204.58 1206.43 1206.58 1208.01 1213.43 1215.44
1218.70 1225.92 1241.12 1247.52 1256.45 1259.91
1276.35 1286.88 1289.42 1290.70 1294.53 1297.29
1302.75 1303.95 1316.63 1317.98 1321.24 1325.21
1326.08 1333.86 1337.88 1339.96 1349.67 1355.50
1359.81 1360.94 1362.35 1370.22 1396.80 1398.49
1406.62 1410.33 1426.37 1427.85 1428.79 1429.19
1429.97 1430.40 1430.89 1431.51 1434.47 1455.59
1458.49 1461.46 1462.33 1463.64 1464.15 1464.82
1465.99 1467.98 1469.94 1480.11 1482.04 1494.07
1494.30 1495.69 1496.51 1497.48 1499.99 1500.35
1500.51 1501.80 1504.42 1504.46 1505.26 1509.85
1510.37 1511.08 1512.21 1513.98 1514.45 1519.70
1521.50 1525.11 1525.20 1526.33 1533.45 1548.42
1552.91 1595.93 1610.65 1623.91 1633.55 1634.57
1637.96 1638.11 1644.46 1646.54 1647.15 1647.79
1648.19 1649.28 1650.33 1650.61 1671.84 1674.04
1770.97 1926.38 2956.95 2991.84 3007.59 3035.04
3036.48 3040.07 3040.46 3040.87 3041.07 3041.42
3042.25 3042.44 3067.23 3098.01 3100.09 3100.84
3101.06 3102.72 3104.10 3104.33 3104.51 3110.43
3111.52 3124.61 3125.78 3128.36 3131.38 3131.84
3132.20 3132.52 3135.21 3152.63 3161.50 3165.14
3167.42 3168.25 3174.92 3177.18 3180.20 3181.58
3182.02 3182.11 3184.43 3185.63 3186.05 3191.89
3192.14 3192.27 3192.79 3195.55 3196.62 3201.17
3207.02 3207.44 3207.56 3207.92 3216.24 3218.10
3220.33 3220.68 3224.48 3224.61 3230.12 3231.59
==============================
15B-TS1’
==============================
-304.40 7.43 11.31 15.09 18.54 22.66
26.22 29.73 31.25 35.01 37.57 41.62
44.10 46.61 47.48 49.97 51.34 53.78
56.33 59.71 66.25 68.40 70.07 73.26
75.90 79.99 82.49 85.71 91.29 94.39
97.85 102.18 105.73 108.66 110.66 127.23
129.25 135.60 142.27 148.41 150.23 150.95
159.15 164.65 166.35 173.78 176.77 179.89
184.33 185.98 187.77 192.09 197.98 202.03
203.62 205.08 211.73 216.40 222.91 226.07
232.66 233.48 236.68 241.92 249.90 252.84
256.48 259.42 268.80 273.96 283.34 288.12
289.85 292.87 301.06 307.56 310.39 323.13
334.70 345.59 368.32 394.59 398.88 413.25
414.20 417.96 432.22 436.50 442.51 446.73
449.38 458.29 463.61 480.40 485.87 493.35
495.18 499.19 510.36 519.04 520.54 521.26
522.70 523.65 524.45 526.78 529.00 529.78
530.50 532.57 535.74 544.72 549.21 552.02
553.30 553.75 560.24 562.45 564.19 566.74
568.15 578.85 595.98 597.72 614.21 620.44
626.39 635.31 638.48 657.65 669.30 679.20
684.53 691.06 701.97 705.74 707.94 710.21
713.38 716.12 754.80 763.45 768.62 776.10
777.16 787.80 789.65 803.45 814.06 832.52
841.09 842.49 852.21 855.32 861.72 862.24
863.43 866.66 871.22 873.21 873.51 876.42
880.70 883.39 889.12 894.77 900.93 901.96
906.06 907.39 910.97 921.10 923.25 928.55
933.39 935.38 940.78 942.77 957.74 959.76
962.64 964.11 969.34 969.91 972.14 984.65
992.88 993.39 1003.39 1004.04 1005.09 1005.99
1008.29 1008.81 1011.83 1012.83 1013.15 1014.48
1024.27 1024.48 1024.81 1025.23 1026.80 1032.52
1043.40 1044.27 1048.14 1048.61 1055.59 1057.73
1058.27 1065.20 1065.41 1065.54 1066.65 1069.05
1069.29 1070.02 1070.70 1071.06 1075.98 1095.22
1116.11 1138.26 1140.76 1155.89 1156.68 1159.57
1165.26 1166.84 1167.30 1173.99 1175.57 1176.26
1187.62 1198.51 1199.36 1200.39 1202.62 1203.69
1204.38 1206.10 1206.96 1210.40 1214.83 1217.60
1221.77 1227.79 1242.89 1255.71 1256.49 1259.09
1280.31 1286.98 1288.67 1288.96 1289.59 1295.19
1300.92 1307.87 1317.66 1320.24 1322.04 1326.77

1327.42 1330.65 1346.01 1351.08 1351.22 1358.67
1359.25 1362.37 1364.11 1365.02 1386.40 1395.33
1408.14 1409.24 1425.30 1427.02 1427.35 1429.56
1429.73 1430.35 1430.79 1431.86 1431.95 1453.25
1459.25 1460.71 1462.35 1463.09 1464.98 1465.41
1465.76 1466.08 1468.40 1479.56 1480.76 1493.37
1494.94 1495.63 1497.04 1497.55 1498.31 1499.13
1499.57 1501.20 1501.87 1503.09 1504.56 1505.71
1507.78 1509.85 1511.26 1512.31 1512.64 1520.12
1520.55 1521.91 1522.71 1527.04 1529.73 1548.21
1551.49 1594.68 1602.36 1619.91 1634.83 1635.31
1635.87 1636.86 1638.20 1639.06 1648.35 1648.99
1649.77 1651.28 1652.57 1653.95 1671.31 1672.68
1777.66 1863.85 2968.24 2984.33 3006.33 3026.08
3038.69 3039.61 3039.95 3040.25 3040.99 3043.94
3049.67 3064.43 3068.98 3093.60 3098.96 3099.39
3099.59 3101.85 3104.91 3109.35 3111.16 3113.22
3116.10 3123.26 3124.20 3125.78 3126.55 3127.38
3127.76 3129.90 3136.57 3145.16 3166.34 3169.65
3170.51 3172.52 3178.98 3179.90 3180.15 3182.22
3182.62 3183.38 3184.01 3184.38 3187.31 3190.42
3191.12 3191.19 3191.83 3192.92 3193.51 3193.97
3203.32 3203.79 3206.07 3206.61 3211.26 3214.48
3215.60 3226.85 3227.08 3228.61 3235.00 3257.41
==============================
15A-TS1''
==============================
-249.25 3.36 8.71 12.01 22.48 25.43
29.60 32.07 33.96 35.53 37.98 42.67
45.52 47.78 49.54 52.57 55.33 56.31
57.30 59.90 64.37 66.11 71.87 74.69
87.19 90.61 96.84 97.97 103.49 106.02
108.48 110.27 113.38 116.67 127.82 133.94
140.81 143.76 154.19 157.20 160.64 164.77
167.08 170.49 171.89 176.68 185.58 188.04
191.40 193.07 195.33 196.33 199.61 206.01
210.33 217.85 220.55 222.63 231.91 232.08
235.11 242.87 245.56 248.13 253.81 257.99
260.09 264.40 271.55 280.05 281.74 286.41
286.57 291.36 293.86 299.89 309.04 324.07
327.25 333.51 344.79 352.03 383.26 395.56
399.47 412.17 418.20 436.76 443.62 446.27
448.91 458.76 466.66 483.01 485.92 486.60
489.36 498.01 499.88 515.63 517.17 519.56
520.63 522.85 524.89 526.36 527.13 530.90
532.00 532.94 539.73 541.76 545.18 546.69
552.88 557.28 559.23 562.73 563.80 568.06
574.22 579.65 584.74 598.32 617.80 619.47
628.85 637.18 658.47 682.16 683.19 684.61
686.56 704.08 708.43 713.70 714.67 718.32
759.53 767.93 768.73 777.41 787.48 803.46
804.05 810.78 830.61 839.81 840.27 853.63
855.23 860.80 863.23 865.68 871.58 872.72
876.42 878.30 879.02 881.07 891.07 891.90
896.01 899.22 900.82 909.85 916.25 919.73
931.97 934.66 936.39 939.03 942.79 952.32
957.24 959.47 960.84 963.38 967.02 968.49
972.22 972.84 975.79 992.70 993.92 1005.53
1007.83 1008.12 1009.99 1010.35 1011.52 1012.48
1013.91 1023.88 1024.52 1025.55 1026.82 1030.61
1036.62 1042.29 1046.33 1047.66 1050.44 1055.64
1058.96 1063.63 1064.39 1065.43 1066.25 1066.74
1067.73 1067.87 1068.99 1069.43 1077.01 1087.09
1122.78 1130.45 1132.59 1142.09 1149.82 1159.54
1161.46 1164.85 1167.51 1172.53 1174.36 1176.36
1180.12 1188.58 1191.60 1198.70 1201.85 1203.55
1204.91 1207.66 1207.71 1208.25 1214.17 1215.22
1218.08 1240.47 1245.68 1255.41 1258.93 1259.81
1288.07 1289.11 1291.33 1294.54 1296.08 1301.65
1303.69 1316.63 1316.99 1319.18 1321.30 1325.38
1325.89 1339.03 1341.20 1349.95 1356.47 1359.79
1360.56 1362.74 1370.45 1396.80 1397.61 1405.35
1409.98 1412.96 1426.15 1427.88 1428.82 1429.17
1429.88 1430.36 1430.95 1431.62 1434.50 1434.95
1456.13 1458.87 1461.66 1462.19 1462.77 1464.34
1465.14 1466.13 1467.80 1469.92 1479.44 1494.19
1494.27 1495.62 1496.48 1497.42 1499.36 1499.42
1499.80 1500.07 1502.90 1503.20 1503.30 1504.44
1505.68 1509.51 1510.52 1511.13 1511.81 1513.54
1513.82 1514.48 1520.02 1521.36 1523.58 1524.94
1527.11 1532.90 1548.73 1551.84 1597.42 1610.55
1635.39 1635.50 1637.80 1638.23 1643.38 1646.50
1648.82 1648.97 1650.17 1650.85 1655.69 1670.47
1673.95 1770.45 1921.11 2955.03 2990.27 3010.12
3036.04 3039.63 3040.46 3040.75 3041.10 3041.29
3041.37 3041.59 3042.69 3049.86 3053.03 3067.25
3068.04 3098.14 3099.64 3101.15 3101.23 3102.80
3103.73 3103.73 3104.74 3111.18 3115.35 3124.67
3125.10 3125.75 3127.53 3128.15 3128.46 3130.57
3130.64 3132.18 3135.02 3138.33 3153.46 3154.71
3162.26 3165.52 3166.15 3167.39 3174.94 3175.20
3180.55 3180.65 3182.58 3183.01 3183.86 3188.22
3190.03 3191.42 3192.36 3193.09 3193.57 3197.77
3206.54 3206.90 3208.26 3214.13 3215.48 3217.57
3221.11 3222.74 3232.87
==============================
15B-TS1''
==============================
-314.32 8.72 17.25 22.20 24.47 26.71
30.02 34.20 36.44 40.07 41.75 43.07
46.08 50.57 51.13 52.22 53.19 56.34
57.97 63.51 66.15 71.43 75.57 76.71
81.62 88.65 90.88 99.22 99.87 103.00
106.58 106.99 111.34 113.86 118.23 123.16
136.55 141.37 143.39 149.61 154.12 155.29
159.24 164.83 173.51 175.25 182.53 183.71
186.12 189.56 196.99 198.25 200.48 202.80
210.59 217.55 219.38 221.06 227.02 230.67
234.91 239.57 243.62 247.05 253.14 255.53
256.28 267.48 269.01 271.93 279.03 283.26
288.35 291.14 293.41 300.26 306.65 307.75
323.31 326.82 333.09 342.76 395.88 398.31
399.24 412.73 417.36 430.45 445.17 445.86
448.61 458.58 463.50 480.79 485.79 492.05
493.07 499.52 514.93 517.59 520.26 520.99
522.94 524.68 526.00 528.19 530.83 532.70
534.66 537.57 537.96 545.04 547.51 553.30
556.25 557.07 558.56 563.25 567.13 568.70
570.22 575.47 579.31 598.24 618.20 620.34
623.55 633.61 650.94 657.18 674.04 684.58
690.77 703.93 704.98 710.49 713.60 716.63
762.73 765.31 768.49 775.42 789.05 791.61

| | | | | | |
|---|---|---|---|---|---|
| 804.24 | 813.96 | 828.73 | 839.21 | 840.37 | 846.01 |
| 856.04 | 862.47 | 864.22 | 865.19 | 871.55 | 871.85 |
| 875.86 | 877.12 | 879.28 | 881.28 | 884.66 | 888.08 |
| 894.79 | 897.10 | 903.36 | 906.47 | 906.89 | 922.48 |
| 926.95 | 930.78 | 933.41 | 936.67 | 948.99 | 950.85 |
| 952.80 | 957.94 | 962.16 | 962.58 | 965.18 | 965.71 |
| 969.07 | 972.58 | 976.00 | 987.98 | 991.97 | 1002.04 |
| 1002.54 | 1002.87 | 1006.51 | 1009.68 | 1011.96 | 1012.82 |
| 1013.97 | 1023.36 | 1023.69 | 1025.05 | 1026.10 | 1026.51 |
| 1038.72 | 1042.21 | 1047.17 | 1048.88 | 1049.52 | 1055.89 |
| 1057.82 | 1063.73 | 1064.75 | 1066.07 | 1066.48 | 1066.73 |
| 1067.04 | 1067.63 | 1069.15 | 1070.39 | 1077.21 | 1094.36 |
| 1122.18 | 1131.93 | 1136.34 | 1139.69 | 1155.18 | 1157.49 |
| 1162.38 | 1165.28 | 1167.17 | 1168.02 | 1173.73 | 1173.78 |
| 1175.89 | 1188.20 | 1193.23 | 1199.42 | 1203.92 | 1204.22 |
| 1204.32 | 1205.83 | 1207.20 | 1210.40 | 1212.33 | 1214.48 |
| 1227.72 | 1244.19 | 1255.73 | 1259.11 | 1262.23 | 1276.58 |
| 1288.69 | 1290.85 | 1290.89 | 1292.60 | 1295.04 | 1301.29 |
| 1312.63 | 1317.19 | 1320.28 | 1321.51 | 1324.92 | 1330.64 |
| 1333.28 | 1347.77 | 1351.14 | 1354.07 | 1358.14 | 1361.05 |
| 1362.11 | 1364.25 | 1367.52 | 1388.51 | 1396.50 | 1408.58 |
| 1409.88 | 1412.52 | 1425.22 | 1425.70 | 1428.34 | 1429.71 |
| 1429.95 | 1430.14 | 1431.29 | 1432.73 | 1433.13 | 1433.51 |
| 1452.64 | 1459.68 | 1461.63 | 1462.98 | 1463.97 | 1464.90 |
| 1465.10 | 1466.39 | 1466.52 | 1467.06 | 1478.86 | 1488.60 |
| 1491.42 | 1494.52 | 1496.21 | 1497.19 | 1498.68 | 1499.05 |
| 1500.04 | 1500.86 | 1501.42 | 1502.81 | 1503.03 | 1503.55 |
| 1504.43 | 1505.17 | 1506.90 | 1510.05 | 1511.01 | 1512.58 |
| 1513.04 | 1514.04 | 1519.42 | 1521.29 | 1521.43 | 1522.44 |
| 1524.39 | 1528.80 | 1548.58 | 1551.32 | 1596.81 | 1602.73 |
| 1635.22 | 1636.55 | 1637.03 | 1637.74 | 1637.96 | 1639.14 |
| 1648.29 | 1649.34 | 1650.39 | 1650.82 | 1651.28 | 1671.50 |
| 1672.58 | 1776.73 | 1844.66 | 2979.62 | 2992.88 | 3002.25 |
| 3036.37 | 3039.09 | 3039.64 | 3041.03 | 3041.30 | 3041.82 |
| 3043.90 | 3045.33 | 3046.04 | 3050.42 | 3066.16 | 3075.48 |
| 3096.29 | 3096.66 | 3100.81 | 3101.16 | 3101.71 | 3102.84 |
| 3106.12 | 3111.01 | 3112.66 | 3114.88 | 3116.74 | 3120.51 |
| 3122.14 | 3122.97 | 3125.07 | 3125.64 | 3126.25 | 3127.55 |
| 3128.60 | 3132.55 | 3135.74 | 3136.01 | 3147.33 | 3148.11 |
| 3164.87 | 3167.75 | 3168.57 | 3175.00 | 3178.30 | 3179.26 |
| 3181.16 | 3183.06 | 3185.33 | 3187.46 | 3188.15 | 3191.62 |
| 3191.76 | 3193.45 | 3194.06 | 3194.76 | 3196.09 | 3199.86 |
| 3200.95 | 3201.63 | 3205.40 | 3206.28 | 3215.92 | 3216.61 |
| 3224.61 | 3229.35 | 3232.52 | | | |
